# Unusual mechanisms in Claisen rearrangements: an ionic fragmentation leading to a *meta*-selective rearrangement†
†Electronic supplementary information (ESI) available: Experimental details, full characterization for new compounds, spectral data and computational details. CCDC 1540803, 1540804 and 1568395. For ESI and crystallographic data in CIF or other electronic format see DOI: 10.1039/c7sc04736c


**DOI:** 10.1039/c7sc04736c

**Published:** 2018-04-10

**Authors:** Boris Maryasin, Dainis Kaldre, Renan Galaverna, Immo Klose, Stefan Ruider, Martina Drescher, Hanspeter Kählig, Leticia González, Marcos N. Eberlin, Igor D. Jurberg, Nuno Maulide

**Affiliations:** a University of Vienna , Faculty of Chemistry , Institute of Organic Chemistry , Währinger Strasse 38 , 1090 Vienna , Austria . Email: nuno.maulide@univie.ac.at ; Email: boris.maryasin@univie.ac.at; b University of Vienna , Faculty of Chemistry , Institute of Theoretical Chemistry , Währinger Strasse 17 , 1090 Vienna , Austria; c State University of Campinas , ThoMSon Mass Spectrometry Laboratory , Institute of Chemistry , Rua Monteiro Lobato 270 , 13083-970 , Campinas , Brazil; d State University of Campinas , Institute of Chemistry , Rua Monteiro Lobato 270 , 13083-970 , Campinas , Brazil . Email: idjurberg@iqm.unicamp.br

## Abstract

A mechanistic investigation of the acid-catalysed redox-neutral oxoarylation reaction of ynamides using electrospray ionisation mass-spectrometry (ESI-MS) and quantum chemical calculations (DFT and MP2) is presented. This study reveals the diversity of pathways and products available from an otherwise deceptively simple-looking, classical transformation: fragmentation, an unusual *meta*-arylation and competing α-carbonyl cation pathways are some of the alternatives unveiled by ESI-MS and mechanistic experiments. Detailed calculations explain the observed trends and rationalise the results.

## Abstract 2

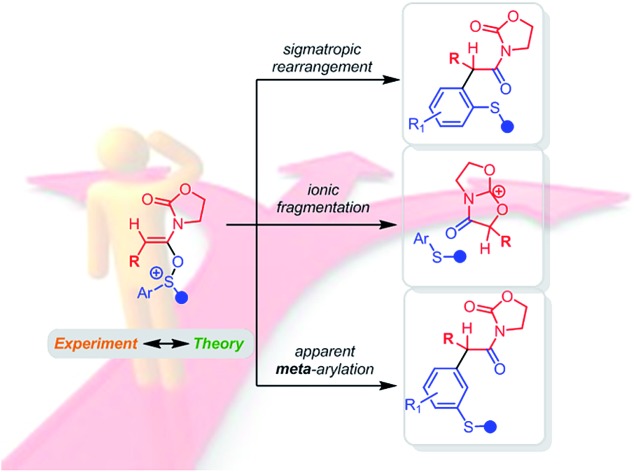
A mechanistic investigation of the acid-catalysed redox-neutral arylation of ynamides intertwining ESI-MS, DFT and experiments reveals diverse pathways available from an otherwise simple-looking transformation.

## Introduction

**Scheme 1:**
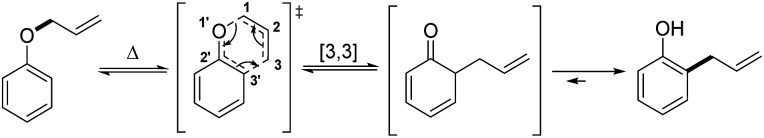
The Claisen rearrangement originally reported in 1912.

The [3,3]-sigmatropic rearrangement of vinyl(allyl)ethers was first introduced in 1912 by the seminal work of L. Claisen, whereby *O*-allyl phenols were thermally converted into 2-allyl phenols (Scheme 1).^1^ Inspired by this contribution, in the following years several studies were reported expanding the scope of this transformation into a powerful and versatile C–C bond forming strategy.^2^ Indeed, a list of well-known examples of such variants now hold “textbook status” in the field of organic chemistry, including those developed by Ireland,^3^ Johnson,^4^ Eschenmoser,^5^ Overman,^6^ and Ficini,^7^ among others.^8^

**Scheme 2:**
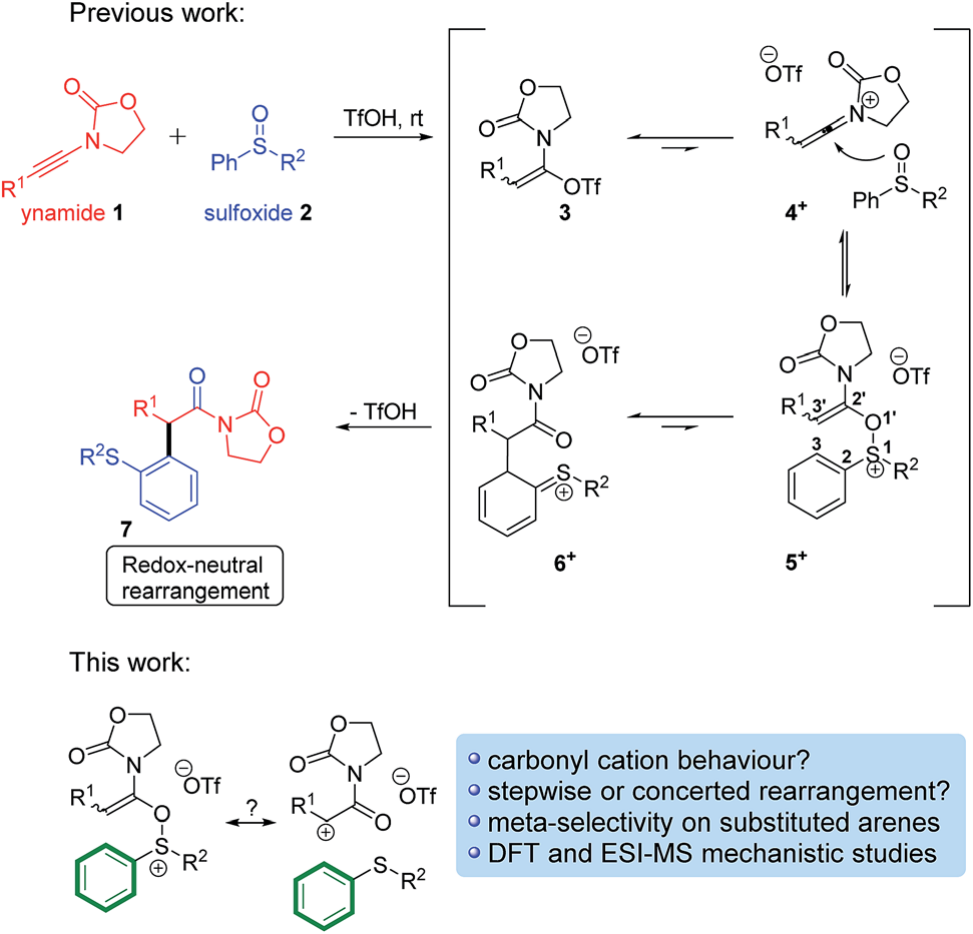
The redox-neutral oxoarylation reaction previously reported^10^ and mechanistic questions addressed in this manuscript.

More recently, one of us^9^ has been interested in the development of novel variants of this venerable reaction that can be deployed in alternative contexts. Maulide and co-workers thus described a TfOH-catalysed oxoarylation of ynamides **1** using sulfoxides **2**. Mechanistic analysis assumed the involvement of a [3,3]-sigmatropic shift analogous to the Claisen rearrangement, most likely taking place on cationic intermediate **5^+^**, *en route* to the corresponding final α-arylated acyloxazolidinone **7** (Scheme 2, top).^10^

Interestingly, whereas the latter rearrangement proceeds at temperatures below and at room temperature, typically in no more than 30 minutes reaction time, and in high yields, the analogous Ficini–Claisen rearrangement generally requires higher temperatures, longer reaction times and generally produces lower yields of the corresponding final alkylated products.^7^

Intrigued by this great difference in reactivity and in order to gather more information concerning the mechanism of this formal α-arylation reaction, we became interested in monitoring this transformation online. Given the abundance of (postulated) positively charged reaction intermediates along the reaction pathway, ESI-MS^11^ emerged as a promising tool to observe and intercept those species directly. Additionally, we aimed to complement those studies with quantum chemical calculations and additional experiments. Herein, we report the results of this study, culminating in a unified mechanistic view of this transformation. On the way to that goal, several unusual, unexpected alternative mechanistic pathways (Scheme 2, bottom) emerged showcasing the complex reactivity network in which this system is embedded.

## Discussion of results

### Analysis of reaction intermediates by online ESI(+)-MS

Based on the putative mechanism outlined in Scheme 2, we were eager to verify whether certain key charged intermediates [**3** + Y]^+^ (Y = H, Na), **4^+^**, **5^+^** and **6^+^** (Scheme 2) could be intercepted and characterised *via* electrospray ionisation MS operated in positive-ion mode (ESI(+)-MS).^12^

**Fig. 1:**
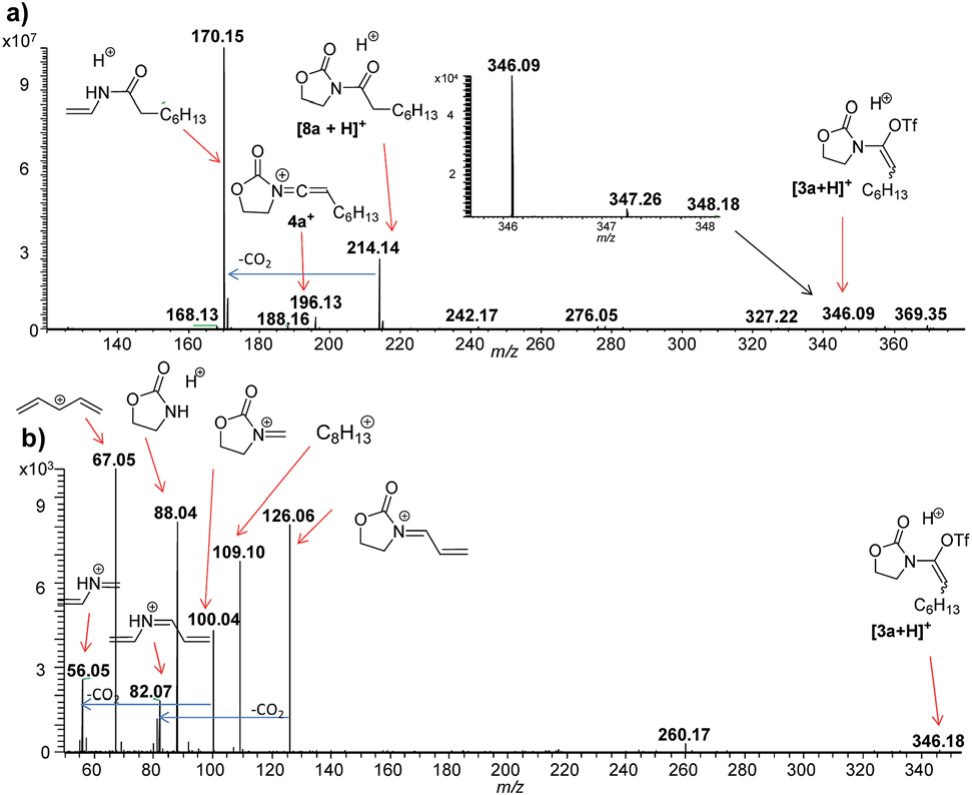
ESI(+)-MS monitoring of the reaction solution containing ynamide **1a** and TfOH in DCM. (a) DESI-MS(+) spectrum. (b) DESI(+)-MS/MS of the ion of *m*/*z* 346.09, attributed to [**3a** + H]^+^.

Initially, we were curious to verify whether, in the absence of sulfoxide **2**, a cationic form of the enamine-triflate [**3** + Y]^+^ (Y = H, Na) could be detected. Nevertheless, initial attempts employing ynamide **1a** (*cf.***1**, with R^1^ = *n*-C_6_H_13_) failed. As the corresponding protonated amide [**8a** + H]^+^, of *m*/*z* 214, was typically the main fragment observed, we rationalised that the timeframe between sample preparation and injection in the mass spectrometer might be too long for the detection of transient [**3a** + Y]^+^. Thus, we switched to DESI(+)-MS,^13^ in order to achieve quasi simultaneous sample preparation and MS analysis. For this purpose, a solution of triflic acid (1 mmol) in DCM (500 μL) was used for the DESI spray, whereas ynamide **1a** (1 μL) was deposited on a glass surface. While the spray droplets slowly mixed the reagents on the glass surface (10 μL min^–1^), the resulting intermediate [**3a** + Y]^+^ was simultaneously transferred to the gas phase. Using this approach, we were indeed able to detect intermediate [**3a** + H]^+^, as well as **4a^+^** at room temperature. The low abundances obtained for these ions are presumably due to their transient nature (Fig. 1).

Being able to detect the key intermediates [**3a** + H]^+^ and **4a^+^** in the presence of TfOH, we then turned our attention to the action of other common Brønsted acids: PhCO_2_H, pTSA and (PhO)_2_P(O)OH. Interestingly, whereas PhCO_2_H barely reacts with either **1a** or **1b** (*cf.***1**, with R^1^ = Ph), with the major fraction of the ynamides remaining unreacted, extensive addition of pTSA and (PhO)_2_P(O)OH to **1a** and **1b** is observed. As a consequence, these three acids are not capable of promoting this Claisen rearrangement (see ESI† for details).

**Fig. 2:**
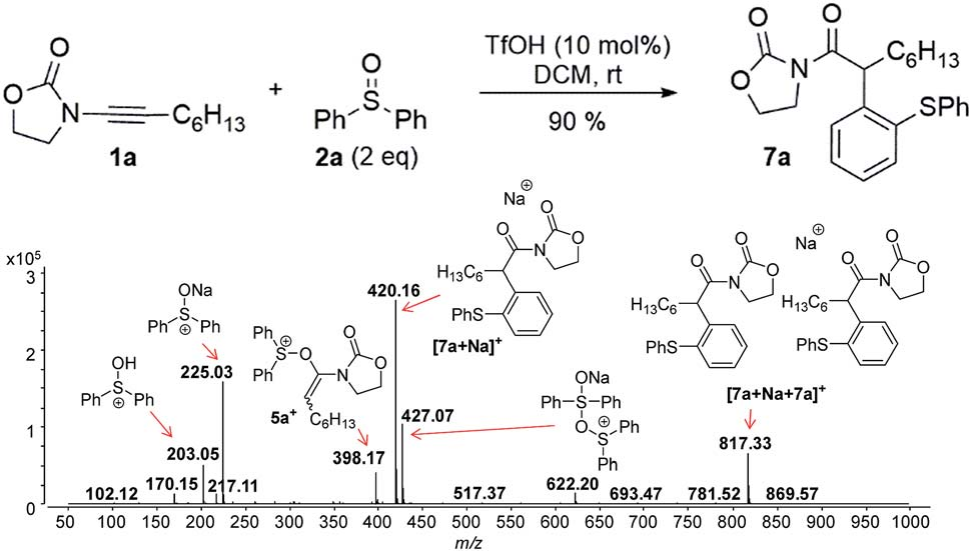
ESI(+)-MS recorded immediately after ynamide **1a** and diphenyl sulfoxide **2a** are mixed in the presence of a catalytic amount of TfOH in DCM.

Next, we investigated the TfOH-catalysed arylation of ynamide **1a** with diphenyl sulfoxide **2a** by ESI(+)-MS. The MS-spectra recorded immediately after the preparation of the reaction mixture already exhibited the key intermediate **5a^+^**, and the final sodiated aryl amide [**7a** + Na]^+^ (Fig. 2).

**Fig. 3:**
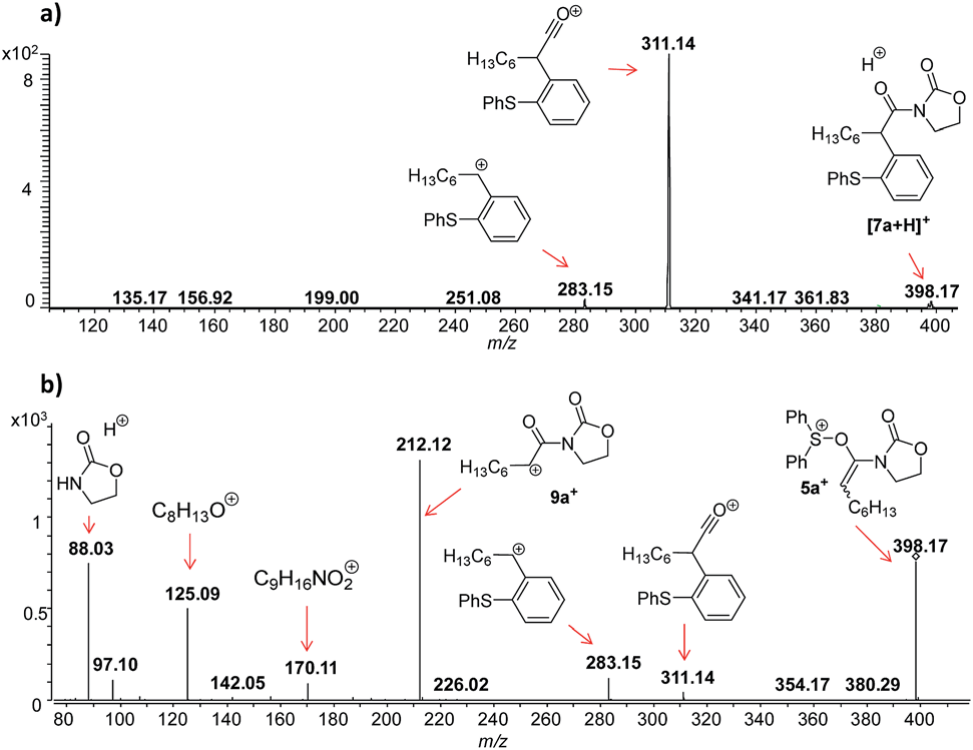
ESI(+)-MS/MS of the ion of *m*/*z* 398 analyzed from (a) isolated [**7a** + H]^+^, and (b) from the reaction mixture, which is clearly not identical with (a) and can be assigned to **5a^+^**.

**Fig. 4:**
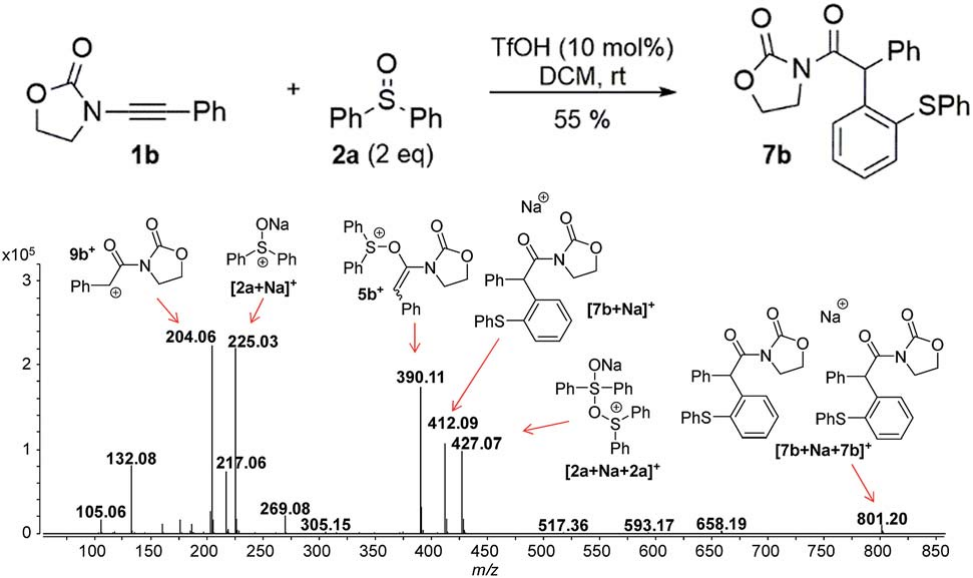
Full ESI-MS(+) immediately recorded after ynamide **1b** and diphenyl sulfoxide **2a** are mixed in the presence of a catalytic amount of TfOH in DCM.

**Fig. 5:**
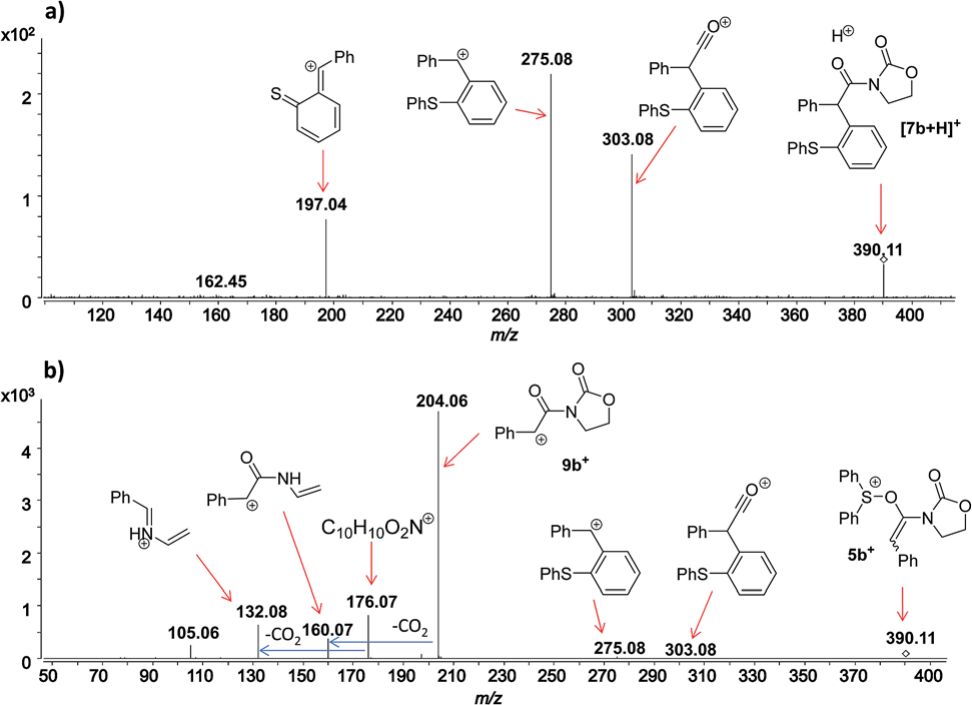
ESI(+)-MS/MS of the ion of *m*/*z* 390 analyzed from (a) isolated [**7b** + H]^+^, and (b) from the reaction mixture, which corresponds to **5b^+^**.

In this context, an important observation is that **7a** binds preferentially to Na^+^, rather than to H^+^ (due to the absence of strong Brønsted basic sites), and is largely detected as [**7a** + Na]^+^ of *m*/*z* 420. Furthermore, although **5a^+^** and [**7a** + H]^+^ are isomers of *m*/*z* 398, they can be distinguished by comparing the ESI(+)-MS/MS spectra obtained from isolated **7a** and intermediate **5a^+^** detected from the reaction mixture. Indeed, the fragmentation patterns are found to be remarkably different. The ESI(+)-MS/MS of [**7a** + H]^+^ of *m*/*z* 398 (acquired from the isolated product) shows major fragment ions of *m*/*z* 283, 311 and 398, whereas the MS-spectrum of **5a^+^**, acquired from the reaction mixture, mainly contains the fragment ions of *m*/*z* 88, 125, 170, 212, 283, 311 and 398 (Fig. 3). Furthermore, the reaction of ynamide **1a** (R^1^ = *n*-C_6_H_13_) with a different sulfoxide **2b** (R^2^ = Me) was also studied, providing similar observations (see ESI† for the corresponding spectra). Under the same experimental conditions, a second reaction involving ynamide **1b** (R^1^ = Ph) was studied. This time, not only intermediate **5b^+^** and the final product [**7b** + Na]^+^ can be observed in the full MS spectrum, but the prominent detection of a carbenium intermediate **9b^+^** (*m*/*z* 204) is a remarkable observation (Fig. 4). In contrast to the previous analyses of **7a** (Fig. 2 and 3), the analogous **9a^+^** was only visible in the MS/MS spectrum of **5a^+^** (Fig. 3b). In the same manner as before, the comparison of MS/MS spectra associated to *m*/*z* 390, assigned to intermediate **5b^+^** and the protonated compound [**7b** + H]^+^ (spectra also acquired from isolated **7b**), allows for the discrimination among them. Indeed, fragments of *m*/*z* 105, 132, 160, 176 and 204 are only found for **5b^+^**, while the fragment of *m*/*z* 197 is unique for [**7b** + H]^+^ (Fig. 5).

### Theoretical study and auxiliary experiments

In order to explain the dependence of experimentally observed intermediates on reaction substrate, namely the marked presence of the cation **9b^+^** in the case of a phenyl-substituted ynamide, and to clarify the mechanism of the considered reactions, we have carried out quantum chemical calculations at the DFT (B3LYP-D3-SMD/6-31+G(d,p)) and MP2//DFT (RI-MP2-COSMO/def2-TZVP//B3LYP-D3-SMD/6-31+G(d,p)) levels of theory (see the ESI† for computational details).^14^

**Fig. 6:**
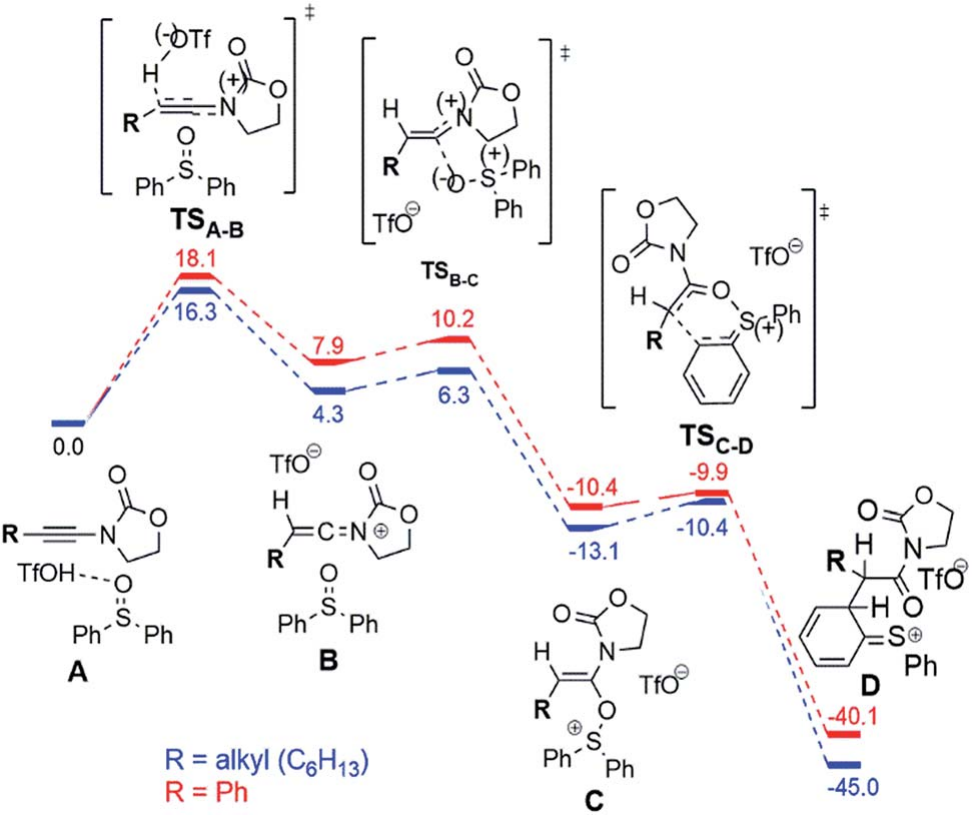
Computed reaction profile (DFT, Δ*G*_298,DCM_) for the conversion of ynamide (reactant complex **A**) into the intermediate **D** for the phenyl (red) and the alkyl (blue) substrates. The energy of the reactant complex **A** is taken as a reference (0.0 kcal mol^–1^).

Fig. 6 shows the computed reaction profile for conversion of the ynamide (**A**) into the cationic intermediate **D** with a characteristic loss of aromaticity.

The first step is formation of the transient keteniminium triflate **B** through protonation by TfOH. In the sequence, reaction with the sulfoxide yields the unusual N,O-ketene acetal **C**, that is further converted to intermediate **D**.

As can be seen, the aryl- and the alkyl ynamide substrates show similar behaviour in the formation of the intermediate **D** from the ynamide **A**. The reaction is exergonic for both substrates, and the barriers are relatively low, in agreement with the mild reaction conditions.

**Fig. 7:**
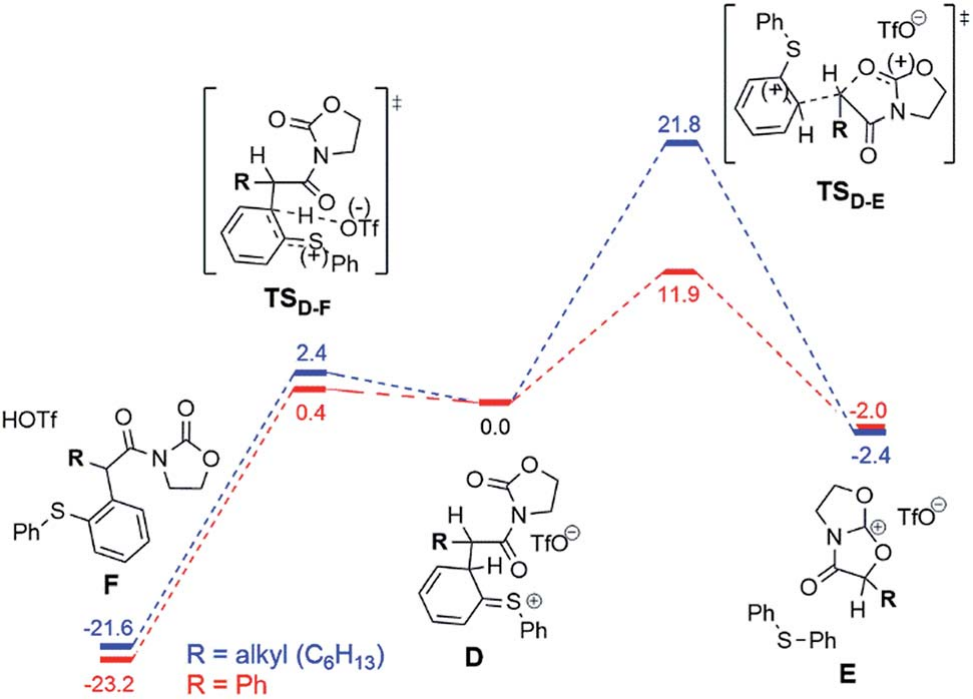
Computed reaction profile (DFT, Δ*G*_298,DCM_) for the conversion of the intermediate **D** into the main product **F** or the bicyclic cation **E** for the phenyl (red) and the alkyl (blue) substrates. The energy of the intermediate **D** is taken as a reference (0.0 kcal mol^–1^).

The situation changes substantially for the next steps, where intermediate **D** undergoes a conversion to the experimentally observed products. Two possible pathways were found and are presented in Fig. 7. The first scenario leads to the formation of the main product **F** (Fig. 7, left), while an alternative is the formation of the bicyclic cation **E** (Fig. 7, right). This cation was detected experimentally by ESI(+)-MS, but only for the phenyl-substituted ynamide (**E** is effectively the more stable form of the cation **9b^+^** shown in Fig. 5).^15^ Analysis of the relative Gibbs free energies presented in Fig. 7 provides a rationale. One can see that for both phenyl- and alkyl substituted intermediates **D**, the formation of the product **F** is energetically favourable, and therefore is experimentally observed for both systems. However, the barriers of the formation of the product **E** are very different for different substrates ΔΔ*G*‡298,DCM = Δ*G*‡298,DCM(alkyl) – Δ*G*‡298,DCM(phenyl) = 9.9 kcal mol^–1^ at the DFT or even 16.4 kcal mol^–1^ at the higher (MP2//DFT) level of theory. This substantial gap between the barriers and the large absolute value of the barrier for the alkyl-substituted system (21.8 kcal mol^–1^ at the DFT or 26.7 kcal mol^–1^ at the MP2//DFT level) is in good agreement with the experimental evidence, namely that the carbenium intermediate **9b^+^** is detected only in the case of the phenyl substituent.

**Fig. 8:**
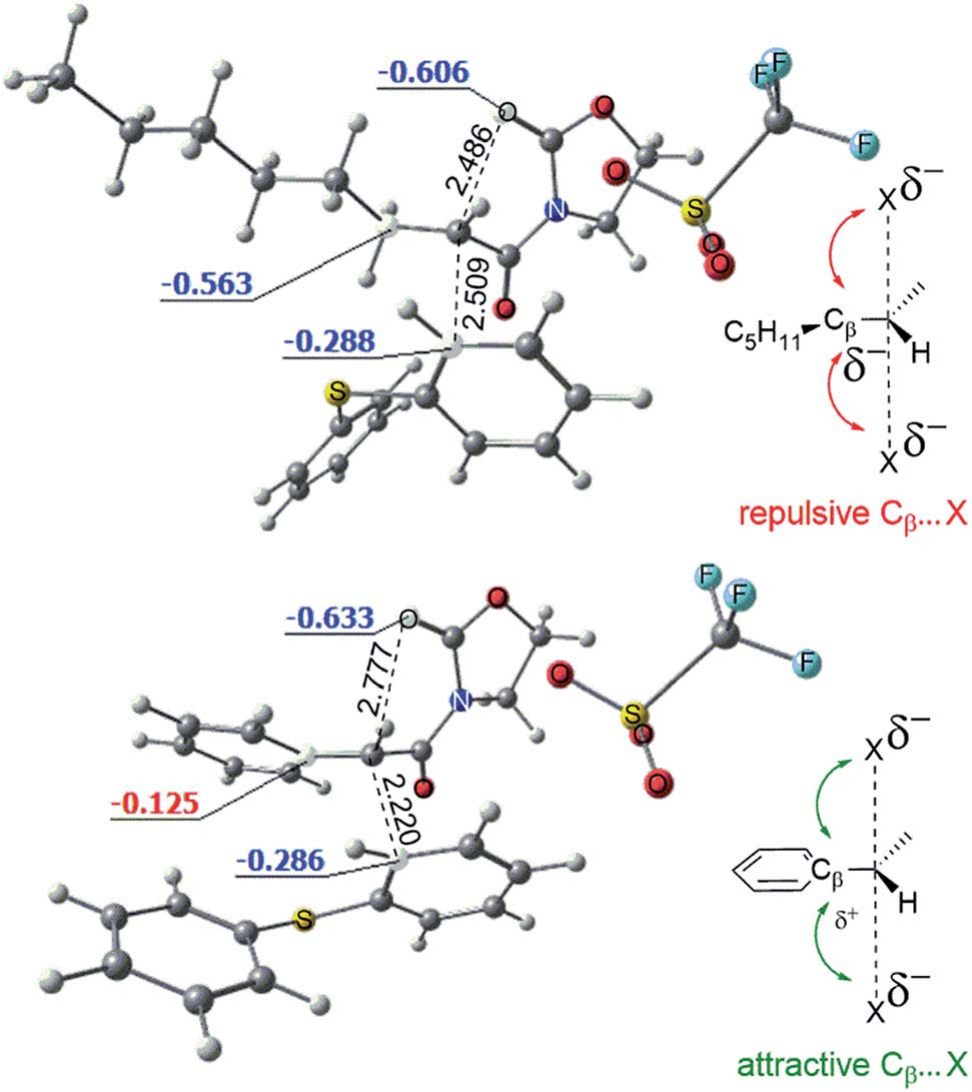
Optimized structures of the transition states **TS_D–E_** for the phenyl (bottom) and alkyl (top) substituted systems. Computed NBO charges of the selected atoms reveal the attractive and repulsive interactions in the transition states of the intramolecular S_N_2 reactions. The breaking and forming bonds are shown (Å).

The conversion of intermediate **D** into the bicycle **E** is an intramolecular S_N_2-type process. Fig. 8 shows the computed structures of the transition states **TS_D–E_** for phenyl (bottom) and alkyl (top) substituents, from which the typical trigonal bipyramidal structure of the S_N_2 transition state can be recognised.

Fig. 8 also depicts the calculated NBO (Natural Bond Orbital) charges for the atoms involved in the S_N_2 transformation. Electrostatic interactions within the transition state stabilise the phenyl variant of the **TS_D–E_** as compared to the alkyl **TS_D–E_**. As shown, attractive C_β_(δ^+^)–X(δ^–^) interactions in the phenyl-substituted structure stabilise the transition state (leading to a higher rate for the S_N_2 process). The importance of such electrostatic interactions for classical S_N_2 reactions was previously explored by Wu *et al.*^16^

**Fig. 9:**
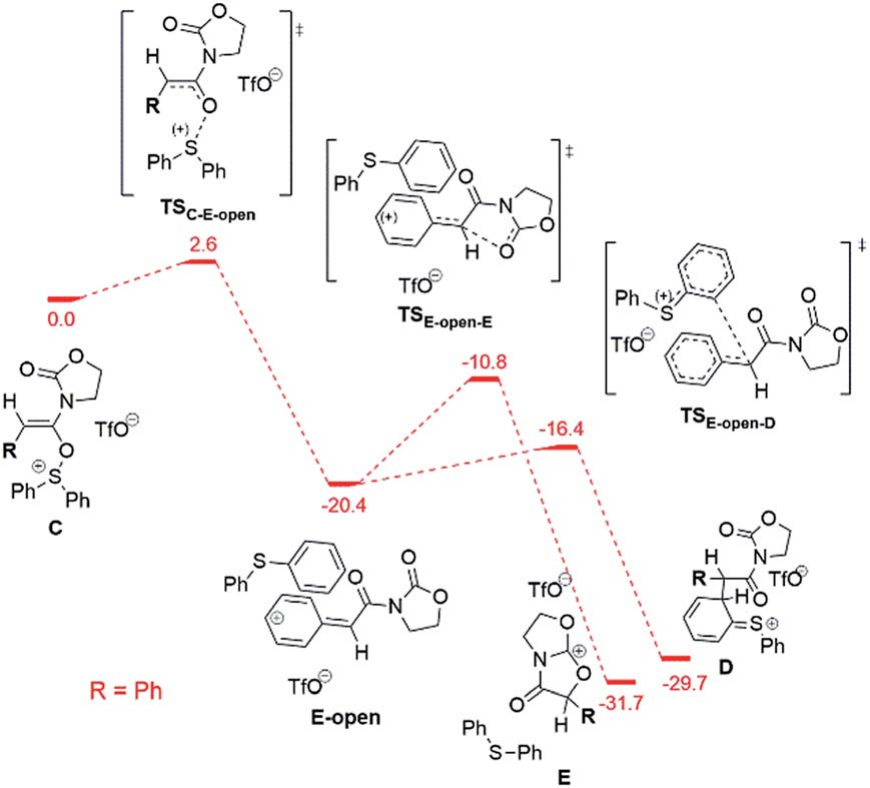
Computed reaction profile (DFT, Δ*G*_298,DCM_) for the conversion of the intermediate **C** into the bicyclic cation **E** for the phenyl substrate. The possible conversion of the “open” intermediate **E-open** to the intermediate **D** is also shown. The energy of the intermediate **C** is taken as a reference (0.0 kcal mol^–1^).

Alternatively, the bicyclic cation **E** can form directly from the intermediate **C** (before the sigmatropic rearrangement **C** → **D**) by a S_N_1-type reaction (Fig. 9).

This **C** → **E** conversion proceeds *via* an open form of the intermediate **9b^+^** (**E-open**), which undergoes annulation to **E** on the last step. The phenyl substituent stabilises the **E-open** cationic intermediate by hyperconjugation in a fashion not accessible to an alkyl group. Therefore, the S_N_1-like process **C** → **E** happens exclusively for the phenyl-substituted system, again in agreement with the experimental results (*vide supra*).

The barriers of the rate limiting steps of the S_N_2 and the S_N_1 processes for the phenyl substituted system are comparable with each other (11.9 kcal mol^–1^*vs.* 9.6 kcal mol^–1^). Thus, both pathways can be realised in the case of the phenyl substituent. However, the S_N_1 mechanism is more probable due to the low barrier of the **D** → **F** step competing to the S_N_2 pathway. In the case of the alkyl substituent both S_N_1 and S_N_2 pathways are unlikely, since the barrier of the S_N_2 pathway is too high (21.8 kcal mol^–1^) and the S_N_1 mechanism is forbidden (the necessary **E-open** intermediate is not formed).

Intrigued by the plausible intermediacy of a cyclic cationic structure such as **E** as inferred from both experimental and theoretical evidence, we set out to experimentally probe its intermediacy in these redox-neutral arylation reactions. In particular, we were aware that, if formed, **E** would necessarily coexist with an equivalent of the Friedel–Crafts-competent nucleophile diphenyl sulfide.

**Scheme 3:**
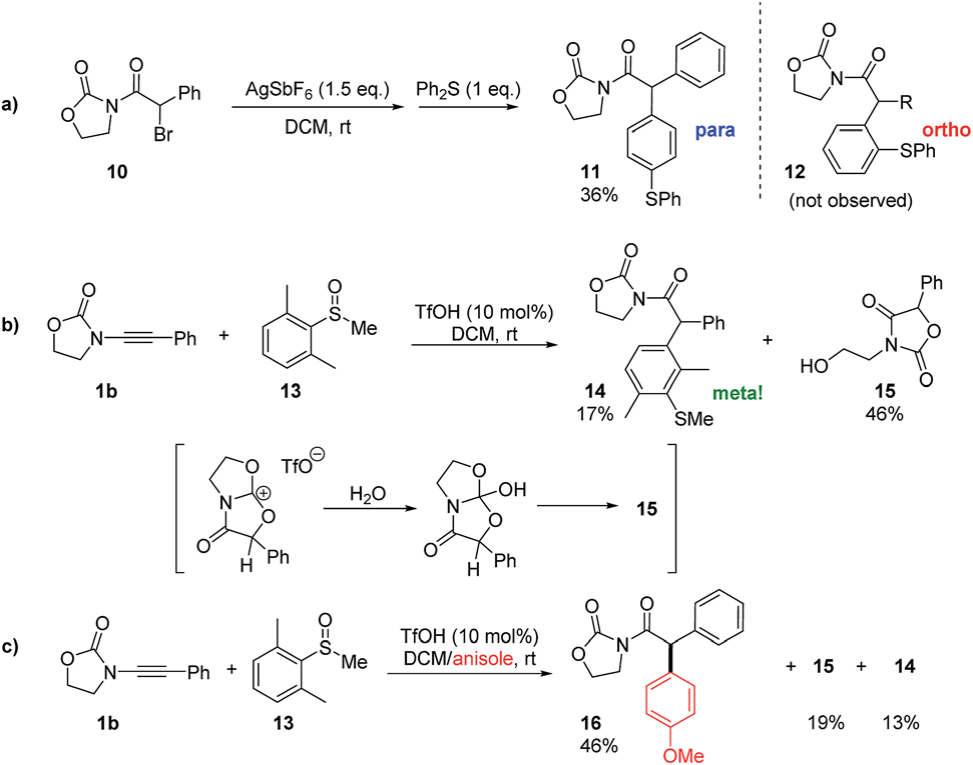
Mechanistic experiments.

As shown in Scheme 3a, treatment of the readily available α-bromoimide **10** with 1.5 equivalents of AgSbF_6_ in the presence of an excess of diphenylsulfide indeed led to a C–C coupled product of the same molecular weight as **12**. Detailed NMR analysis and X-ray single crystal analysis, however, revealed this to be the *para*-substituted product and not the *ortho*-adduct that was exclusively obtained in the previously described catalytic transformation.^10^ Thus, while electrophilic aromatic substitution of the cationic intermediate **E** is possible, its outcome is predictably governed by sterics.

The absence of *para*-products akin to **11** in any of the redox arylation transformations previously reported by Maulide and co-workers suggests that this is not an operative pathway in the originally reported catalytic reaction.^10^

Nonetheless, this result intriguingly suggested a catalytic experiment in which a sulfoxide **13**, where both *ortho*-positions are blocked, is employed as a nucleophile (Scheme 3b). Two products were observed: the alcohol **15**, which appears to be the product of hydrolysis of **E** (Scheme 3b), and the unanticipated *meta*-substituted, α-arylated product **14** in a low yield.^17^ The result of this experiment suggests that **E** might indeed be an energy minimum in cases where evolution to product **F** is blocked, and we hypothesised that it might be possible to intercept **E** if a more powerful aromatic nucleophile were present. We were delighted to see that the repetition of the experiment shown in Scheme 3b, in this instance in the presence of a large excess of anisole, led to a respectable 46% yield of the intermolecular C–C bond captured product **16** along with 19% of the hydrolysis product **15** and 13% of the *meta*-arylated compound **14** (Scheme 3c).

**Scheme 4:**
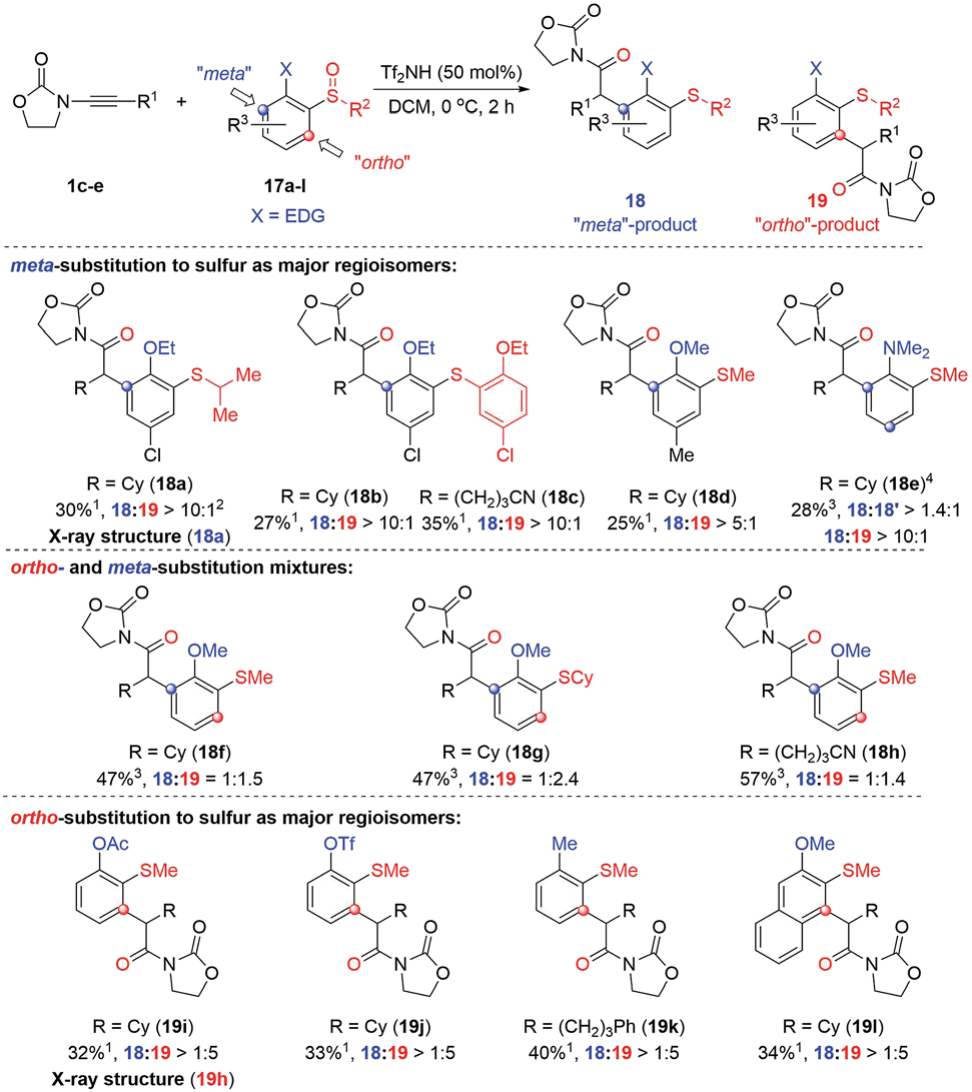
Acid-catalysed reaction of ynamides with (*ortho*-donor-arylsulfoxides). ^1^Isolated yields of the product; ^2^Ratio of product **18** : **19** determined from by crude ^1^H NMR analysis; ^3^Overall isolated yield of **18** + **19**. ^4^120 mol% of Tf_2_NH was used.

The observation of *meta-*substituted products on the rearrangement of *ortho*-substituted sulfoxides is an unusual result.^18^ We hypothesised that strongly electron-donating moieties located *ortho* to the sulfoxide might be able to render this an even more efficient process and thus we decided to probe the reaction with (2-alkoxy)aryl sulfoxides. The results of our ensuing investigation are compiled in Scheme 4 (for the optimisation of reaction conditions see the ESI†).

**Fig. 10:**
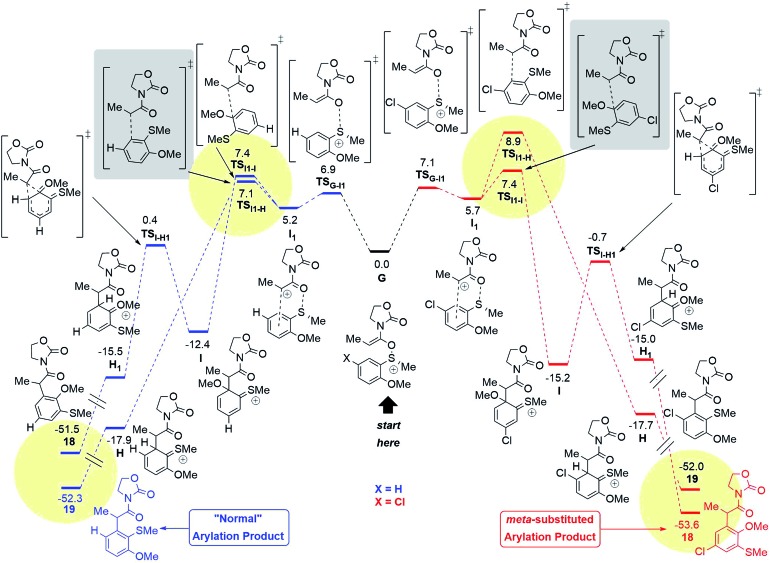
Computed reaction profile (SCS-RI-MP2-COSMO/def2-QZVP//RI-MP2/def2-TZVP,^24^ Δ*G*_298,DCM_) for the conversion of the intermediate **G** into both possible products for the X: H (blue) and the X: Cl (red) systems. The summarised energies of the intermediate **G** and the counterion NTf_2_^–^ are taken as a reference (0.0 kcal mol^–1^). The yellow circles highlight the kinetic and thermodynamic factors, responsible for the regioselectivity. The grey boxes highlight the key transition states responsible for the proposed pathways.

As can be seen, two regioisomeric products compete: the unexpected “*meta*”-arylated product **18** and the “*ortho*”-arylated compound **19**. Even though yields are modest,^19^ high regioselectivities were observed in many cases which led us to divide the substrates into three subgroups dependent on the ratio in which the respective isomers were formed. As shown, strongly electron-donating groups, such as dimethyl amino, ethoxy- or methoxy-groups, in combination with an additional substituent, clearly favour the formation of the “*meta*” products **18** (*cf.***18a–18e**; the structure of **18a** was confirmed by X-ray analysis, see ESI† for details).^20^ However, removing the sterically biasing substituent (**18f–18h***cf.*Fig. 10 and discussion thereafter to rationalise the effect of the –Cl substituent) or changing to less powerful electron-donating groups such as –OAc or –OTf or even –Me (**18f–18h***vs.***19g–19i**) led to increased formation of the “*ortho*” arylated products **19i–k**.^21^ Counterintuitively, in case of the naphthalene derivative **19l** the “*ortho*”-isomer was the major product suggesting the fused aromatic system to favour an *ortho* arylation in this case.^22^

**Scheme 5:**
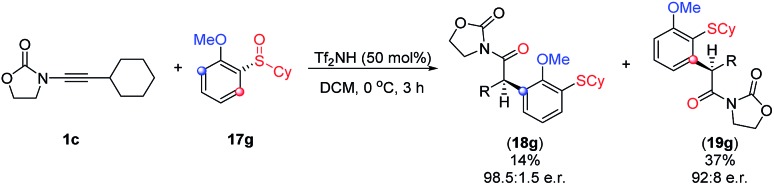
Asymmetric rearrangement with chiral enantiopure sulfoxides.

Unsure whether the *meta*-substituted products **18** arise by a direct process akin to a sigmatropic rearrangement, we carried out the experiment shown in Scheme 5. As depicted, the use of the commercially available, chiral enantioenriched sulfoxide **17g** in conjunction with ynamide **1c** led to both rearranged products **18g** and **19g** in good to excellent levels of chirality transfer.^23^ This suggests a high degree of concertedness, that is not readily compatible with a Friedel Crafts-type mechanism.

Quantum chemical calculations have been performed in order to clarify the observed regioselectivity. The reaction profile in Fig. 10 represents both possible pathways for the formation of products with the general structure **18** and **19** for two different systems: the aromatic ring with a chloro-substituent in the *para*-position (X = Cl, shown in red) and the unsubstituted system (X = H, shown in blue).

The initial N,O-ketene acetal intermediate **G** (Fig. 10, middle) can undergo two types of reactions (*vide supra*): (1) the classical concerted rearrangement and (2) the fragmentation. In the case of the concerted mechanism, the intermediate **G** must be a divergence point for the reaction to form one of the products **18** or **19**. However, the DFT results and moreover the calculations at the higher level of theory (*ab initio*) RI-MP2/def2-TZVP support the stepwise mechanism,^24^ where the S–O bond of the intermediate **G** breaks *via***TS_G–I1_** leading to the intermediate **I_1_**. The formation of the intermediate **I_1_** is energetically similar for both systems (X = H or X = Cl) as shown in Fig. 10.

After the intermediate **I_1_**, two possibilities exist. One of them proceeds *via***TS_I1–H_** and forms the intermediate **H**, which rearomatises to the “normal” product with general structure **19.** Alternatively, the reaction can proceed *via* the **TS_I1–I_** leading to the intermediate **I**, where a C–C bond is formed to the methoxy-substituted atom of the aromatic system. Intermediate **I** can undergo a 1,2-shift^18^ to form the intermediate **H_1_**, which finally rearomatises analogously to the intermediate **H** to form the *meta*-product with the general structure **18**.

In the case of X = H (blue), the pathway to form **19** is preferable compared to the formation of **18** (see **18d–f**Scheme 4), while the situation is changed for X = Cl with the “abnormal” product **18** as a main product (see **18a–c**Scheme 4). This switch of regioselectivity is in good agreement with the experimental results. The calculations show that the observed regioselectivity is controlled by both kinetic and thermodynamic factors. Indeed, for the case of X = H, the barrier associated with the **TS_I1–I_** is 0.3 kcal mol^–1^ larger as compared to that of **TS_I1–H_**, while for X = Cl, the **I_1_–I** barrier is *vice versa* 1.5 kcal mol^–1^ smaller than the **I_1_–H** barrier. Thermodynamically, the “abnormal” pathway (formation of the product **18**) is also less favourable than the “normal” rearrangement for X = H: Δ*G*(**G** → **18**) = –51.5 kcal mol^–1^, while Δ*G*(**G** → **19**) = –52.3 kcal mol^–1^. However, for the chloro-substituent, the formation of the *meta*-product **18** becomes more probable: Δ*G*(**G**(Cl) → **18**(Cl)) = –53.6 kcal mol^–1^*vs.* Δ*G*(**G**(Cl) → **19**(Cl)) = –52.0 kcal mol^–1^. In any event, it is remarkable that a chiral sulfoxide is able to mediate efficient chirality transfer from sulfur to carbon over a sequence of transformations, as depicted in Fig. 10 and Scheme 5.

## Conclusions

In summary, we have presented a combined ESI-MS/quantum chemical mechanistic analysis of the redox-neutral arylation of ynamides and sulfoxides. Several noteworthy observations result from this study. First, ESI(+)-MS suggested the unusual presence of α-carbonyl cations whenever aryl substitution is present. Second, these were substantiated by computations, which outline the existence of a fine borderline between sigmatropy and fragmentation that can be crossed for particularly stabilised systems. Third, key mechanistic experiments showcased in practice how particularly “difficult” (or “sluggish”) rearrangements may easily cross the aforementioned borderline between sigmatropy and fragmentation. Fourth, additional work shows how electronically biased systems can provide *meta*-arylated products by a combination of unusual [3,3]- and [1,2]-shifts. Together, these observations reveal a rich panorama of mechanistic pathways available to an otherwise apparently “innocent”, classical Claisen-type [3,3]-sigmatropic rearrangement.

## Conflicts of interest

There are no conflicts to declare.

## Supplementary Material

Supplementary information

Crystal structure data
