# Supplementary material for: Unusual mechanisms in Claisen rearrangements: an ionic fragmentation leading to a meta-selective rearrangement
Source: Chem Sci. 2018 Apr 10;9(17):4124–31. doi: 10.1039/c7sc04736c (PMC5941282; doi:10.1039/c7sc04736c)
Supplement: Supplementary file 1 [file SC-009-C7SC04736C-s001.pdf]

Electronic Supplementary Material (ESI) for Chemical Science.
This journal is © The Royal Society of Chemistry 2018

Unusual Mechanisms in Claisen Rearrangements: an Ionic Fragmentation leading to a *meta*-Selective Rearrangement

Boris Maryasin,[a,b] * Dainis Kaldre,[a,†] Renan Galaverna,[c,†] Immo Klose,[a] Stefan Ruider,[a] Martina Drescher,[a] Hanspeter Kählig,[a] Leticia González,[b] Marcos N. Eberlin,[c] Igor D. Jurberg[d,]* and Nuno Maulide[a,]*

[a] University of Vienna, Faculty of Chemistry, Institute of Organic Chemistry, Währinger Strasse 38, 1090 Vienna, Austria; [b] University of Vienna, Faculty of Chemistry, Institute of Theoretical Chemistry, Währinger Strasse 17, 1090 Vienna, Austria; [c] State University of Campinas, ThoMSon Mass Spectrometry Laboratory, Institute of Chemistry, Rua Monteiro Lobato 270, 13083-970, Campinas, Brazil [d] State University of Campinas, Institute of Chemistry, Rua Monteiro Lobato 270, 13083-970, Campinas, Brazil.

Supporting Information

Table of contents

1.Materials and Methods: 3

2.Mass spectrometry data: 3

3.ESI(+)-MS/MS and accurate mass measurements for the main species detected in the monitoring of the Claisen rearrangement of ynamides and sulfoxides/alcohols. 4

4.General procedures and characterisation of compounds 22

5.NMR Spectra 40

6.HPLC spectra 77

7.X-ray crystallographic data for the **18a**, **19g** and **10** 80

8.Computational details 89

8.1.General approaches 89

8.2.Computations to clarify the *meta*-substitution effect 89

8.3.Thermochemical data 92

8.4.Cartesian coordinates 158

9.References 209

1.Materials and Methods:

All reactions were carried out under air, in oven dried glassware with magnetic stirring, unless otherwise noted. Liquids and solutions were transferred via syringe. All reagents were purchased from commercial suppliers and used without further purification. All dry solvents employed in the reactions were distilled from appropriate drying agents prior to use or Acros Extra dry solvents with AcroSeal were used (Experiments for scheme 6 and 7).

Organic solutions were concentrated under reduced pressure on a IKA rotary evaporator RV-10 Control. Reactions were monitored by thin-layer chromatography (TLC) on Silica gel 60 F_{254} aluminium plates (Merck). Chromatograms were visualized by fluorescence quenching with UV light at 254 nm or by staining using potassium permanganate or para-anisaldehyde solutions. Flash column chromatography was performed using Merck silica gel 60 (particle size 35-70μm) using reagent grade solvents. ^{1}H and ^{13}C NMR spectra were recorded on either Bruker DPX-250, AV-400, AV-500 or AV-600 MHz spectrometers. Chemical shifts (δ) are given in parts per million, referenced to the residual peak of $CDCl_3$, δ = 7.26 (^{1}H NMR) and δ = 77.0 (^{13}C NMR) as internal references. Coupling constants (J) are reported in Hertz (Hz). The following abbreviations were used to designate chemical shift multiplicities: s = singlet, d = doublet, t = triplet, q = quartet, quint. = quintuplet, sext. = sextuplet, sept. = septuplet, m = multiplet. High-resolution mass spectra were recorded on a Q-Exactive Hybrid Quadrupole-Orbitrap Mass Spectrometer (Thermo Scientific, Bremen, Germany) and a 6500 Series Accurate-Mass Quadrupole Time-of-Flight (Q-TOF) LC/MS (Agilent technologies, California, USA) or using a Finnigan MAT 8200 or (70 eV) or an Agilent 5973 (70 eV) spectrometer, using electrospray ionization (ESI). Optical rotations were measured on a Perkin Elmer 341 polarimeter using a 100 mm path-length cell at 589 nm (c given in g/100 mL). Chiral HPLC was performed using AGILENT Infinity 1260 with Chiralpak IC column. Details of chromatographic conditions are indicated under each compound.

2.Mass spectrometry data:

Ultrahigh resolution MS and MS/MS data was obtained using two different mass spectrometers.

1) Q-Exactive Hybrid Quadrupole-Orbitrap Mass Spectrometer (Thermo Scientific, Bremen, Germany) equipped with a electrospray ionization source operating in the positive mode ESI(+)-MS in the following conditions: VCap 3.9 kV; tube transfer temperature 280°C; nebulizer gas at 30 psi; S-lens 100 V and collision energy for the MS/MS experiments were varied in a range of 30-50 V using N_2. The diluted samples were injected via a syringe pump at a flow rate of 5 $\mu L.min^{-1}$. Data was recorded in full MS mode in ESI(+) from *m/z* 50 to 1000. The average resolving power (Rp) was 140000 at *m/z* 200, where Rp was calculated as $M/\Delta M_{50\%}$, that is, the *m/z* value divided by the

peak width at 50% peak height. Mass spectra were the result of over 3 microscans and processed via the Xcalibur 3.0.63 software (Thermo Scientific, Bremen, Germany).

2) Agilent ifunnel Q-TOF 6550 LC–MS with source Dual Agilent Jet Stream ESI (Dual AJS-ESI) in the following conditions: drying gas temperature at 280 ºC; drying gas flow 12 $L.min^{-1}$; nebulizer gas at 30 psi; sheath gas temperature at 300 °C; sheath gas flow 12 L min^{-1}; VCap 2500 V; nozzle voltage 0 V; fragmentor 150 V; OCT 1 RF Vpp 750 V and collision energy for the MS/MS experiments were varied in a range of 20-30 V using N_2. The diluted samples were injected via LC in FIA mode at a flow rate of 100 $\mu L.min^{-1}$. Data were recorded in full MS mode in ESI(+) from *m/z* 50 to 1000. Mass spectra were processed via the Mass Hunter B.06.00 software (Agilent technologies, California, USA).

The values of *m/z* reported and their errors below 5 ppm correspond to values found in Full ESI(+)-MS analyses. Values of *m/z* reported for ESI(+)-MS/MS experiments have typically a lower resolution and are not necessarily below the range of 5 ppm. The values of *m/z* reported in the SI and in the manuscript are truncated in the second decimal value only for simplification, but they were measured with very high accuracy.

3.ESI(+)-MS/MS and accurate mass measurements for the main species detected in the monitoring of the Claisen rearrangement of ynamides and sulfoxides/alcohols.

General procedure of analysis: All reactions were prepared following the *general procedure C (see section 4 of this SI)*, in a 0.1 mmol-scale in relation to the ynamide. Aliquots (1 µL) were taken from the reaction mixture, diluted in 1 mL of acetonitrile and injected in the the electrospray ionization source operating in positive ion mode ESI(+)-MS for reaction monitoring.

Mass Spectra derived from the monitoring of model reactions, which were not incorporated in the main text of the manuscript, are provided below.

The redox-neutral oxoarylation protocol employing ynamides **1a/1b** and diphenyl sulfoxide **2a**, catalyzed by either $PhCO_2H$, *p*-TsOH or HOP(O)$(OPh)_2$ (10 mol%), do not lead to the corresponding arylated amide **7a/ 7b**, respectively, to any significant extent. Whereas $PhCO_2H$ seems not to be a strong enough acid to catalyze the reaction, pTsOH and $(PhO)_2$P(O)OH lead to enolate derivatives **3a'**/ **3a''**, and **3b´**/ **3b´´** respectively, but they do not evolve further to the corresponding **7a**/ **7b**. These intermediates rather appear to constitute "dead ends" mechanistically speaking (Scheme S1).

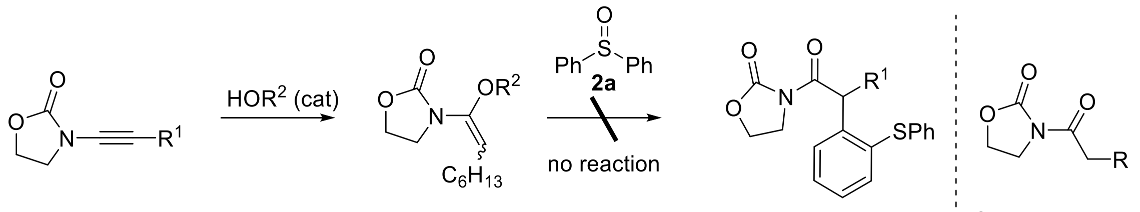

1a, R1 = C_6H_{13}
1b, R1 = Ph

3a´, R1 = C_6H_{13}, R2 = *p*-Ts
3a´´, R1 = C_6H_{13}, R2 = P(O)(OPh)2
3b´, R1 = Ph, R2 = *p*-Ts
3b´´, R1 = Ph, R2 = P(O)(OPh)2

7a, R1 = C_6H_{13}
7b, R1 = Ph

Scheme S1. Oxoarylation of ynamides **1a** and **1b** employing $PhCO_2H$, p-TsOH or $(PhO)_2P(O)OH$ as catalysts.

Analysis of the reaction of ynamides **1a** and **1b** with common acids**:**

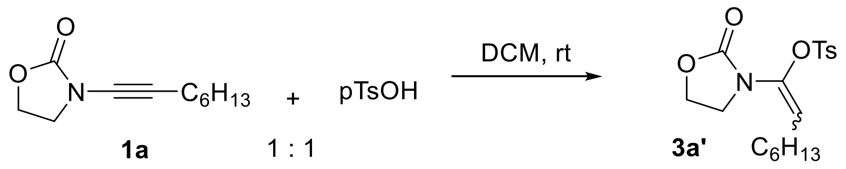

Scheme S2. Stoichiometric reaction of ynamide **1a** with pTsOH.

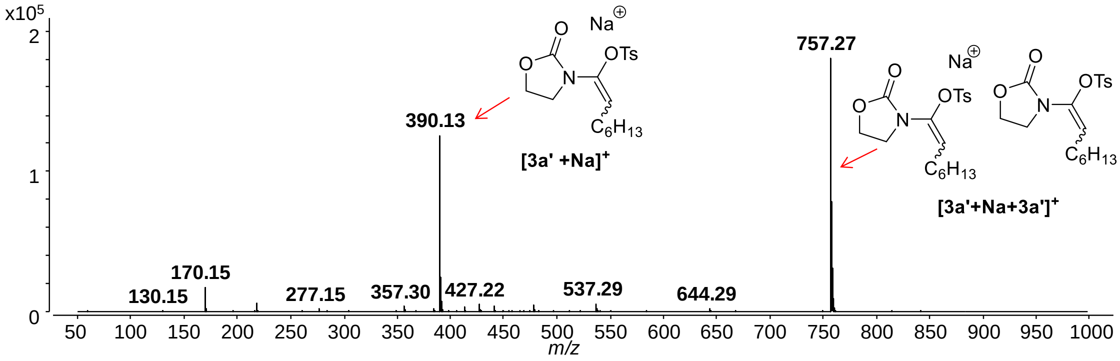

Figure S1. ESI(+)-MS of stoichiometric reaction of ynamide **1a** with pTsOH.

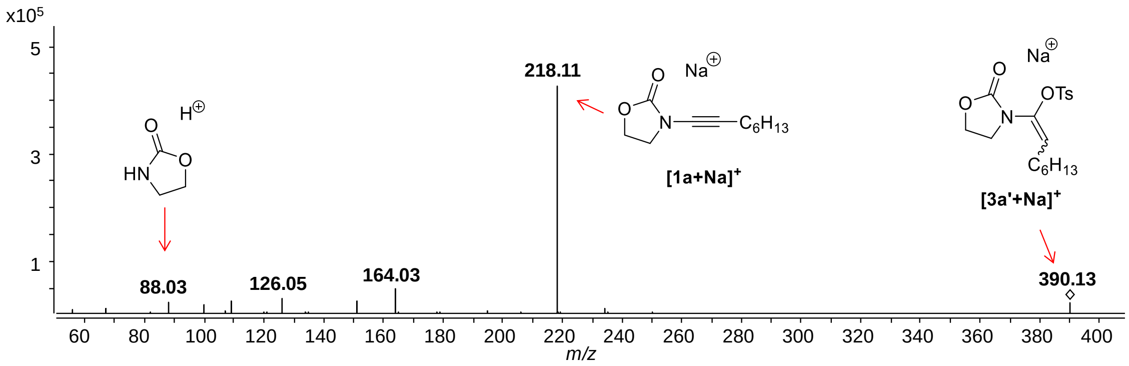

Figure S2. ESI(+)-MS/MS of [**3a'**+Na]$^+$: $C_{18}H_{25}NO_5NaS$, calculated: 390.13456, found: 390.13310; 3.74 ppm error.

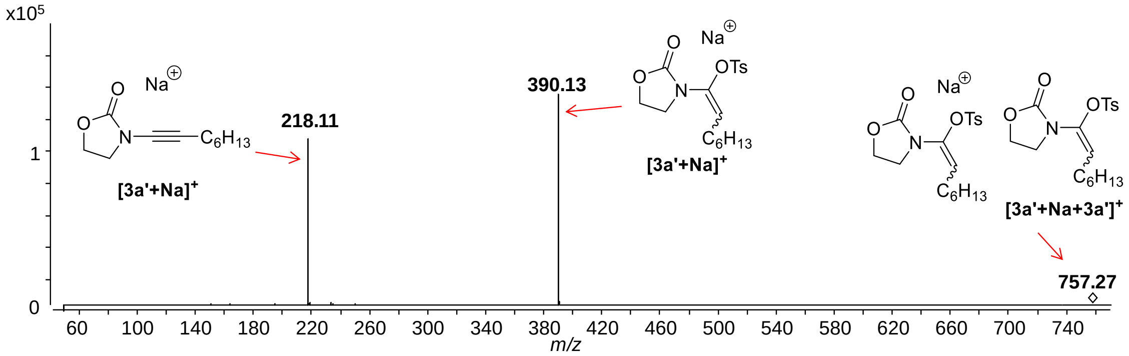

Figure S3. ESI(+)-MS/MS of [**3a'**+Na+**3a'**]$^+$: $C_{36}H_{50}N_2O_{10}NaS_2$, calculated: 757.27991, found: 757.27698; 3.87 ppm error.

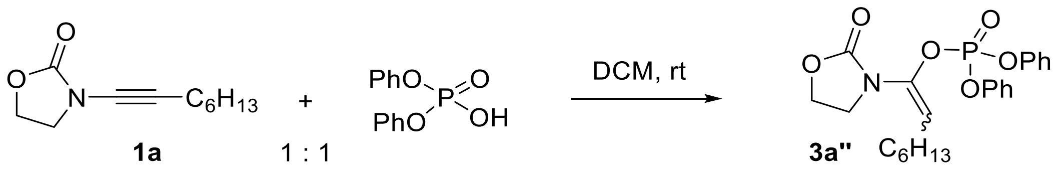

Scheme S3. Stoichiometric reaction of ynamide **1a** with $(PhO)_2P(O)OH$.

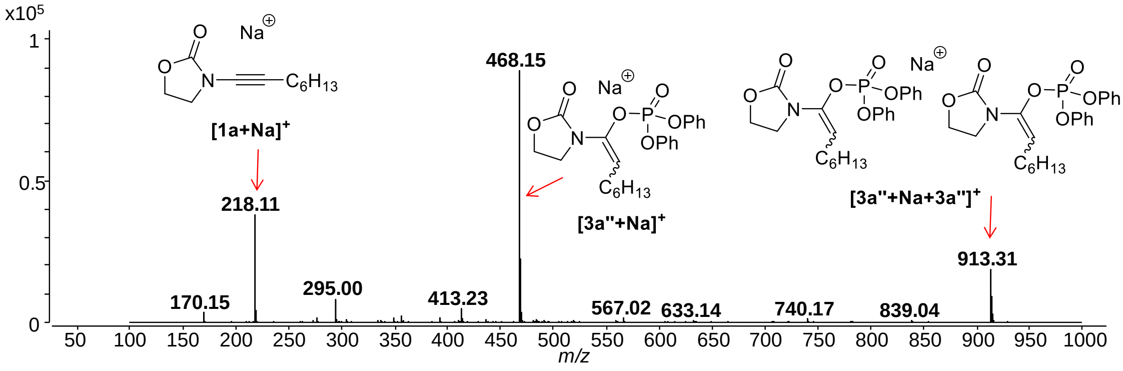

Figure S4. ESI(+)-MS of stoichiometric reaction of ynamide **1a** with $(PhO)_2P(O)OH$.

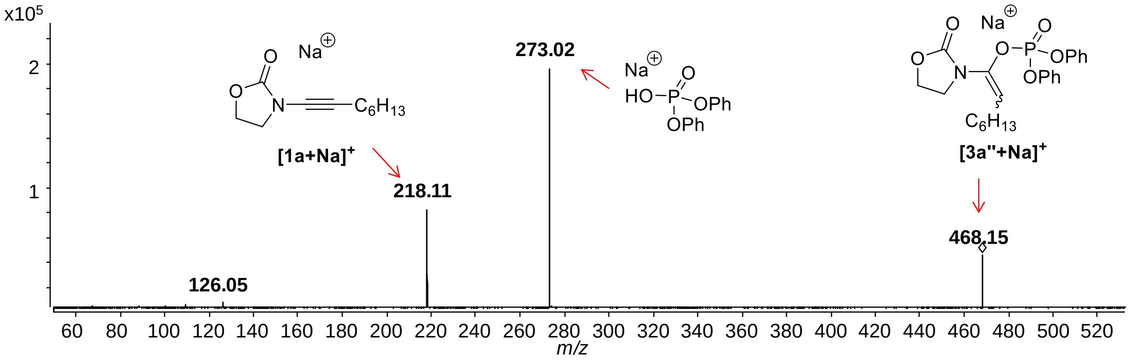

Figure S5. ESI(+)-MS/MS of [**3a**''+Na]$^+$: $C_{23}H_{28}NO_6NaP$, calculated: 468.15465, found: 468.15382; 1.77 ppm error.

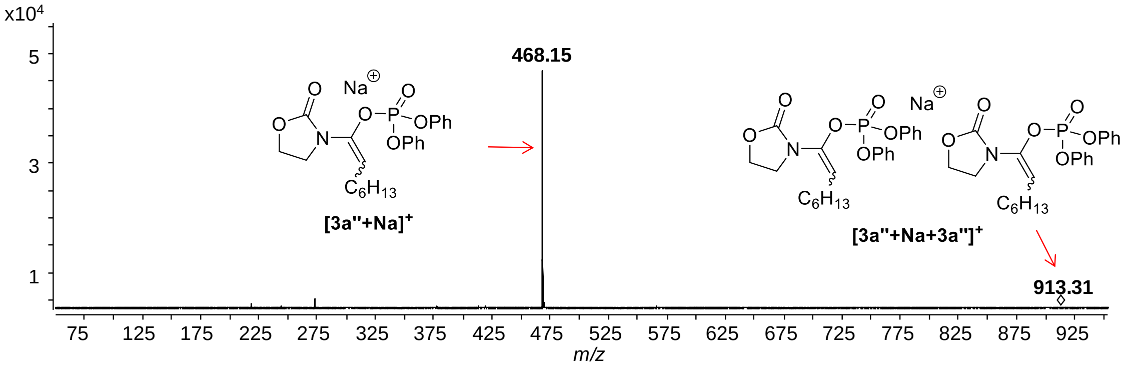

Figure S6. ESI(+)-MS/MS of [**3a**''+Na+**3a**'']$^+$: $C_{46}H_{56}N_2O_{12}NaP_2$, calculated: 913.32007, found: 913.31807; 2.19 ppm error.

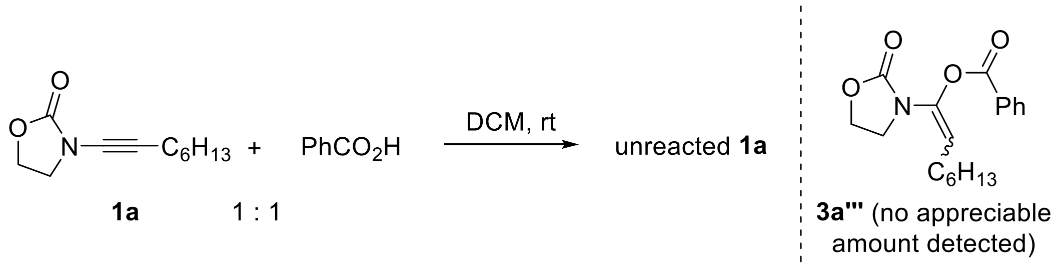

Scheme S4. Stoichiometric reaction of ynamide **1a** with $PhCO_2H$.

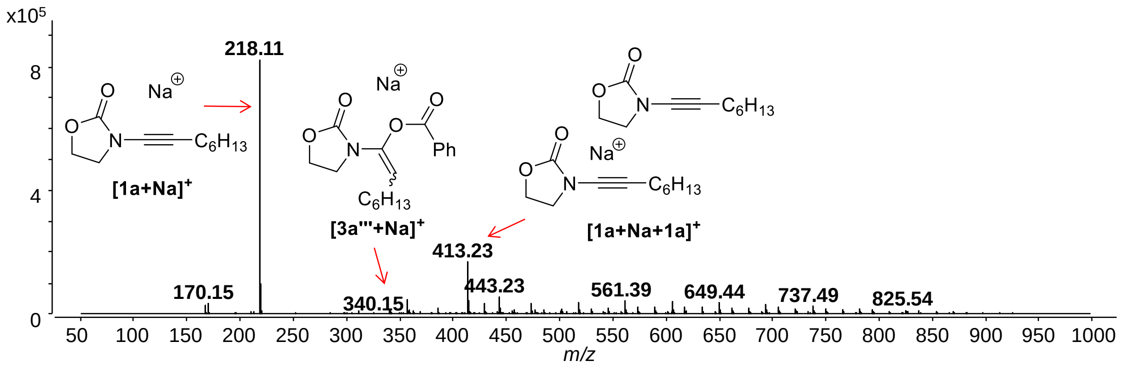

Figure S7. ESI(+)-MS of stoichiometric reaction of ynamide **1a** with $PhCO_2H$.

It was not possible to acquire the ESI(+)-MS/MS spectrum of *m/z* 340 due to the low abundance of this signal.

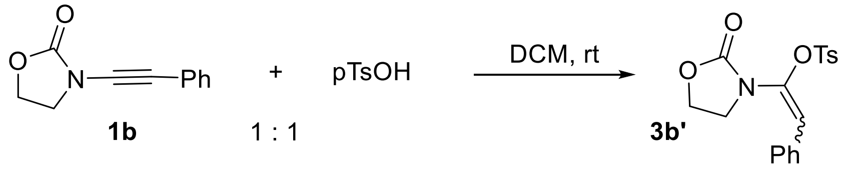

Scheme S5. Stoichiometric reaction of ynamide **1b** with pTsOH.

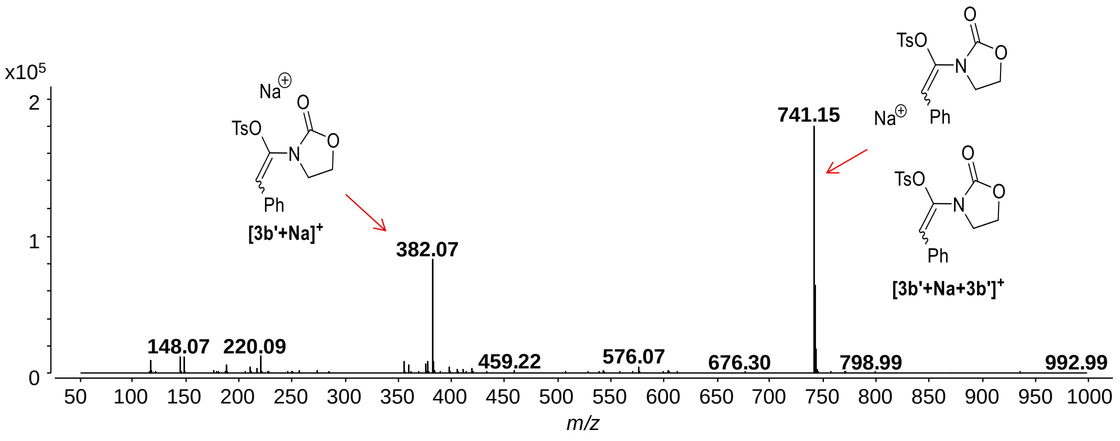

Figure S8. ESI(+)-MS of stoichiometric reaction of ynamide **1b** with pTsOH.

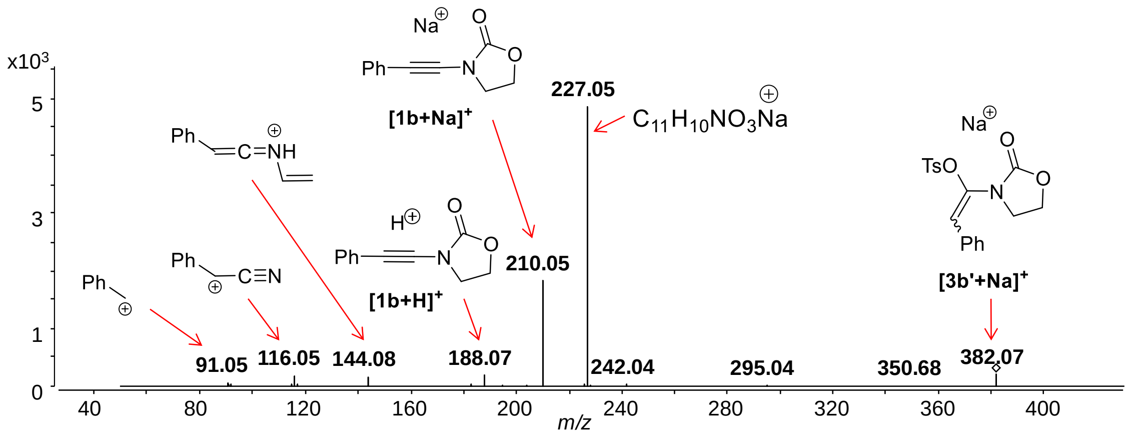

Figure S9. ESI(+)-MS/MS of [**3b**'+Na]$^+$: $C_{18}H_{17}NO_5SNa$, calculated: 382.07196, found: 382.07215; 0.50 ppm error.

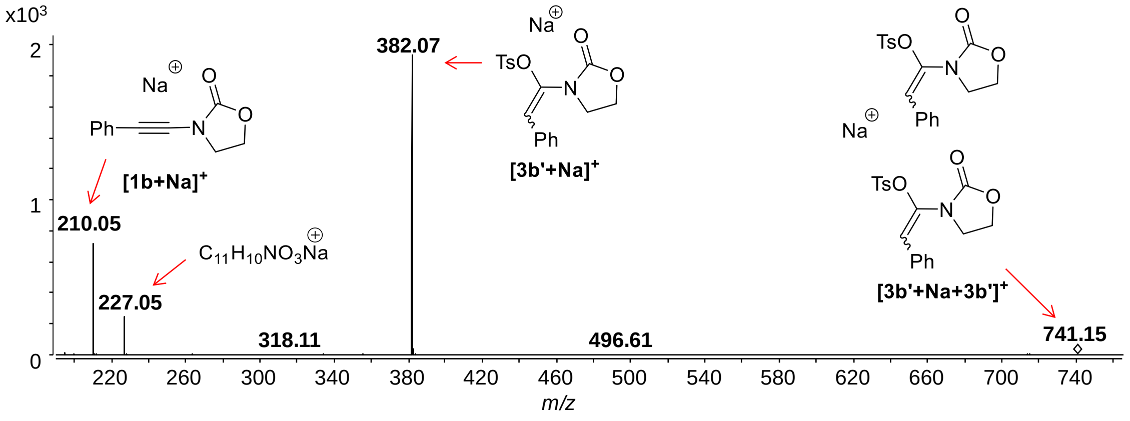

Figure S10. ESI(+)-MS/MS of [**3b**'+Na+**3b**']$^+$: $C_{36}H_{34}N_2O_{10}S_2Na$, calculated: 741.15471, found: 741.15489, 0.24 ppm error.

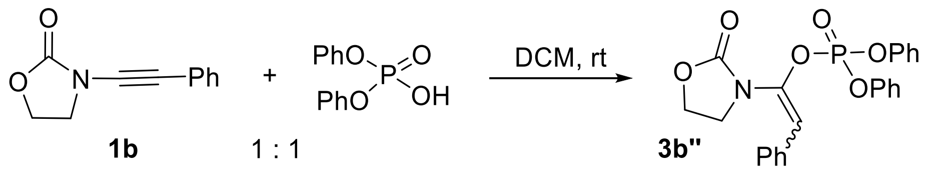

Scheme S6. Stoichiometric reaction of ynamide **1b** with $(PhO)_2P(O)OH$.

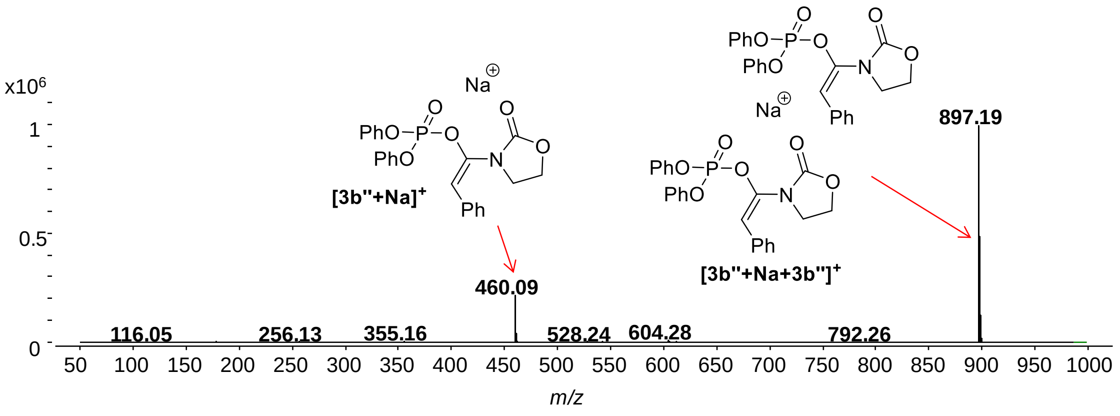

Figure S11. ESI(+)-MS of stoichiometric reaction of ynamide **1b** with $(PhO)_2P(O)OH$.

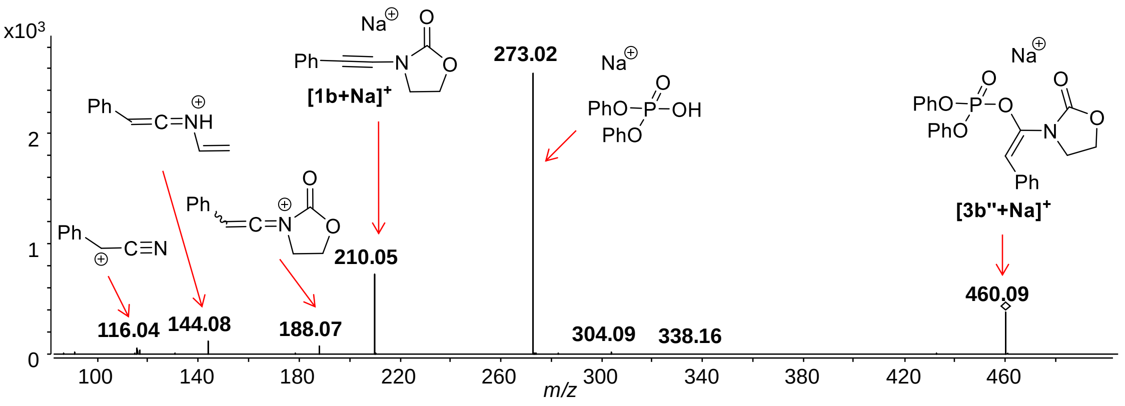

Figure S12. ESI(+)-MS/MS of [**3b''**+Na]$^+$: $C_{23}H_{20}NO_6PNa$, calculated: 460.09204, found: 460.09199; 0.11 ppm error.

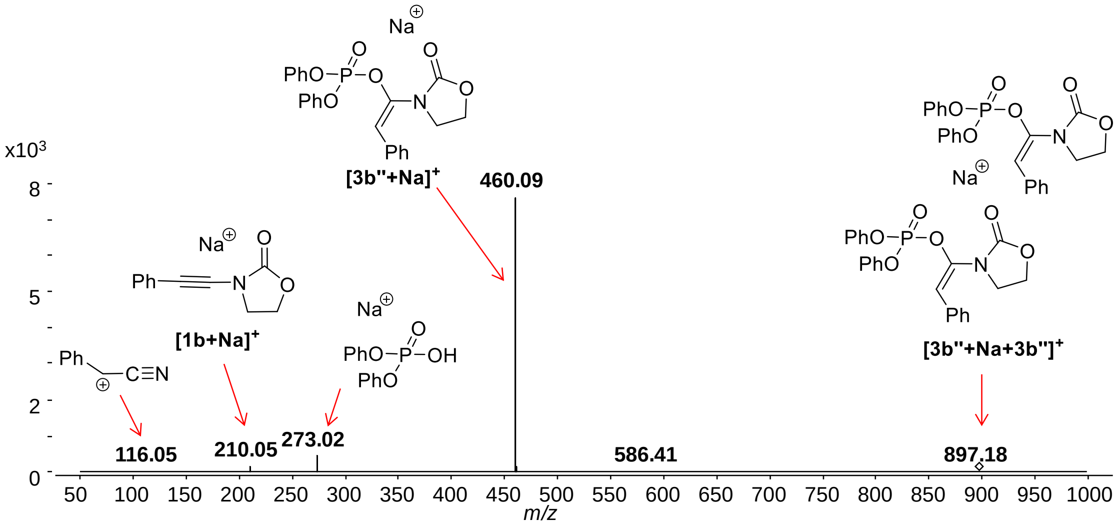

Figure S13. ESI(+)-MS/MS of [**3b**''+Na+**3b**'']$^{+}$: $C_{46}H_{40}N_2O_{12}P_2Na$ calculated: 897.19487, found: 897.19234; 2.82 ppm error.

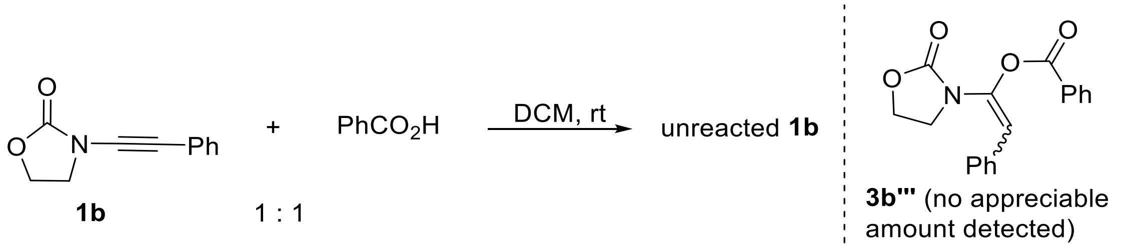

Scheme S7. Stoichiometric reaction of ynamide **1b** with $PhCO_2H$.

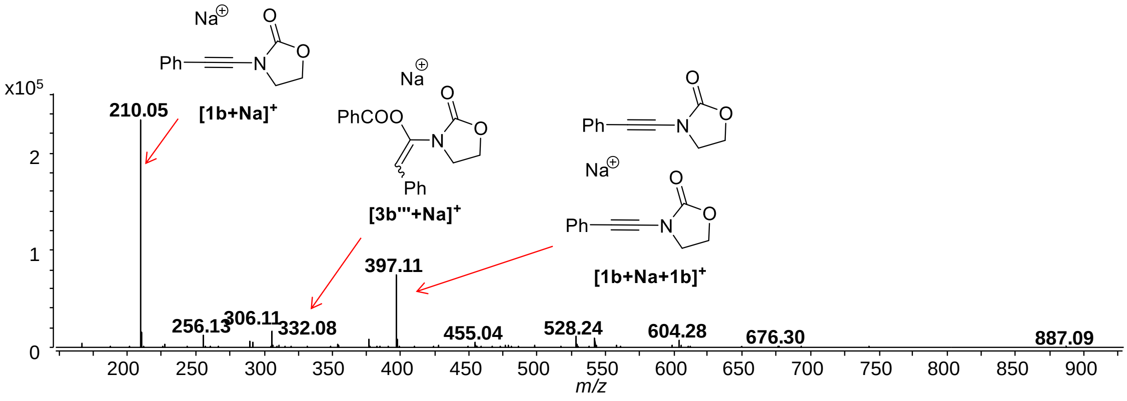

Figure S14. ESI(+)-MS of stoichiometric reaction of ynamide **1b** with $PhCO_2H$.

It was not possible to acquire the ESI(+)-MS/MS spectrum of *m/z* 332 due to the low abundance of this signal.

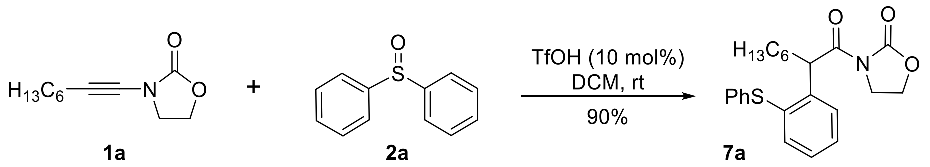

Scheme S8. Reaction of ynamide **1a** with diphenyl sulfoxide **2a**.

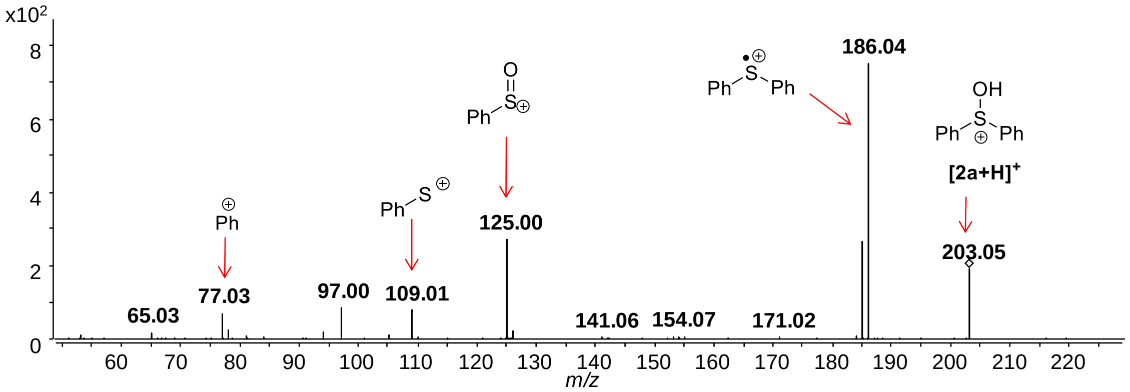

Figure S15. ESI(+)-MS/MS of [**2a**+H]$^{+}$: $C_{12}H_{11}OS$, calculated: 203.05251, found 203.05252; 0.04 ppm error.

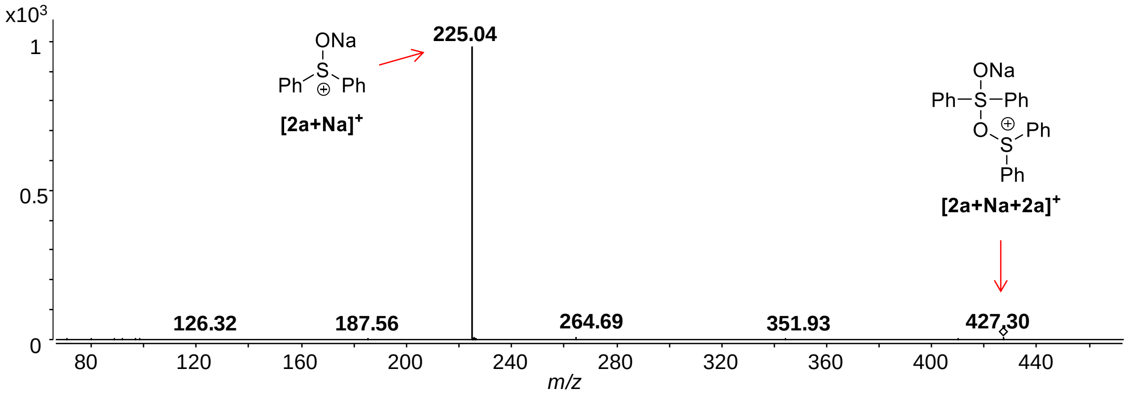

Figure S16. ESI(+)-MS/MS of [**2a**+Na+**2a**]$^{+}$: $C_{24}H_{20}O_2S_2Na$, calculated: 427.07969, found: 427.07966; 0.08 ppm error.

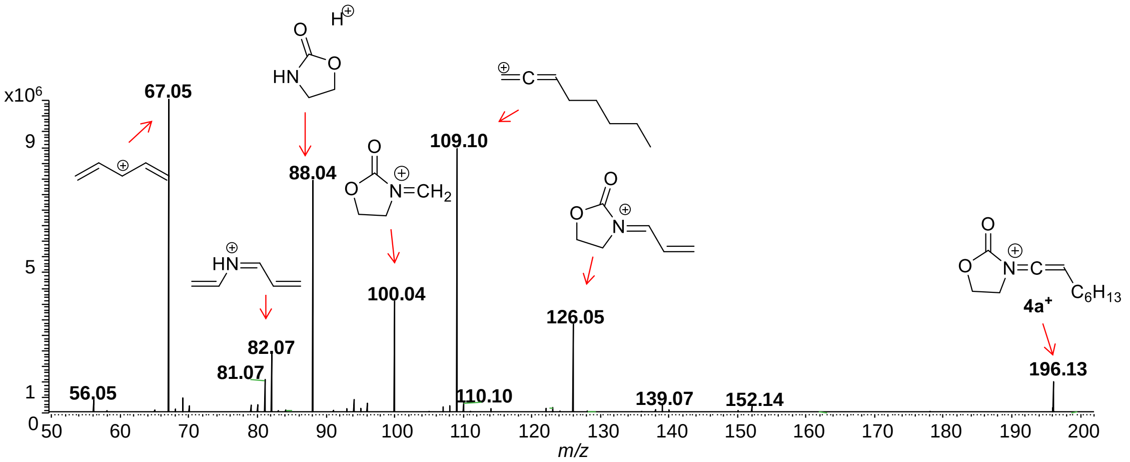

Figure S17. ESI(+)-MS/MS of **4a**$^+$: $C_{11}H_{18}NO_2$, calculated: 196.13321, found: 196.13297; 1.2 ppm error.

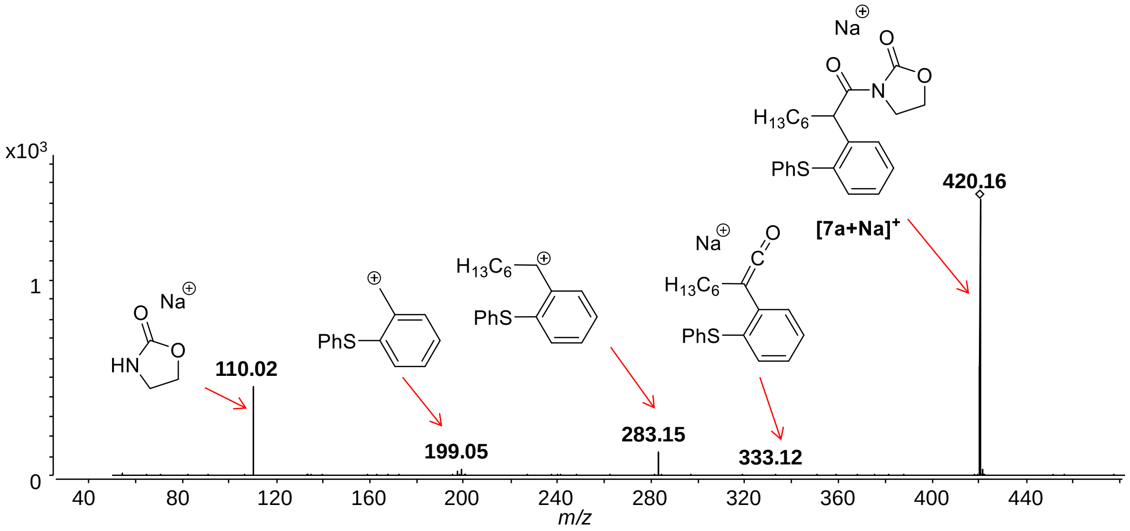

Figure S18. ESI(+)-MS/MS of [**7a**+Na]$^+$: $C_{23}H_{27}NO_3SNa$, calculated: 420.16039, found: 420.16081; 1.0 ppm error.

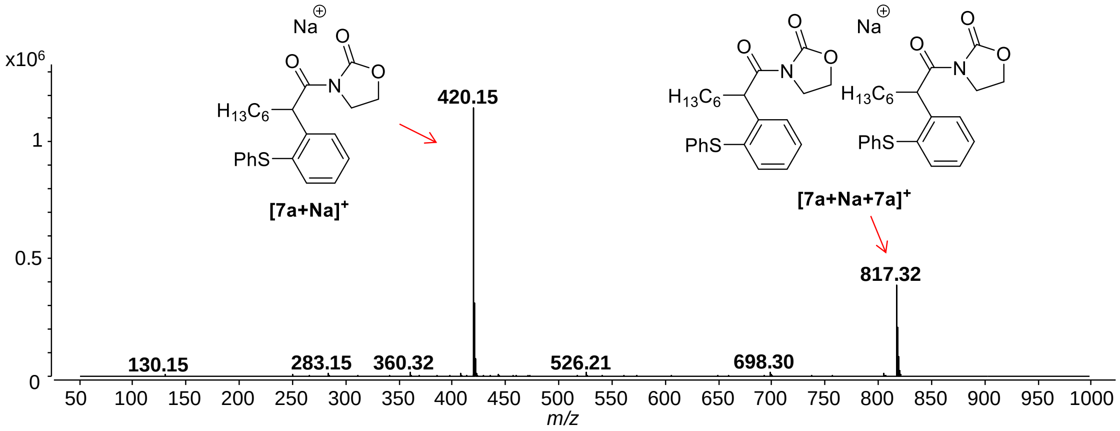

Figure S19. ESI(+)-MS/MS of [**7a**+Na+**7a**]$^+$: $C_{46}H_{54}N_2O_6S_2Na$, calculated: 817.33155, found: 817.33141; 0.17 ppm error.

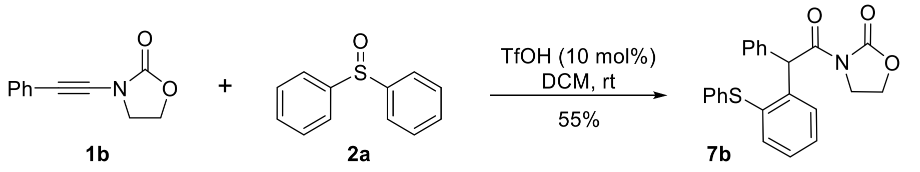

Scheme S9. Reaction of ynamide **1b** with diphenyl sulfoxide **2a**.

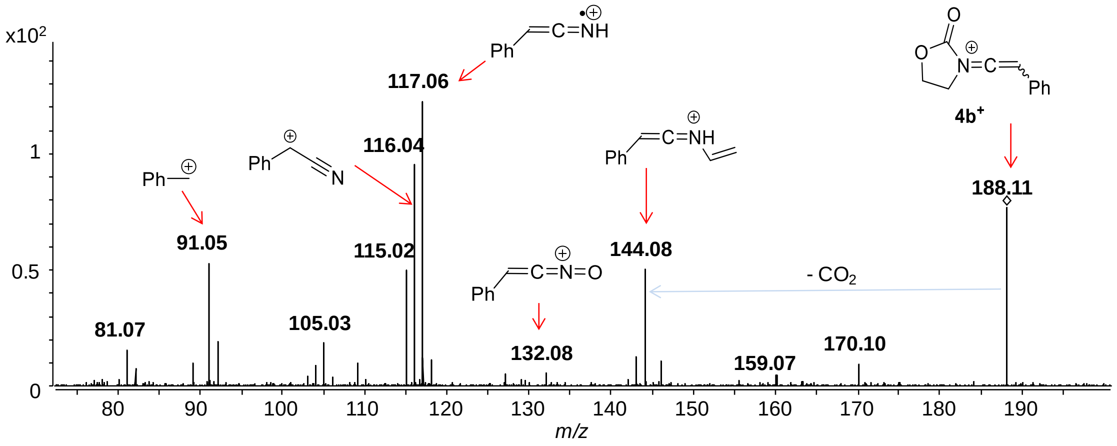

Figure S20. ESI(+)-MS/MS of **4b**$^+$: $C_{11}H_{10}NO_2$, calculated: 188.07060, found: 188.07010; 2.7 ppm error

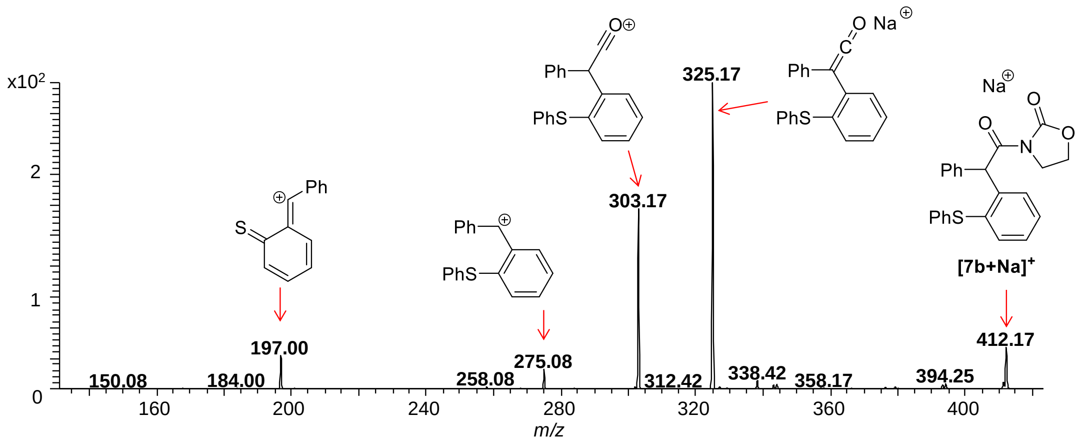

Figure S21. ESI(+)-MS/MS of [**7b**+Na]$^+$: $C_{23}H_{19}NO_3SNa$, calculated: 412.09779, found: 412.09770; 0.2 ppm error.

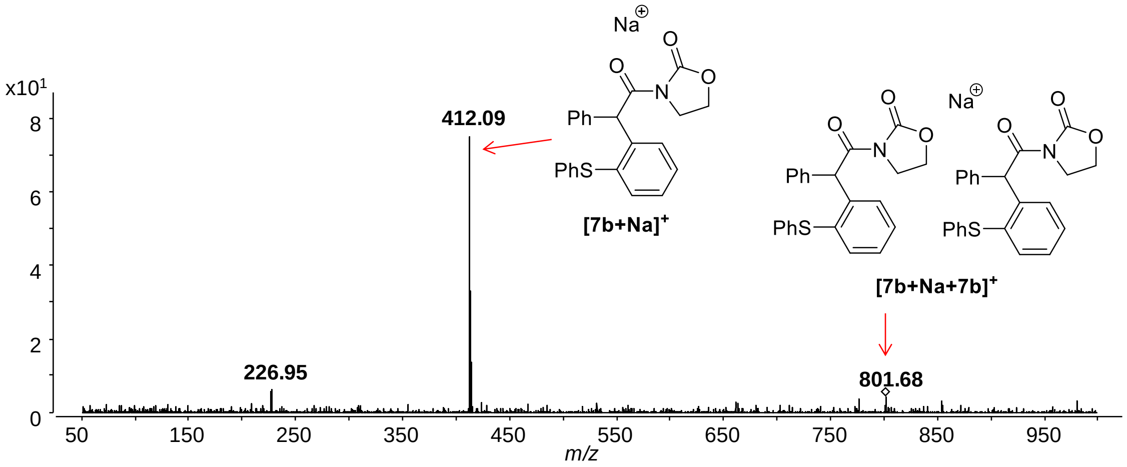

Figure S22. ESI(+)-MS/MS of [**7b**+Na+**7b**]$^+$: $C_{46}H_{38}N_2O_6SNa$, calculated: 801.20635, found: 801.20546; 1.1 ppm error.

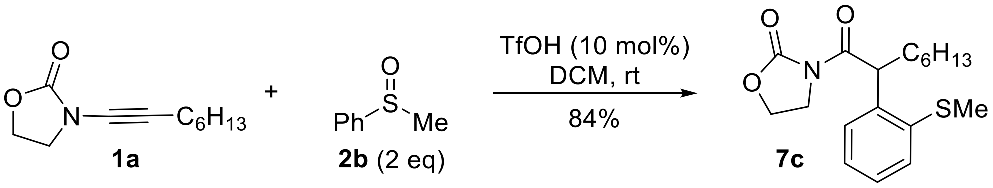

Scheme S10. Reaction of ynamide **1a** with diphenyl sulfoxide **2b**.

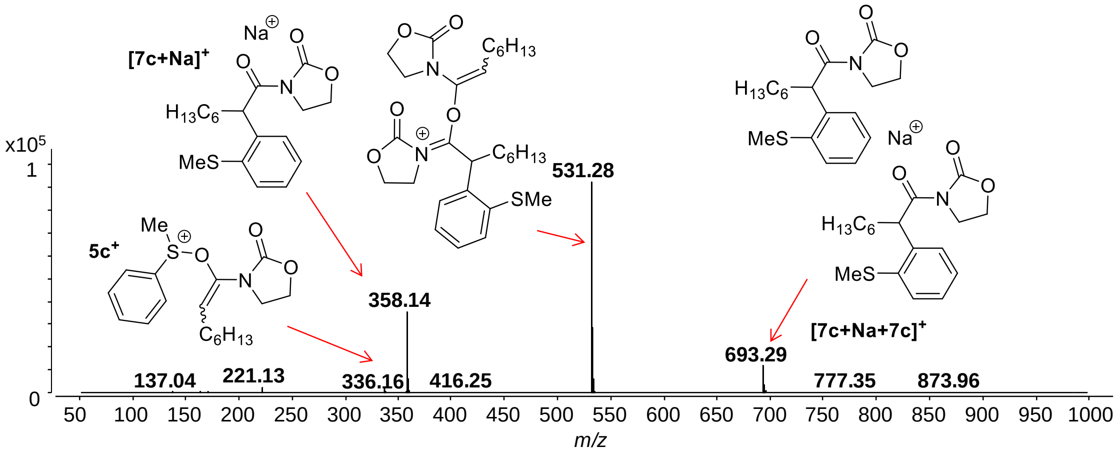

Figure S23. ESI(+)-MS immediately recorded after ynamide **1a** and diphenyl sulfoxide **2b** are mixed in the presence of a catalytic amount of TfOH in DCM.

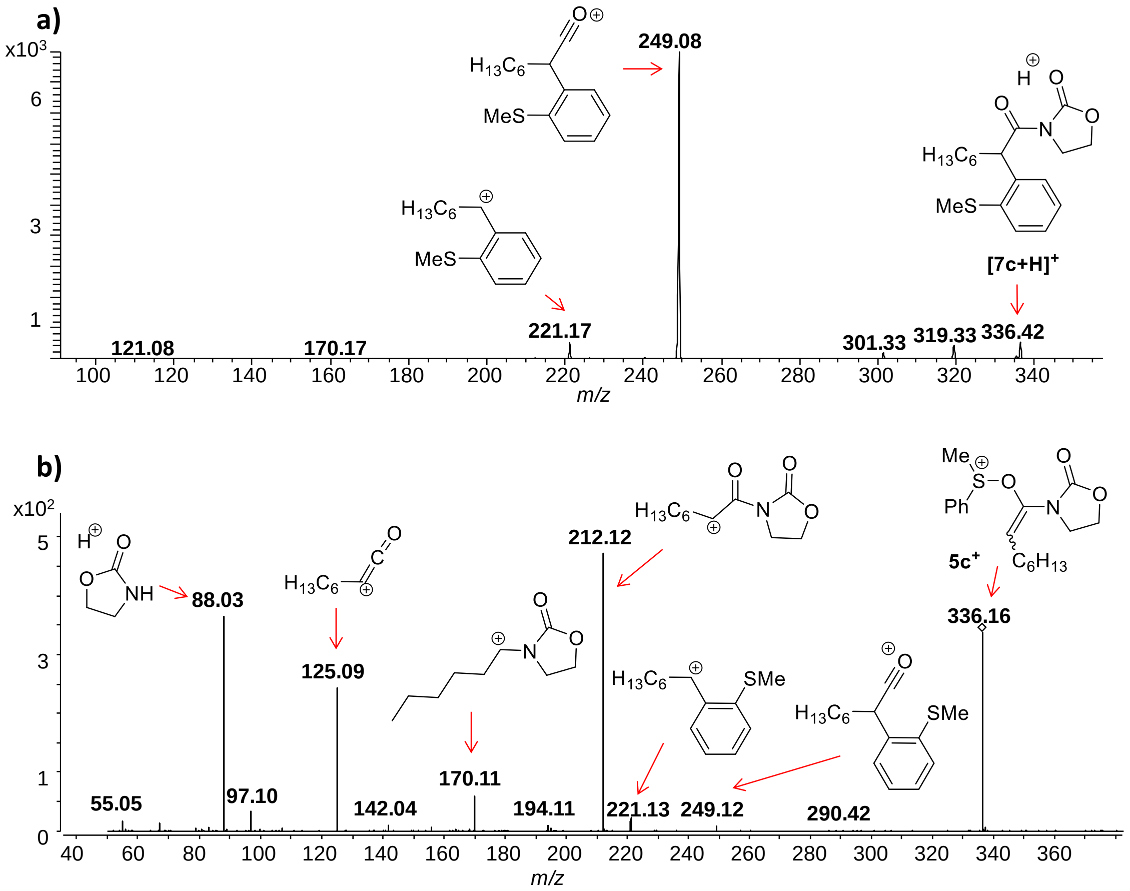

Figure S24. a) ESI(+)-MS/MS of *m/z* 336 analyzed from a) the isolated compound, which is assigned to to [**7c** + H]$^+$, and **b)** from the reaction mixture, which is assigned to **5c**$^+$.

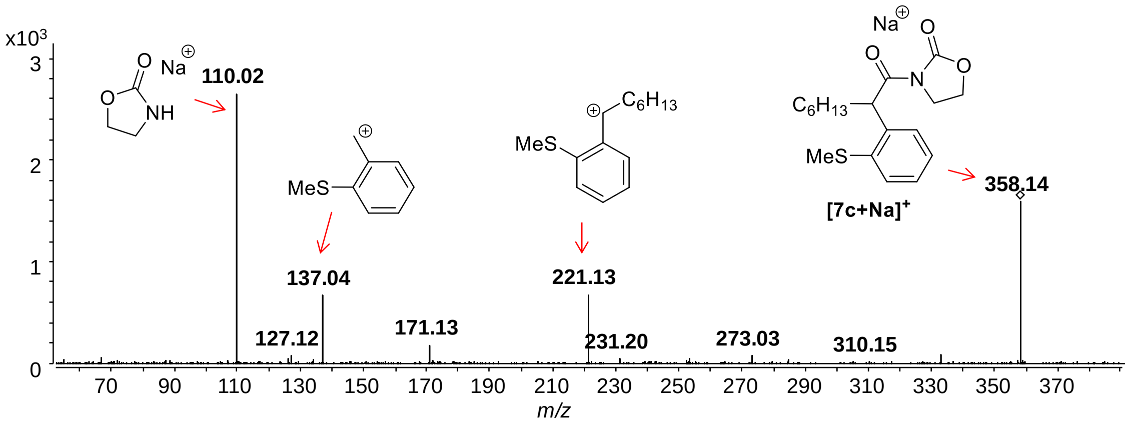

Figure S25. ESI(+)-MS/MS of [**7c**+Na]$^+$: $C_{18}H_{25}NO_3SNa$, calculated: 358.14474, found: 358.14374; 2.79 ppm error.

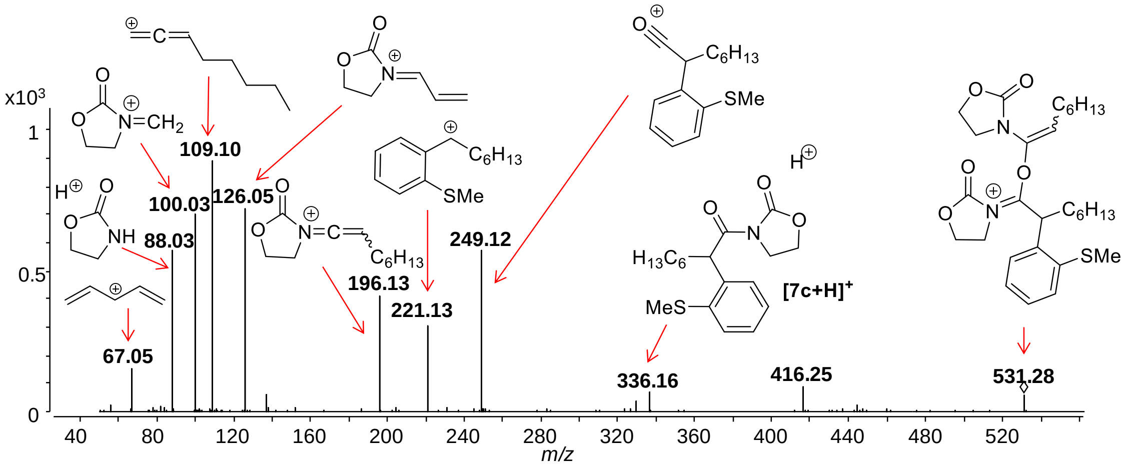

Figure S26. ESI(+)-MS/MS of **13**$^{+}$: $C_{29}H_{43}N_2O_5S$, calculated: 531.28872, found: 531.28710; 3.1 ppm error.

Furthermore, we also monitored by ESI(+)-MS the attempted TfOH-catalyzed Ficini-Claisen reaction of ynamides **1a** and **1b** with both allyl (**20**) and benzyl (**21**) alcohols, which could potentially lead to the corresponding alkylated amides **22** or **23**, respectively (Scheme S2). Although these transformations have been reported to fail at room temperature[1] we hypothesized that ESI(+)-MS analysis of the reaction mixtures resulting thereof could still provide insightful observations. In the event, when alcohols **20** or **21** are used as nucleophilic traps, both hydrated ynamides **8a** and **8b** are the major species identified. Interestingly, the corresponding vinylallyl/ vinylbenzyl ether intermediates can also be detected, but appear to be unsuitable for productive rearrangement. These results can be rationalized by assuming that, without a thermodynamically favourable subsequent C-C bond-forming event, formation of vinylallyl ether and related intermediates is probably reversible and eventually is thwarted by addition of adventitious water. Even after prolonged reaction times (*ca.* 5h), these MS-detected intermediates fail to afford any of the expected corresponding α-allyl or benzyl amides **22** or **23**, respectively.

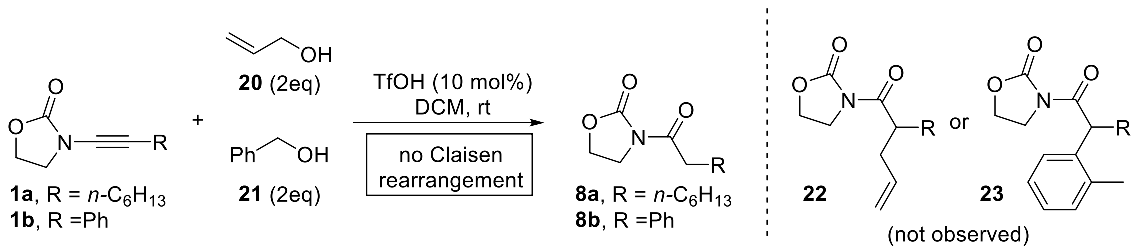

Scheme S11. Ficini-Claisen rearrangement monitored by MS.

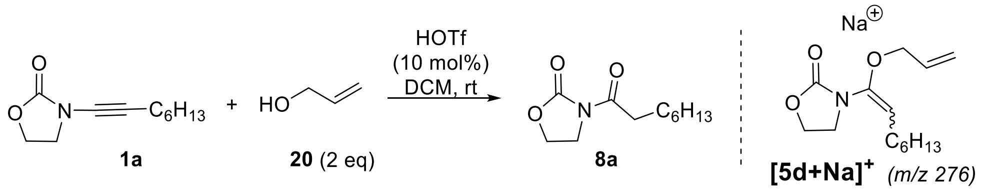

Scheme S12. Reaction of ynamide **1a** with allyl alcohol **20**.

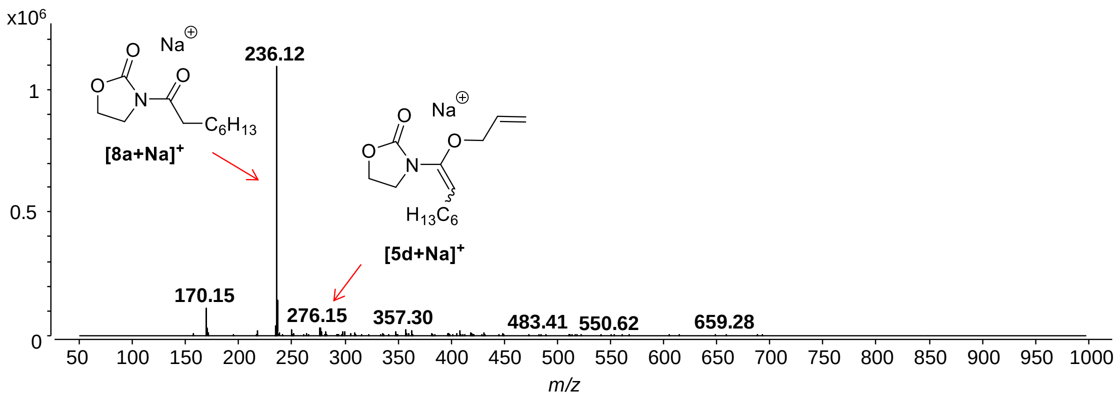

Figure S27. ESI(+)-MS immediately recorded after ynamide **1a** and allyl alcohol **20** are mixed in the presence of a catalytic amount of TfOH in DCM.

It was not possible to acquire the ESI(+)-MS/MS spectrum of *m/z* 276, assigned to [**5d**+Na]$^+$, due to the low abundance of this signal.

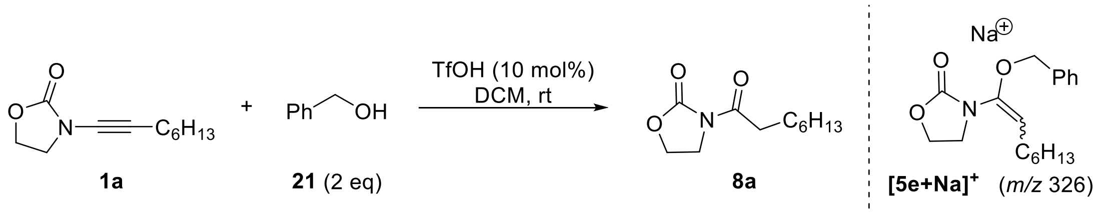

Scheme S13. Reaction of ynamide **1a** with benzyl alcohol **21**.

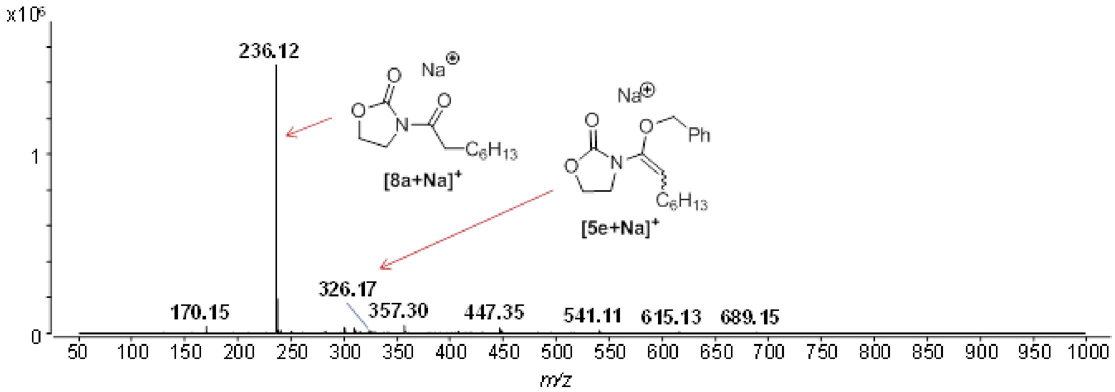

Figure S28. ESI(+)-MS immediately recorded after ynamide **1a** and benzyl alcohol **21** are mixed in the presence of a catalytic amount of TfOH in DCM.

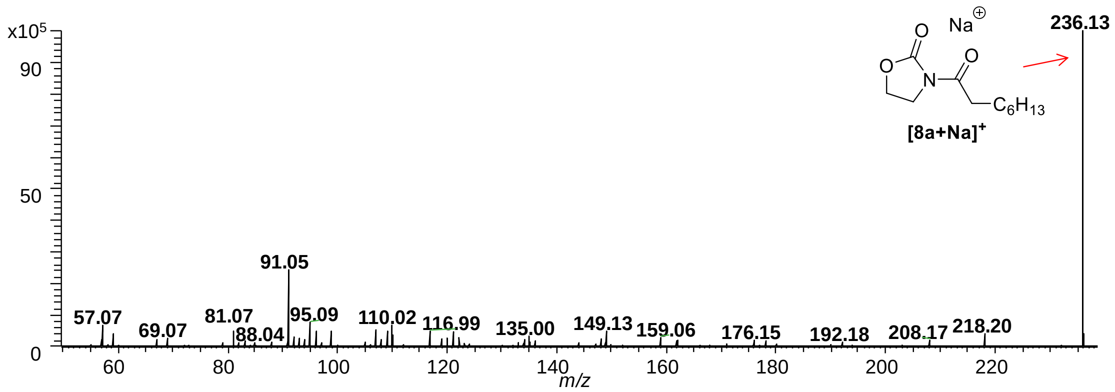

Figure S29. ESI(+)-MS/MS of [**8a**+Na]$^+$: $C_{11}H_{19}NO_3Na$, calculated: 236.12571, found: 236.12609, 1.61 ppm error.

It was not possible to acquire the ESI(+)-MS/MS spectrum of *m/z* 326, assigned to [**5e**+Na]$^+$, due to the low abundance of this signal.

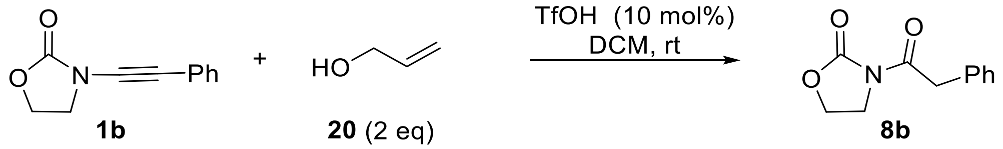

Scheme S14. Reaction of ynamide **1b** with allyl alcohol **20**.

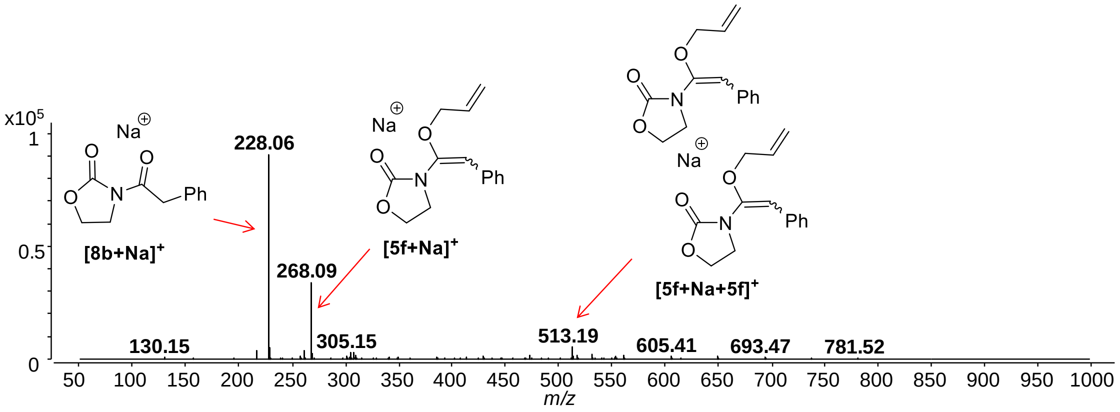

Figure S30. Full ESI(+)-MS recorded after 4h30 of reaction between ynamide **1b** and allyl alcohol **10**, in the presence of a catalytic amount of TfOH in DCM.

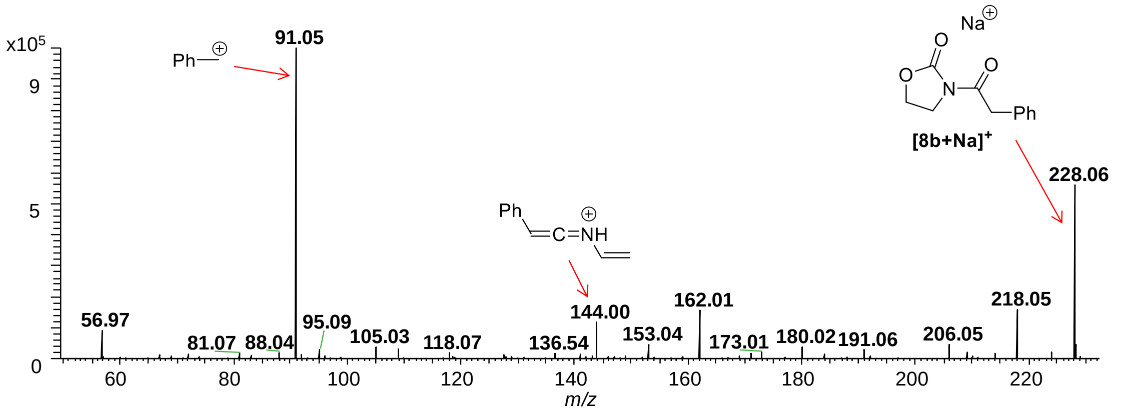

Figure S31. ESI(+)-MS/MS of [**8b**+Na]$^+$: $C_{11}H_{11}NO_3Na$, calculated: 228.06311, found: 228.06315, 0.18 ppm error.

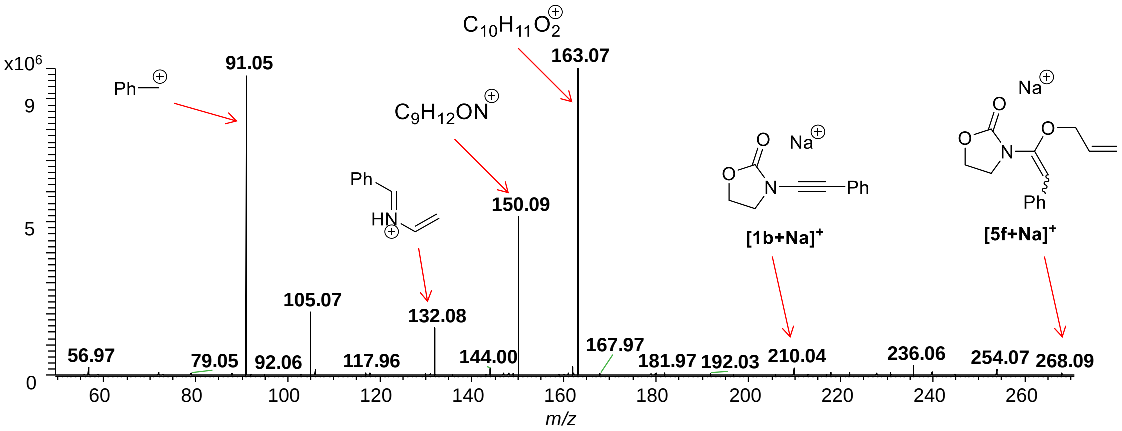

Figure S32. ESI(+)-MS/MS of [**5f**+Na]$^+$: $C_{14}H_{15}NO_3Na$, calculated: 268.09441, found: 268.09377, 2.39 ppm error.

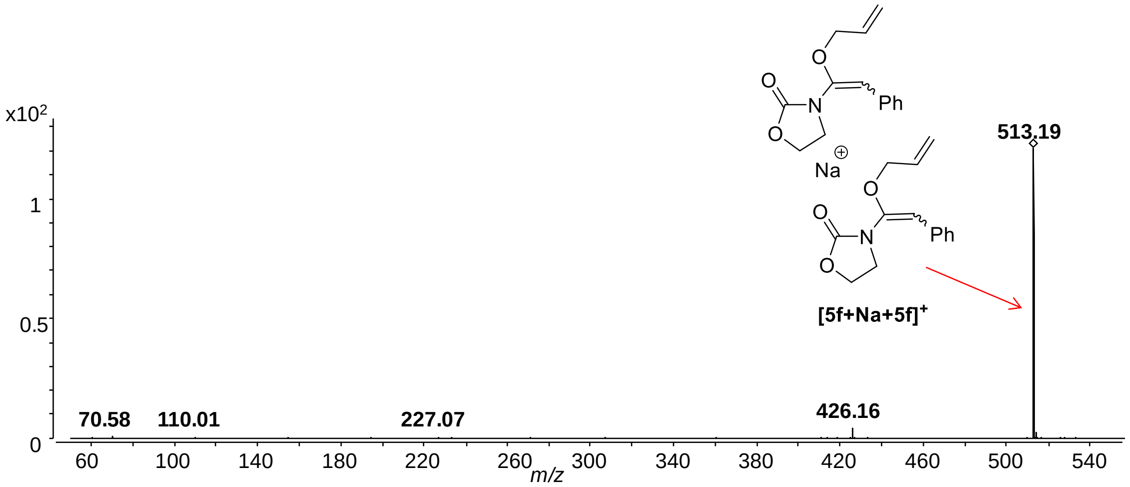

Figure S33. ESI(+)-MS/MS of [**5f**+Na+**5f**]$^+$: $C_{28}H_{30}N_2O_6Na$, calculated: 513.19961, found: 513.19850; 2.16 ppm error**.**

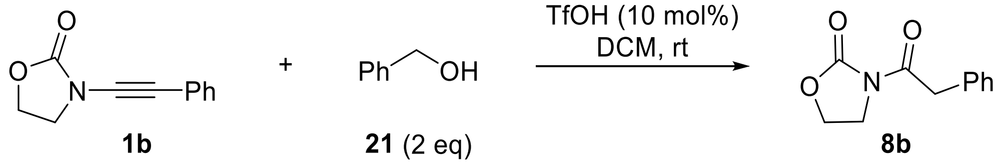

Scheme S15. Reaction of ynamide **1b** with benzyl alcohol **21**.

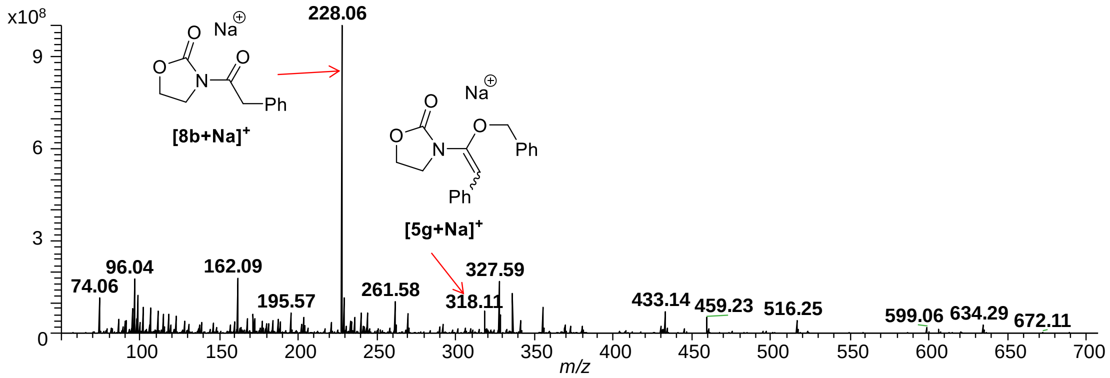

Figure S34. Full ESI-MS(+) recorded after 4h30 of reaction between ynamide **1a** and benzyl alcohol **21**, in the presence of a catalytic amount of TfOH in DCM.

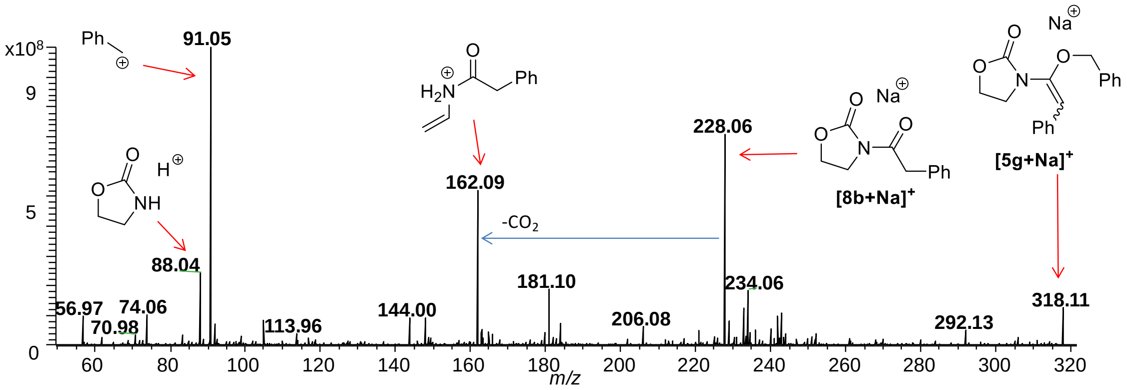

Figure S35. ESI(+)-MS/MS of [**5g**+Na]$^+$: $C_{18}H_{17}NO_3Na$, calculated: 318.11006, found: 318.11070; 2.01 ppm error.

4.General procedures and characterisation of compounds

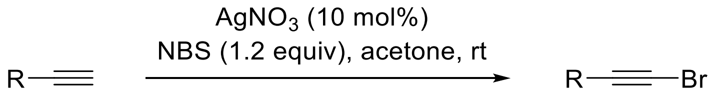

General procedure A: A round bottom flask is charged with a terminal alkyne (1 eq), *N*-bromosuccimide (1.2 eq), acetone (1M in respect to the alkyne), and $AgNO_3$ (10 mol %). The reaction mixture is allowed to stir at room temperature protected from light for 3h. Upon reaction completion (*cf.* TLC), the reaction mixture is concentrated under reduced pressure, diluted in H_2O, extracted with AcOEt (3x), dried ($MgSO_4$) and concentrated under reduced pressure. Purification by flash column chromatography affords the corresponding bromoalkyne in the stated yields.

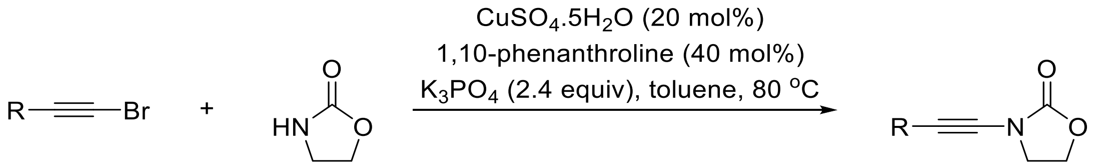

1

General Procedure B:[2] A round bottom flask is charged with a bromoalkyne (1 eq), 2-oxazolidone (1.2 eq), $CuSO_4.5H_2O$ (20 mol%), 1,10-phenanthroline (40 mol%), K_3PO_4 (2.4 eq) and toluene (0.33M in respect to bromoalkyne). The reaction is closed with a septum and allowed to stir at 80 °C for 48h. The reaction is then filtered through celite and concentrated under reduced pressure. Purification by flash column chromatography affords the corresponding ynamides **1** in the stated yields.

3-(oct-1-yn-1-yl)oxazolidin-2-one **1a**

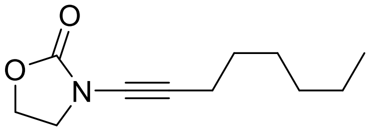

General procedure A is employed with 1-octyne (368 μL, 2.5 mmol, 1 eq), NBS (534 mg, 3 mmol, 1.2 eq), acetone (2.5 mL, 1M) and $AgNO_3$ (43 mg, 0.25 mmol, 10 mol%). Purification by flash column chromatography (hexanes) affords the corresponding 1-bromo-1-octyne as a transparent oil (423 mg, 90%). Next, **general procedure B** is employed with 1-bromo-1-octyne (382 mg, 2 mmol, 1 eq), 2-oxazolidone (209 mg, 2.4 mmol, 1.2 eq), $CuSO_4.5H_2O$ (100 mg, 0.4 mmol, 40 mol%), 1,10-phenanthroline (144 mg, 0.8 mmol, 40 mol%), K_3PO_4 (1.02 g, 4.8 mmol, 2.4 eq) and toluene (6 mL, 0.33M). Purification by flash column chromatography (SiO_2, gradient: Hex - 9/1 Hex:AcOEt - 8/2 Hex:AcOEt - 7/3 Hex:AcOEt) affords the title compound as a transparent oil (169 mg, 43%).

^{1}H NMR (500 MHz, $CDCl_3$): 4.39 (t, *J* = 8.0Hz, 2H), 3.86 (t, *J* = 8.0Hz, 2H), 2.28 (t, *J* = 7.2Hz, 2H), 1.53-1.48 (m, 2H), 1.39-1.33 (m, 2H), 1.31-1.24 (m, 4H), 0.87 (t, *J* = 7.1Hz, 3H).

^{13}C NMR (125 MHz, $CDCl_3$): 156.6, 71.2, 69.9, 62.7, 47.0, 31.3, 28.7, 28.5, 22.5, 18.4, 14.0.

HRMS: Calcd. for $[C_{11}H_{17}NO_2+H]^+$: 196.1332 Found: 196.133.

3-(phenylethynyl)oxazolidin-2-one **1b**

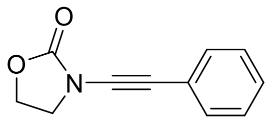

General procedure A is employed with phenylacetylene (274 μL, 2.5 mmol, 1 eq), NBS (534 mg, 3 mmol, 1.2 eq), acetone (2.5 mL, 1M) and $AgNO_3$ (43 mg, 0.25 mmol, 10 mol%). Purification by flash chromatography (SiO_2, hexanes) affords the corresponding (bromoethynyl)benzene as a pale yellow solid (360 mg, 80%). Next, **general procedure B** is employed with (bromoethynyl)benzene (360 mg, 2 mmol, 1 eq), 2-oxazolidone (209 mg, 2.4 mmol, 1.2 eq), $CuSO_4.5H_2O$ (100 mg, 0.4 mmol, 40 mol%), 1,10-phenanthroline (144 mg, 0.8 mmol, 40 mol%), K_3PO_4 (1.02 g, 4.8 mmol, 2.4 eq) and toluene (6 mL, 0.33M). Purification by flash column chromatography (SiO_2, gradient: Hex - 9/1 Hex:AcOEt - 8/2 Hex:AcOEt - 7/3 Hex:AcOEt) affords the title compound as a transparent oil (183 mg, 49%).

^{1}H NMR (400 MHz, $CDCl_3$): 7.45-7.43 (m, 2H), 7.32-7.29 (m, 3H), 4.51-4.47 (m, 2H), 4.03-3.99 (m, 2H).

^{13}C NMR (100 MHz, $CDCl_3$): 155.9, 131.5, 128.2, 128.1, 122.1, 78.9, 71.1, 46.9. **HRMS**: Calcd. for $[C_{11}H_9NO_2+H]^+$: 188.0701 Found: 188.0706.

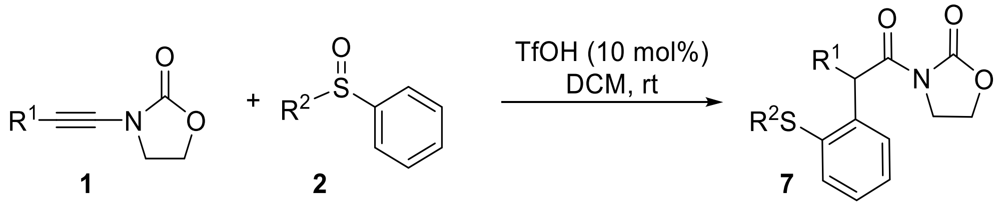

General procedure C:[3] A 4mL-vial is charged with the ynamide **1** (1 eq), the sulfoxide **2** (2 eq) and DCM (0.1M in respect to the ynamide). TfOH (10 mol%) is added and the reaction mixture is allowed to stir at room temperature. Upon reaction completion (*cf.* TLC), the reaction is concentrated under reduced pressure and purified by flash column chromatography to afford the corresponding arylated amides **7** in the stated yields.

3-(2-(2-(phenylthio)phenyl)octanoyl)oxazolidin-2-one **7a**

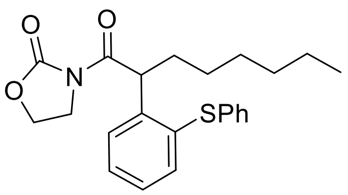

General procedure C is employed with 3-(oct-1-yn-1-yl)oxazolidin-2-one **1a** (20 mg, 0.1 mmol, 1 eq), diphenyl sulfoxide **2a** (40 mg, 0.2 mmol, 2 eq), TfOH (1 µL, 10 mol%) and DCM (1mL). Purification by flash column chromatography (SiO_2, gradient: Hex-9:1 Hex/AcOEt - 85:15 Hex/AcOEt) affords the corresponding arylated amide as a colorless oil (36 mg, 90%).

^{1}H NMR (500 MHz, $CDCl_3$): 7.50 (ddd, *J* = 7.6Hz, *J* = 3.5Hz, *J* = 1.2Hz, 2H), 7.40 (td, *J* = 7.6Hz, *J* = 1.2Hz, 1H), 7.29-7.23 (m, 3H), 7.18-7.14 (m, 3H), 5.68 (dd, *J* = 7.6Hz, *J* = 7.0, 1H), 4.33-4.28 (m, 1H), 4.15-4.10 (m, 1H), 3.93-3.88 (m, 1H), 3.66-3.61 (m, 1H), 2.07-2.00 (m, 1H), 1.83-1.76 (m, 1H), 1.33-1.20 (m, 8H), 0.86 (t, *J* = 7.0Hz, 3H). **^{13}C NMR (125 MHz, $CDCl_3$):** 174.0, 152.5, 141.1, 137.8, 135.3, 134.1, 129.2, 128.9, 128.8, 128.7, 127.8, 125.7, 61.5, 46.3, 42.7, 33.4, 31.5, 29.0, 27.5, 22.5, 14.0.

HRMS: Calcd. for $[C_{23}H_{27}NO_3S+Na]^+$: 420.1604 Found: 420.1608.

3-(2-phenyl-2-(2-(phenylthio)phenyl)acetyl)oxazolidin-2-one[3] **7b**

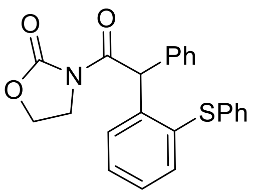

General procedure C is employed with 3-(phenylethynyl)oxazolidin-2-one **1b** (19 mg, 0.1 mmol, 1 eq), diphenyl sulfoxide **1a** (40 mg, 0.2 mmol, 2 eq), TfOH (1 µL, 10 mol%) and DCM (1 mL). Purification by flash column chromatography (SiO_2, gradient: Hex-9:1 Hex/AcOEt - 8:2 Hex/AcOEt) affords the corresponding arylated amide as a colorless oil (20 mg, 55%).

^{1}H NMR (400 MHz, $CDCl_3$): 7.40 (dd, *J* = 7.4Hz, *J* = 1.5Hz, 1H), 7.28-7.14 (m, 9H), 7.09-7.03 (m, 4H), 6.96 (s, 1H), 4.19-4.10 (m, 2H), 3.83-3.76 (m, 1H), 3.64-3.57 (m, 1H).

^{13}C NMR (100 MHz, CDCl$_3$): 171.6, 152.3, 141.9, 137.2, 136.8, 135.4, 133.1, 130.9, 129.6, 128.9 (x2), 128.7 (x2), 128.1, 127.5, 126.0, 61.5, 52.6, 42.7.

HRMS: Calcd. for $[C_{23}H_{19}NO_3S+Na]^+$: 412.0978 Found: 412.0977.

3-(2-(2-(methylthio)phenyl)octanoyl)oxazolidin-2-one **7c**

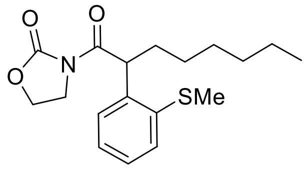

General procedure C is employed with 3-(oct-1-yn-1-yl)oxazolidin-2-one **1a** (20 mg, 0.1 mmol, 1 eq), methyl phenyl sulfoxide **2b** (40 mg, 0.2 mmol, 2 eq), TfOH (1 µL, 10 mol%) and DCM (1 mL). Purification by flash column chromatography (SiO_2, gradient: Hex-9:1 Hex/AcOEt - 85:15 Hex/AcOEt) affords the corresponding arylated amide as a colorless oil (28 mg, 84%).

^{1}H NMR (400 MHz, CDCl$_3$): 7.29-7.26 (m, 1H), 7.26-7.21 (m, 2H), 7.15-7.11 (m, 1H), 5.36 (dd, J = 8.0Hz, J = 6.2Hz, 1H), 4.42-4.30 (m, 2H), 4.11-3.97 (m, 2H), 2.48 (s, 3H), 2.08-1.99 (m, 1H), 1.80-1.71 (m, 1H), 1.38-1.24 (m, 8H), 0.85 (t, J = 6.9Hz, 3H). **^{13}C (100 MHz, CDCl$_3$):** 174.1, 152.8, 138.3, 137.6, 127.6, 127.3, 126.9, 125.4, 61.8, 46.3, 42.9, 33.3, 31.6, 29.2, 27.9, 22.6, 16.8, 14.1.

HRMS: Calcd. for $[C_{18}H_{25}NO_3S+Na]^+$: 358.1447 Found: 358.1437.

3-(2-bromo-2-phenylacetyl)oxazolidin-2-one **13**

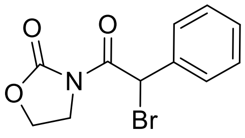

Compound **13** was prepared according to previously described procedure.[4]

^{1}H NMR (700 MHz, CDCl$_3$): 7.62 (dd, J = 7.8, 1.3 Hz, 2H), 7.48 – 7.33 (m, 3H), 6.90 (s, 1H), 4.48 (td, J = 9.1, 6.9 Hz, 1H), 4.41 (td, J = 9.1, 6.8 Hz, 1H), 4.14 (ddd, J = 11.0, 9.4, 6.7 Hz, 1H), 4.03 (ddd, J = 11.1, 9.4, 6.9 Hz, 1H) ppm;

3-(2-phenyl-2-(4-(phenylthio)phenyl)acetyl)oxazolidin-2-one **11**

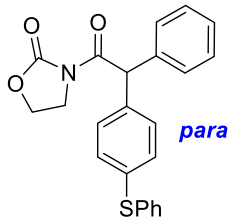

To a 3-(2-bromo-2-phenylacetyl)oxazolidin-2-one **10** (70 mg, 0.246 mmol, 1.0 equiv) and phenyl sulfide (45.9 mg, 0.246 mmol, 1.0 equiv) in DCM (2 mL) at rt was added silver hexafluoroantimonate (V) (127 mg, 0.37 mmol, 1.5 equiv) and reaction was stirred overnight. The reaction was stopped by addition of sat. $NaHCO_3$ solution and extracted with DCM (3 x 10 mL). The organic phases were dried with MgSO4 and the solvent removed under reduced pressure. Purification by flash chromatography gave final product (35 mg, 36%) as a colorless crystals.

^{1}H NMR (400 MHz, $CDCl_3$) δ 7.37 – 7.23 (14 H, m), 6.49 (s, 1H), 4.38 (m, 2H), 4.07 (t, *J* = 8.02 Hz, 2H).

^{13}C NMR (151 MHz, $CDCl_3$) δ 172.13, 152.95, 137.87, 137.00, 135.24, 135.09, 131.55, 130.57, 129.91, 129.23, 129.08, 128.68, 127.50, 127.30, 61.75, 53.55, 43.00.

IR (thin film) ν 1774, 1683, 1390, 1362, 1211, 1113, 1020 cm^{-1}.

HRMS (ESI) $[M+Na]^+$ calcd for $C_{23}H_{19}NO_3SNa$: 412.0978, found 412.0970.

Structure confirmed by X-ray crystallographic data (see SI section 7 for the details).

3-(2-(2,4-dimethyl-3-(methylthio)phenyl)-2-phenylacetyl)oxazolidin-2-one **14**

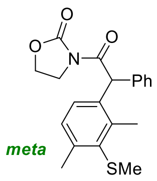

To a ynamide **1b** (73 mg, 0.39 mmol, 1.0 equiv) in DCM (4 mL) was added 2,6-dimethylphenylmethyl sulfoxide **13** (726 mg, 3.9 mmol, 10 equiv) followed by 2,6-dimethlphenyl-methylsulfoxide (98.4 mg, 10.585 mmol, 15 equiv) and TfOH (5.6 uL, 0.04 mmol, 10 mol%) and reaction was stirred overnight at rt. The reaction was stopped by addition of sat. $NaHCO_3$ solution and extracted with DCM (3 x 10 mL). The organic phases were dried with $MgSO_4$ and the solvent removed under reduced pressure. Purification by flash chromatography gave final product **14** (24 mg, 17%) and **15** (40 mg, 46%).

^{1}H NMR (600 MHz, $CDCl_3$) δ 7.34 (t, *J* = 7.5 Hz, 2H), 7.30 (d, *J* = 7.3 Hz, 1H), 7.25 (d, *J* = 7.4 Hz, 2H), 7.09 (d, *J* = 7.7 Hz, 1H), 7.02 (d, *J* = 7.9 Hz, 1H), 6.60 (s, 1H), 4.40 (dd, *J* = 12.2, 5.4 Hz, 2H), 4.18 – 3.96 (m, 2H), 2.63 (s, 3H), 2.56 (s, 3H), 2.21 (s, 3H).

^{13}C NMR (151 MHz, $CDCl_3$) δ 172.58, 152.90, 141.91, 141.73, 137.01, 136.62, 135.07, 129.67, 128.54, 127.75, 127.55, 127.40, 61.77, 52.77, 43.02, 22.27, 18.63, 17.72.

IR (thin film) ν 1769, 1692, 1451, 1383, 1201, 1107, 1031 cm^{-1}.

HRMS (ESI) $[M+Na]^+$ calcd for $C_{20}H_{21}NO_3SNa$: 378.1134, found 378.1125.

3-(2-hydroxyethyl)-5-phenyloxazolidine-2,4-dione **15**

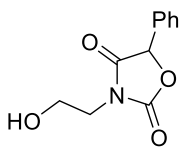

Product **15** is prepared according to procedure for **14**, being isolated in 46% yield.

^{1}H NMR (600 MHz, $CDCl_3$) δ 7.49 – 7.44 (m, 5H), 5.80 (s, 1H), 3.95 – 3.90 (m, 2H), 3.85 – 3.81 (m, 2H), 1.94 (t, *J* = 5.9 Hz, 1H).

^{13}C NMR (151 MHz, $CDCl_3$) δ 171.72, 155.54, 131.38, 129.94, 129.18, 126.22, 80.47, 59.66, 42.94.

IR (thin film) ν 1729, 1512, 1411, 1283, 1095, 1011 cm^{-1}.

HRMS (ESI) $[M+Na]^+$ calcd for $C_{11}H_{11}NO_4Na$: 244.0580, found 244.0574.

3-(2-phenyl-2-(2,4,6-trimethyl-3-(methylthio)phenyl)acetyl)oxazolidin-2-one **14b**

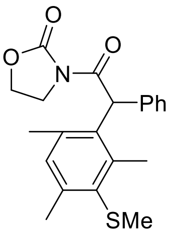

Product **14b** prepared according to procedure for the **14** in a15% yield.

^{1}H NMR (600 MHz, $CDCl_3$) δ 7.36 – 7.25 (m, 3H), 7.03 (d, *J* = 7.4 Hz, 2H), 6.98 (s, 1H), 6.40 (s, 1H), 4.45 (td, *J* = 9.1, 4.8 Hz, 1H), 4.35 (q, *J* = 8.8 Hz, 1H), 4.21 (dt, *J* = 18.3, 9.1 Hz, 1H), 4.05 (ddd, *J* = 11.0, 9.1, 4.9 Hz, 1H), 2.55 (s, 3H), 2.55 (s, 3H), 2.21 (s, 3H), 2.19 (s, 3H).

^{13}C NMR (151 MHz, $CDCl_3$) δ 173.92, 152.50, 142.54, 141.82, 138.33, 136.86, 134.12, 133.79, 131.05, 128.76, 128.43, 126.97, 61.97, 53.39, 43.47, 22.14, 21.31, 19.23, 18.77.

IR (thin film) ν 1782, 1381, 1216, 1195, 1103, 1036 cm^{-1}.

HRMS (ESI) $[M+Na]^+$ calcd for $C_{21}H_{23}NO_3SNa$: 392.1291, found 392.1283.

3-(2-(4-methoxyphenyl)-2-phenylacetyl)oxazolidin-2-one **16**

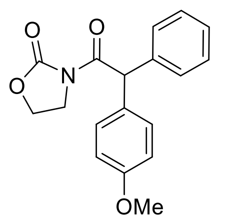

To a ynamide **1b** (50 mg, 0.141 mmol, 1.0 equiv) in DCM (2 mL) and anisole (2 mL) at rt was added 2,6-dimethylphenylmethyl sulfoxide **13** (23.7 mg, 0.141 mmol, 1.0 equiv) and TfOH (2.4 uL, 0.271 mmol, 20 mol%) and mixture was stirred utill starting material was consumed. The reaction was quenched by addition of sat. $NaHCO_3$ solution and extracted with DCM (3 x 10 mL). The organic phases were dried with $MgSO_4$ and the solvent removed under reduced pressure. Purification by flash chromatography gave final product **16** (20 mg, 46%) and **15** (6 mg, 19%) and **14** (6 mg, 13%)

^{1}H NMR (600 MHz, $CDCl_3$) δ 7.38 – 7.26 (m, 7H), 6.88 (d, *J* = 8.8 Hz, 2H), 6.49 (s, 1H), 4.40 (t, *J* = 8.1 Hz, 2H), 4.11 – 4.04 (m, 2H), 3.80 (s, 3H).

^{13}C NMR (151 MHz, $CDCl_3$) δ 172.66, 158.85 (2x), 152.97, 138.59, 130.25, 128.97, 128.56, 127.25, 113.99, 61.72, 55.25, 53.20, 43.01.

IR (thin film) ν 1773, 1694, 1382, 1219, 1106, 1039 cm^{-1}.

HRMS (ESI) $[M+Na]^+$ calcd for $C_{18}H_{17}NO_4Na$: 334.1050, found 334.1042.

Synthesis of sulfoxides

Sulfoxides **17a** and **17b** are prepared according to following scheme:

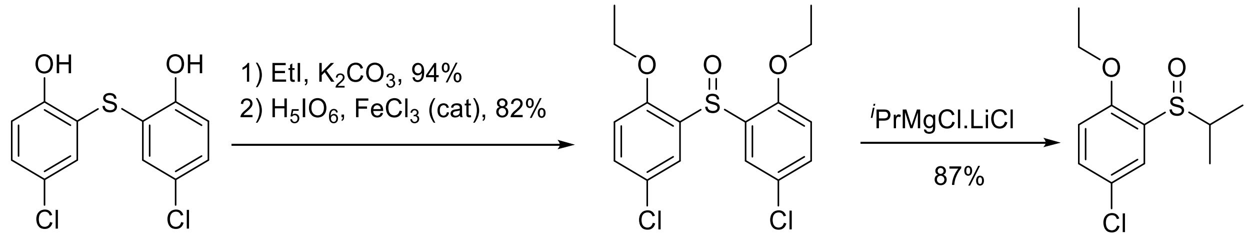

TCI Chemicals **17b** **17a**

Sulfoxides **17e**,[5] **17f**,[6] **17i**[7] and **17j**[8] prepared according to known literature methods

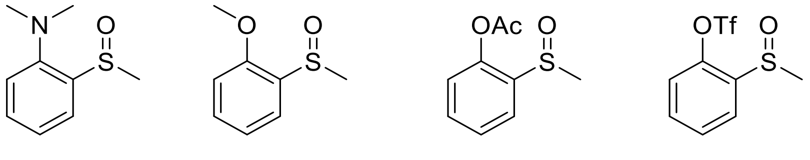

17e **17f** **17i** **17j**

4-chloro-1-ethoxy-2-(isopropylsulfinyl)benzene **17a**

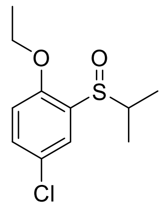

A 2,2'-sulfinylbis(4-chloro-1-ethoxybenzene) **17b** (1.02 g, 2.84 mmol, 1.0 equiv) was dissolved in THF (30 mL) and cooled to – 20 °C. To that solution was added ^{i}PrMgBr.LiCl solution (6.2 mL, 1M solution in THF, 2.2 equiv) and reaction mixture was stirred at that temperature for 1 h. Reaction was stopped by the addition of sat. NH_4Cl solution and extracted with Et_2O. Organic layers were dried with the Na_2SO_4 and evaporated *in vacuo*. Crude mixture was purified by column chromatography (heptane/ethyl acetate 90:10 to 60:40) to give final product **17a** (492 mg, 70%).

^{1}H NMR (400 MHz, $CDCl_3$) δ 7.70 (d, *J* = 2.6 Hz, 1H), 7.37 (dd, *J* = 8.7, 2.7 Hz, 1H), 6.84 (d, *J* = 8.7 Hz, 1H), 4.20 – 4.02 (m, 2H), 3.15 (dt, *J* = 13.9, 6.9 Hz, 1H), 1.44 (t, *J* = 7.0 Hz, 3H), 1.44 (d, *J* = 7.1 Hz, 2H), 1.03 (d, *J* = 6.8 Hz, 2H).

^{13}C NMR (101 MHz, $CDCl_3$) δ 153.28, 132.26, 131.45, 126.95, 126.27, 112.66, 77.33, 77.01, 76.69, 64.67, 50.98, 17.35, 14.54, 12.53.

IR (thin film) ν 2976, 2932, 1461, 1258, 1038 cm^{-1}.

HRMS (ESI) $[M+Na]^+$ calcd for $C_{11}H_{15}O_2SClNa$: 269.0379, found 269.0371.

2,2'-sulfinylbis(4-chloro-1-ethoxybenzene) **17b**

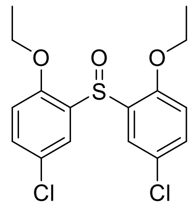

Step 1: 2,2'-thiobis(4-chlorophenol) (2.87 g, 1.0 equiv, 10.0 mmol) was dissolved in acetone (50 mL) and K_2CO_3 (4.14 g, 30 mmol, 3.0 equiv) was added followed by the EtI (2.0 mL, 25 mmol, 2.5 equiv) and reaction was stirred for 24 h at rt. Mixture was filtered through the celite and evaporated *in vacuo* to give NMR clean product (3.50 g, 97%) that was submitted to next step without further purification.

Step 2: To a bis(5-chloro-2-ethoxyphenyl)sulfane (1.71 g, 5.0 mmol, 1.0 equiv) in MeCN (25 mL) was added periodic acid (1.25 g, 5.5 mmol, 1.1 equiv) and catalytic amounts of $FeCl_3$. Reaction was stirred at rt for 2 h, then filtered through celite and filtrated, followed by evaporation in vacuo. Column chromatography 5 to 30% EtOAc in heptanes gave final product **17b** as an orange solid (1.51 g, 84%).

^{1}H NMR (400 MHz, $CDCl_3$) δ 7.59 (d, *J* = 2.6 Hz, 2H), 7.27 (dd, *J* = 8.7, 2.6 Hz, 2H), 6.72 (d, *J* = 8.8 Hz, 2H), 3.91 (qd, *J* = 7.0, 1.2 Hz, 4H), 1.24 (t, *J* = 7.0 Hz, 6H).

^{13}C NMR (101 MHz, $CDCl_3$) δ 154.61, 133.11, 132.36, 127.50, 126.21, 113.26, 64.77, 14.30.

IR (thin film) ν 3084, 2937, 1486, 1168, 1036 cm^{-1}.

HRMS (ESI) $[M+Na]^+$ calcd for $C_{16}H_{16}O_3SCl_2Na$: 381.0095, found 381.0099.

General procedure for 17d, 17l and S17m

Step 1: Following a reported procedure,[9] in a dry Schlenk methoxyarene (2.5 mmol), TMEDA (2.6 mmol, 1.05 equiv) were dissolved in pentane (2 mL) and cooled in an ice bath. nBuLi (1.6 mL of 1.6M solution in hexane, 2.6 mmol, 1.05 equiv) was added dropwise at 0 °C. After 10 minutes, dimethyl disulfide (0.22 mL, 2.5 mmol, 1.0 equiv) was added slowly and the reaction was stirred at 0 °C for 1 h and at rt for 12 h. The reaction was quenched by addition of diluted sulfuric acid and extracted with ether, dried over Na_2SO_4 and concentrated. The crude residue was purified by column chromatography to give the corresponding sulfide.

Step 2: To a solution of sulfide (0.5 mmol) in DCM (0.3M) at 0 °C was added mCPBA (93 mg, 0.55 mmol, 1.1 equiv). The icebath was removed and the reaction stirred for 1.5 h at rt. The reaction was quenched by addition of sat. $NaHCO_3$ solution, extracted with DCM, dried over Na_2SO_4 and concentrated *in vacuo* to give a crude sulfoxide which was purified by column chromatography.

1-methoxy-4-methyl-2-(methylsulfinyl)benzene **17d**

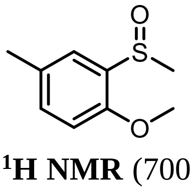

Step 1: 300 mg of 4-methylanisole gave 100 mg (24%, 39% brsm) of sulfide. **Step 2:** 95 mg of sulfide led to 60 mg (58%) of sulfoxide **17d**.

^{1}H NMR (700 MHz, $CDCl_3$) δ 7.64 (d, *J* = 2.0 Hz, 1H), 7.24 (dd, *J* = 8.3, 1.7 Hz, 1H), 6.83 (d, *J* = 8.3 Hz, 1H), 3.87 (s, 3H), 2.78 (s, 4H), 2.39 (s, 3H).

^{13}C NMR (176 MHz, $CDCl_3$) δ 152.68, 132.57, 132.22, 131.43, 124.79, 110.54, 55.75, 41.28, 20.55.

IR (thin film) ν 1493, 1462, 1273, 1244, 1072, 1040, 811 cm^{-1}.

HRMS (ESI): calcd for $[C_9H_{12}O_2S+Na]^+$: 207.0450, found: 207.0450.

2-methoxy-3-(methylsulfinyl)naphthalene **17l**

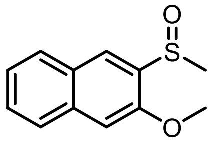

Step 1: 400 mg of 2-methoxynaphthalene gave 125 mg (24%) of sulfide. **Step 2:** 122 mg of sulfide led to 125 mg (95%) of sulfoxide **17l**.

^{1}H NMR (600 MHz, $CDCl_3$) δ 8.31 (s, 1H), 7.91 (d, *J* = 8.1 Hz, 1H), 7.79 (d, *J* = 8.1 Hz, 1H), 7.53 (ddd, *J* = 8.2, 6.9, 1.2 Hz, 1H), 7.43 (ddd, *J* = 8.1, 6.9, 1.1 Hz, 1H), 7.17 (s, 1H), 3.99 (s, 3H), 2.85 (s, 3H).

^{13}C NMR (151 MHz, $CDCl_3$) δ 152.56, 135.48, 134.96, 128.66, 128.47, 127.90, 126.62, 125.65, 124.81, 106.01, 55.79, 41.66.

IR (thin film) ν 2919, 1624, 1592, 1501, 1244, 1031, 748 cm^{-1}.

HRMS (ESI): calcd for $[C_9H_{12}O_2S+Na]^+$: 243.0450, found: 243.0447.

1,3-dimethoxy-2-(methylsulfinyl)benzene **S17m**[10]

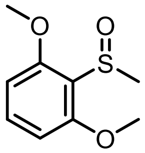

Step 1: 300 mg of 1,3-dimethoxybenzene gave 244 mg (84%) of sulfide.

Step 2: 220 mg of sulfide gave 223 mg (94%) of sulfoxide **S17m**.

^{1}H NMR (400 MHz, $CDCl_3$) δ 7.44 (t, *J* = 8.5 Hz, 1H), 6.65 (d, *J* = 8.5 Hz, 2H), 3.93 (s, 6H), 3.28 (s, 3H).

^{13}C NMR (101 MHz, $CDCl_3$) δ 159.28 (2C), 134.75, 117.85, 105.23 (2C), 56.71 (2C), 46.08.

No meta-arylated product was observed when **S17m** was reacted with ynamide and Tf_2NH under standard condition *vide infra*.

Table S1. Optimization of reaction conditions for α-arylation (Scheme 4)

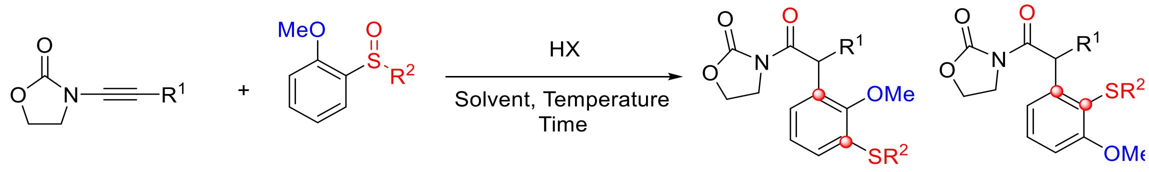

Entry[a]	R^1	R^2	HX	Solvent	T, °C	Time, h	NMR yield **18**:**19** [%][b]
1	$CH_2CH_2CH_2Ph$	Cy	TfOH (50 mol%)	DCM	0	1	12:30
2	$CH_2CH_2CH_2Ph$	Cy	TfOH (50 mol%)	Toluene	0	2	5:24
3[c]	$CH_2CH_2CH_2Ph$	Cy	TfOH (50 mol%)	DCM	0	2	< 20%
4	$CH_2CH_2CH_2Ph$	Cy	TfOH (100 mol%)	DCM	0	2	< 20%
5	Cy	Me	Tf_2NH (50 mol%)	DCM	0	2	21:33
6	Cy	Me	TfOH (50 mol%)	DCM	0	2	15:25

[a] In all experiments: acid added to ynamide ansd sulfoxide in the solvent at given temperature; [b] Yield measured directly on crude reaction mixture with the internal standard 1,3,5-trimethoxybenzene or mesitylene (1.0 equiv); [c] Ynamide added over 1 h to a sulfoxide and TfOH.

Several other conditions were also explored. Change of solvent (toluene, DCE, cyclohexane/DCM), addition of extra water (2 equiv) to stabilize reaction intermediates and use of 2 equiv of sulfoxide and 1 equiv of ynamide led to reaction with < 30% overall NMR yield of **18** and **19** therefore the scope was done according to the conditions shown in table S1 entry 5.

General procedure for the α-arylation (Scheme 4)

In a disposable vial, Tf_2NH (0.125 mmol, 0.5 equiv) was dissolved in DCM (1 mL) and cooled to 0 °C. A solution of ynamide **1** (0.25 mmol, 1.0 equiv) and sulfoxide **17** (0.50 mmol, 2.0 equiv) in DCM (1.0 mL) was added in one portion and the reaction was stirred

at 0 °C for 2 h. Saturated $NaHCO_3$ solution (3 mL) was added and mixture was extracted with DCM (3 x 5 mL). Organic layers were dried with the Na_2SO_4 and evaporated *in vacuo*. The crude mixture was purified by column chromatography (heptane/ethyl acetate) to give the final product.

3-(2-(5-chloro-2-ethoxy-3-(isopropylthio)phenyl)-2-cyclohexylacetyl)oxazolidin-2-one **18a**

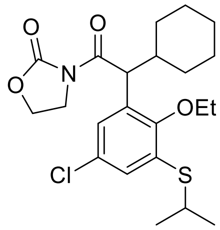

According to general procedure isolated in 30% yield as a white solid.

^{1}H NMR (400 MHz, $CDCl_3$) δ 7.37 (d, *J* = 2.5 Hz, 1H), 7.07 (d, *J* = 2.5 Hz, 1H), 5.40 (d, *J* = 10.4 Hz, 1H), 4.41 – 4.20 (m, 2H), 4.07 – 3.74 (m, 4H), 3.51 – 3.33 (m, 1H), 2.00 – 1.87 (m, 1H), 1.70 – 1.54 (m, 4H), 1.38 (t, *J* = 7.0 Hz, 3H), 1.25 (dd, *J* = 8.6, 6.7 Hz, 6H), 1.26 – 1.03 (m, 5H), 0.96 – 0.86 (m, 1H).

^{13}C NMR (101 MHz, $CDCl_3$) δ 174.49, 154.72, 153.05, 133.20, 132.40, 129.51, 128.23, 126.98, 69.59, 61.48, 46.16, 43.01, 42.78, 35.96, 30.95, 30.67, 26.22, 26.09, 22.94, 22.90, 15.34.

IR (thin film) ν 2982, 2931, 1777, 1697, 1387 cm^{-1}.

HRMS (ESI) $[M+Na]^+$ calcd for $C_{22}H_{30}NO_4SClNa$: 462.1482, found 462.1477.

Structure determined by X-ray analysis (see SI section 7 for the further details).

3-(2-(5-chloro-3-((5-chloro-2-ethoxyphenyl)thio)-2-ethoxyphenyl)-2-cyclohexylacetyl)oxazolidin-2-one **18b**

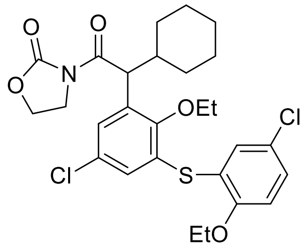

According to general procedure isolated in 27% yield as a transparent oil.

^{1}H NMR (600 MHz, $CDCl_3$) δ 7.50 (d, J = 2.5 Hz, 1H), 7.23 (dd, J = 8.7, 2.6 Hz, 1H), 7.15 (d, J = 2.6 Hz, 1H), 6.83 (d, J = 2.6 Hz, 1H), 6.82 (d, J = 8.8 Hz, 1H), 5.49 (d, J = 10.4 Hz, 1H), 4.41 (td, J = 9.2, 5.4 Hz, 1H), 4.34 (q, J = 8.8 Hz, 1H), 4.16 – 3.91 (m, 6H), 2.06 – 2.02 (m, 1H), 1.79 – 1.61 (m, 4H), 1.42 (t, J = 6.9 Hz, 3H), 1.28 (t, J = 7.0 Hz, 3H), 1.32 – 1.13 (m, 5H), 1.02 – 0.95 (m, 1H).

^{13}C NMR (151 MHz, $CDCl_3$) δ 174.40, 156.13, 154.60, 153.08, 133.43, 132.53, 130.80, 129.67, 129.09, 129.01, 128.08, 125.66, 123.31, 113.07, 70.04, 64.62, 61.51, 46.12, 43.01, 42.71, 30.95, 30.58, 26.15, 26.03, 15.34, 14.47.

IR (thin film) ν 2979, 2925, 2852, 1776, 1692, 1244 cm^{-1}.

HRMS (ESI) $[M+Na]^+$ calcd for $C_{27}H_{31}Cl_2NO_5SNa$: 574.1198, found 574.1199.

5-(5-chloro-3-((5-chloro-2-ethoxyphenyl)thio)-2-ethoxyphenyl)-6-oxo-6-(2-oxooxazolidin-3-yl)hexanenitrile **18c**

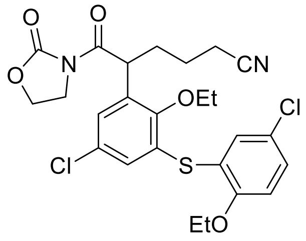

According to general procedure isolated in 28% yield as a transparent oil.

^{1}H NMR (400 MHz, $CDCl_3$) δ 7.17 (dd, *J* = 8.7, 2.6 Hz, 1H), 7.10 (d, *J* = 2.6 Hz, 1H), 7.09 (d, *J* = 2.6 Hz, 1H), 6.82 (d, *J* = 2.5 Hz, 1H), 6.76 (d, *J* = 8.8 Hz, 1H), 5.30 (t, *J* = 7.2 Hz, 1H), 4.49 – 4.26 (m, 1H), 4.16 – 4.05 (m, 1H), 4.06 – 3.89 (m, 5H), 2.34 (dd, *J* = 10.7, 4.0 Hz, 2H), 2.10 (dt, *J* = 20.7, 7.5 Hz, 1H), 1.90 – 1.74 (m, 1H), 1.64 (dd, *J* = 14.9, 7.3 Hz, 2H), 1.33 (t, *J* = 7.0 Hz, 3H), 1.21 (t, *J* = 7.0 Hz, 3H).

^{13}C NMR (101 MHz, $CDCl_3$) δ 173.01, 156.25, 154.04, 152.99, 133.54, 132.59, 131.28, 129.79, 129.68, 129.19, 127.00, 125.76, 123.18, 119.23, 113.24, 69.78, 64.73, 61.99, 43.01, 41.74, 32.46, 23.50, 16.91, 15.50, 14.50.

IR (thin film) ν 2980, 2934, 1778, 1697, 1387 cm^{-1}.

HRMS (ESI) $[M+Na]^+$ calcd for $C_{25}H_{26}Cl_2N_2O_5SNa$: 559.0837, found 559.0837.

3-(2-cyclohexyl-2-(2-methoxy-5-methyl-3-(methylthio)phenyl)acetyl)oxazolidin-2-one **18d**

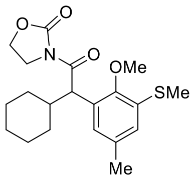

According to general procedure isolated in 25% yield as a transparent oil.

^{1}H NMR (400 MHz, $CDCl_3$) δ 7.18 (d, *J* = 1.5 Hz, 1H), 6.85 (d, *J* = 1.6 Hz, 1H), 5.43 (d, *J* = 10.5 Hz, 1H), 4.37 (td, *J* = 9.1, 5.4 Hz, 1H), 4.29 (dd, *J* = 17.3, 8.8 Hz, 1H), 4.04 (ddd, *J* = 10.9, 9.4, 8.3 Hz, 1H), 3.92 (ddd, *J* = 11.1, 9.1, 5.4 Hz, 1H), 3.81 (s, 3H), 2.41 (s, 3H), 2.33 (s, 3H), 2.13 – 2.02 (m, 1H), 1.78 – 1.71 (m, 4H), 1.49 (d, *J* = 13.3 Hz, 1H), 1.35 – 1.23 (m, 2H), 1.22 – 1.10 (m, 2H), 1.01 – 0.91 (m, 1H).

^{13}C NMR (101 MHz, $CDCl_3$) δ 174.98, 153.62, 153.11, 134.18, 131.89, 130.55, 126.51, 125.57, 61.43, 61.00, 46.29, 43.02, 41.97, 31.11, 30.71, 26.25 (2C), 26.19, 21.31, 14.83.

IR (thin film) ν 2925, 1779, 1693, 1382, 1245, 1103 cm^{-1}.

HRMS (ESI) $[M+Na]^+$ calcd for $C_{20}H_{27}NO_4SNa$: 400.1553, found 400.01555.

3-(2-cyclohexyl-2-(2-(dimethylamino)-3-(methylthio)phenyl)acetyl)oxazolidin-2-one **18e** and 3-(2-cyclohexyl-2-(4-(dimethylamino)-3-(methylthio)phenyl)acetyl)oxazolidin-2-one **18e'**

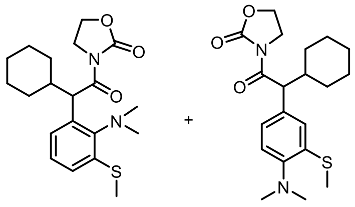

According to general procedure using 120 mol% of Tf_2NH. Isolated in 28% yield as a mixture of:**18e'** 1:1.3. **19e** was not observed.

¹H NMR (400 MHz, $CDCl_3$) δ 7.41 (dd, *J* = 7.8, 1.0 Hz, 1.0H), 7.19 (t, *J* = 7.8 Hz, 1.0H), 7.16 – 7.09 (m, 2.7H), 7.03 (d, *J* = 7.8, 0.9 Hz, 1.0H), 6.98 (d, *J* = 8.0 Hz, 1.3H), 5.57 (d, *J* = 10.4 Hz, 1.0H), 4.80 (d, *J* = 10.7 Hz, 1.3H), 4.42 – 4.34 (m, 2.3H), 4.33 – 4.25 (m, 2.3H), 4.09 – 4.00 (m, 2.3H), 3.98 – 3.90 (m, 1.3H), 3.89 – 3.83 (m, 1.0H), 2.84 (s, 3H), 2.73 (s, 10.8H), 2.44 (s, 3.9H), 2.40 (s, 3H), 2.19 – 2.03 (m, 2.7H), 1.84 – 1.59 (m, 11.5H), 1.38 – 1.23 (m, 6.0H), 1.20 – 1.10 (m, 4.7H), 0.85 – 0.75 (m, 1.0H).

18e: ¹³C NMR (151 MHz, $CDCl_3$) δ 175.57, 153.09, 148.27, 140.61, 139.05, 126.45, 125.91, 124.16, 61.46, 47.82, 43.13, 42.38, 41.98, 41.65, 31.17, 30.79, 26.40, 26.37, 26.16, 15.36;

18e': ¹³C NMR (151 MHz, $CDCl_3$) δ 174.28, 153.19, 149.97, 134.05, 132.80, 125.61, 125.38, 118.89, 61.50, 53.98, 44.30 (2C), 42.23, 41.18, 31.99, 30.21, 26.31, 26.15, 26.06, 14.88.

IR (thin film) ν 2924, 1776, 1694, 1384, 1199, 705 cm^{-1}.

HRMS (ESI) $[M+H]^+$ calcd for $C_{20}H_{29}N_2O_3S$: 377.1893, found 377.1892.

3-(2-cyclohexyl-2-(2-methoxy-3-(methylthio)phenyl)acetyl)oxazolidin-2-one **18f**

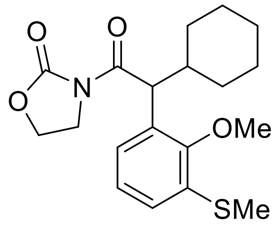

According to general procedure isolated in 19% yield as a transparent oil. Regioisomers separated by preparative LC/MS X-Bridge column (MeCN/H_2O).

¹H NMR (700 MHz, $CDCl_3$) δ 7.42 (dd, *J* = 7.8, 1.3 Hz, 1H), 7.15 (t, *J* = 7.8 Hz, 1H), 7.08 (dd, *J* = 7.8, 1.3 Hz, 1H), 5.50 (d, *J* = 10.5 Hz, 1H), 4.47 – 4.37 (m, 1H), 4.32 (q, *J* = 8.8 Hz, 1H), 4.06 (dt, *J* = 10.9, 9.3 Hz, 1H), 3.95 (ddd, *J* = 11.1, 9.2, 5.2 Hz, 1H), 3.87 (s, 3H), 2.45 (s, 3H), 2.12 (dt, *J* = 11.1, 2.9 Hz, 1H), 1.80 – 1.63 (m, 4H), 1.53 – 1.47 (m, 1H), 1.41 – 1.26 (m, 2H), 1.25 – 1.13 (m, 2H), 1.01 – 0.97 (m, 1H).

¹³C NMR (176 MHz, $CDCl_3$) δ 174.91, 155.61, 153.14, 132.55, 131.09, 126.05, 124.82, 124.72, 61.46, 60.94, 46.24, 43.02, 42.00, 31.08, 30.75, 26.25, 26.18, 14.73.

IR (thin film) ν 2923, 2850, 1774, 1692, 1198 cm^{-1}.

HRMS (ESI) $[M+Na]^+$ calcd for $C_{19}H_{25}NO_4SNa$: 386.1403, found 386.1404.

3-(2-cyclohexyl-2-(3-methoxy-2-(methylthio)phenyl)acetyl)oxazolidin-2-one **19f**

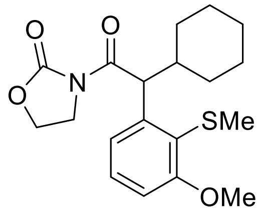

According to general procedure isolated in 28% yield as a transparent oil. Regioisomers separated by preparative LC/MS X-Bridge column (MeCN/H_2O).

^{1}H NMR (700 MHz, $CDCl_3$) δ 7.30 – 7.25 (m, 1H), 7.15 (dd, *J* = 7.9, 1.0 Hz, 1H), 6.82 (dd, *J* = 8.1, 0.7 Hz, 1H), 5.87 (d, *J* = 10.0 Hz, 1H), 4.43 – 4.31 (m, 1H), 4.29 (dd, *J* = 16.8, 8.9 Hz, 1H), 4.08 (ddd, *J* = 10.9, 9.4, 7.8 Hz, 1H), 3.98 – 3.91 (m, 4H), 2.35 (s, 3H), 2.11 – 2.04 (m, 1H), 1.80 – 1.71 (m, 2H), 1.67 – 1.61 (m, 2H), 1.37 – 1.23 (m, 3H), 1.23 – 1.06 (m, 3H).

^{13}C NMR (176 MHz, $CDCl_3$) δ 174.66, 160.18, 152.62, 141.96, 128.92, 126.19, 120.32, 109.52, 61.57, 55.92, 51.63, 43.16, 43.06, 31.62, 29.98, 26.53, 26.40, 26.29, 18.22.

IR (thin film) ν 2924, 2851, 1777, 1689, 1258, 1101 cm^{-1}.

HRMS (ESI) $[M+Na]^+$ calcd for $C_{19}H_{25}NO_4SNa$: 386.1403, found 386.1402.

(R)-3-(2-cyclohexyl-2-(3-(cyclohexylthio)-2-methoxyphenyl)acetyl)oxazolidin-2-one **18g**

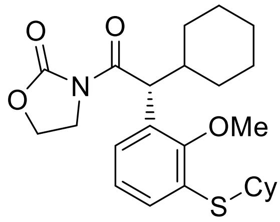

Prepared according to general procedure in 14% yield from enantipure sulfoxide **17g**.

Racemic product prepared according to general procedure in 14% yield from the racemic sulfoxide **17g**.

^{1}H NMR (600 MHz, $CDCl_3$) δ 7.44 (dd, *J* = 7.8, 1.5 Hz, 1H), 7.23 (dd, *J* = 7.8, 1.5 Hz, 1H), 7.08 (t, *J* = 7.8 Hz, 1H), 5.48 (d, *J* = 10.5 Hz, 1H), 4.40 (td, *J* = 9.2, 5.3 Hz, 1H), 4.32 (dd, *J* = 17.3, 8.8 Hz, 1H), 4.12 – 4.01 (m, 1H), 3.95 (ddd, *J* = 11.0, 9.2, 5.3 Hz, 1H), 3.88 (s, 3H), 3.32 – 3.16 (m, 1H), 2.15 – 2.09 (m, 1H), 2.05 – 1.90 (m, 2H), 1.88 – 1.61 (m, 7H), 1.52 – 1.25 (m, 8H), 1.24 – 1.10 (m, 2H), 1.04 – 0.94 (m, 1H).

^{13}C NMR (151 MHz, $CDCl_3$) δ 174.98, 157.57, 153.10, 131.51, 129.80, 129.46, 127.31, 124.40, 61.46, 61.22, 46.54, 44.53, 43.03, 42.06, 33.28, 33.22, 31.18, 30.60, 26.26, 26.24, 26.19, 26.06, 26.01, 25.83.

IR (thin film) ν 2926, 2851, 1778, 1690, 1579, 1474, 1268, 1241 cm^{-1}.

HRMS (ESI) $[M+Na]^+$ calcd for $C_{24}H_{33}NO_4SNa$: 454.2028, found 454.2019.

$[\alpha]_D^{20}$ = + 11.5 (c = 1.00, $CHCl_3$).

Enantiomeric ration 98.5:1.5 was determined by chiral HPLC analysis: Chiralpak IC, *n*-heptane/IPA 92:8, 1 mL/min, 25 °C, detection at 254 nm, retention time (min): 8.3 (minor) and 10.8 (major).

(R)-3-(2-cyclohexyl-2-(2-(cyclohexylthio)-3-methoxyphenyl)acetyl)oxazolidin-2-one **19g**

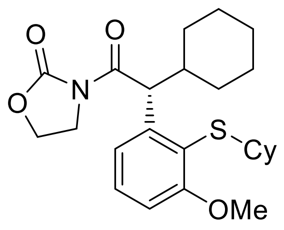

Prepared according to general procedure in 37% yield from enantipure sulfoxide **17d**.

Racemic product prepared according to general procedure in 33% yield from the racemic sulfoxide **17d**.

^{1}H NMR (600 MHz, $CDCl_3$) δ 7.33 (s, 1H), 7.33 (q, *J* = 8.0 Hz, 1H), 6.82 (dd, *J* = 6.6, 2.8 Hz, 1H), 5.98 (d, *J* = 10.3 Hz, 1H), 4.40 (td, *J* = 9.1, 4.7 Hz, 1H), 4.29 (q, *J* = 8.8 Hz, 1H), 4.05 (dt, *J* = 11.0, 9.1 Hz, 1H), 3.93 (ddd, *J* = 11.0, 8.9, 4.7 Hz, 1H), 3.89 (s, 3H), 3.17 (ddd, *J* = 10.9, 7.2, 3.7 Hz, 1H), 2.24 – 2.09 (m, 1H), 1.88 – 1.62 (m, 8H), 1.55 – 1.44 (m, 2H), 1.42 – 1.11 (m, 8H), 0.95 – 0.83 (m, 2H).

^{13}C NMR (151 MHz, $CDCl_3$) δ 175.39, 160.33, 152.74, 143.60, 128.92, 123.47, 121.48, 109.24, 61.58, 55.87, 50.74, 46.59, 43.22, 42.99, 33.17, 33.10, 30.67, 30.54, 26.43, 26.41, 26.23, 26.13, 25.82.

IR (thin film) ν 2851, 1778, 1690, 1580, 1475, 1241 cm^{-1}.

HRMS (ESI) $[M+Na]^+$ calcd for $C_{24}H_{33}NO_4SNa$: 454.2028, found 454.2025.

$[\alpha]_D^{20}$ = + 20.7 (c = 1.00, $CHCl_3$).

Enantiomeric ration 92:8 was determined by chiral HPLC analysis: Chiralpak IC, *n*-heptane/IPA 92:8, 1 mL/min, 25 °C, detection at 254 nm, retention time (min): 14.4 (major) and 16.4 (minor).

5-(2-methoxy-3-(methylthio)phenyl)-6-oxo-6-(2-oxooxazolidin-3-yl)hexanenitrile **18h**

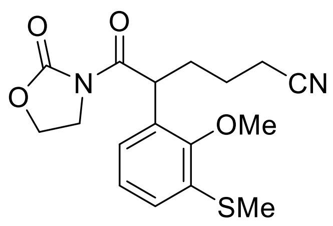

According to general procedure isolated in 23% yield as a transparent oil. Regioisomers separated by reverse phase preparative LC/MS X-Bridge column ($MeCN/H_2O$).

^{1}H NMR (600 MHz, $CDCl_3$) δ 7.22 (dd, *J* = 8.5, 2.3 Hz, 1H), 7.14 (d, *J* = 2.3 Hz, 1H), 6.86 (d, *J* = 8.6 Hz, 1H), 5.30 (t, *J* = 7.2 Hz, 1H), 4.43 (td, *J* = 9.1, 6.2 Hz, 1H), 4.36 (dt, *J* = 16.5, 8.3 Hz, 1H), 4.18 – 4.06 (m, 1H), 4.02 (ddd, *J* = 11.0, 9.3, 6.2 Hz, 1H), 3.87 (s, 1H), 2.46 (s, 1H), 2.45 – 2.36 (m, 1H), 2.18 – 2.06 (m, 1H), 1.97 – 1.85 (m, 1H), 1.78 – 1.65 (m, 1H).

^{13}C NMR (151 MHz, $CDCl_3$) δ 173.52, 155.73, 152.75, 129.04, 128.58, 127.99, 127.34, 119.57, 111.76, 61.87, 56.02, 42.94, 41.50, 31.63, 23.32, 17.77, 16.97.

IR (thin film) ν 2927, 2839, 2246, 1776, 1691, 1572, 1262 cm^{-1}.

HRMS (ESI) $[M+Na]^+$ calcd for $C_{17}H_{20}N_2O_4SNa$: 371.1042, found 371.1039.

5-(3-methoxy-2-(methylthio)phenyl)-6-oxo-6-(2-oxooxazolidin-3-yl)hexanenitrile **19h**

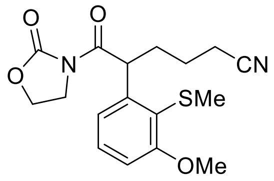

According to general procedure isolated in 34% yield as a transparent oil. Regioisomers separated by reverse phase preparative LC/MS X-Bridge column (MeCN/H_2O).

^{1}H NMR (600 MHz, $CDCl_3$) δ 7.27 (t, *J* = 8.0 Hz, 1H), 6.85 (dd, *J* = 8.3, 0.8 Hz, 1H), 6.79 (dd, *J* = 7.8, 1.0 Hz, 1H), 5.75 (dd, *J* = 8.4, 5.0 Hz, 1H), 4.43 (td, *J* = 9.2, 6.3 Hz, 1H), 4.35 (td, *J* = 9.1, 7.4 Hz, 1H), 4.13 (ddd, *J* = 11.0, 9.4, 7.3 Hz, 1H), 4.02 (ddd, *J* = 11.0, 9.3, 6.3 Hz, 1H), 3.94 (s, 1H), 2.44 – 2.41 (m, 1H), 2.41 (s, 1H), 2.18 (dd, *J* = 13.7, 5.4 Hz, 1H), 1.88 – 1.71 (m, 1H).

^{13}C NMR (151 MHz, $CDCl_3$) δ 173.54, 160.56, 152.55, 142.79, 129.34, 124.54, 119.58, 118.69, 110.05, 61.91, 55.98, 47.03, 42.97, 32.84, 23.69, 17.87, 17.11.

IR (thin film) ν 2924, 2840, 2245, 1777, 1694, 1246 cm^{-1}.

HRMS (ESI) $[M+Na]^+$ calcd for $C_{17}H_{20}N_2O_4SNa$: 371.1042, found 371.1035.

3-(1-cyclohexyl-2-oxo-2-(2-oxooxazolidin-3-yl)ethyl)-2-(methylthio)phenyl acetate **19i**

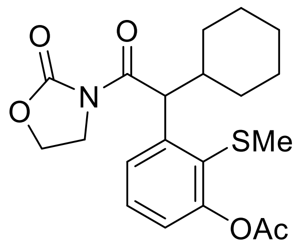

According to general procedure isolated in 32% yield as a white solid.

^{1}H NMR (600 MHz, $CDCl_3$) δ 7.54 (dd, *J* = 8.0, 1.2 Hz, 1H), 7.35 (t, *J* = 8.0 Hz, 1H), 7.03 (dd, *J* = 7.9, 1.3 Hz, 1H), 5.89 (d, *J* = 10.2 Hz, 1H), 4.40 (td, *J* = 9.1, 5.7 Hz, 1H), 4.32 (dd, *J* = 16.9, 8.9 Hz, 1H), 4.08 (ddd, *J* = 11.0, 9.4, 8.0 Hz, 1H), 3.96 (ddd, *J* = 11.1, 9.2, 5.7 Hz, 1H), 2.39 (s, 3H), 2.33 (s, 3H), 2.07 (td, *J* = 10.9, 3.0 Hz, 1H), 1.84 – 1.70 (m, 2H), 1.70 – 1.57 (m, 2H), 1.41 – 1.24 (m, 3H), 1.24 – 1.01 (m, 3H).

^{13}C NMR (151 MHz, $CDCl_3$) δ 174.52, 169.41, 152.96, 152.82, 142.91, 131.33, 129.20, 126.31, 121.73, 61.61, 51.12, 43.63, 43.13, 31.47, 30.08, 26.45, 26.32, 26.20, 21.12, 19.21.

IR (thin film) ν 2926, 2853, 1773, 1691, 1196 cm^{-1}.

HRMS (ESI) $[M+Na]^+$ calcd for $C_{20}H_{25}NO_5SNa$: 414.1351, found 414.1346.

Structure determined by the X-ray analysis (See section 7 in SI for the details).

3-(1-cyclohexyl-2-oxo-2-(2-oxooxazolidin-3-yl)ethyl)-2-(methylthio)phenyl trifluoromethanesulfonate **19j**

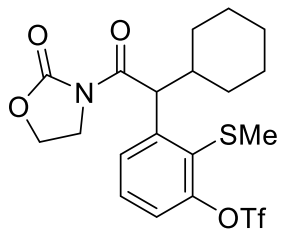

According to general procedure isolated in 33% yield as a transparent oil.

¹H NMR (600 MHz, $CDCl_3$) δ 7.67 (dd, *J* = 8.0, 1.1 Hz, 1H), 7.40 (t, *J* = 8.1 Hz, 1H), 7.23 (d, *J* = 8.0 Hz, 1H), 5.90 (d, *J* = 10.1 Hz, 1H), 4.43 (td, *J* = 9.1, 6.1 Hz, 1H), 4.36 (dt, *J* = 16.5, 8.3 Hz, 1H), 4.20 – 4.05 (m, 1H), 3.99 (ddd, *J* = 11.1, 9.2, 6.1 Hz, 1H), 2.41 (s, 3H), 2.13 – 1.94 (m, 1H), 1.79 – 1.75 (m, 2H), 1.74 – 1.62 (m, 2H), 1.42 – 1.03 (m, 6H).

¹³C NMR (151 MHz, $CDCl_3$) δ 174.04, 152.76, 152.05, 144.26, 132.97, 129.63, 128.45, 120.53, 118.63 (q, *J* = 320.3 Hz, $-CF_3$), 61.68, 51.29, 43.76, 43.08, 31.42, 30.00, 26.41, 26.28, 26.10, 19.12.

IR (thin film) ν 2990, 2854, 1782, 1693, 1210 cm^{-1}.

HRMS (ESI) $[M+Na]^+$ calcd for $C_{19}H_{22}NO_6S_2Na$: 504.0738, found 504.0734.

3-(2-cyclohexyl-2-(3-methyl-2-(methylthio)phenyl)acetyl)oxazolidin-2-one **19k**

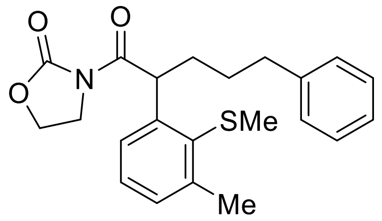

According to general procedure isolated in 40% yield as a transparent oil.

¹H NMR (400 MHz, $CDCl_3$) δ 7.20 – 7.14 (m, 2H), 7.14 – 7.05 (m, 5H), 6.98 (t, *J* = 4.7 Hz, 1H), 5.73 (dd, *J* = 8.1, 5.3 Hz, 1H), 4.40 – 4.14 (m, 2H), 4.04 – 3.84 (m, 2H), 2.69 – 2.55 (m, 2H), 2.50 (s, 3H), 2.16 (s, 3H), 2.11 – 1.96 (m, 1H), 1.73 – 1.54 (m, 3H).

¹³C NMR (101 MHz, $CDCl_3$) δ 174.54, 152.70, 143.57, 143.25, 142.17, 135.96, 129.43, 128.43, 128.38, 128.24, 125.69, 124.74, 61.75, 47.88, 43.01, 35.79, 33.74, 29.53, 21.69, 18.70.

IR (thin film) ν 2989, 2850, 1780, 1691 cm^{-1}.

HRMS (ESI) $[M+Na]^+$ calcd for $C_{22}H_{25}NO_3SNa$: 406.1453, found 406.1450.

3-(2-cyclohexyl-2-(3-methoxy-2-(methylthio)naphthalen-1-yl)acetyl)oxazolidin-2-one **19l**

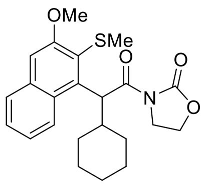

According to general procedure isolated in 34% yield as a transparent oil. Peak broadening was observed due to restricted rotation.

^{1}H NMR (600 MHz, $CDCl_3$) δ 8.10 (d, *J* = 8.7 Hz, 1H), 7.69 (d, *J* = 8.1 Hz, 1H), 7.39 (t, *J* = 7.4 Hz, 1H), 7.30 (s br, 1H), 7.08 (s, 1H), 5.92 (s br, 1H), 4.21 (s br, 1H), 4.08 – 3.90 (m br, 5H), 3.76 (s br, 1H), 2.70 – 2.59 (m br, 1H), 2.47 (s br, 2H), 2.09 (s br, 1H), 1.76 (d, *J* = 11.8 Hz, 1H), 1.63 (d, *J* = 12.7 Hz, 1H), 1.59 – 1.50 (m, 1H), 1.45 – 1.32 (m, 2H), 1.22 – 1.13 (m, 1H), 1.12 – 1.04 (m, 2H), 0.98 – 0.91 (m br, 1H).

^{13}C NMR (151 MHz, $CDCl_3$) δ 172.78, 156.77, 152.00, 134.17, 127.43, 126.14, 124.40, 124.02, 105.83, 61.86, 61.76, 55.79, 43.69, 39.94, 33.16, 30.09, 26.98, 26.68, 26.35, 18.51; 3 carbon peaks were not observed due to peak broadening.

IR (thin film) ν 2925, 1782, 1692, 1384, 1198, 1077, 732 cm^{-1}.

HRMS (ESI) $[M+Na]^+$ calcd for $C_{23}H_{27}NO_4SNa$: 436.1553, found 436.1552.

5.NMR Spectra

Molecule 1a (400 MHz, $CDCl_3$)

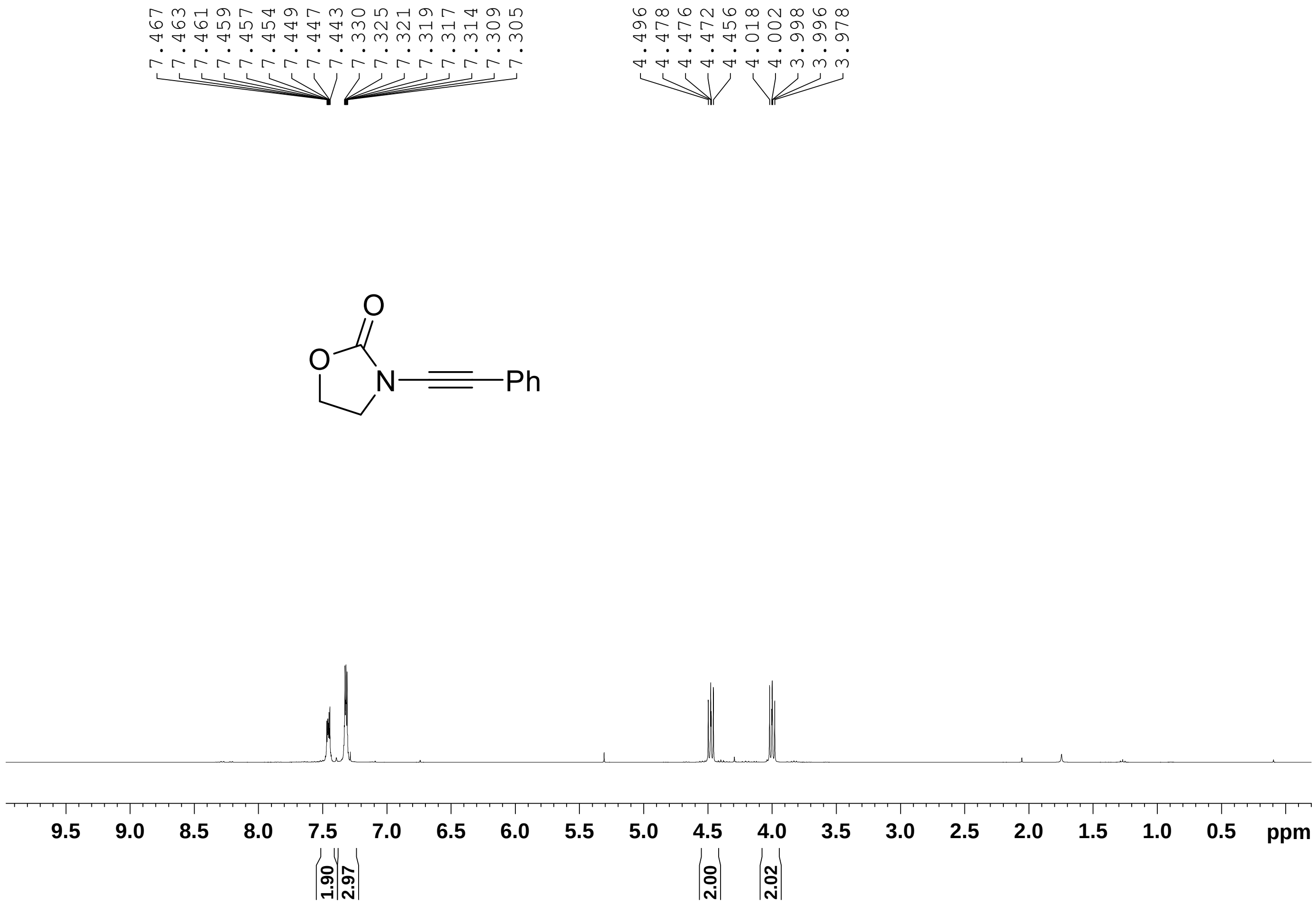

Molecule 1a (100 MHz, $CDCl_3$)

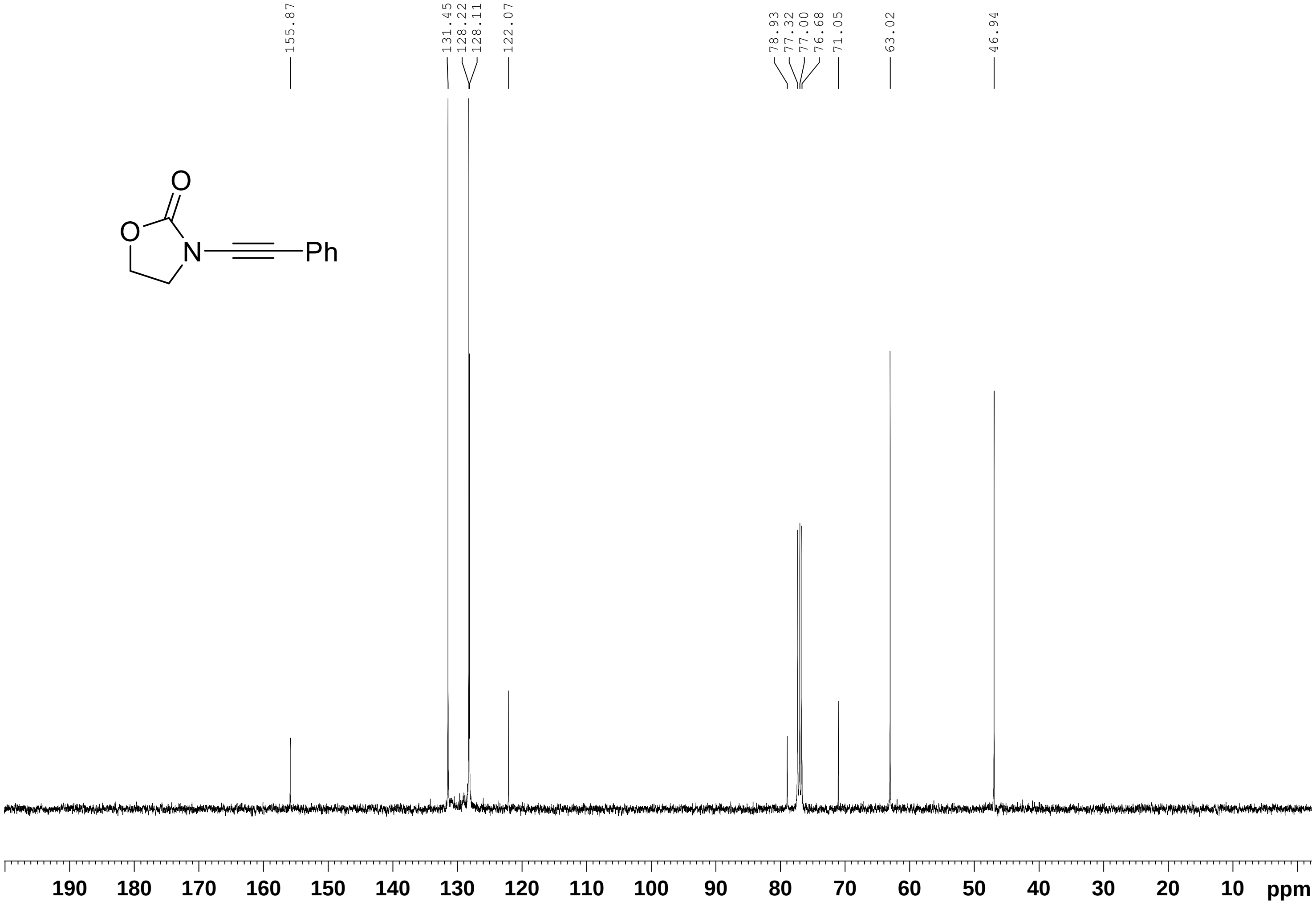

Molecule 1b (500 MHz, $CDCl_3$)

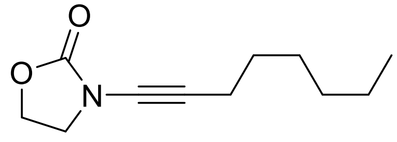

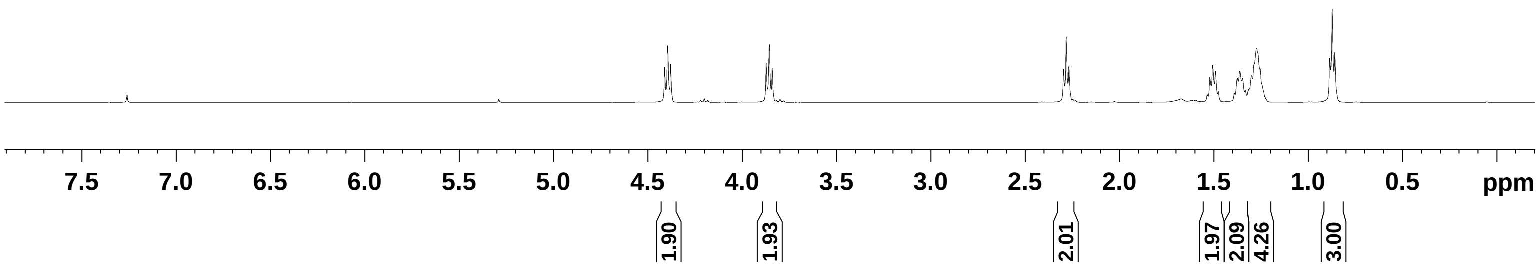

Molecule 1b (125 MHz, $CDCl_3$)

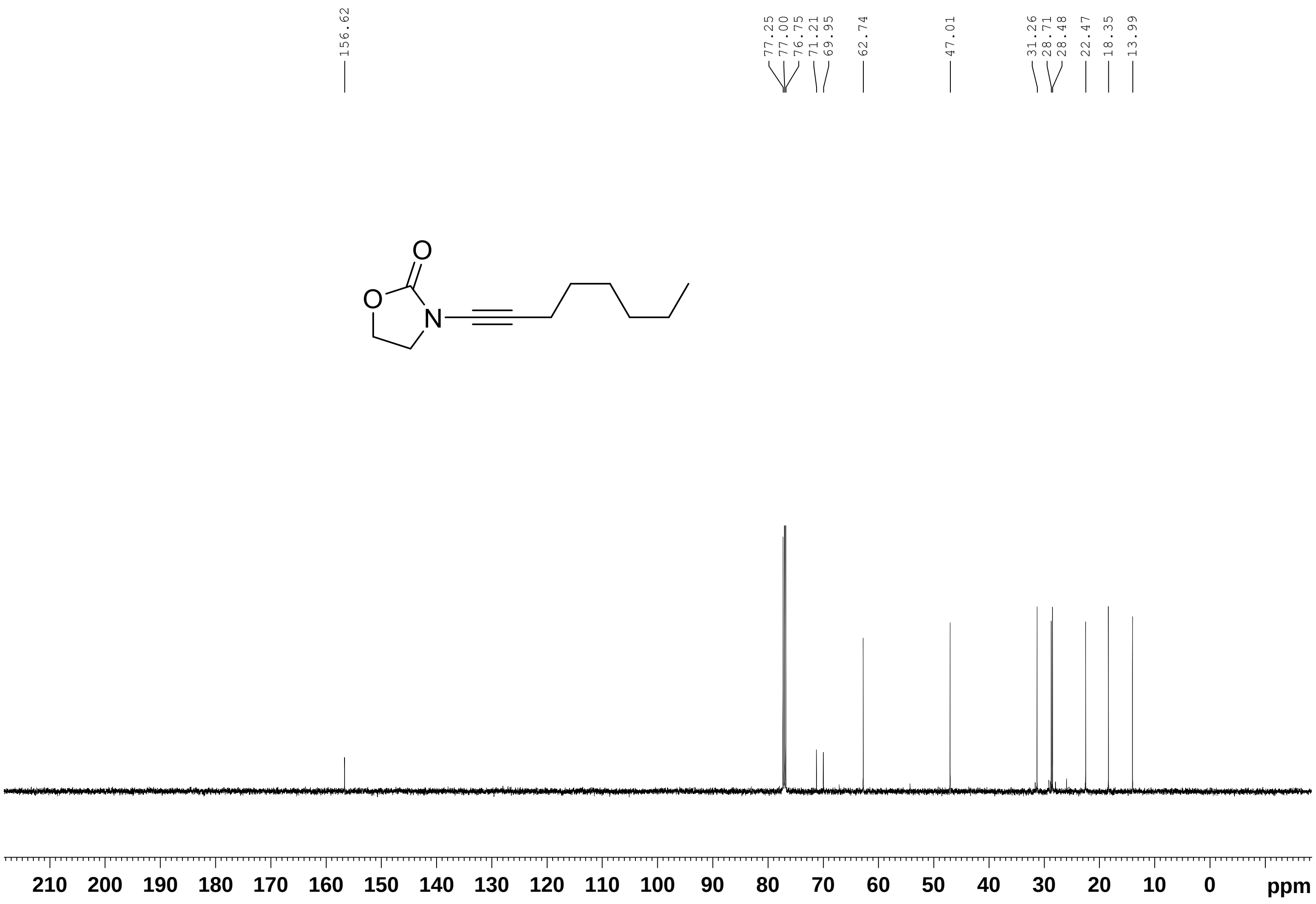

Molecule 7a (500MHz, $CDCl_3$)

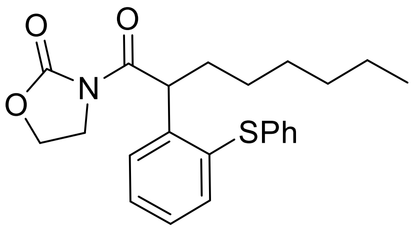

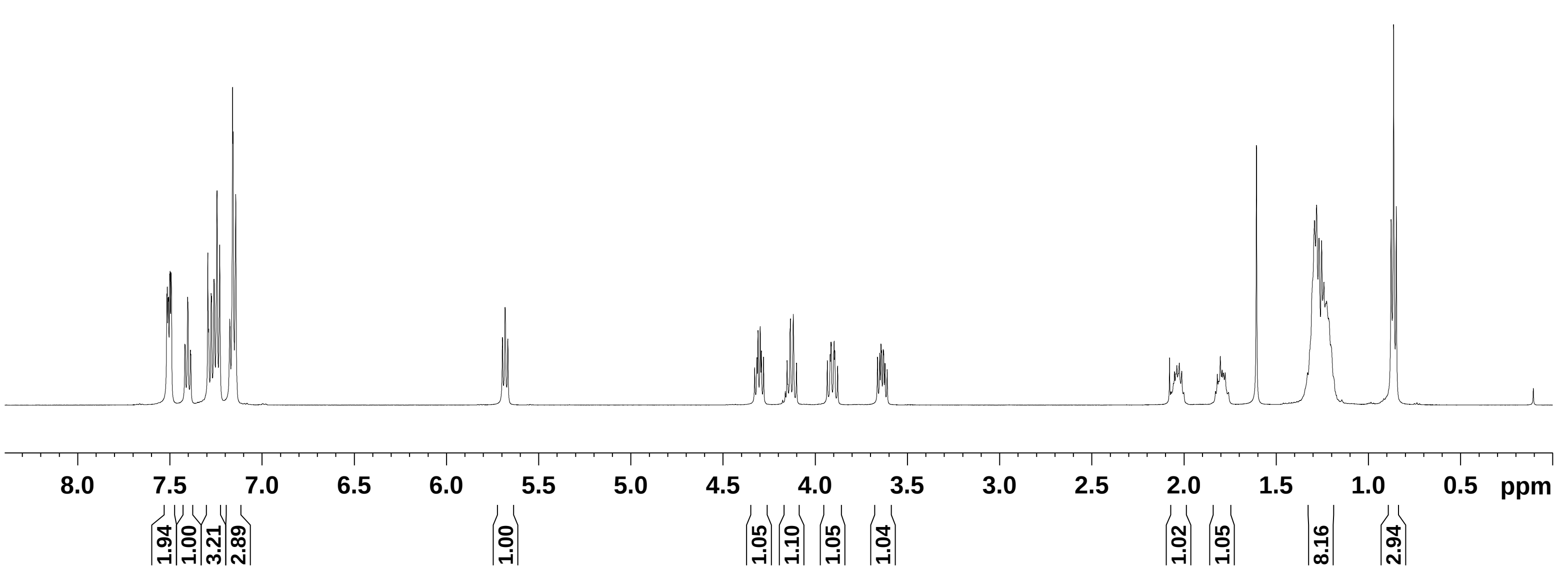

Molecule 7a (125MHz, $CDCl_3$)

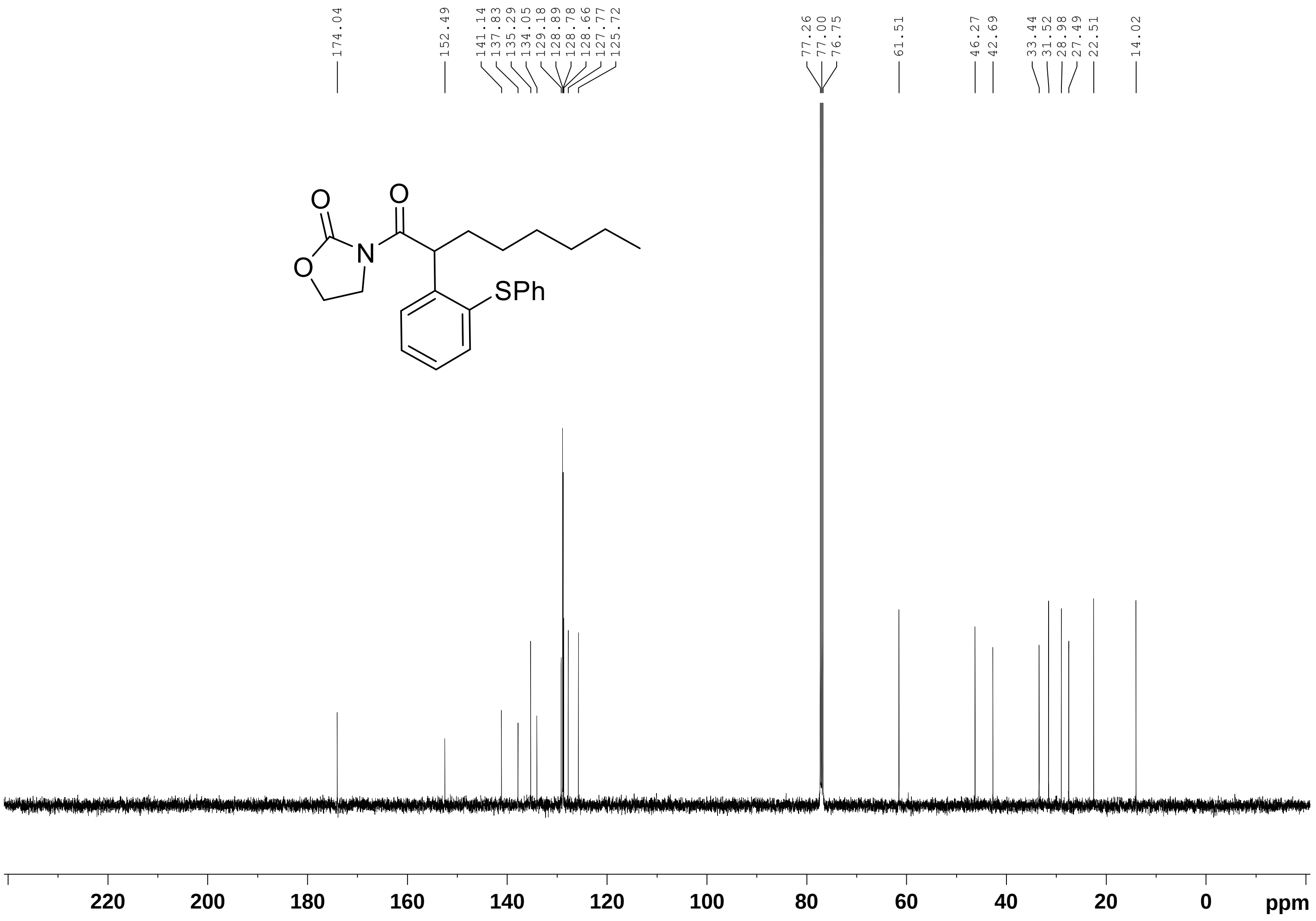

Molecule 7b (400MHz, $CDCl_3$)

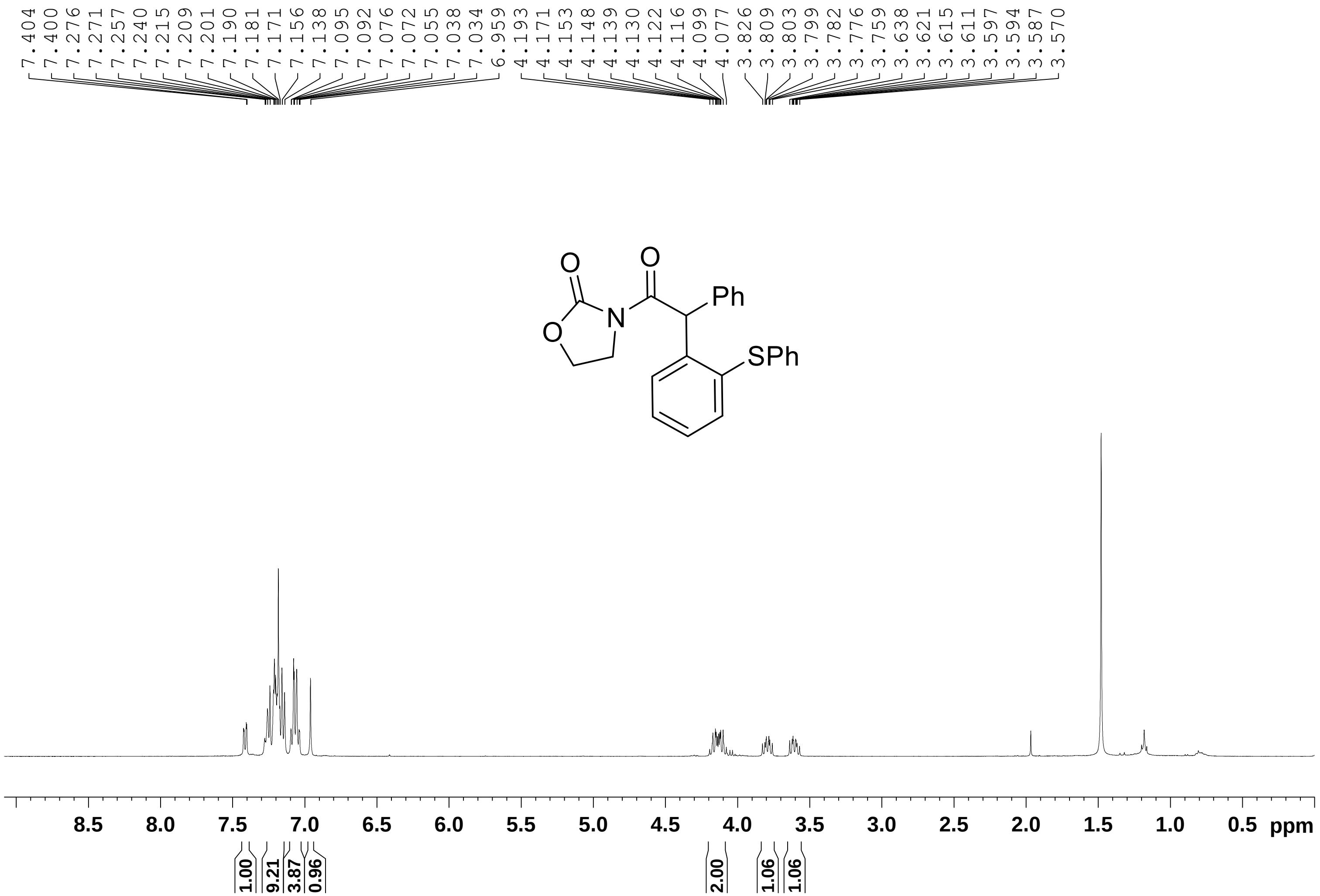

Molecule 7b (100MHz, $CDCl_3$)

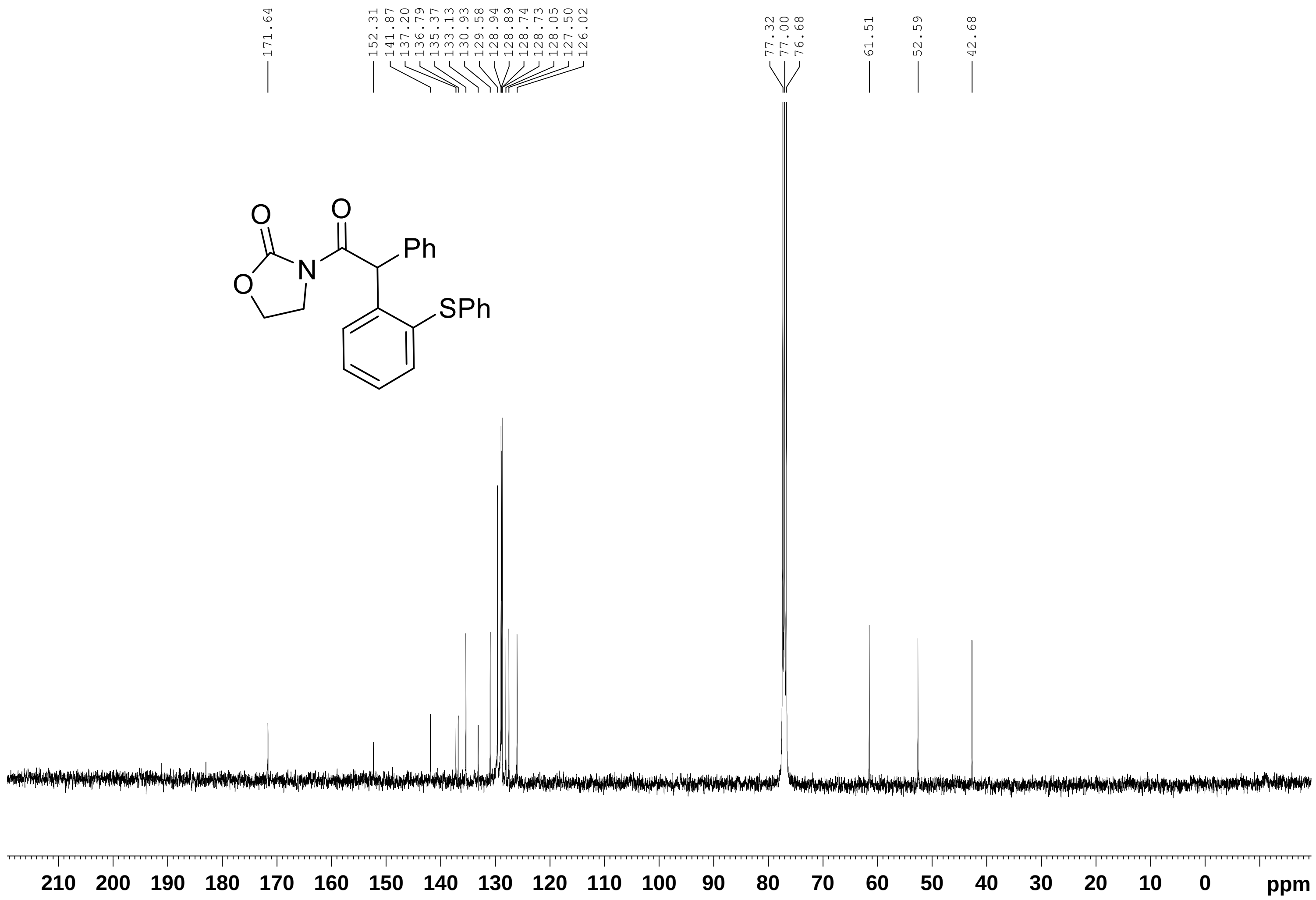

Molecule 7c (400MHz, $CDCl_3$)

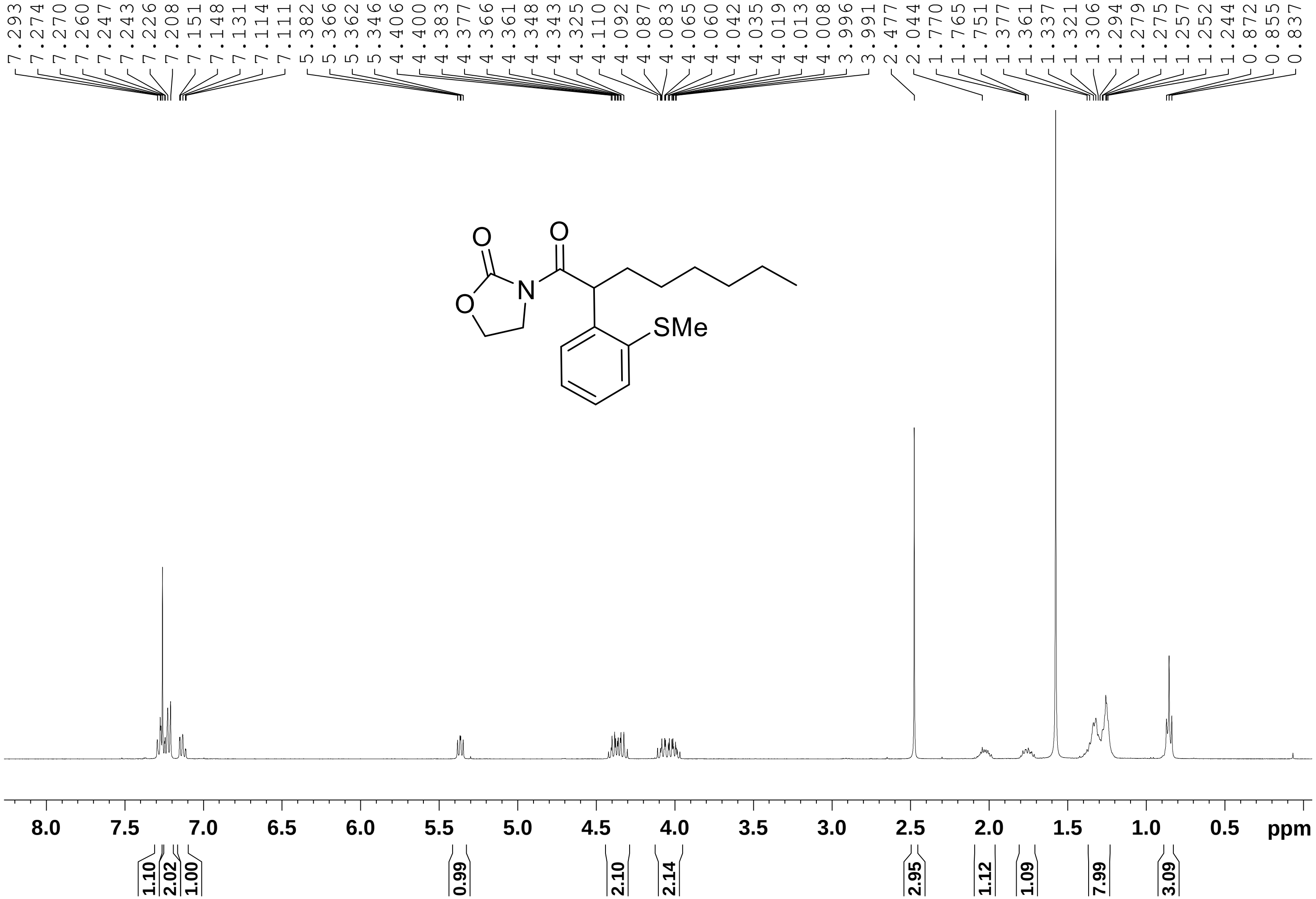

Molecule 7c (100MHz, $CDCl_3$)

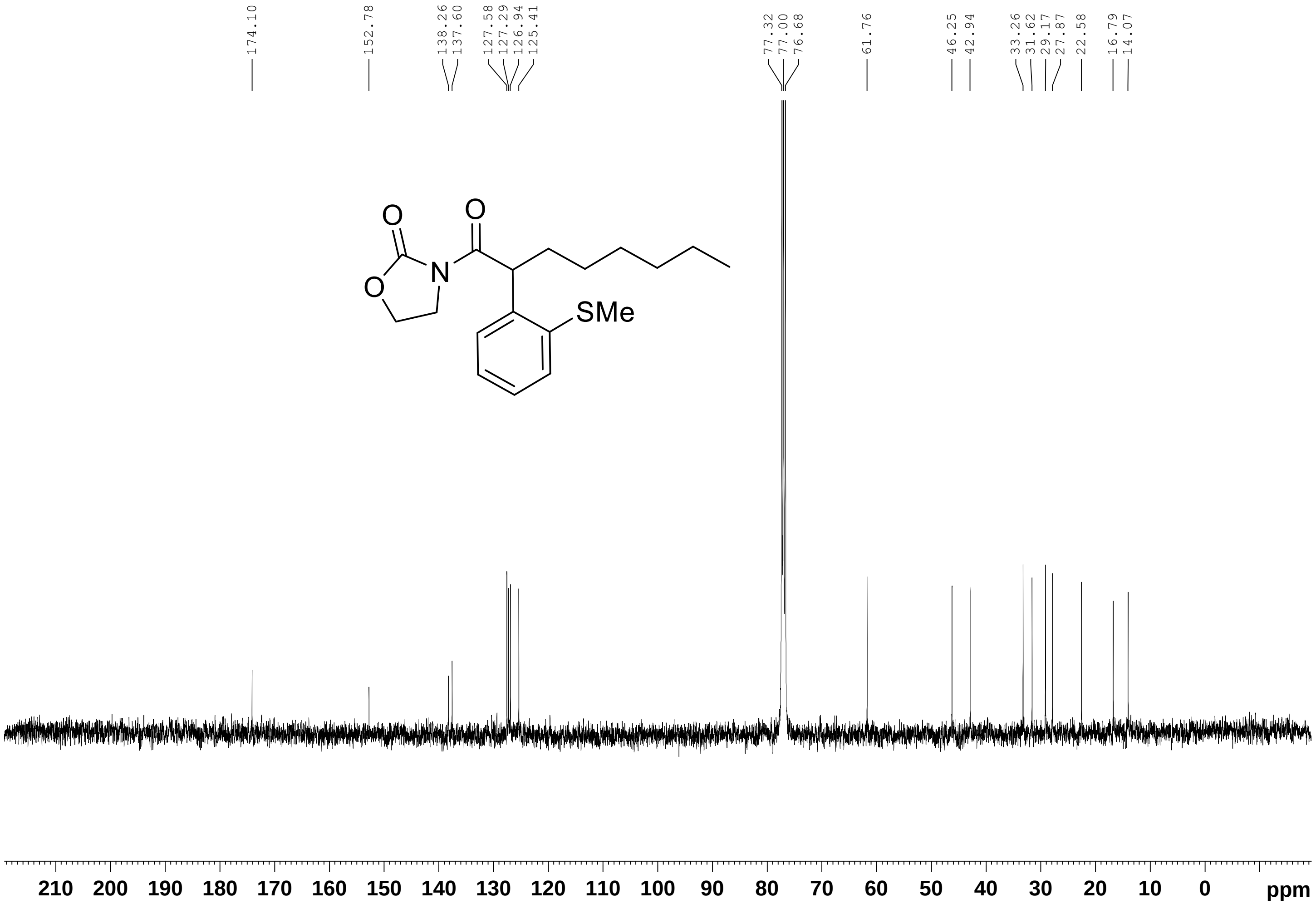

Compound **11**: ^{1}H NMR

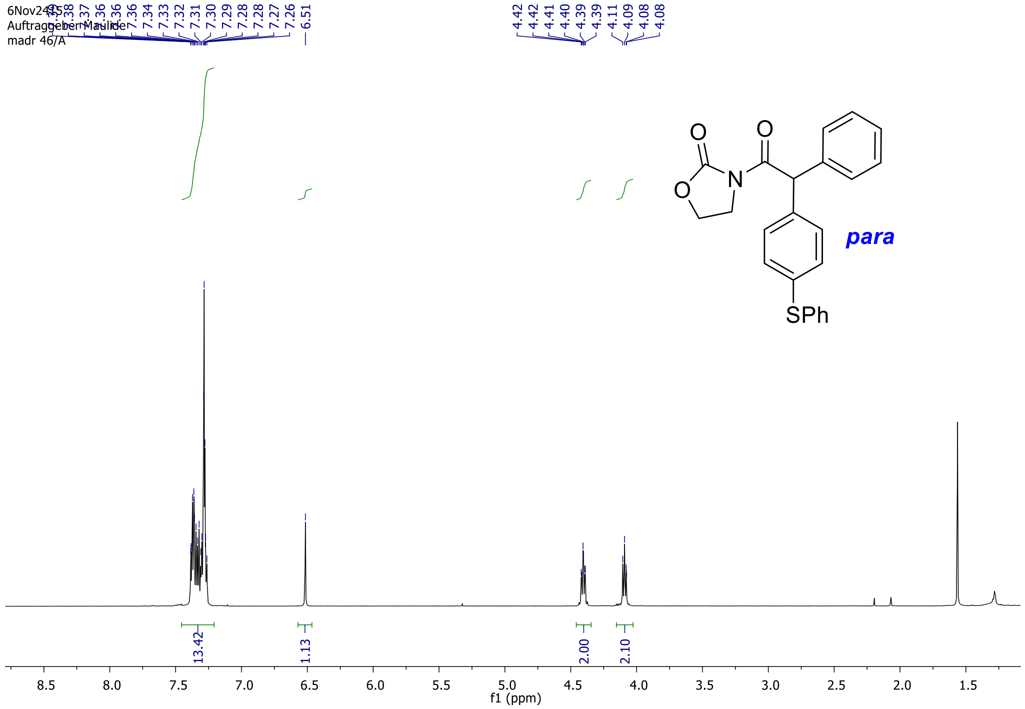

Compound **11**: ^{13}C NMR

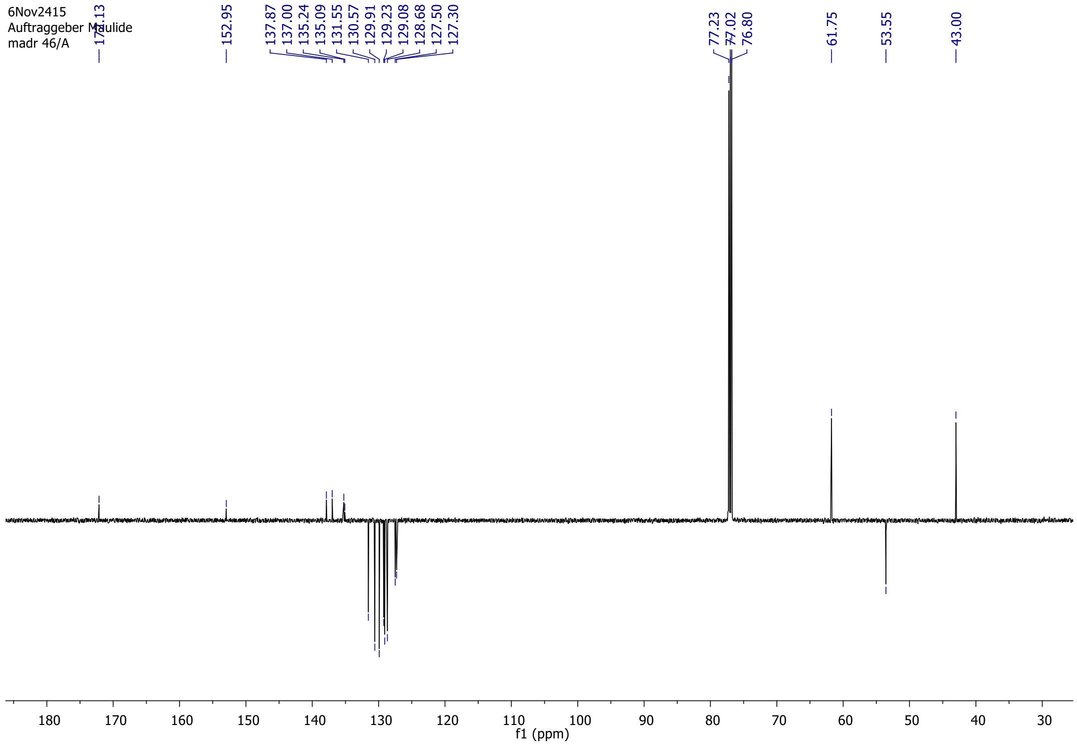

Compound **14**: ^{1}H NMR

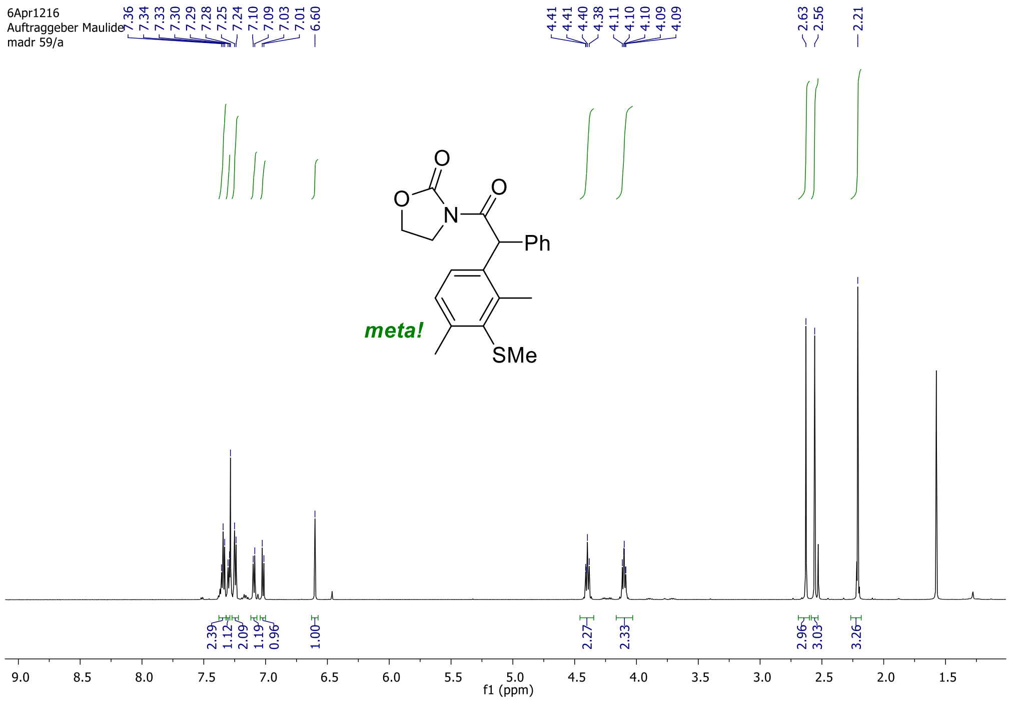

Compound **14**: ^{13}C NMR

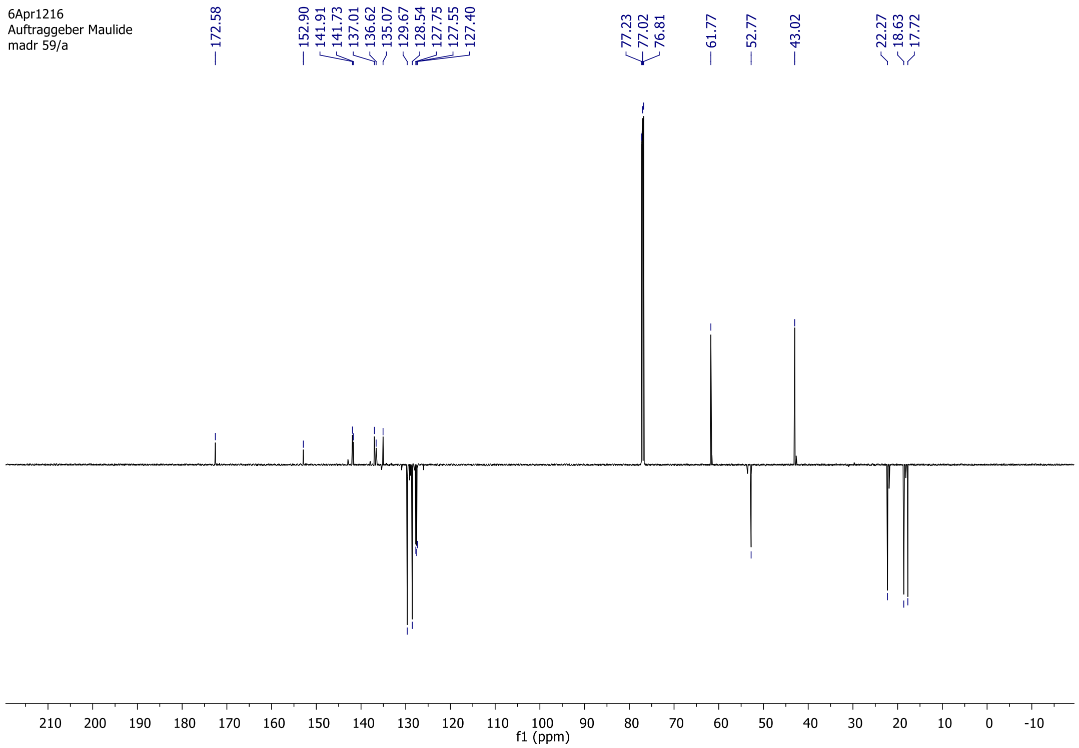

Compound **14**: COSY

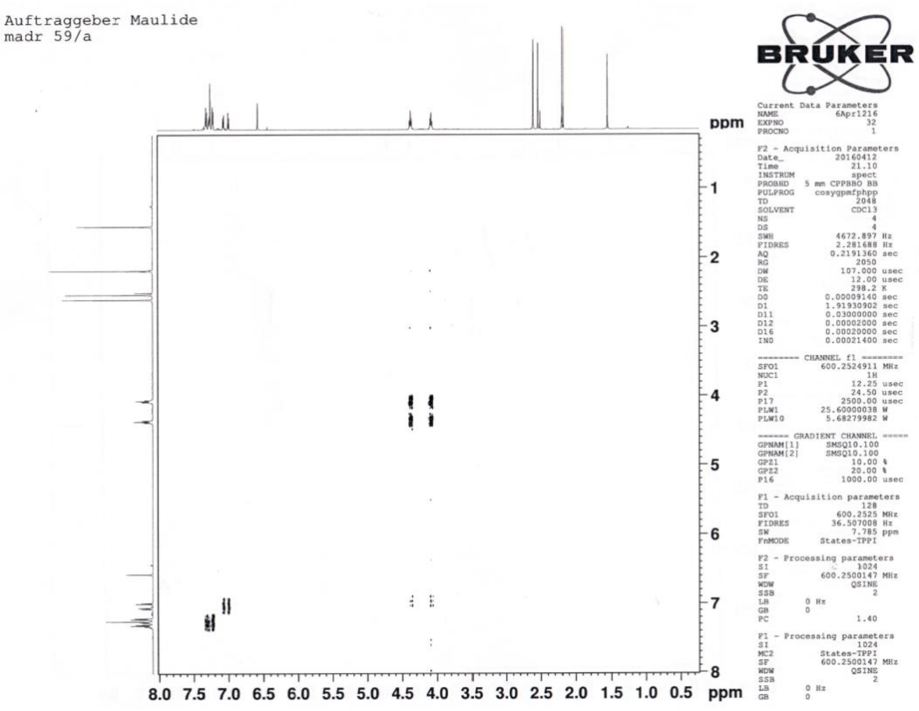

Compound **14**: HSQC

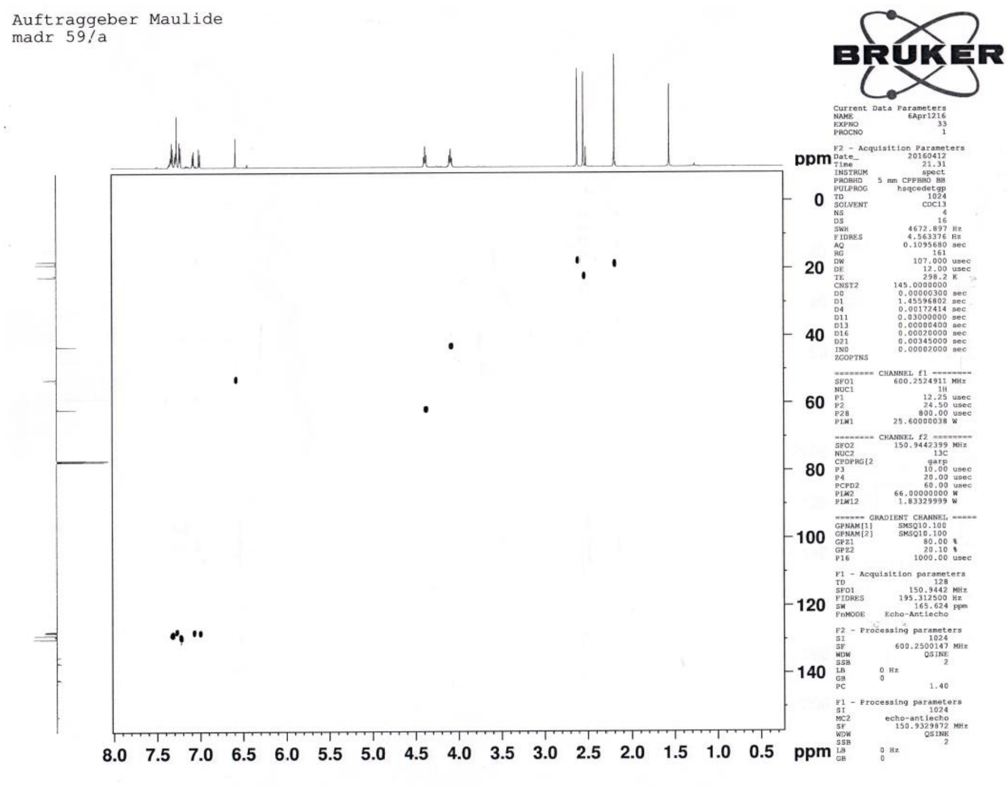

Compound **15**: ^{1}H NMR

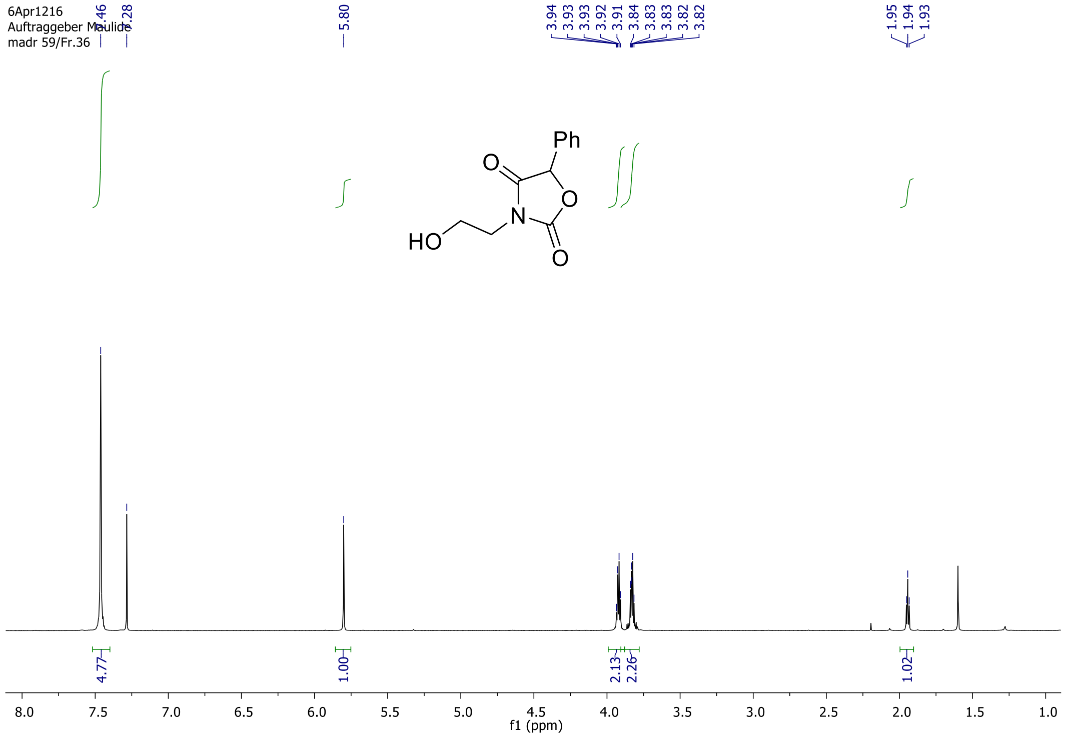

Compound **15**: ^{13}C NMR

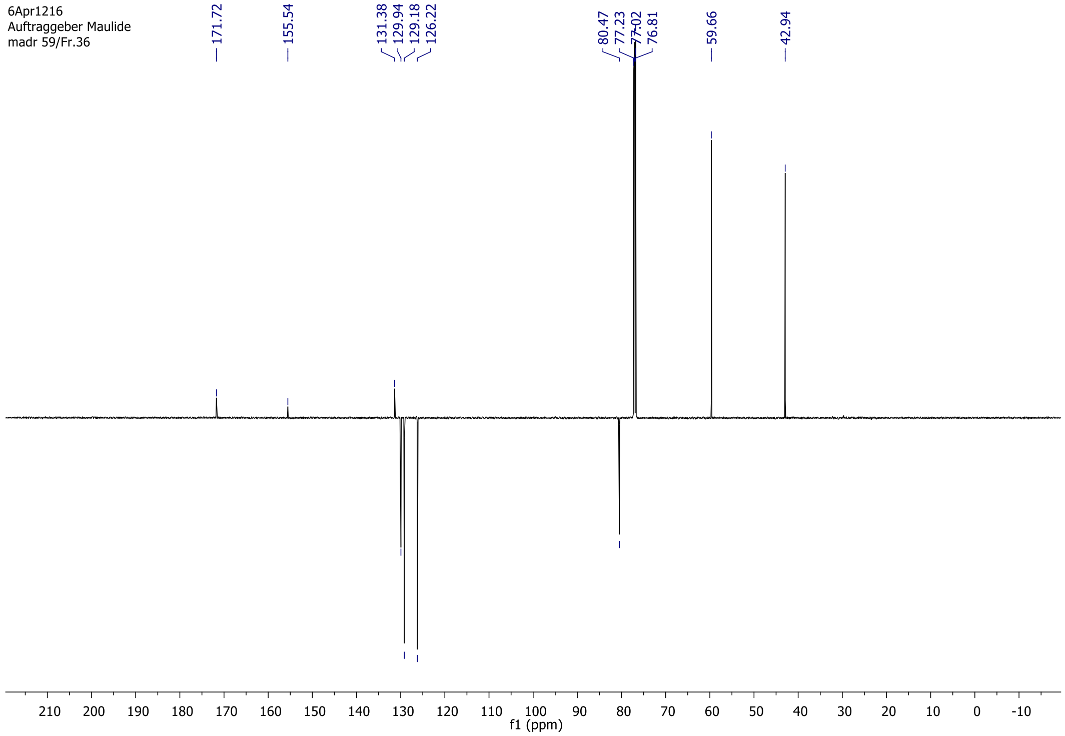

Compound **14b**: ^{1}H NMR

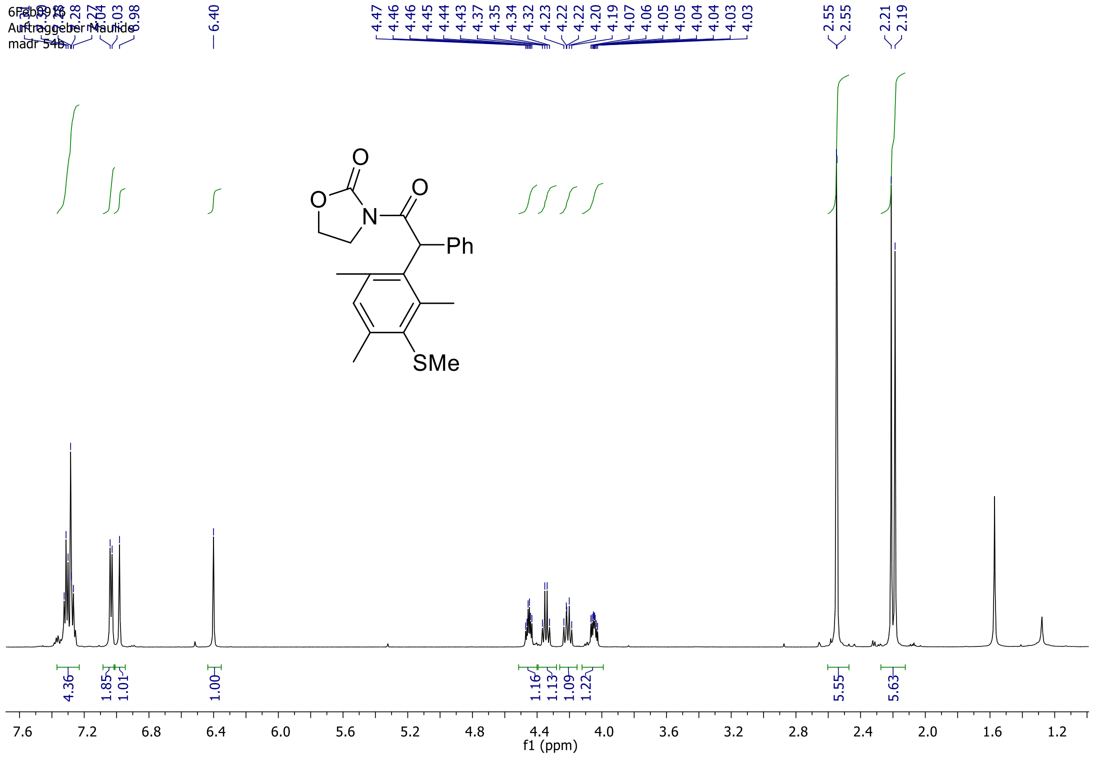

Compound **14b**: ^{13}C NMR

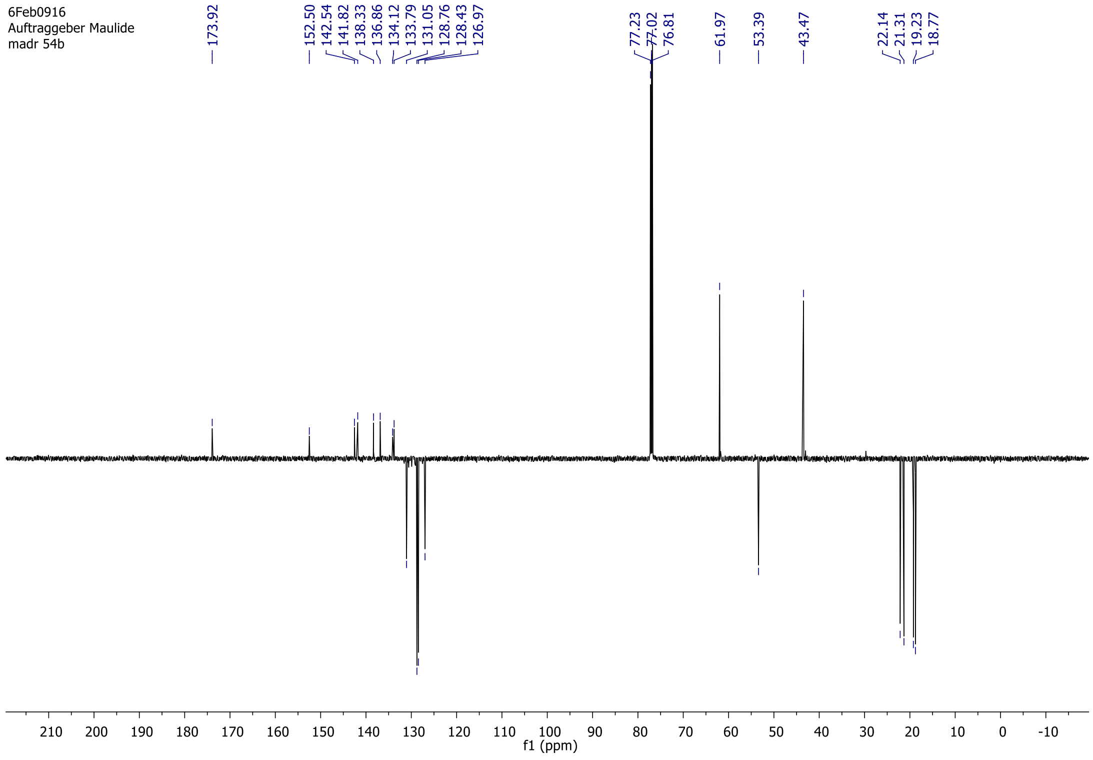

Compound **16**: ^{1}H NMR

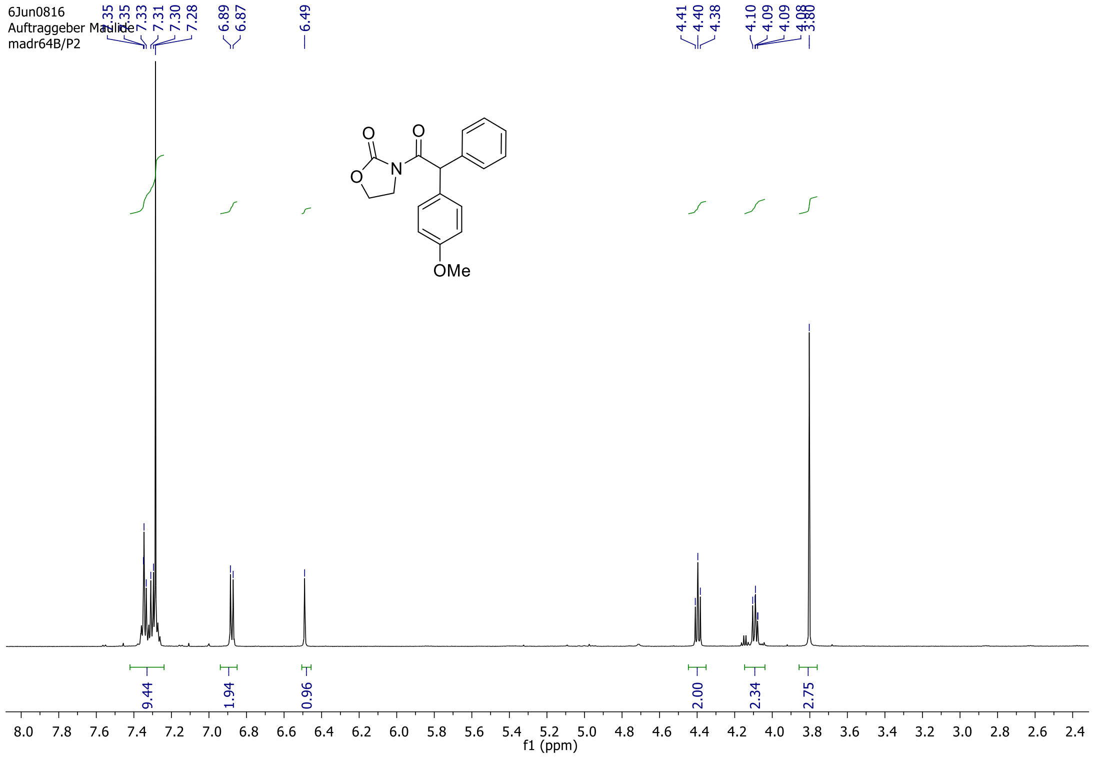

Compound **16**: ^{13}C NMR

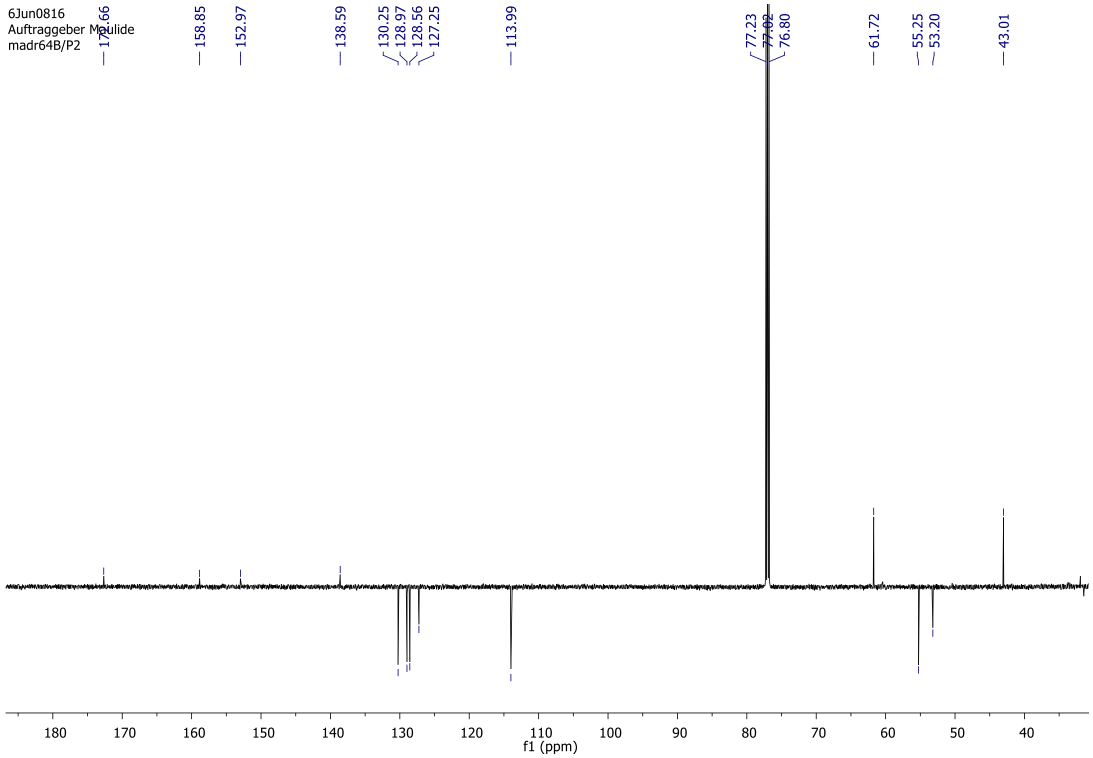

Compound **17b**: ^{1}H NMR

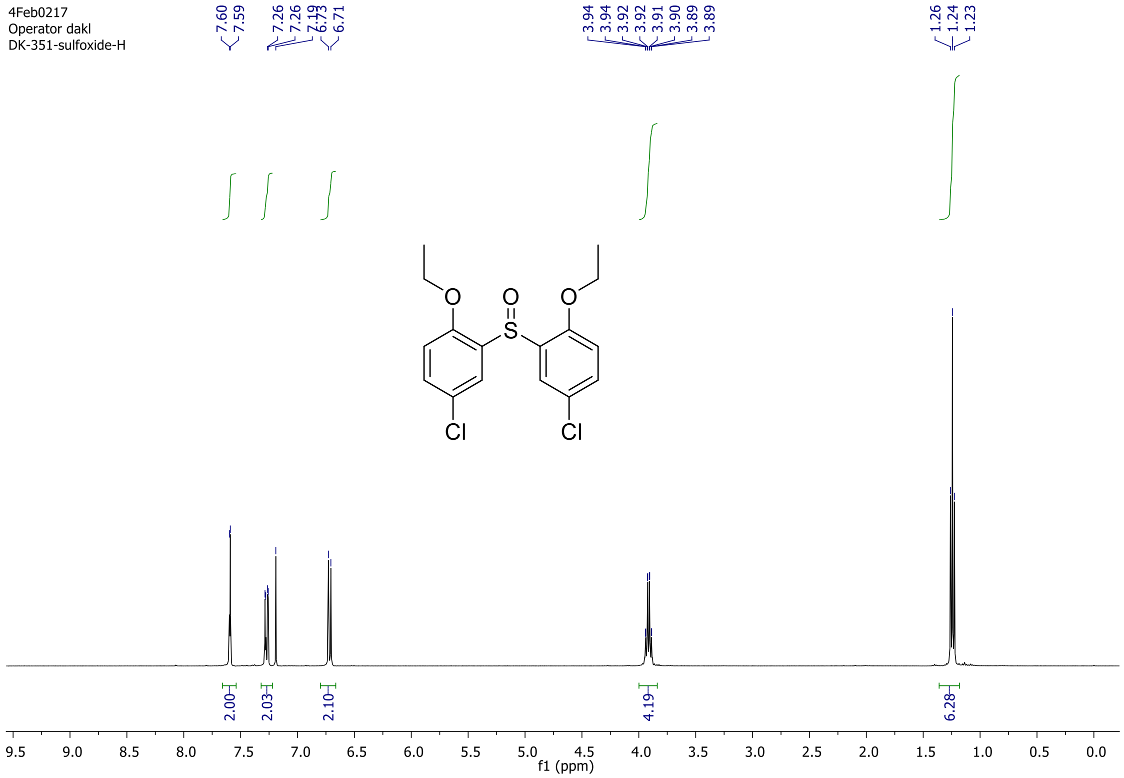

Compound **17b**: ^{13}C NMR

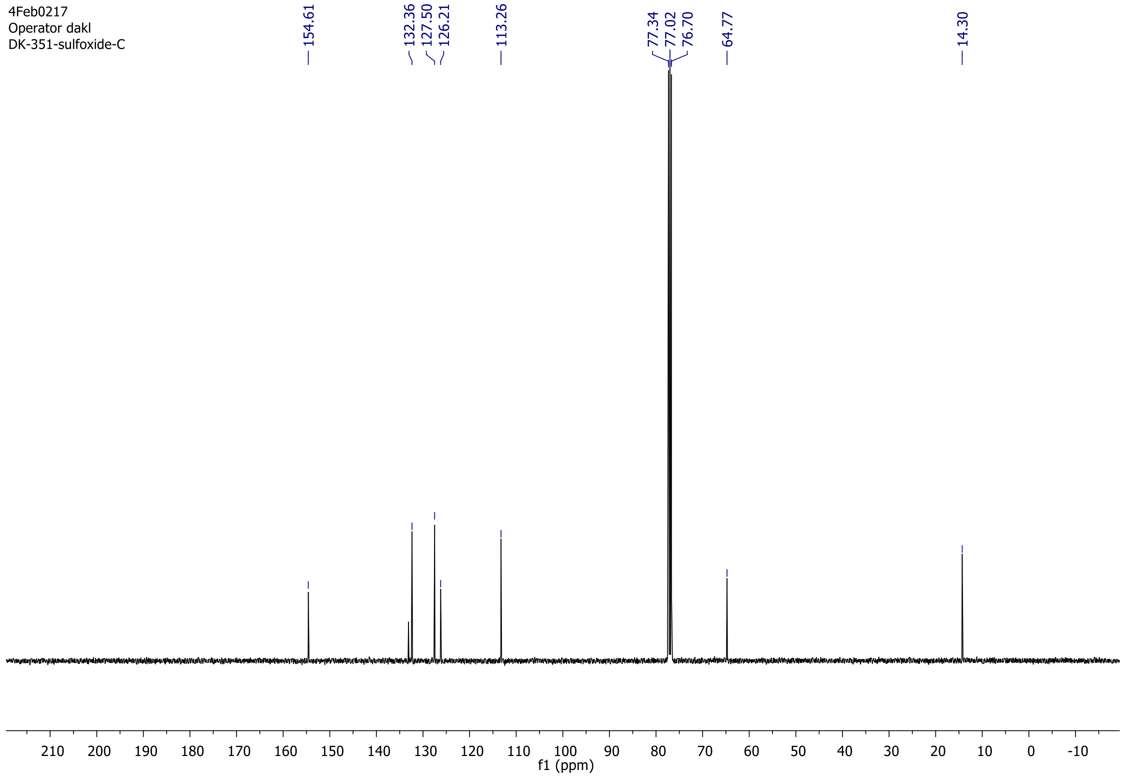

Compound **17a**: ^{1}H NMR

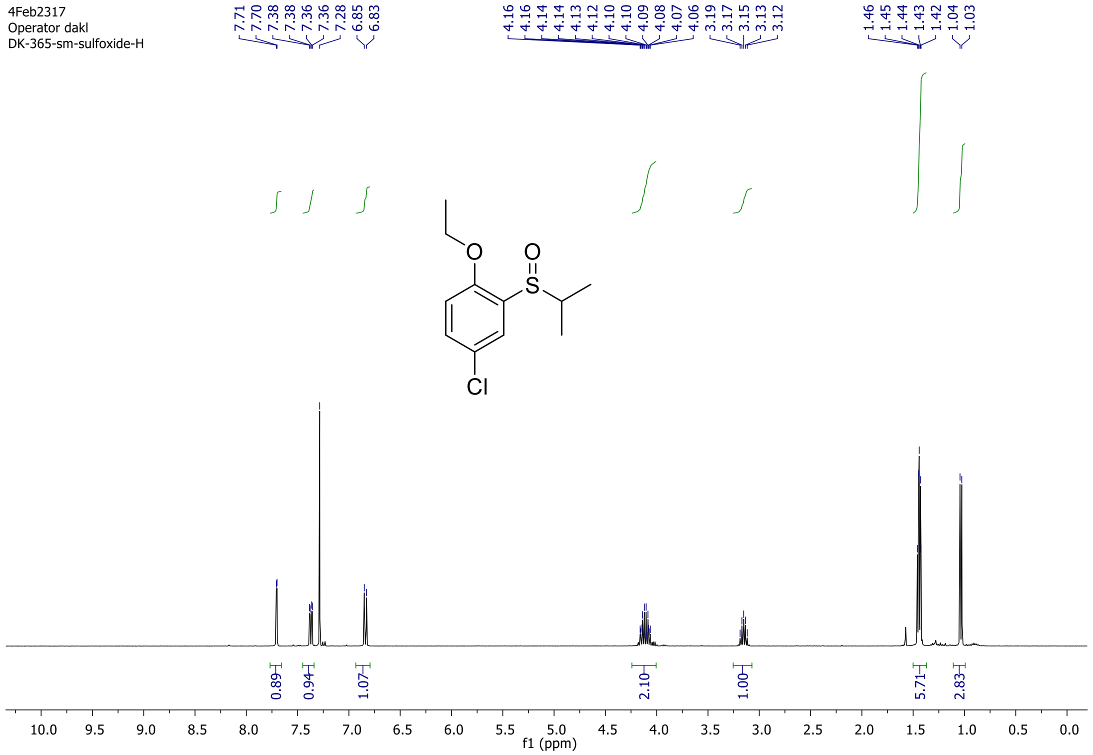

Compound **17a**: ^{13}C NMR

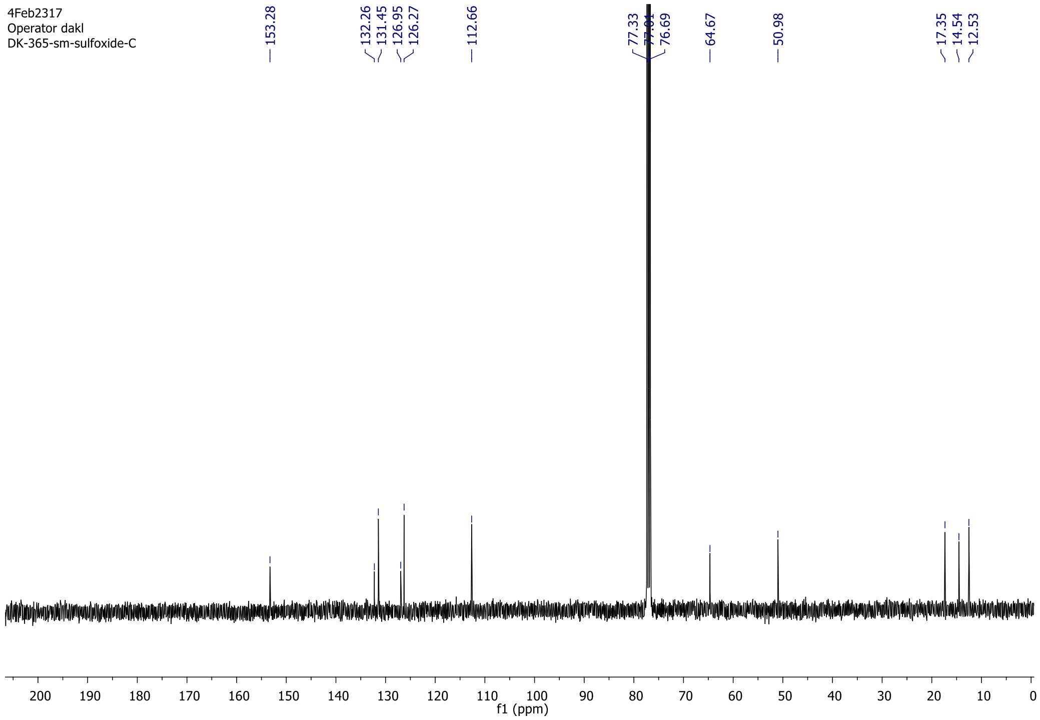

Compound **17d**: ^{1}H NMR

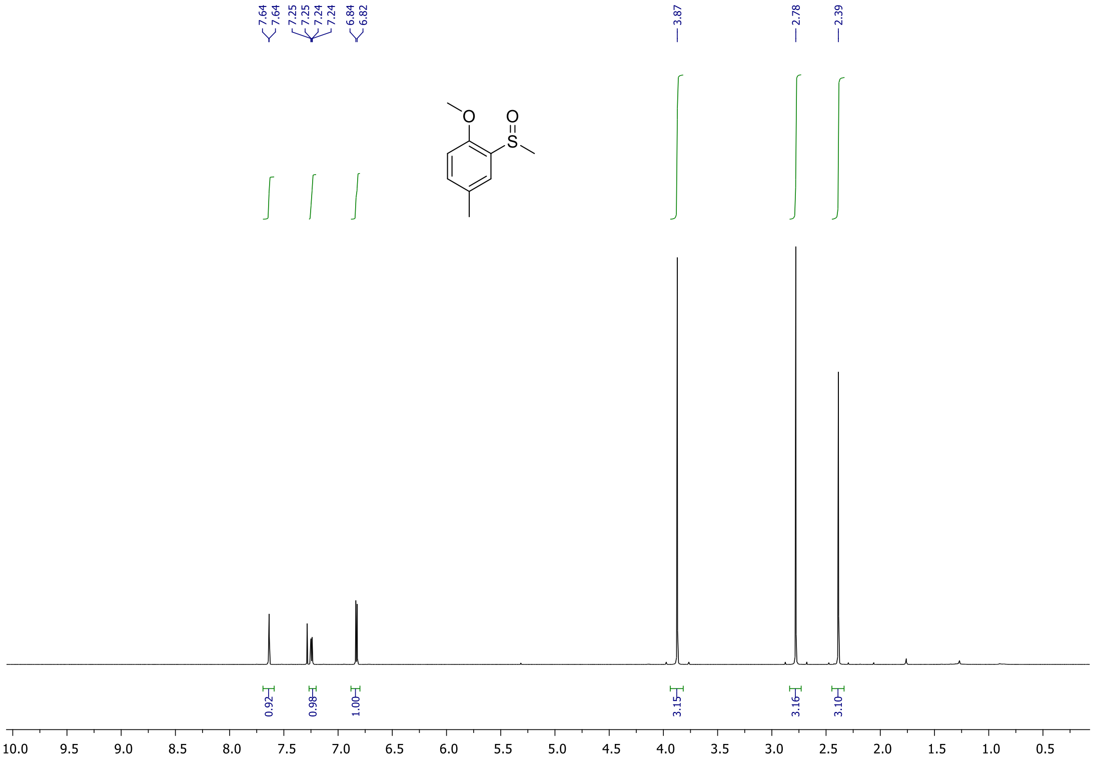

Compound **17d**: ^{13}C NMR

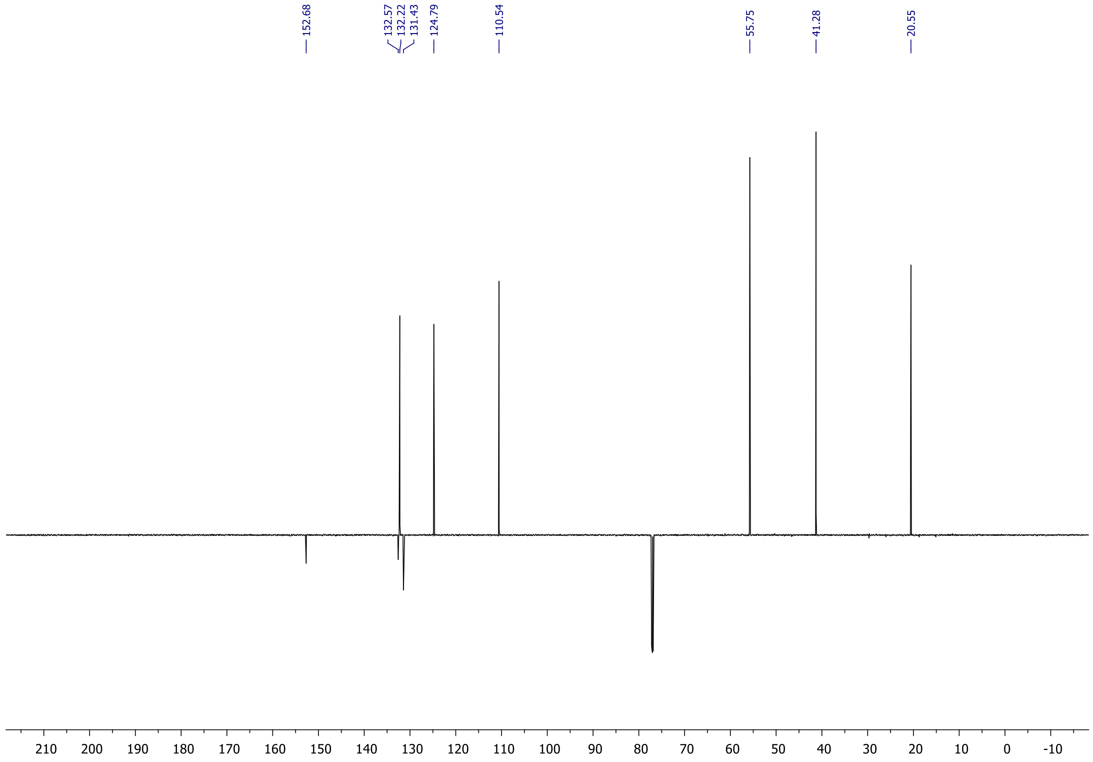

Compound **17l**: ^{1}H NMR

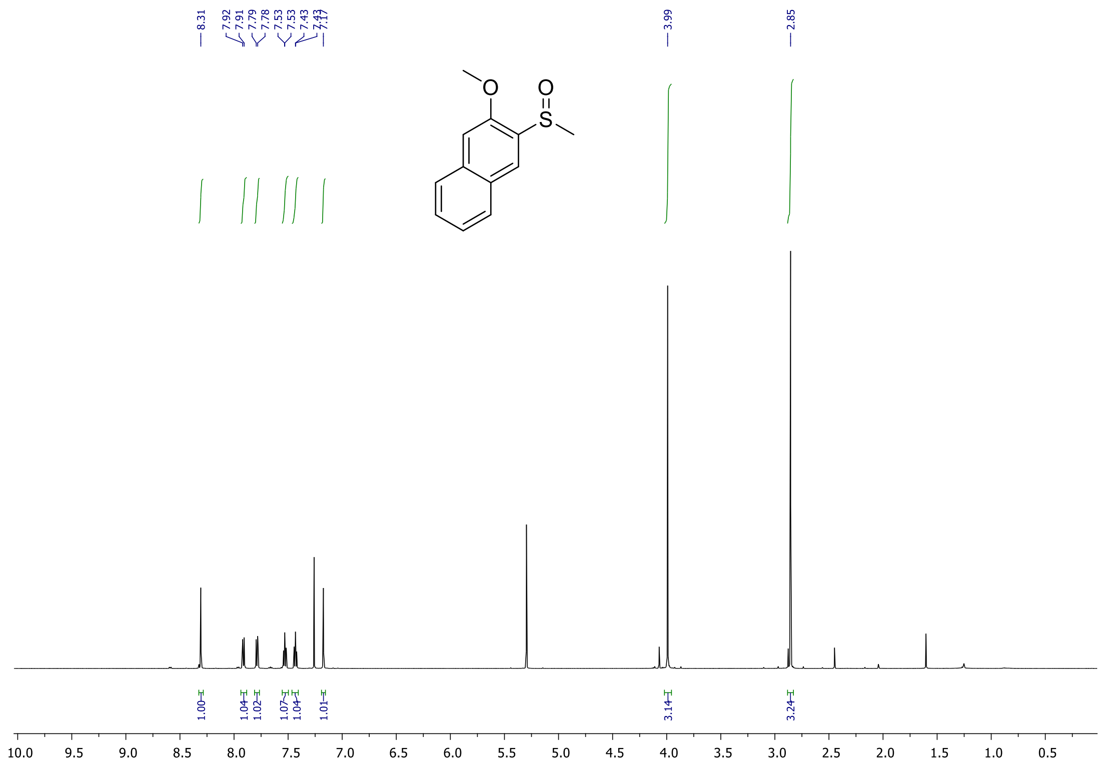

Compound **17l**: ^{13}C NMR

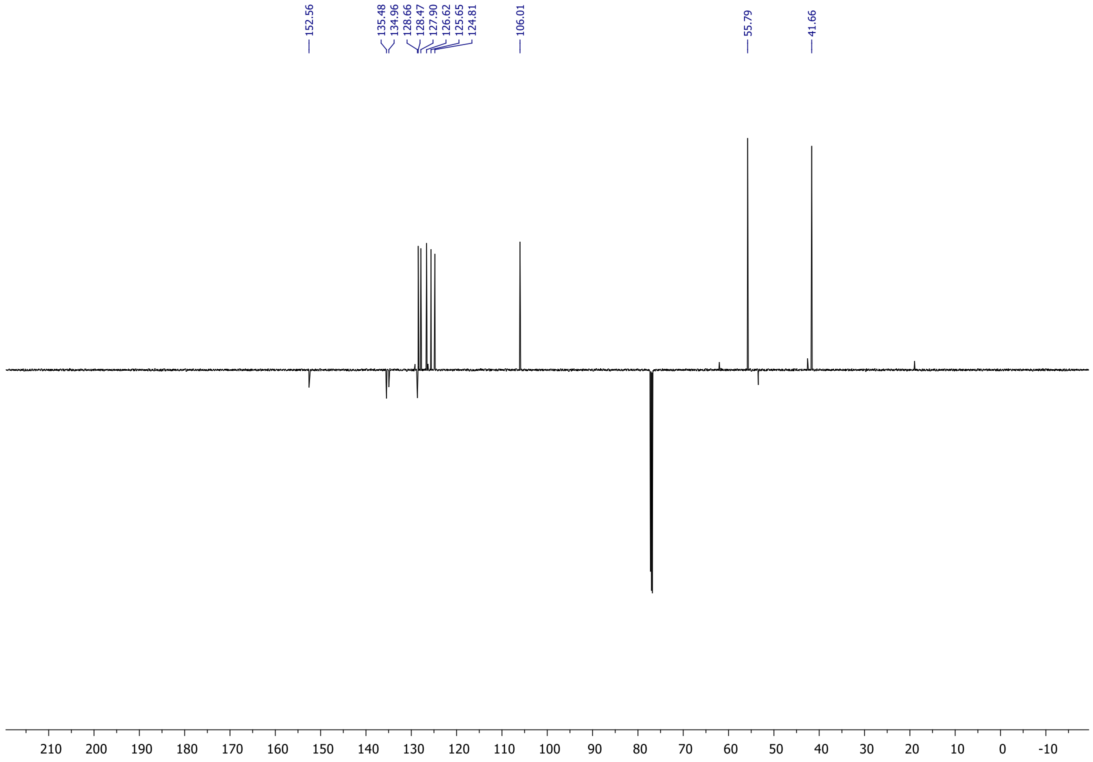

Compound **S17m**: ^{1}H NMR

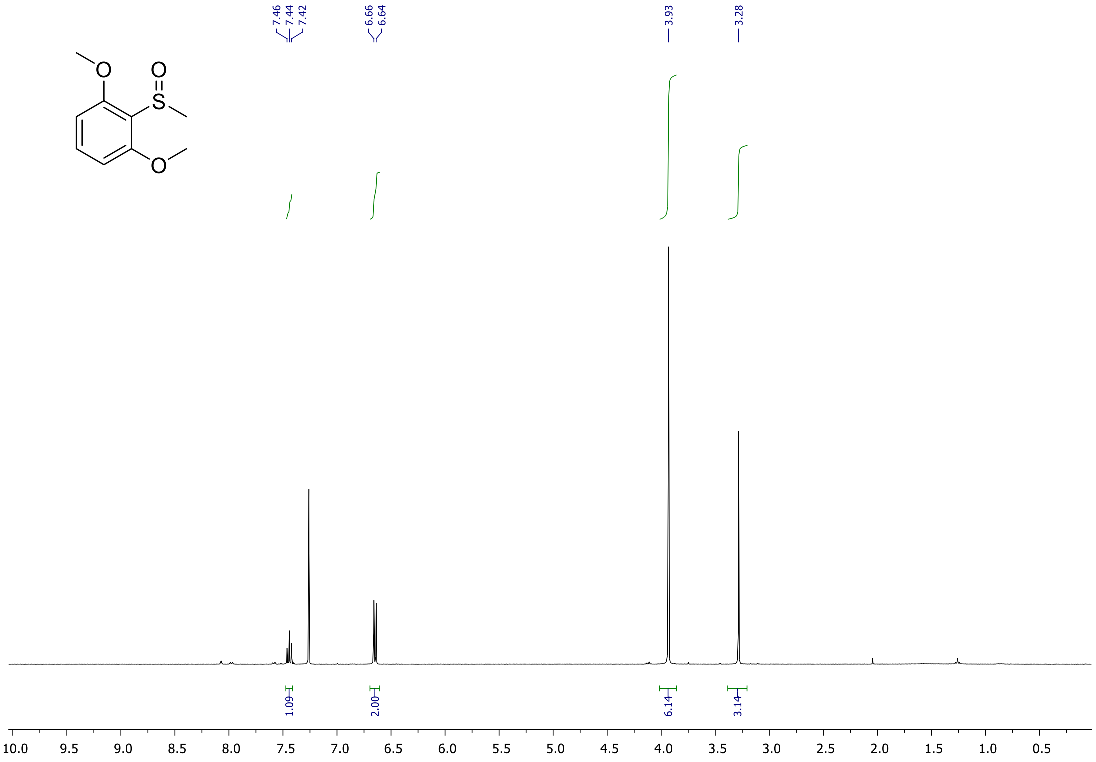

Compund **18a**: ^{1}H NMR

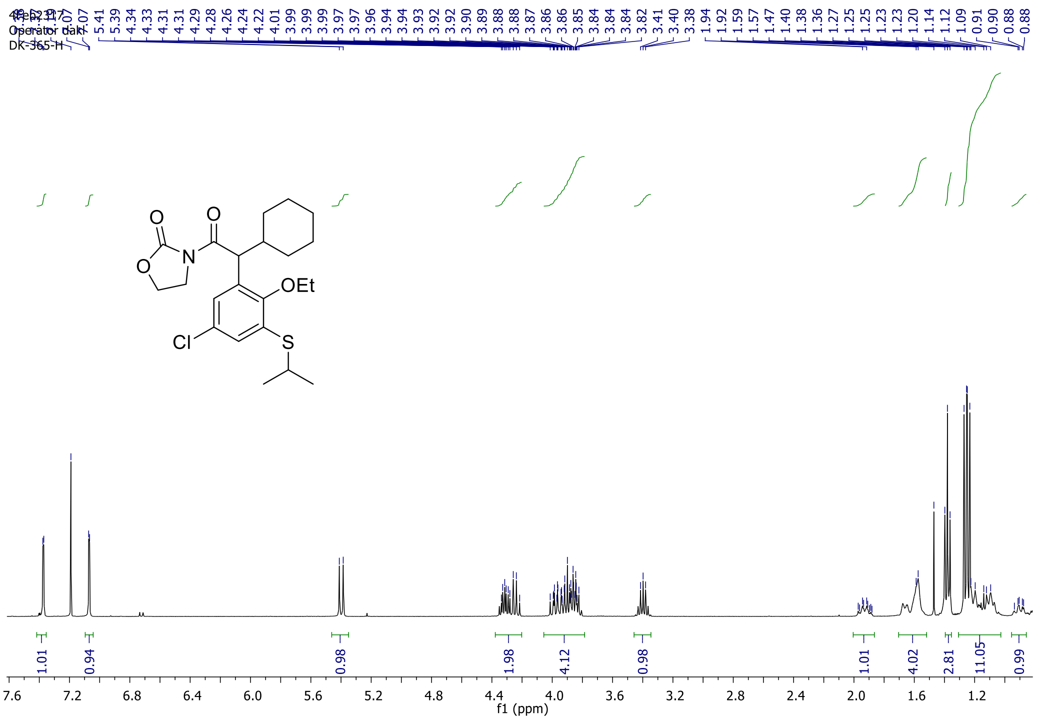

Compound **18a**: ^{13}C NMR

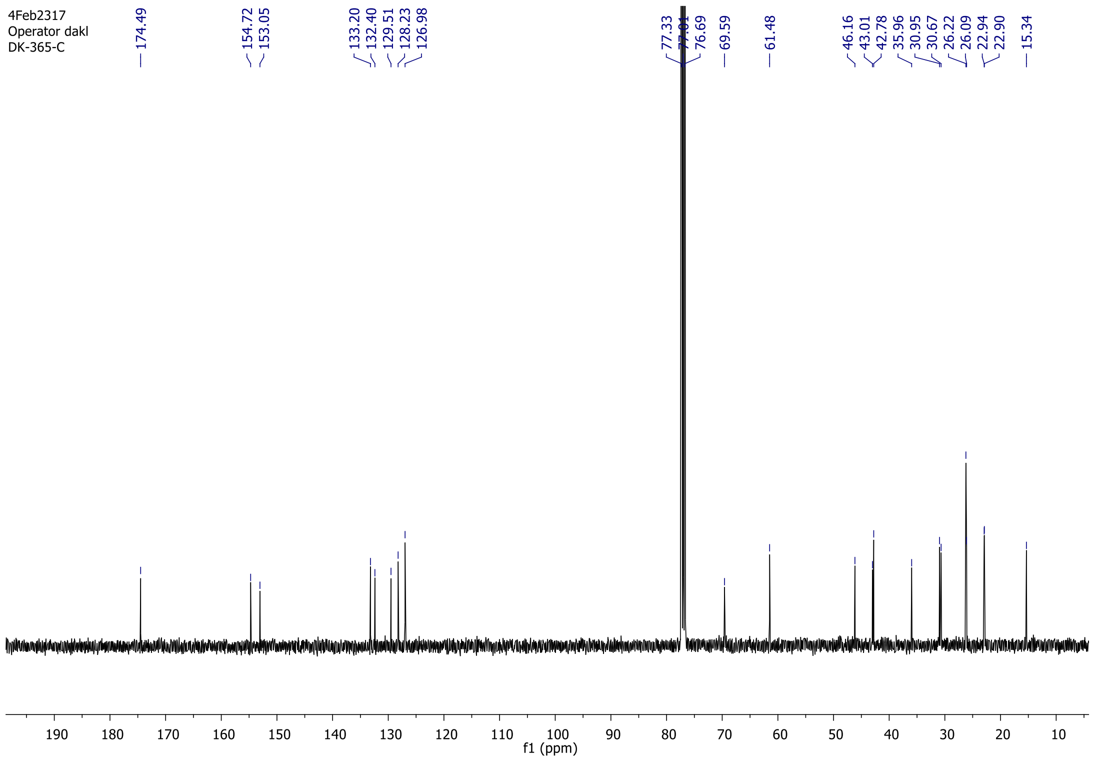

Compound **18b**: ^{1}H NMR

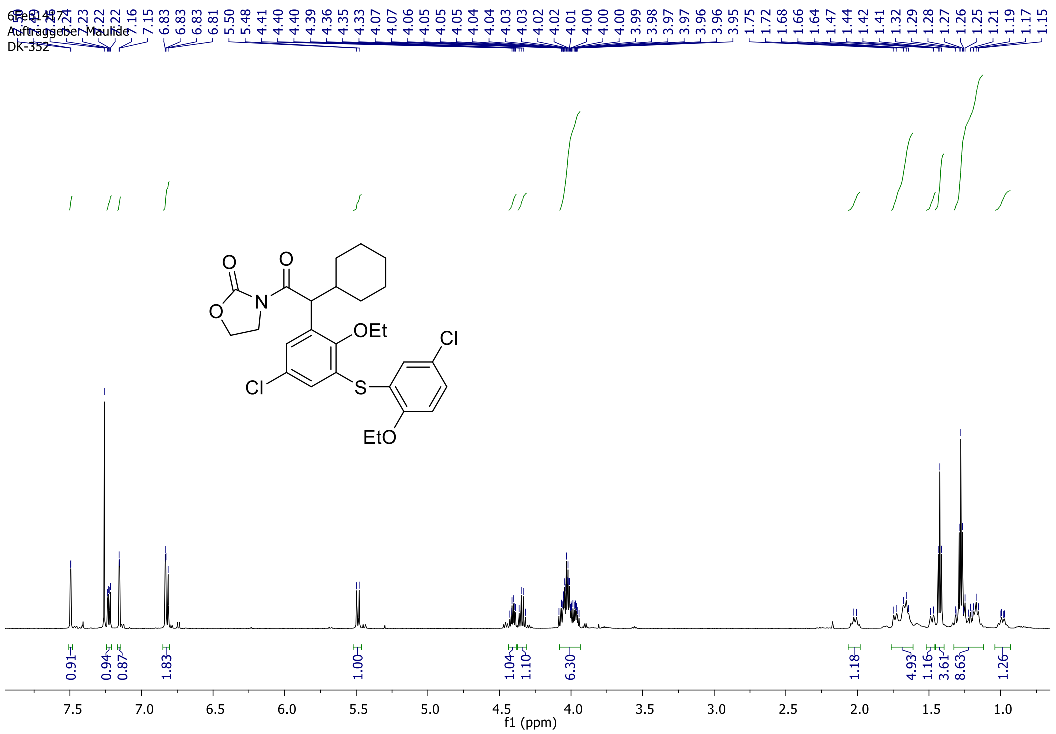

Compound **18b**: ^{13}C NMR

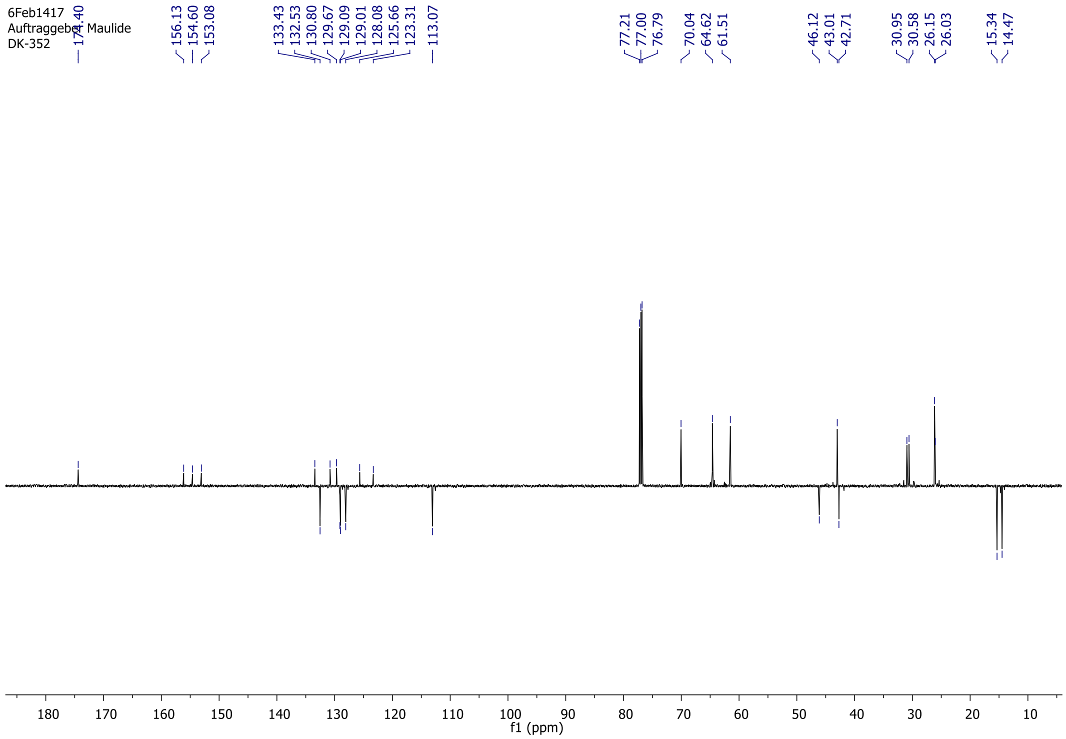

Compound **18c**: ^{1}H NMR

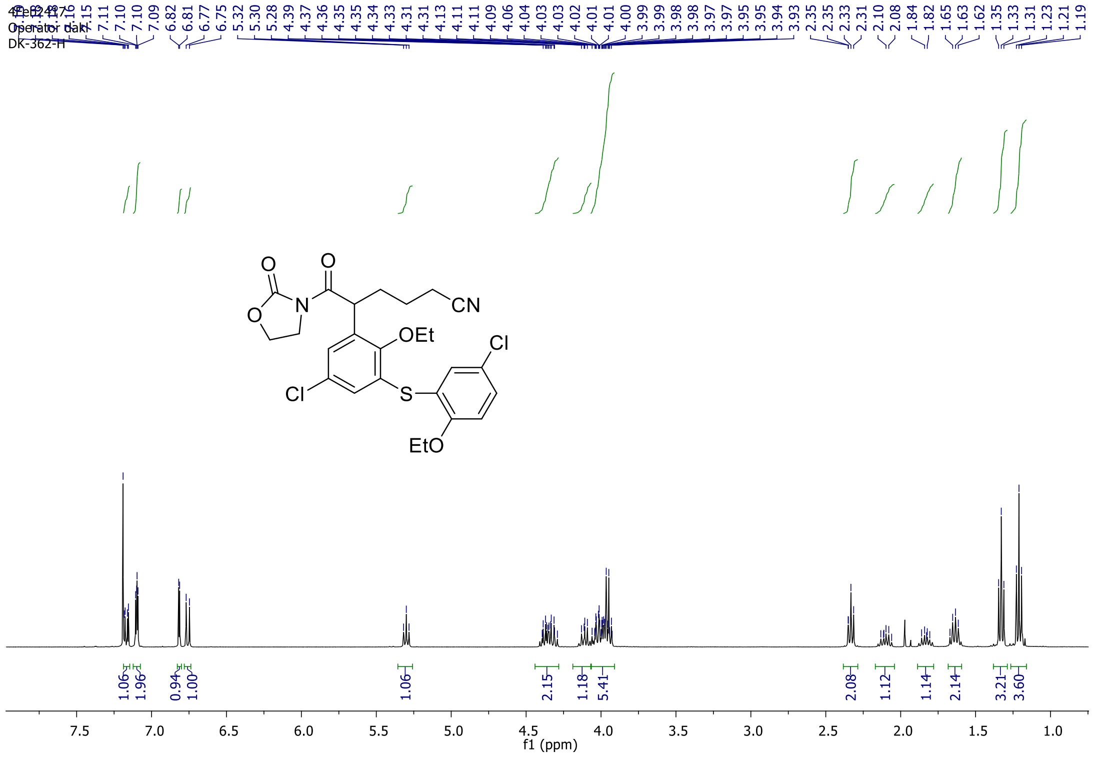

Compound **18c**: ^{13}C NMR

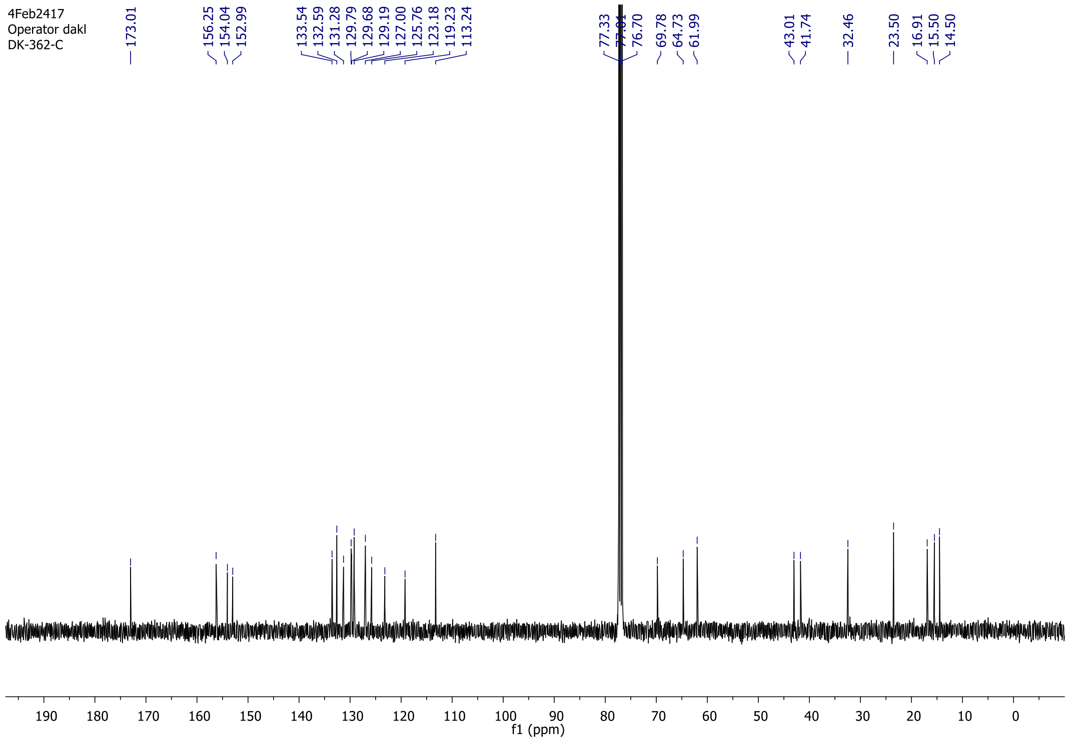

Compound **18d**: ^{1}H NMR

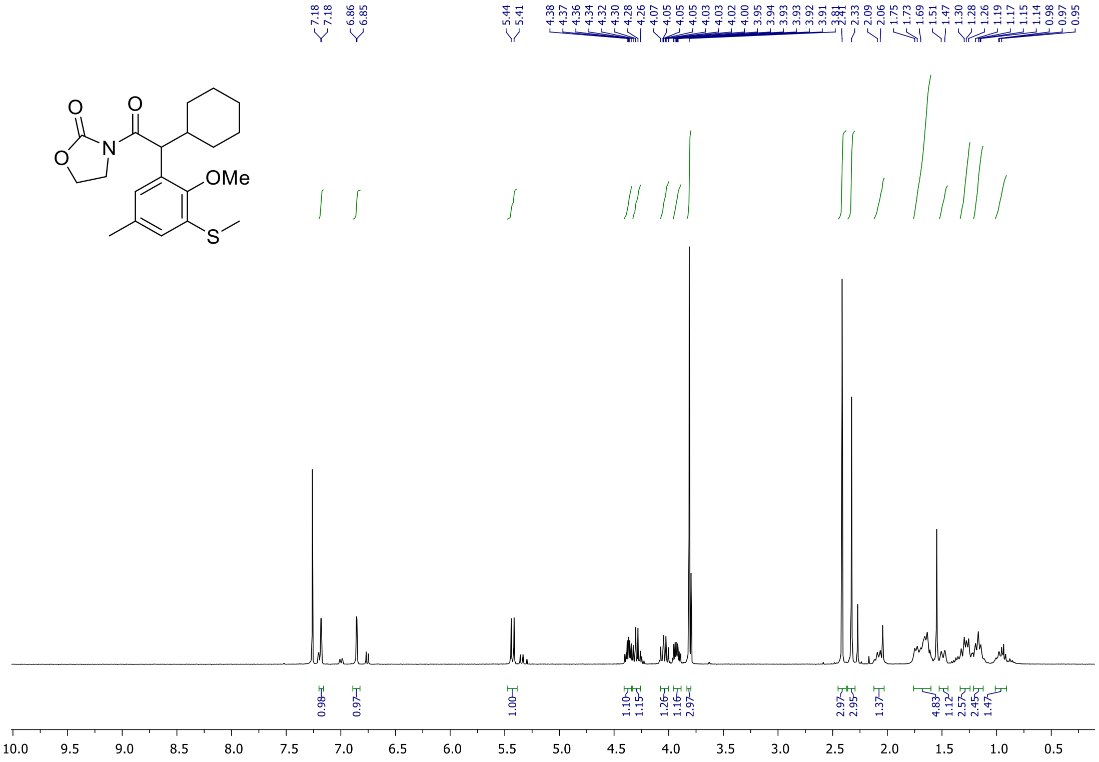

Compound **18d**: ^{13}C NMR

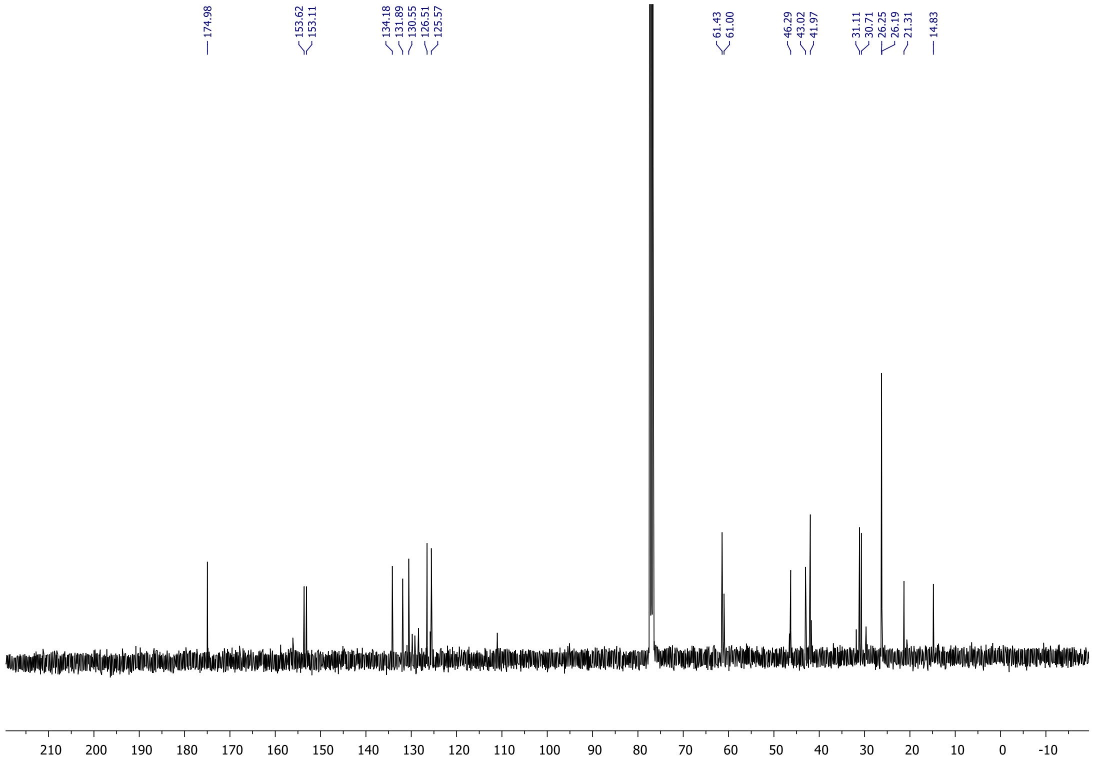

Compound **18e+18e’**: ^{1}H NMR

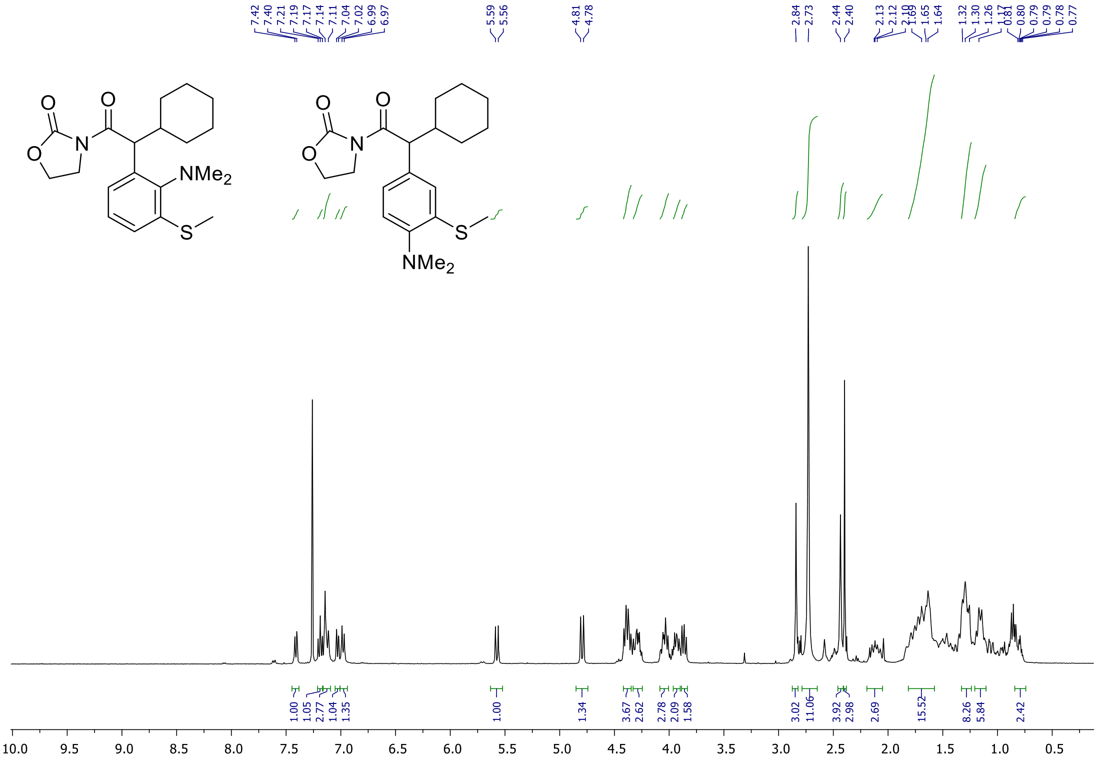

Compound **18e+18e’**: ^{13}C NMR

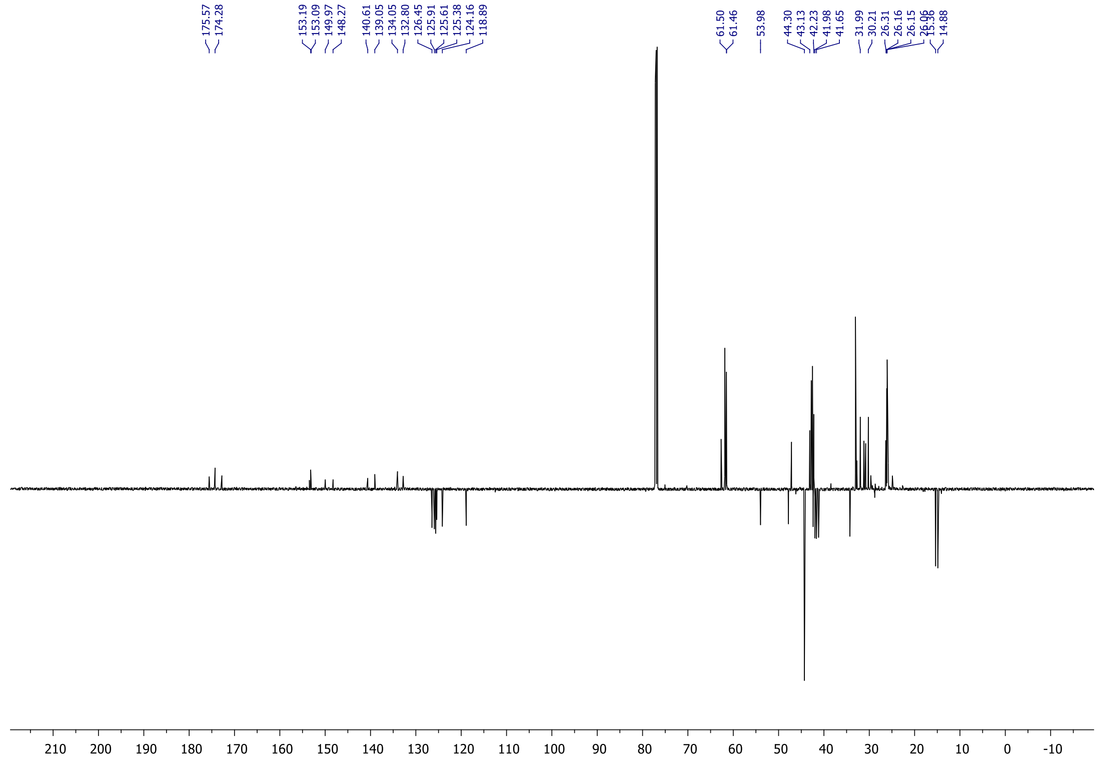

NOESY of **18e**

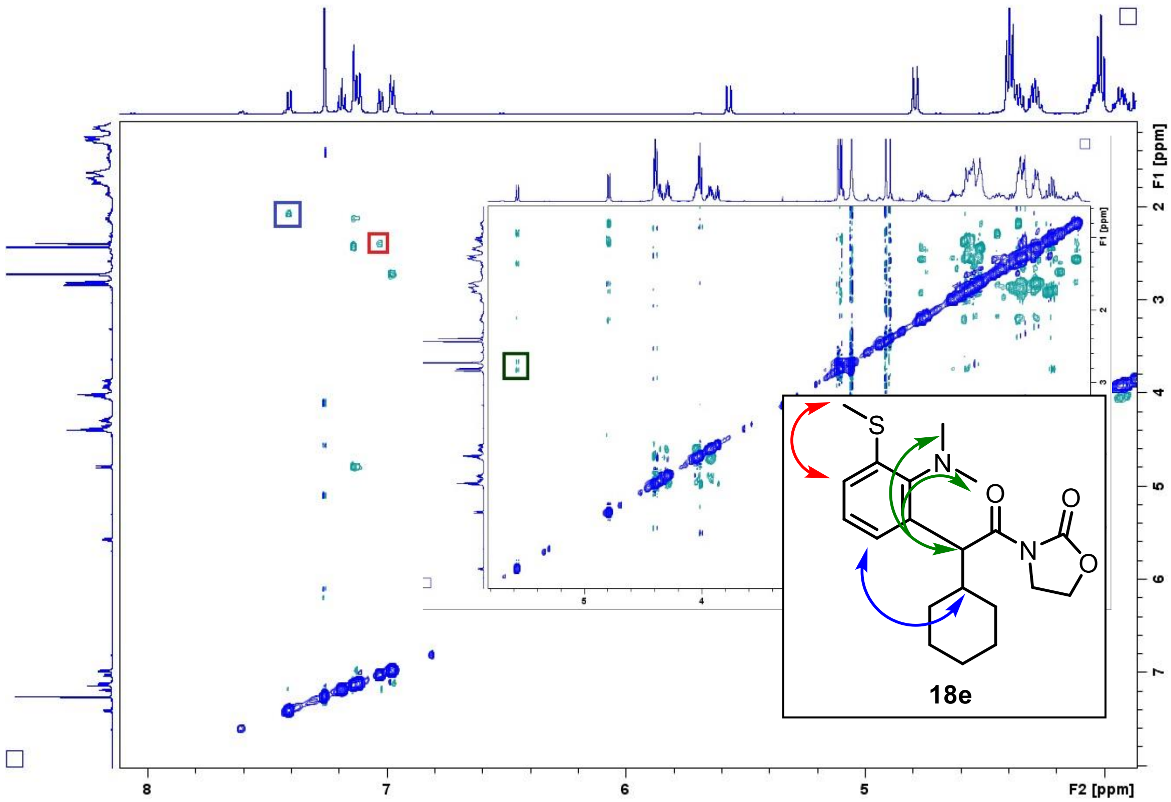

NOESY of **18e'**

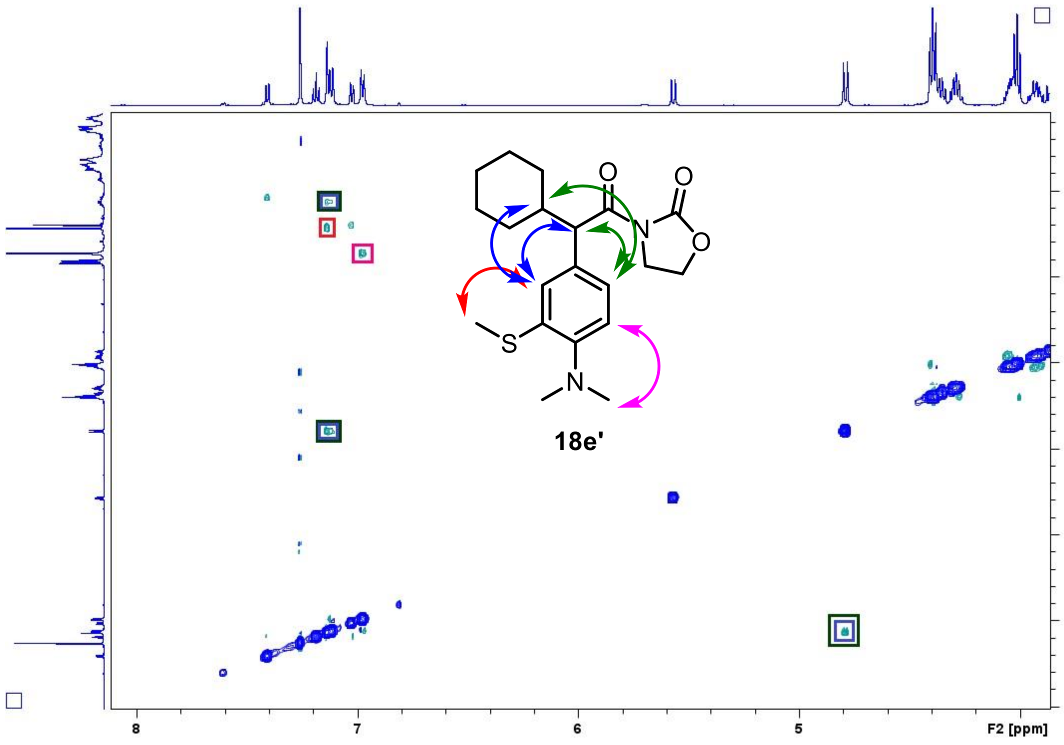

Compound **18f**: ^{1}H NMR

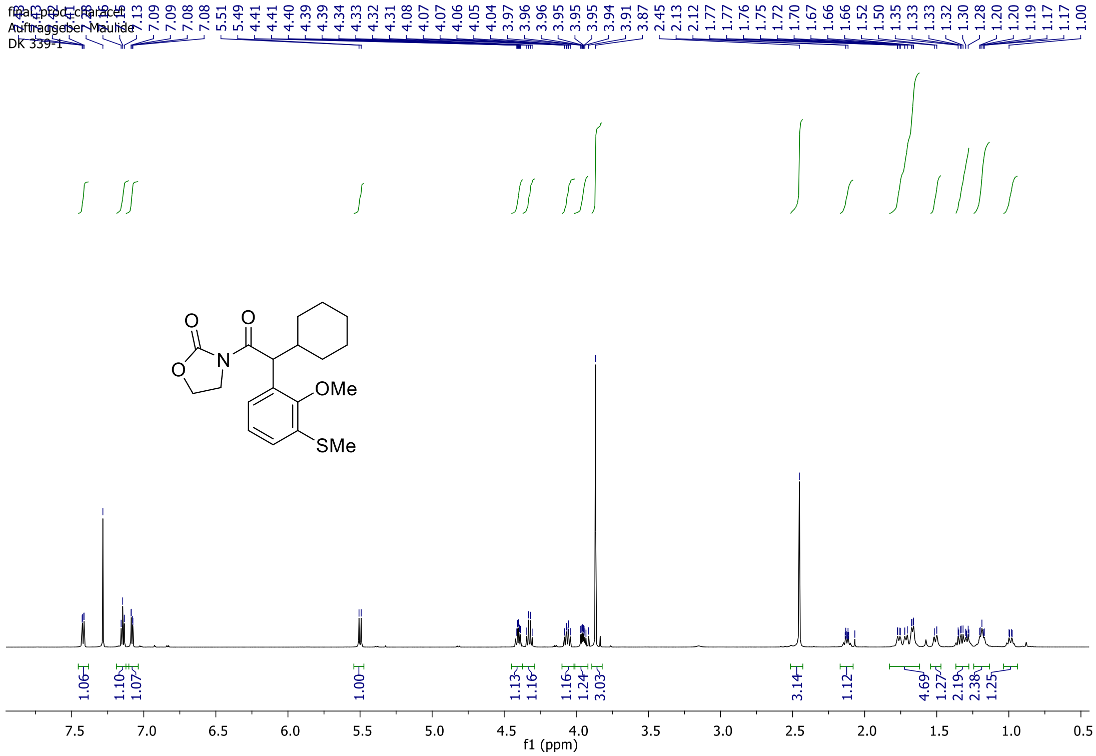

Compound **18f**: ^{13}C NMR

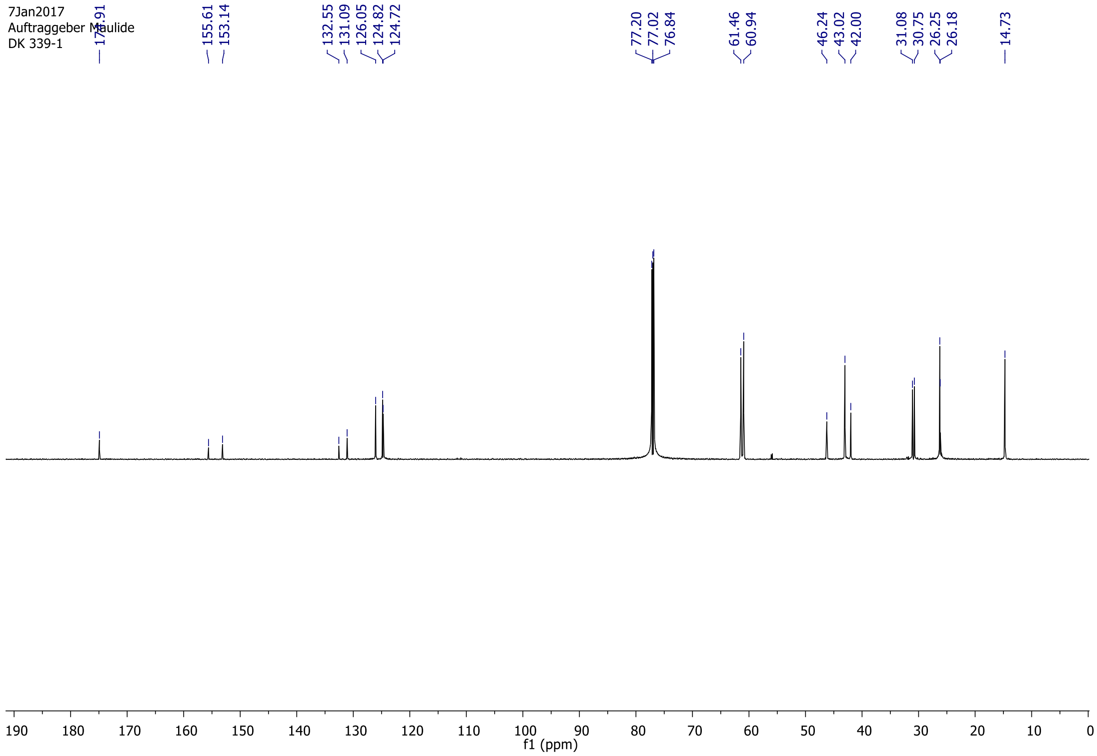

Compound **19f**: ^{1}H NMR

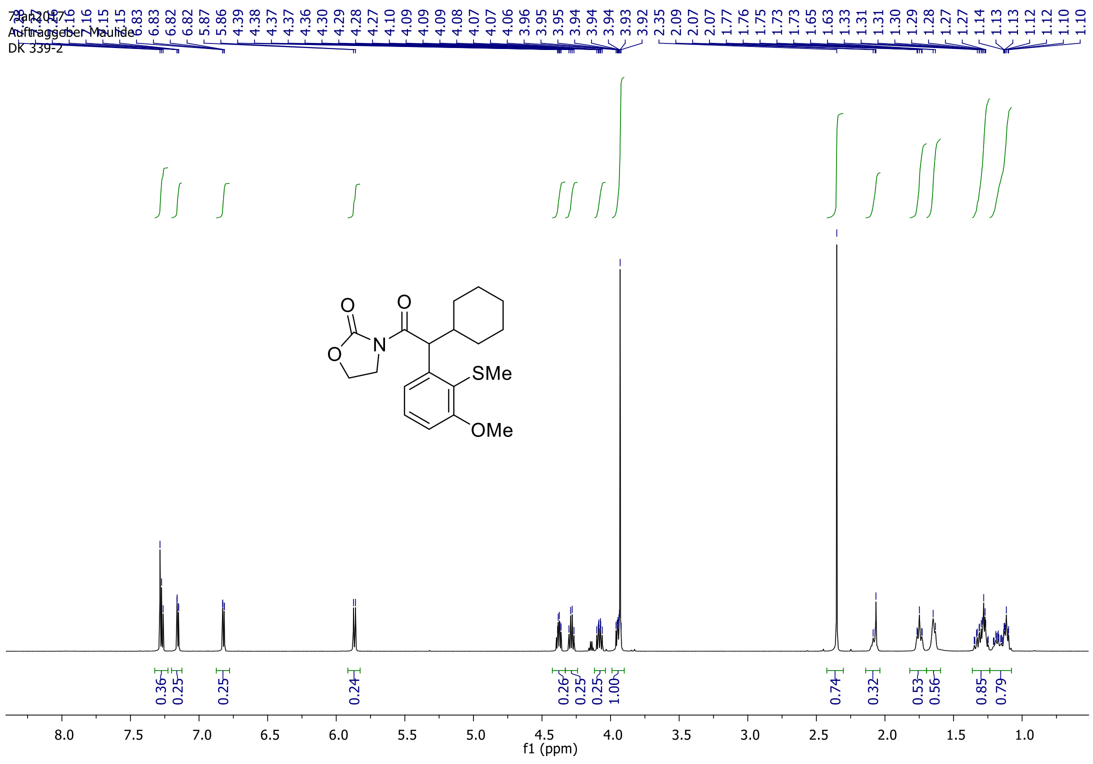

Compound **19f**: ^{13}C NMR

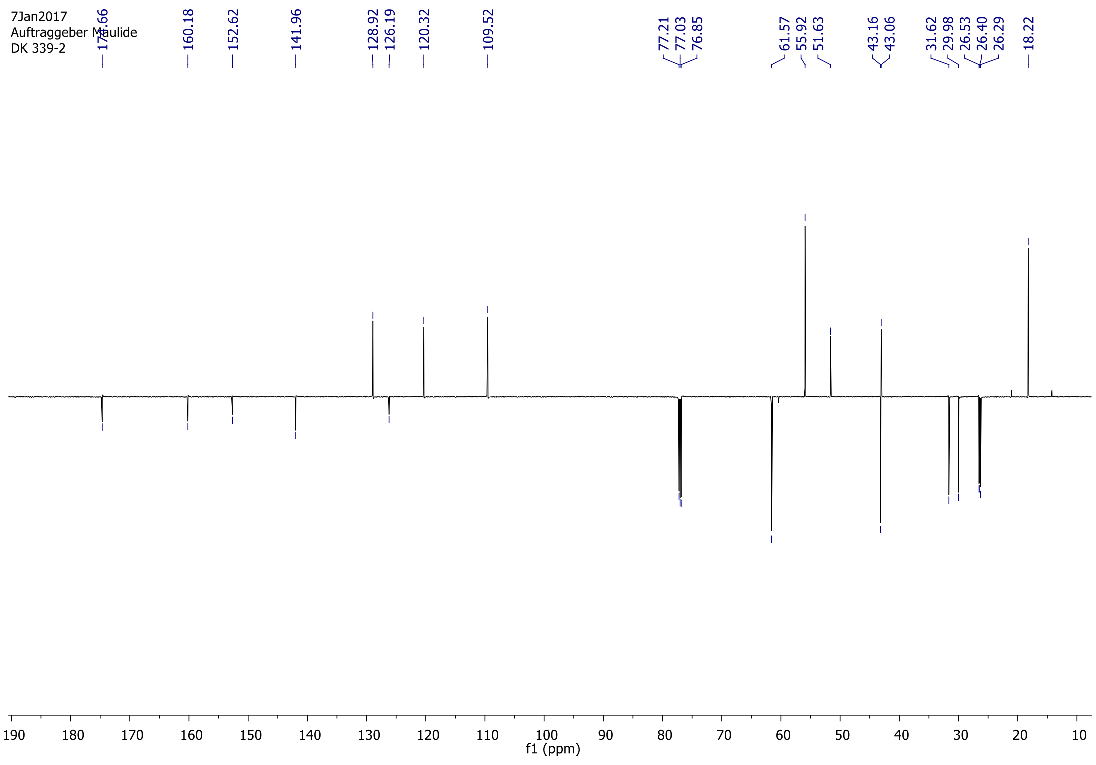

Compound **18g**: ^{1}H NMR

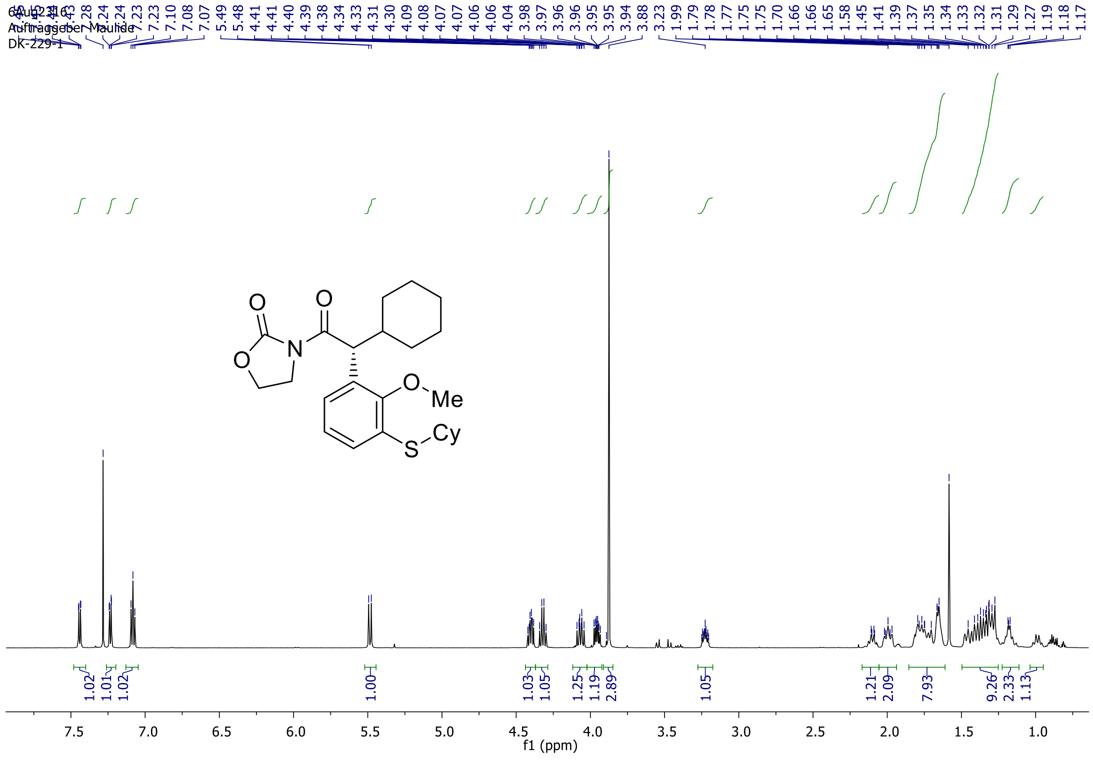

Compound **18g**: ^{13}C NMR

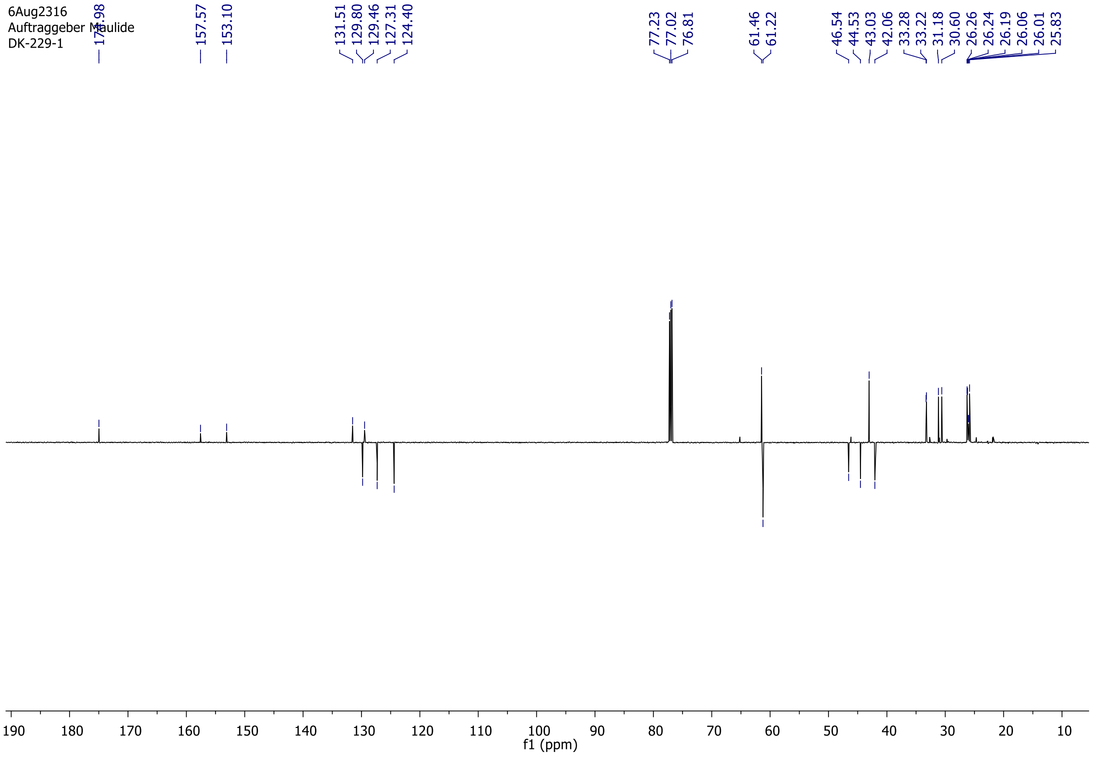

Compound **19g**: ^{1}H NMR

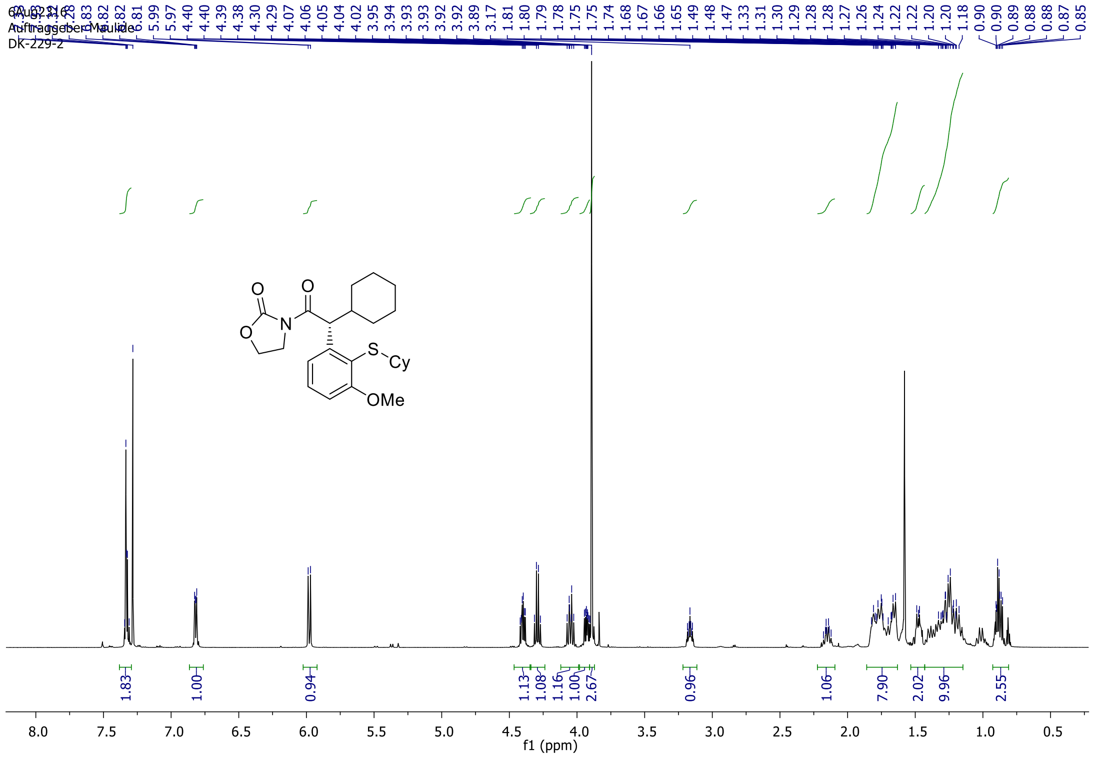

Compound **19g**: ^{13}C NMR

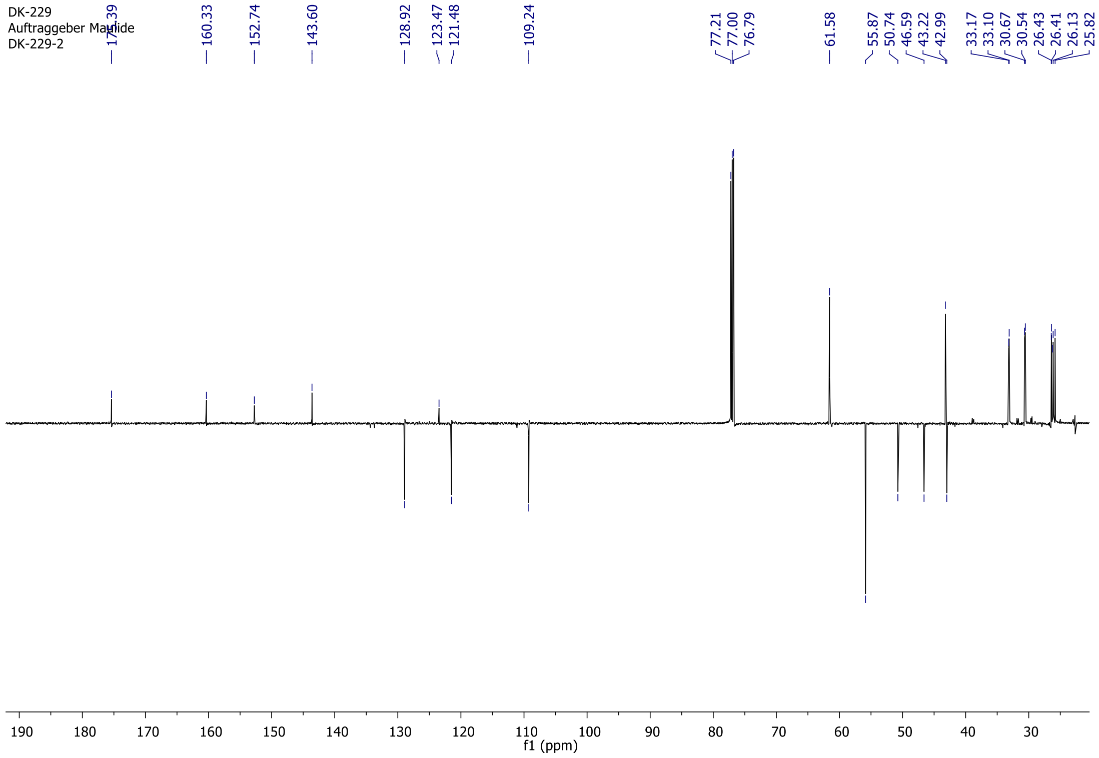

Compound **18h**: ^{1}H NMR

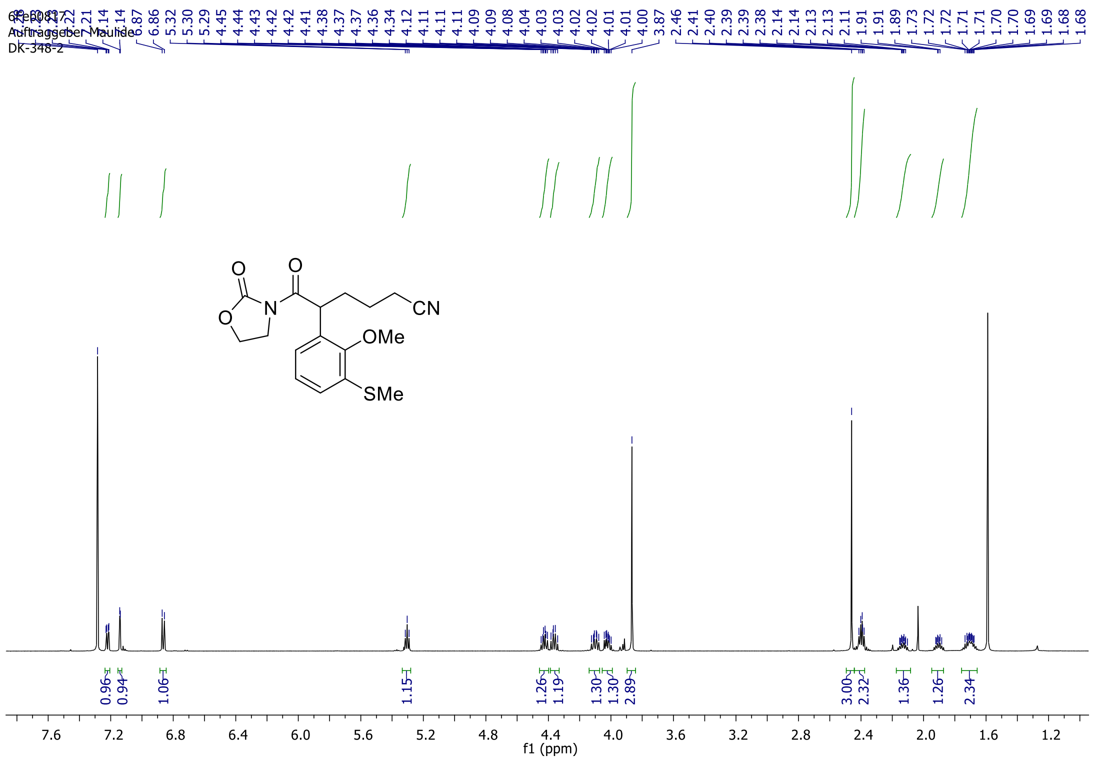

Compound **18h**: ^{13}C NMR

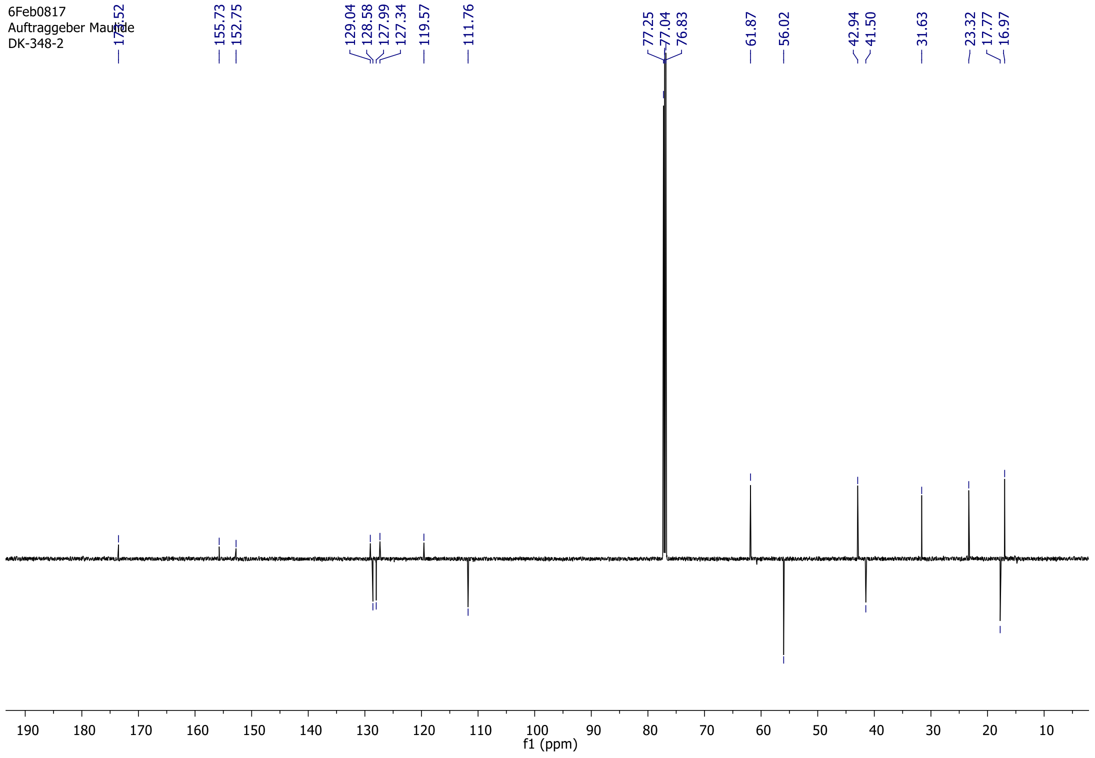

Compound **19h**: ^{1}H NMR

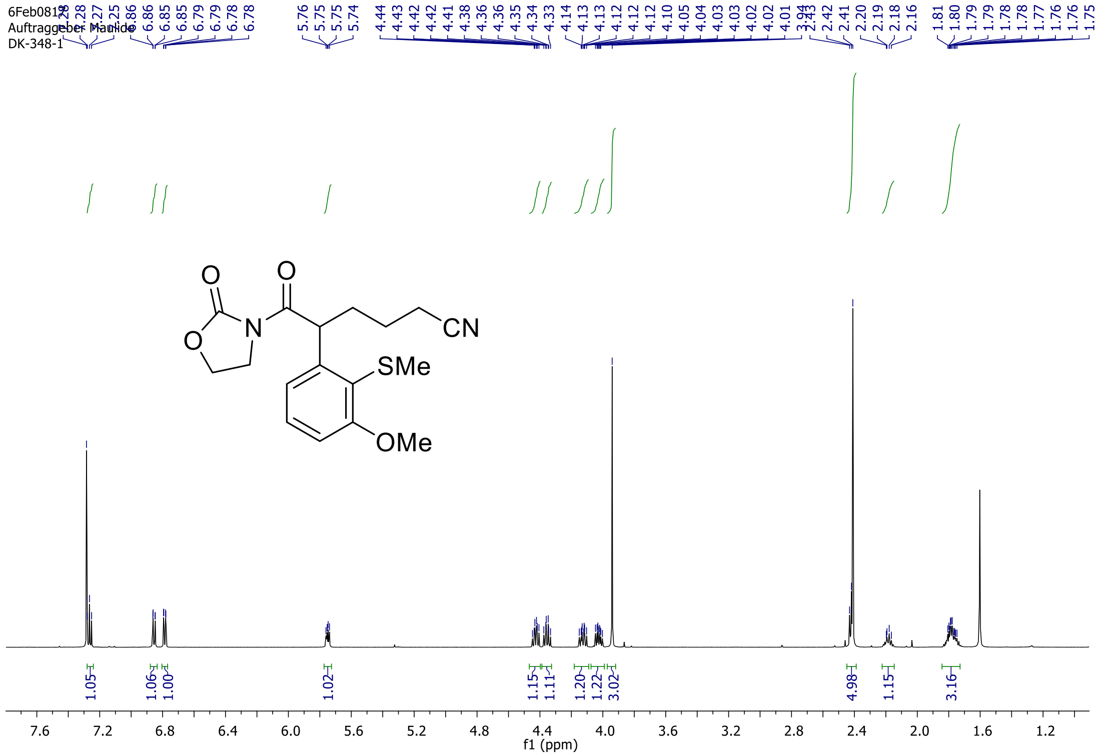

Compound **19h**: ^{13}C NMR

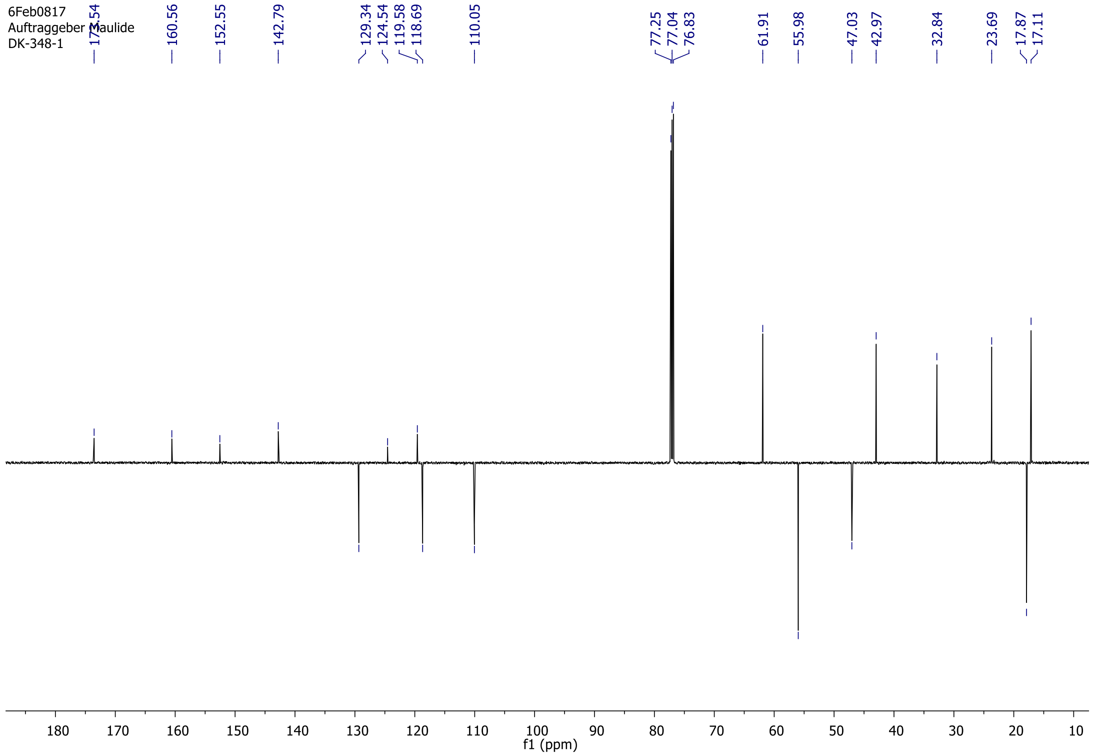

Compound **19i**: ^{1}H NMR

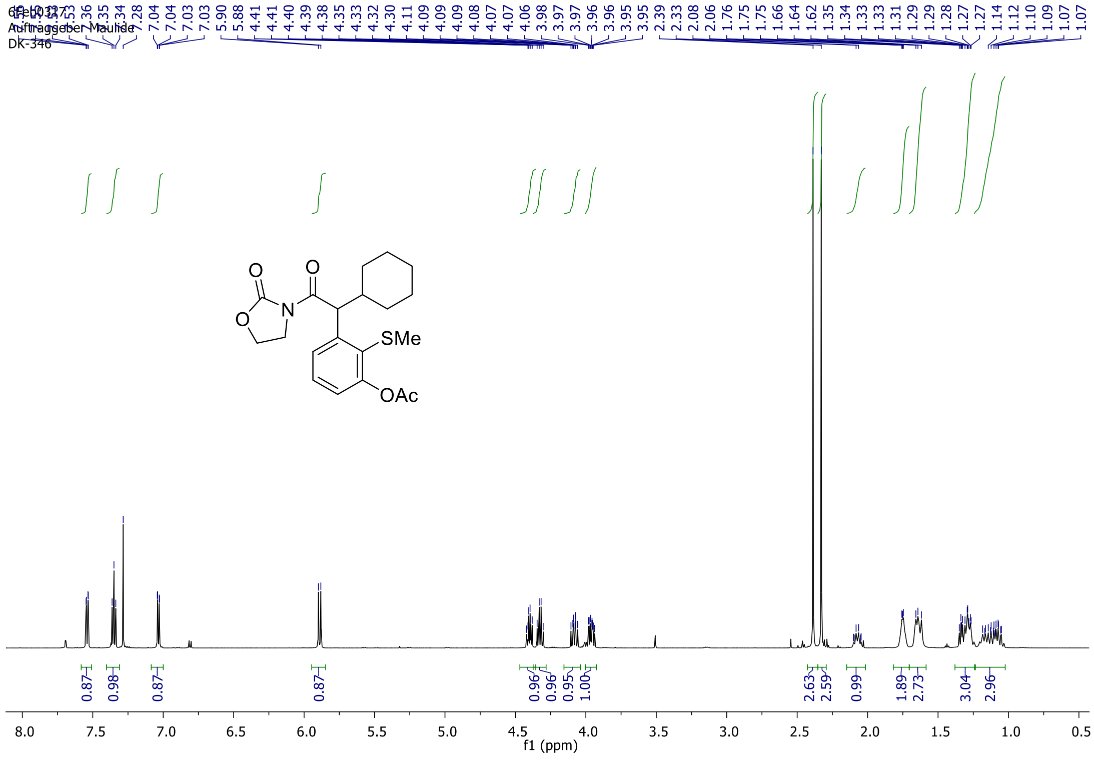

Compound **19i**: ^{13}C NMR

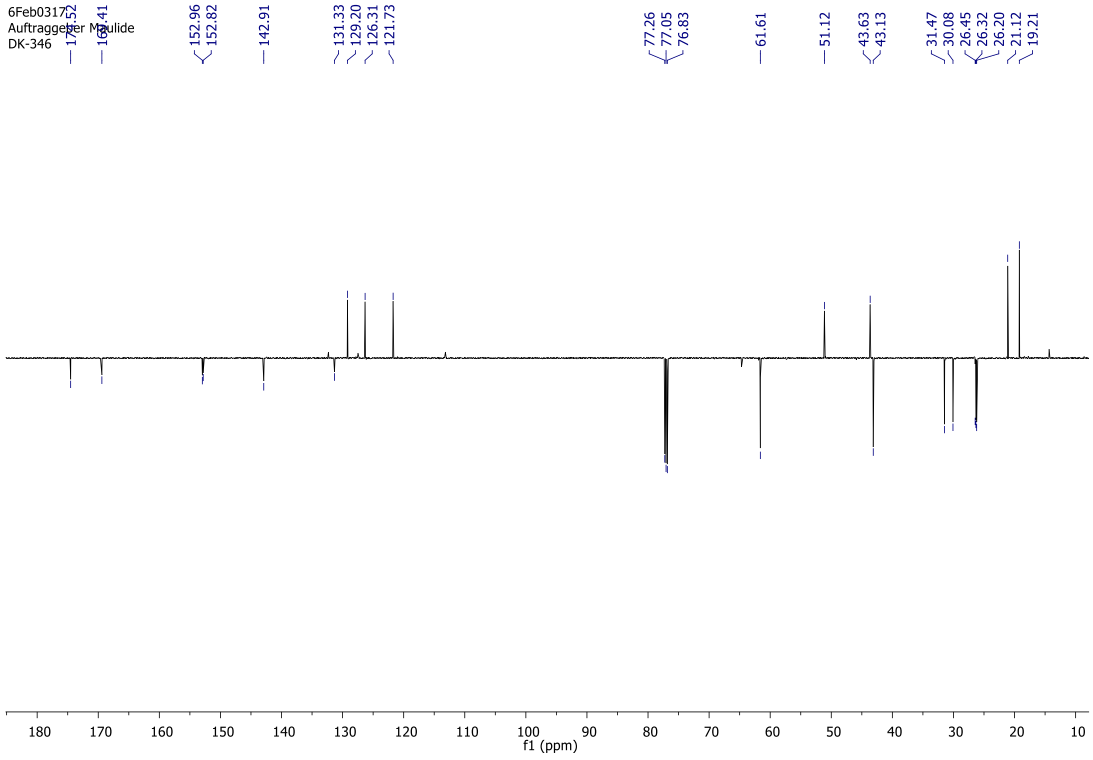

Compound **19j**: ^{1}H NMR

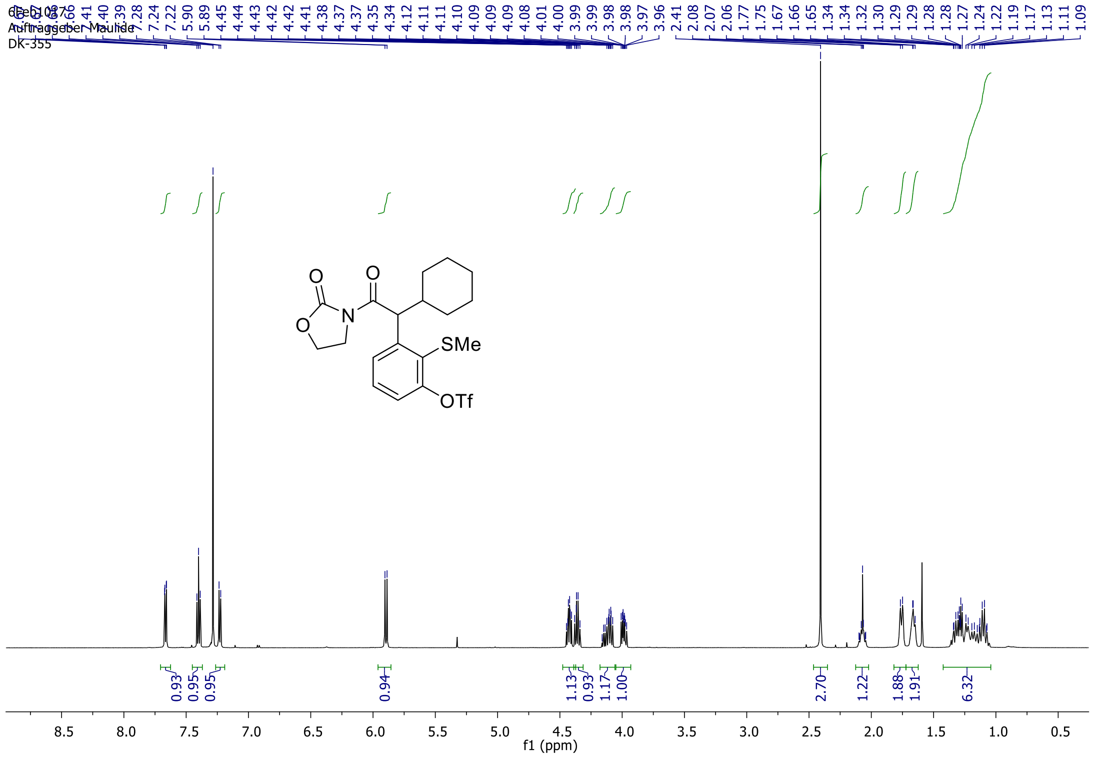

Compound **19j**: ^{13}C NMR

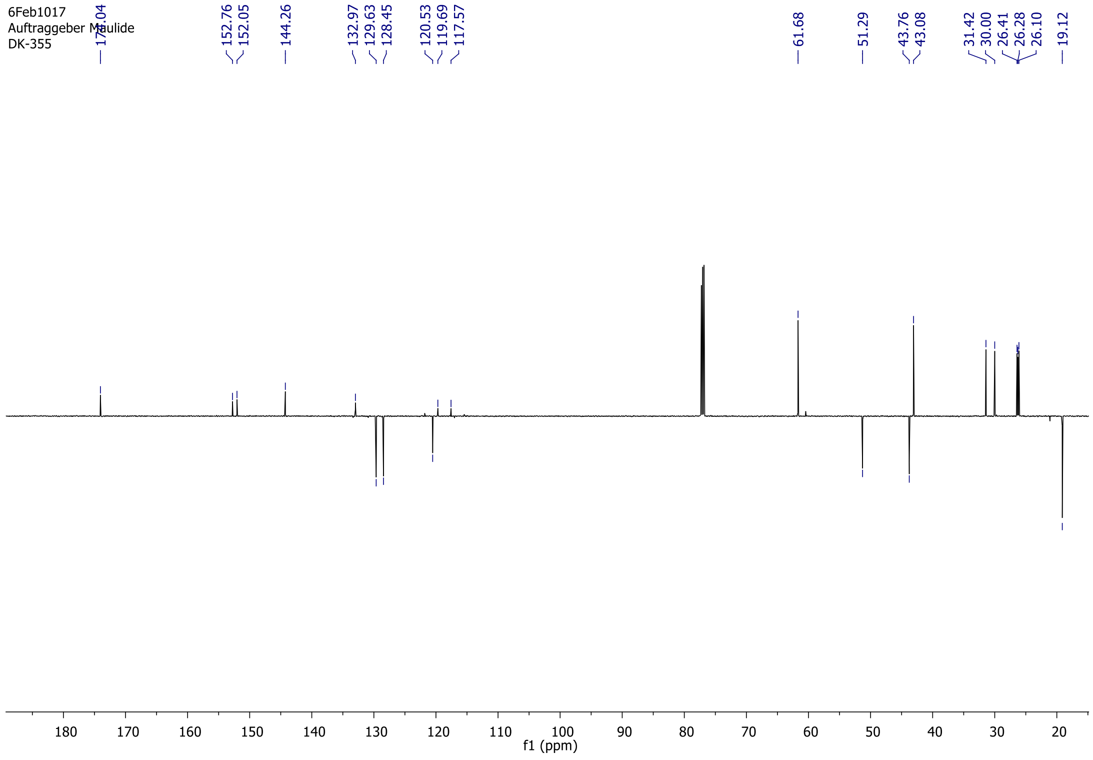

Compound **19k**: ^{1}H NMR

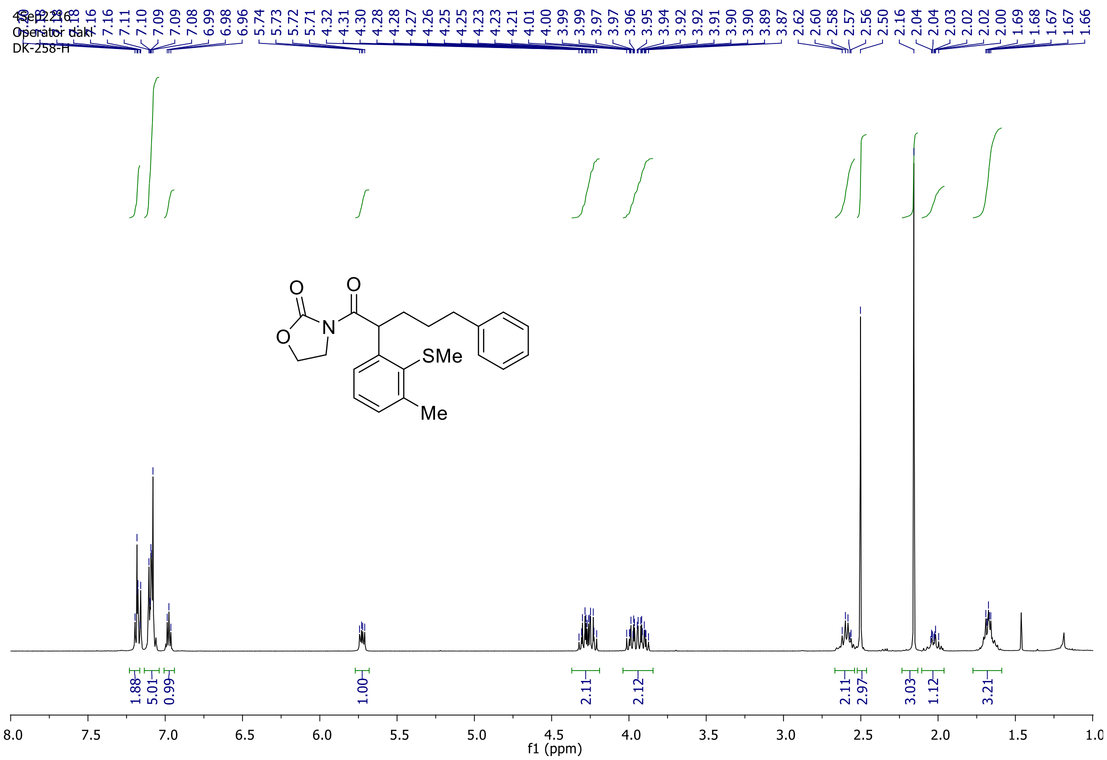

Compound **19k**: ^{13}C NMR

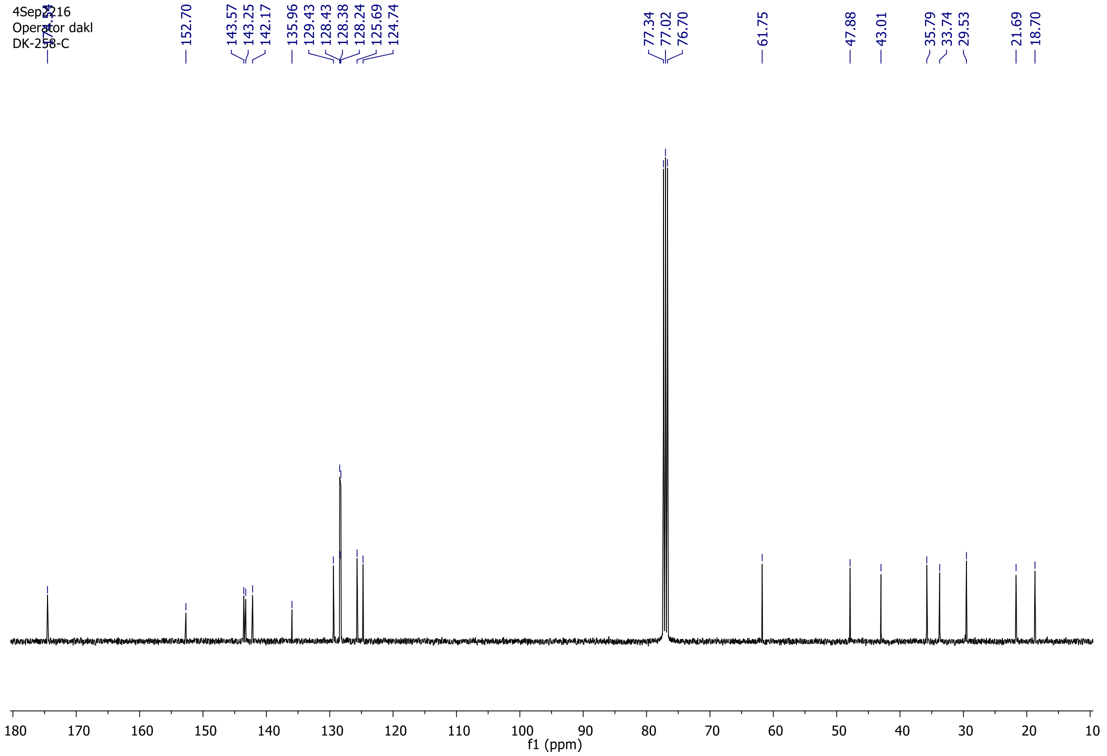

Compound **19l**: ^{1}H NMR

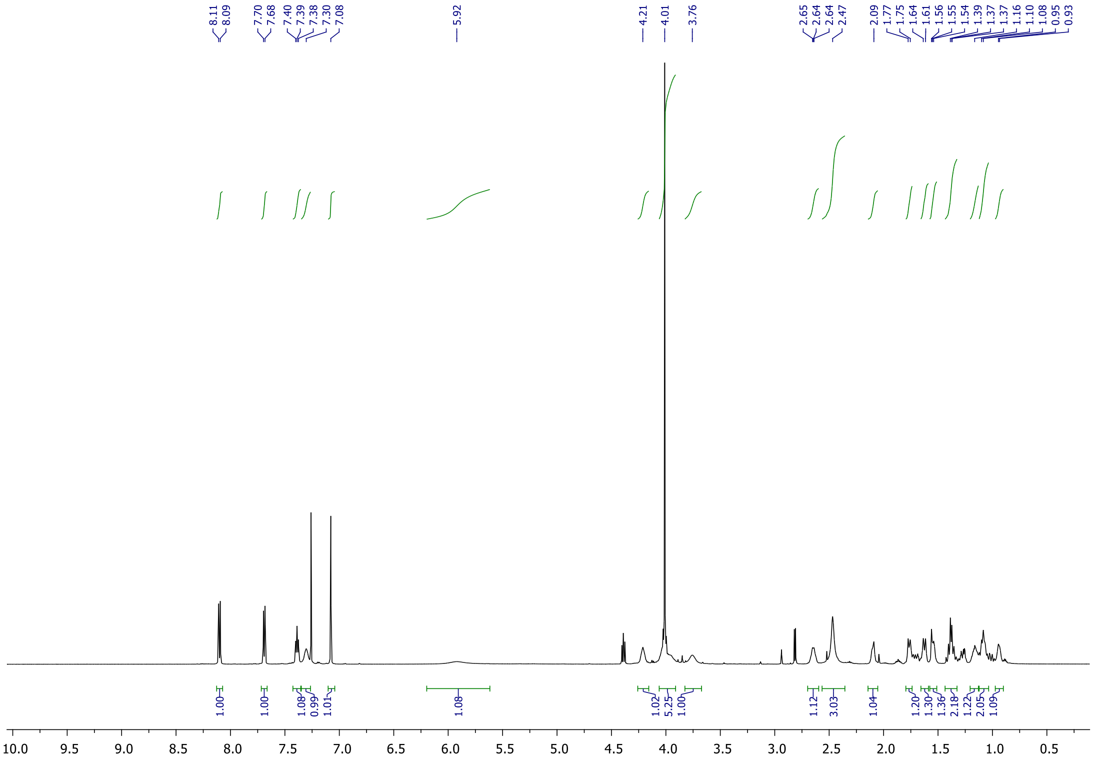

Compound **19l**: ^{13}C NMR

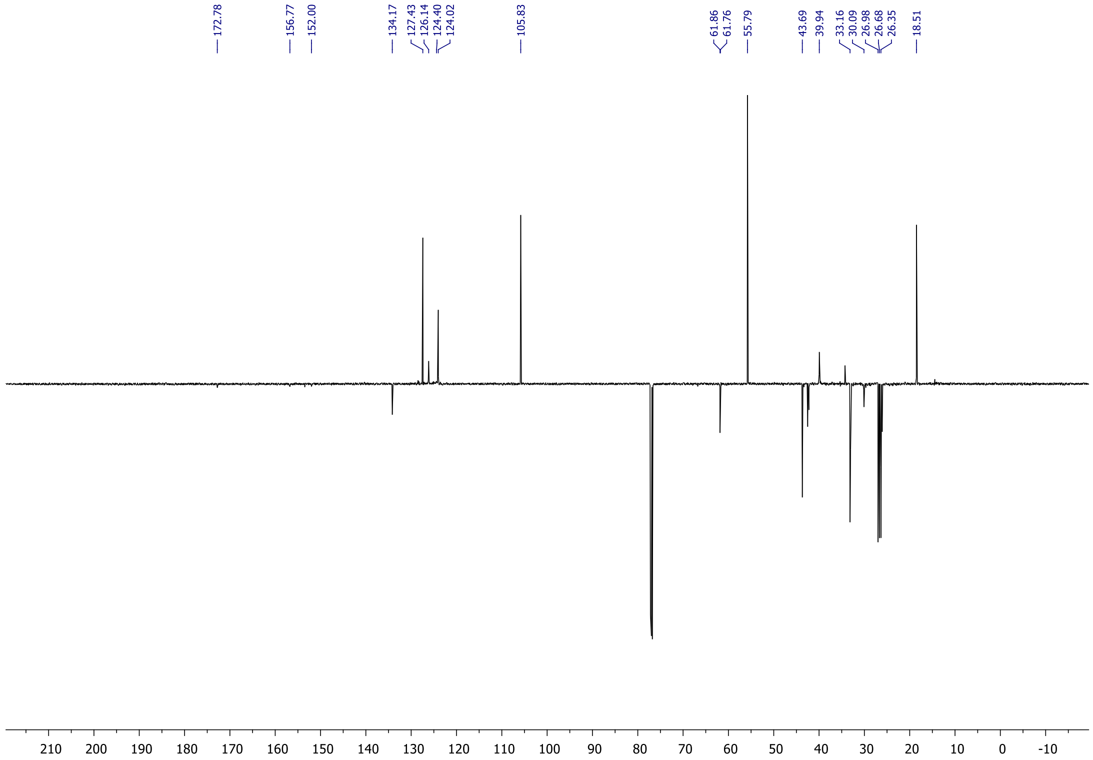

6.HPLC spectra

Compound **18g**-racemic

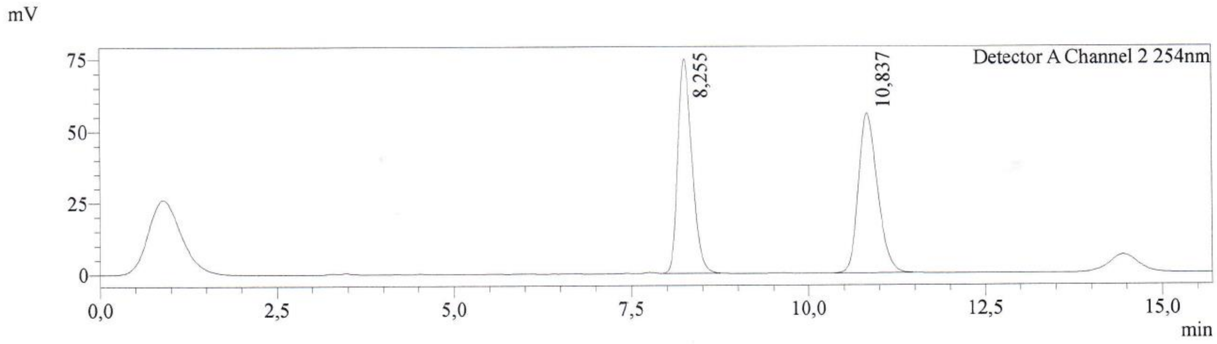

Detector A Channel 2 254nm

Peak#	Ret. Time	Area	Area%
1	8,255	1039539	49,639
2	10,837	1054678	50,361
Total		2094216	100,000

Compound **18g** enantioenriched:

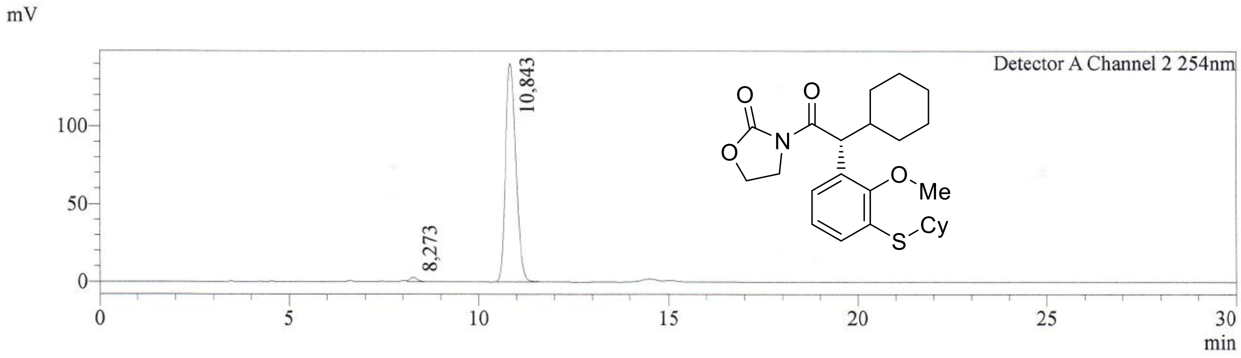

Detector A Channel 2 254nm

Peak#	Ret. Time	Area	Area%
1	8,273	37498	1,405
2	10,843	2630911	98,595
Total		2668409	100,000

Compound **19g**-racemic

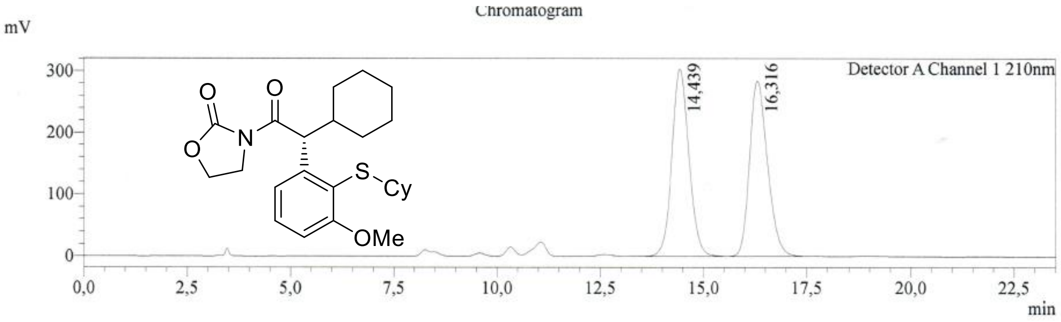

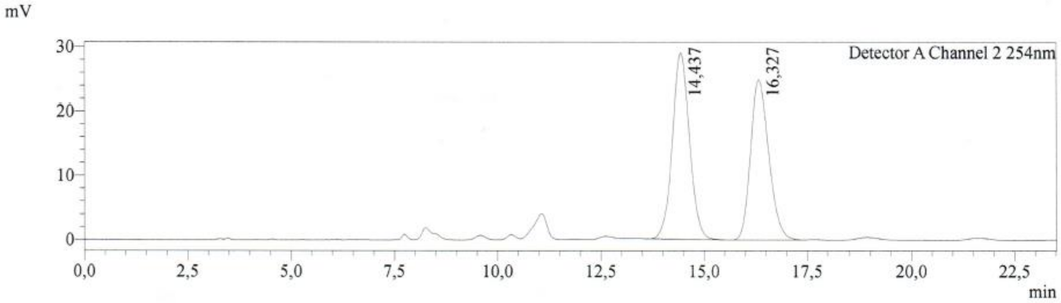

Peak Table

Detector A Channel 1 210nm

Peak#	Ret. Time	Area	Area%
1	14,439	8731118	50,264
2	16,316	8639476	49,736
Total		17370594	100,000

Detector A Channel 2 254nm

Peak#	Ret. Time	Area	Area%
1	14,437	838251	52,132
2	16,327	769684	47,868
Total		1607935	100,000

Compound **19g** enantioenriched:

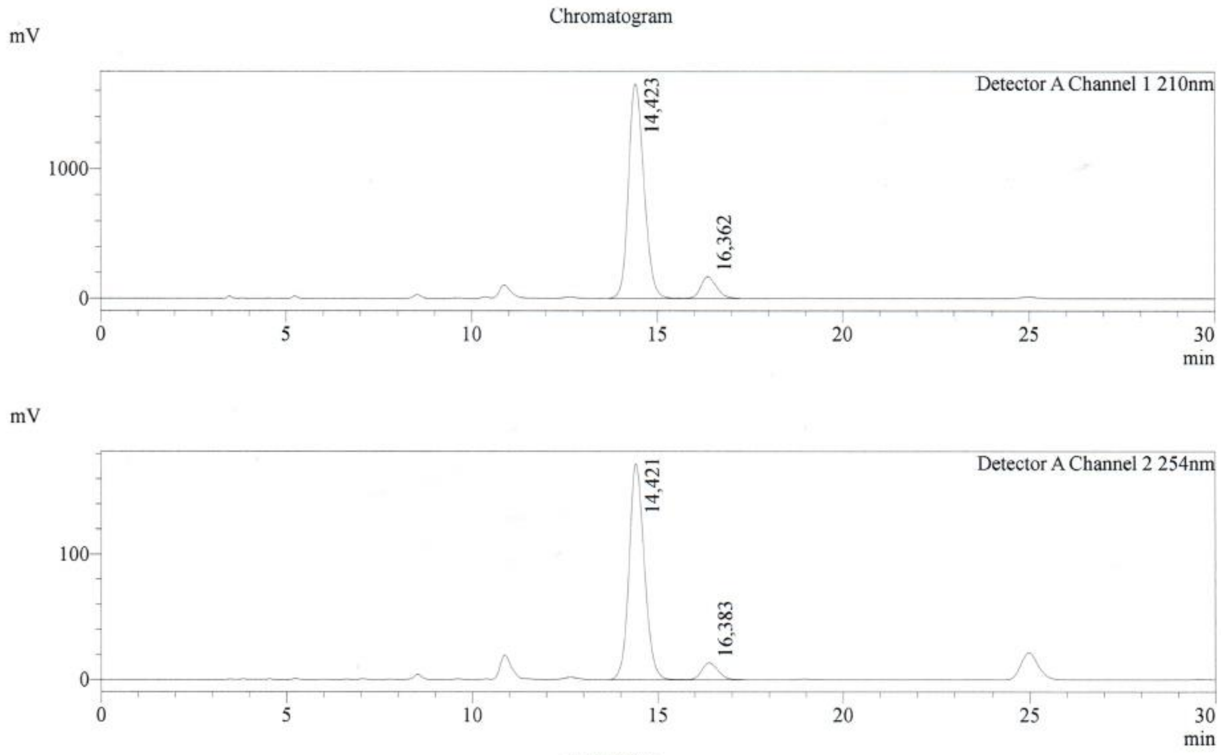

Peak Table

Detector A Channel 1 210nm

Peak#	Ret. Time	Area	Area%
1	14,423	47869698	90,376
2	16,362	5097549	9,624
Total		52967246	100,000

92 : 8

Detector A Channel 2 254nm

Peak#	Ret. Time	Area	Area%
1	14,421	4932506	92,120
2	16,383	421958	7,880
Total		5354464	100,000

7.X-ray crystallographic data for the **18a**, **19g** and **10**

The X-ray intensity data was measured on Bruker D8 Venture diffractometer equipped with multilayer monochromators, Mo K/α INCOATEC micro focus sealed tube, Photon detector and Kryoflex cooling device. The structures were solved by direct methods and refined by full-matrix least-squares techniques. Non-hydrogen atoms were refined with anisotropic displacement parameters. Hydrogen atoms were inserted at calculated positions and refined with a riding model. The following software was used: APEX2 (v2013.6-2)[11] for data collection, cell refinement, data reduction. *SADABS*[12] for absorption correction, *OLEX2*[13] for structure solution, refinement, molecular diagrams and graphical user-interface, *Shelxle*[14] for refinement and graphical user-interface *SHELXS-2013*[15] for structure solution, *SHELXL-2013*[16] for refinement, *Platon*[17] for symmetry check and spacegroup transformation. Experimental data and CCDC-code can be found in Table 1. Crystal data, data collection parameters, and structure refinement details are given in Tables 2 to 5. Molecular structure in "Ortep View" is displayed in Figure 1 and 2.

Table S2. Experimental parameter and CCDC-Code.

Sample	Machine	Source	Temp.	Detector Distance	Time/ Frame	#Frames	Frame width	CCDC
			[K]	[mm]	[s]		[°]	
**18a**	D8	Mo	100.0	34	3	1078	0.6	154080
**19g**	D8	Mo	100.0	34	40	2128	0.5	154080

3-(2-(5-chloro-2-ethoxy-3-(isopropylthio)phenyl)-2-cyclohexylacetyl)oxazolidin-2-one **[18a]**
CCDC1540803

Table S3. Sample and crystal data of [**18a**].

Chemical formula	$C_{22}H_{30}ClNO_4S$	Crystal system	monoclinic
Formula weight [g/mol]	439.98	Space group	P21/n
Temperature [K]	100	Z	4
Measurement method	\f and \w scans	Volume [Å^3]	2181.05(19)

Radiation (Wavelength [Å])	MoKα (λ = 0.71073)	Unit cell dimensions [Å] and [°]	12.0663(6)	90
Crystal size / [mm^3]	0.995 × 0.558 × 0.186		10.1526(5)	103.196(2)
Crystal habit	clear colourless plate		18.2867(10)	90
Density (calculated) / [g/cm^3]	1.34	Absorption coefficient / [mm^{-1}]	0.299	
Abs. correction Tmin	0.3209	Abs. correction Tmax	0.746	
Abs. correction type	multi-scan	F(000) [e$^-$]	936	

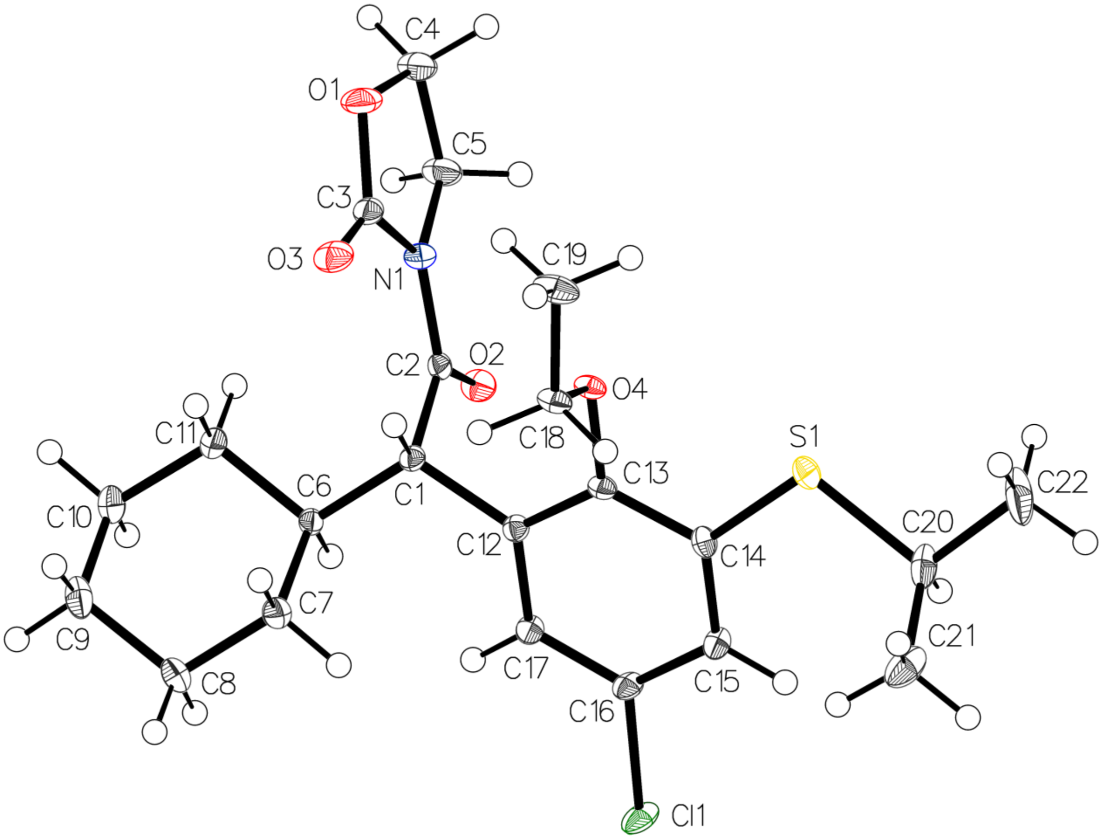

Figure S36. Asymmetric Unit of [**18a**], drawn with 50% displacement ellipsoids. Bond precision: C-C= 0.0017Å. Point group $2_1/n$ determines the chiral centre C1 as R and S. P*$2_1/c$ as alternative* crystallographic setup was detected by Platon.[17]

Table S4. Data collection and structure refinement of [**18a**].

Index ranges	-15 ≤ h ≤ 17, -14 ≤ k ≤ 14, -25 ≤ l ≤ 25	Theta range for data collection [°]	4.57 to 60.166	
Reflections number	63457	Data / restraints / parameters	6407/0/265	
Refinement method	Least squares	Final R indices	all data	R1 = 0.0497, wR2 = 0.0945

Function minimized	$\Sigma\, w(F_o^2 - F_c^2)^2$		I>2σ(I)	R1 = 0.0366, wR2 = 0.0889
Goodness-of-fit on F^2	1.032	Weighting scheme	$w=1/[\sigma^2(F_o^2)+(0.0396P)^2+0.7640P]$	
Largest diff. peak and hole [e Å^{-3}]	0.46/-0.31		where $P=(F_o^2+2F_c^2)/3$	

3-(1-cyclohexyl-2-oxo-2-(2-oxooxazolidin-3-yl)ethyl)-2-(methylthio)phenyl acetate **[19i]** CCDC1540804

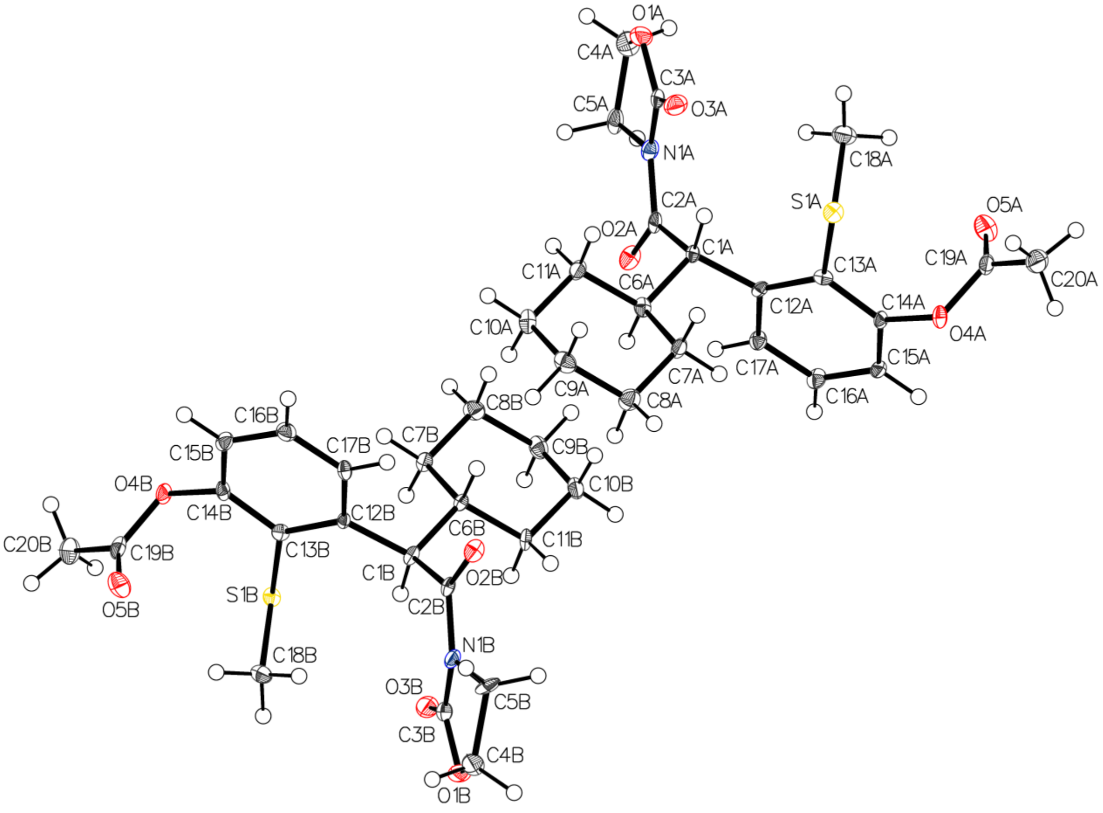

Figure S37. Asymmetric Unit of [**19i**], drawn with 50% displacement ellipsoids. Bond precision: C-C= 0.0059Å. The two independent molecules (A, B) in the asymmetric unit identify the two chiral centres C1A as “S” and C1B as “R”. No obvious change in spacegroup was detected by Platon.[17]

Table S5. Sample and crystal data of [**19i**].

Chemical formula	C20H25NO5S	Crystal system	orthorhombic	
Formula weight [g/mol]	391.48	Space group	Pca21	
Temperature [K]	100	Z	8	
Measurement method	\f and \w scans	Volume [Å^3]	3827.2(7)	
Radiation (Wavelength [Å])	MoKα (λ = 0.71073)	Unit cell dimensions [Å] and [°]	11.7592(13)	90
Crystal size / [mm^3]	0.295 × 0.152 × 0.024		10.1198(10)	90
Crystal habit	clear colourless plate		32.161(3)	90
Density (calculated) / [g/cm^3]	1.359	Absorption coefficient / [mm^{-1}]	0.201	
Abs. correction Tmin	0.6371	Abs. correction Tmax	0.7452	
Abs. correction type	multi-scan	F(000) [e$^-$]	1664	

Table S6. Data collection and structure refinement of [**19i**].

Index ranges	$-14 \leq h \leq 14$, $-12 \leq k \leq 12$, $-38 \leq l \leq 38$	Theta range for data collection [°]	4.756 to 50.76	
Reflections number	100589	Data / restraints / parameters	7014/1/491	
Refinement method	Least squares	Final R indices	all data	R1 = 0.0457, wR2 = 0.1156
Function minimized	$\Sigma\, w(F_o^2 - F_c^2)^2$		$I>2\sigma(I)$	R1 = 0.0445, wR2 = 0.1147
Goodness-of-fit on F^2	1.081	Weighting scheme	$w=1/[\sigma^2(F_o^2)+(0.0716P)^2+3.0440P]$	
Largest diff. peak and hole [e Å^{-3}]	0.75/-0.28		where $P=(F_o^2+2F_c^2)/3$	

3-(2-phenyl-2-(4-(phenylthio)phenyl)acetyl)oxazolidin-2-one (**11**)

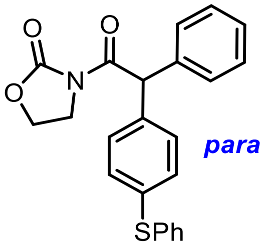

Table S7. Experimental parameter and CCDC-Code.

Sample	Machine	Source	Temp.	Detector Distance	Time/ Frame	#Frames	Frame width	CCDC
	Bruker		[K]	[mm]	[s]		[°]	
11	X8	Mo	130	35	120	1557	0.5	156839

3-(2-phenyl-2-(4-(phenylthio)phenyl)acetyl)oxazolidin-2-one [11]

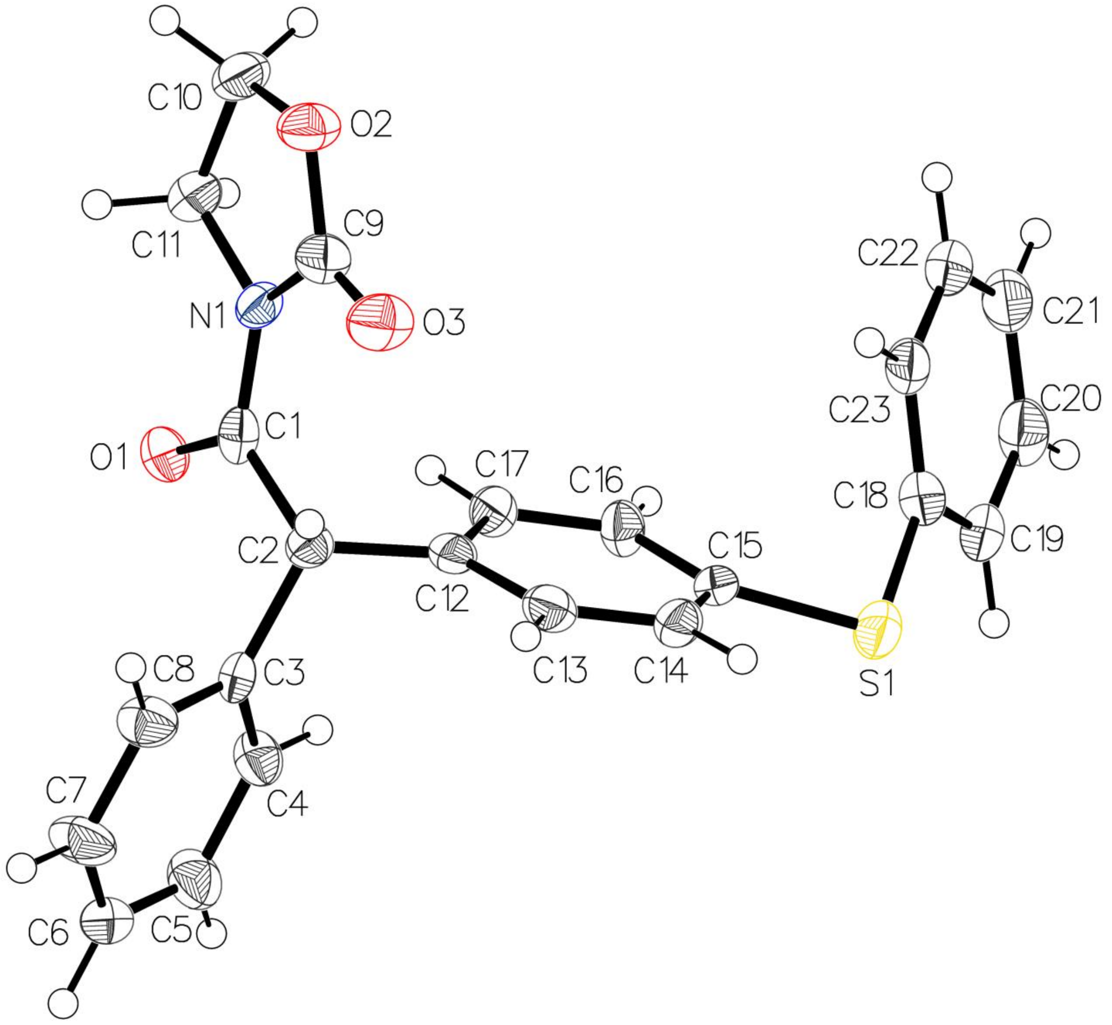

Figure S38. Asymmetric Unit of [**11**], drawn with 50% displacement ellipsoids. Bond Precision on C-C Bonds: 0.00968 Ang.

Table S8. Data collection and structure refinement of [**11**].

Index ranges	-26 ≤ h ≤ 26, -67 ≤ k ≤ 69, -6 ≤ l ≤ 6	Theta range for data collection [°]	4.592 to 50.698
Reflections number	19341	Data / restraints / parameters	3404/1/253

Refinement method	Least squares	Final R indices	all data	R1 = 0.1154, wR2 = 0.1412
Function minimized	Σ w(F_o^2 - F_c^2)^2		I>2σ(I)	R1 = 0.0627, wR2 = 0.1219
Goodness-of-fit on F^2	1.018	Weighting scheme	w=1/[$\sigma^2(F_o^2)+(0.0634P)^2$]	
Largest diff. peak and hole [e Å^{-3}]	0.24/-0.37		where P=(F_o^2+2F_c^2)/3	

Table S9. Sample and crystal data of [**11**].

Chemical formula	C23H19NO3S	Crystal system	orthorhombic	
Formula weight [g/mol]	389.45	Space group	Fdd2	
Temperature [K]	130	Z	16	
Measurement method	\f and \w scans	Volume [Å^3]	7532(8)	
Radiation (Wavelength [Å])	MoKα (λ = 0.71073)	Unit cell dimensions [Å] and [°]	22.362(13)	90
Crystal size / [mm^3]	0.15 × 0.11 × 0.06		58.28(3)	90
Crystal habit	clear colourless block		5.779(4)	90
Density (calculated) / [g/cm^3]	1.374	Absorption coefficient / [mm^{-1}]	0.197	
Abs. correction Tmin	0.5916	Abs. correction Tmax	0.7452	
Abs. correction type	multi-scan	F(000) [e$^-$]	3264	

8.Computational details

8.1.General approaches

The conformational space of all flexible molecules has been initially searched using OPLS_2005 force field[18] and the systematic Monte Carlo conformers search routine implemented in MACROMODEL 11.5.[19]

To consider the flexibility of the complexes with individual fragments (e.g. the complex of a cation with the negatively charged TfO^- counterion), the electrostatic potential of the ions has been studied applying natural bond population analysis (NBO charges). The reciprocal positions of the fragments have been determined based on the calculated charges. The obtained complexes have been used for the additional round of the conformational search to obtain the set of complexes for the subsequent quantum chemical reoptimization.

The structures located at force field level have then been reoptimized at the B3LYP-D3/6-31+G(d,p) level of theory.[20–25] The nature of all stationary points (minima and transition states) was verified through computation of the vibrational frequencies. The thermal corrections to the Gibbs free energy were combined with: a) the energies obtained at the same level of theory to yield DFT Gibbs free energies (G_{298}) at 298.15 K and b) the single point energies calculated at the RI-MP2/def2-TZVP[26] to yield MP2//DFT Gibbs free energies ("G_{298}") at 298.15 K.

To study the regioselectivity of the methoxy-substituted sulfoxides additional quantum chemical methods have been applied for the geometries and the frequencies: 1) DFT approaches: PBE0[27] and M062X,[28] 2) RI-MP2. Additionaly the energies of the DFT-optimized structures have been recalculated at the DLPNO-CCSD(T)/cc-pVTZ level of theory.[29–31] The method comparison is explained in the section 8.2 of the SI in more detail.

All energies are reported in kcal mol^{-1}.

The density-based solvation model SMD[32] (for geometry optimization) and Conductor-like screening model COSMO[33] (for RI-MP2 single-point calculations) were applied to consider solvent effects.

The DFT calculations have been performed with the Gaussian09 program package,[34] whereas for the RI-MP2 calculations the Turbomole V7.2 program package[35] was used. The ORCA 3.0.3 program system was applied for the DLPNO-CCSD(T) computations[36]. Computed structures were visualized using the Chemcraft software.[37]

8.2.Computations to clarify the *meta*-substitution effect

The high dependency of the computational results on the applied level of theory were found for the described system. Various functionals were used for the calculations shown in Fig. 10, in particular

for the problematic intermediate **I_1** and the corresponding transition states **$TS_{G\text{-}I1}$**. Specifically, four families of approaches have been tested:

1) DFT//DFT (B3LYP, M06-2X, PBE0 using 6-31+G(d,p) and def2-TZVP basis sets);

2) DLPNO-CCSD(T)//DFT (DLPNO-CCSD(T)/cc-pVTZ//B3LYP-D3/def2-TZVP);

3) MP2//DFT (RI-MP2/def2-TZVP//B3LYP-D3/def2-TZVP);

4) MP2//MP2 (RI-MP2/def2-SVP; RI-MP2/def2-TZVP; RI-MP2 COSMO/def2-QZVP//RI-MP2/def2-TZVP; SCS-RI-MP2-COSMO/def2-QZVP//RI-MP2/def2-TZVP).[38]

The reason for the method comparison was the initially unexpected observation of the intermediate **I_1** at the B3LYP-D3 level of theory (two basis sets tested: 6-31+G(d,p) and def2-TZVP). In order to verify these results, we applied DLPNO-CCSD(T)//DFT and RI-MP2//DFT approaches. Unfortunately, the results of these composite approaches were unphysical: the free energy of the intermediate **I_1** was found to be ~1.5 kcal mol^{-1} higher as the free energy of the corresponding transition state **$TS_{I1\text{-}I}$**.

The M06-2X and the PBE0-D3 DFT functionals were not able to locate the intermediate **I_1** and the transition state **$TS_{I1\text{-}I}$**. However, these functionals successfully found the transition state **$TS_{G\text{-}I1}$**. The "usual" concerted pathway was also not supported by the M06-2X and PBE0-D3. Thus, these methods failed to describe the system and were also discarded.

Only the MP2//MP2 approaches were in agreement with the B3LYP-D3. Namely the intermediate **I_1** and both transition states **$TS_{I1\text{-}I}$** and **$TS_{G\text{-}I1}$** could be successfully located. In contrast to the MP2//DFT results, the MP2//MP2 showed reasonable energetic characteristics (the Table 4 of the SI)

In the intermediate **I_1** (Figure S39) the distance r(S-O) is 2.279 Å, which is substantially smaller as the sum of the Van Der Waals Radii of the sulfur and oxygen atoms (1.8 (S) + 1.52 (O) = 3.32 Å). Therefore, the intermolecular contact between the fragments within the intermediate **I_1** is very strong, and the sulfur is chiral, as it is shown in Figure S39. Thus, the intermediate **I_1** does not disturb the chirality transfer event. The role of this intermediate is to redistribute the energetic strain of the chirality transfer step between two transition states **$TS_{G\text{-}I1}$** and **$TS_{I1\text{-}H}$**.

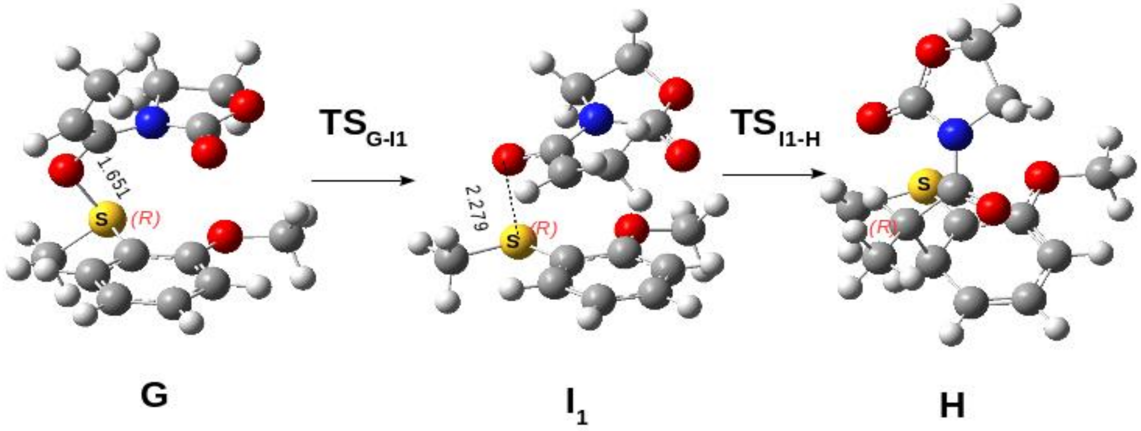

Figure S39. The stepwise mechanism of the chirality transfer event via the intermediate **I$_1$**

Table S10. Relative free energies (kcal mol^{-1}) for localized stationary points (best conformations) at different levels of theory for the X: H (blue) and the X: Cl (red) systems.

	DFT[a]		MP2-1[b]		MP2-2[c]		MP2-3[d]		MP2-4[e]	
	X=H	X=Cl	X=H	X=Cl	X=H	X=Cl	X=H	X=Cl	X=H	X=Cl
**G** + NTf$_2^-$	0.0	0.0	0.0	0.0	0.0	0.0	0.0	0.0	0.0	0.0
**TS$_{G-I1}$** + NTf$_2^-$	4.6	4.1	0.4	0.5	3.5	3.4	4.7	4.8	6.9	7.1
**I$_1$** + NTf$_2^-$	-0.2	0.5	-9.0	-9.1	-2.7	-2.9	-0.9	-0.7	5.2	5.7
**TS$_{I1-I}$** + NTf$_2^-$	4.6	3.5	-5.1	-6.6	4.0	2.6	5.6	4.9	7.4	7.4
**I** + NTf$_2^-$	-11.0	-12.8	-20.9	-25.7	-9.7	-14.2	-10.3	-13.4	-12.5	-15.2
**TS$_{I1-H}$** + NTf$_2^-$	1.5	1.3	-7.7	-6.7	0.4	0.6	2.5	3.0	7.1	8.9
**H** + NTf$_2^-$	-18.1	-16.1	-28.2	-30.4	-17.1	-18.3	-17.1	-18.0	-17.9	-17.7
**TS$_{I-H1}$** + NTf$_2^-$	-2.2	-2.5	-9.7	-13.3	-1.5	-4.1	-1.3	-2.9	0.4	-0.7
**H$_1$** + NTf$_2^-$	-15.8	-14.9	-26.5	-24.7	-14.8	-12.8	-14.4	-13.3	-15.5	-15.0

**22** + NTf_2H	-41.2	-43.6	-137.6	-142.5	-116.1	-120.3	-51.2	-53.8	-51.5	-53.6
**23** + NTf_2H	-41.3	-40.2	-139.7	-142.3	-117.5	-119.5	-52.4	-53.2	-52.3	-52.0

[a]B3LYP-D3-SMD/def2-TZVP; [b]RI-MP2/def2-SVP; [c]RI-MP2/def2-TZVP; [d]RI-MP2-COSMO/def2-QZVP//RI-MP2/def2-TZVP; [e]SCS-RI-MP2-COSMO/def2-QZVP//RI-MP2/def2-TZVP

8.3.Thermochemical data

Table S11. Total energies (Hartree), Gibbs free energies (Hartree), relative free energies (kcal mol^{-1}), the magnitudes of the imaginary frequencies for the transition states (cm^{-1}) for the calculations shown in Fig 6-9.

System	*Etot*	*Gcorr*	*G*	*Imaginary freq.*	*G,rel*	*G,rel*
R=Ph						
**A_1**	**-2528.665397**	**0.329970**	**-2528.335427**	**NO**	**0.00**	
A_2	-2528.665397	0.330018	-2528.335379	NO	0.03	
A_3	-2528.665398	0.330189	-2528.335209	NO	0.14	
A_4	-2528.665398	0.330312	-2528.335086	NO	0.21	
A_5	-2528.665398	0.330438	-2528.334960	NO	0.29	
A_6	-2528.665398	0.330441	-2528.334957	NO	0.30	
A_7	-2528.665398	0.330453	-2528.334945	NO	0.30	
A_8	-2528.665398	0.330706	-2528.334692	NO	0.46	
A_9	-2528.665398	0.330751	-2528.334647	NO	0.49	
A_10	-2528.665398	0.330780	-2528.334618	NO	0.51	
A_11	-2528.665397	0.330803	-2528.334594	NO	0.52	
A_12	-2528.665397	0.330806	-2528.334591	NO	0.52	
A_13	-2528.664344	0.331487	-2528.332857	NO	1.61	
A_14	-2528.664344	0.331513	-2528.332831	NO	1.63	

A_15	-2528.664344	0.331513	-2528.332831	NO	1.63	
A_16	-2528.664345	0.331520	-2528.332825	NO	1.63	
A_17	-2528.663773	0.330957	-2528.332816	NO	1.64	
A_18	-2528.664344	0.331537	-2528.332807	NO	1.64	
A_19	-2528.663370	0.330745	-2528.332625	NO	1.76	
A_20	-2528.663371	0.330754	-2528.332617	NO	1.76	
A_21	-2528.663641	0.331363	-2528.332278	NO	1.98	
A_22	-2528.663297	0.331049	-2528.332248	NO	2.00	
A_23	-2528.662719	0.330597	-2528.332122	NO	2.07	
A_24	-2528.658438	0.326376	-2528.332062	NO	2.11	
A_25	-2528.663298	0.331343	-2528.331955	NO	2.18	
A_26	-2528.662908	0.331091	-2528.331817	NO	2.27	
A_27	-2528.663536	0.331812	-2528.331724	NO	2.32	
A_28	-2528.665397	0.333725	-2528.331672	NO	2.36	
A_29	-2528.665398	0.333730	-2528.331668	NO	2.36	
A_30	-2528.665398	0.333732	-2528.331666	NO	2.36	
A_31	-2528.662405	0.330815	-2528.331590	NO	2.41	
A_32	-2528.663099	0.331800	-2528.331299	NO	2.59	
A_33	-2528.663099	0.331829	-2528.331270	NO	2.61	
A_34	-2528.663302	0.332113	-2528.331189	NO	2.66	
A_35	-2528.663301	0.332113	-2528.331188	NO	2.66	
A_36	-2528.663301	0.332115	-2528.331186	NO	2.66	
A_37	-2528.663012	0.331841	-2528.331171	NO	2.67	
A_38	-2528.663099	0.331929	-2528.331170	NO	2.67	

A_39	-2528.663689	0.332521	-2528.331168	NO	2.67	
A_40	-2528.663301	0.332134	-2528.331167	NO	2.67	
A_41	-2528.662540	0.331375	-2528.331165	NO	2.67	
A_42	-2528.663013	0.331873	-2528.331140	NO	2.69	
A_43	-2528.663012	0.331896	-2528.331116	NO	2.71	
A_44	-2528.661014	0.329908	-2528.331106	NO	2.71	
A_45	-2528.663287	0.332207	-2528.331080	NO	2.73	
A_46	-2528.662825	0.331795	-2528.331030	NO	2.76	
A_47	-2528.662827	0.331802	-2528.331025	NO	2.76	
A_48	-2528.662161	0.331160	-2528.331001	NO	2.78	
A_49	-2528.662430	0.331485	-2528.330945	NO	2.81	
A_50	-2528.659098	0.328204	-2528.330894	NO	2.84	
A_51	-2528.661025	0.330237	-2528.330788	NO	2.91	
A_52	-2528.663267	0.332540	-2528.330727	NO	2.95	
A_53	-2528.663267	0.332549	-2528.330718	NO	2.96	
A_54	-2528.662663	0.331979	-2528.330684	NO	2.98	
A_55	-2528.660027	0.329484	-2528.330543	NO	3.07	
A_56	-2528.661194	0.330927	-2528.330267	NO	3.24	
A_57	-2528.663249	0.332995	-2528.330254	NO	3.25	
A_58	-2528.660780	0.330570	-2528.330210	NO	3.27	
A_59	-2528.659840	0.329660	-2528.330180	NO	3.29	
A_60	-2528.660489	0.330343	-2528.330146	NO	3.31	
A_61	-2528.661912	0.331868	-2528.330044	NO	3.38	
A_62	-2528.660210	0.330220	-2528.329990	NO	3.41	

A_63	-2528.659496	0.329568	-2528.329928	NO	3.45	
A_64	-2528.659496	0.329602	-2528.329894	NO	3.47	
A_65	-2528.659775	0.329914	-2528.329861	NO	3.49	
A_66	-2528.660213	0.330475	-2528.329738	NO	3.57	
A_67	-2528.663232	0.333531	-2528.329701	NO	3.59	
A_68	-2528.662513	0.332812	-2528.329701	NO	3.59	
A_69	-2528.659400	0.329820	-2528.329580	NO	3.67	
A_70	-2528.663848	0.334270	-2528.329578	NO	3.67	
A_71	-2528.660792	0.331274	-2528.329518	NO	3.71	
A_72	-2528.659094	0.329649	-2528.329445	NO	3.75	
A_73	-2528.660761	0.331357	-2528.329404	NO	3.78	
A_74	-2528.660170	0.330788	-2528.329382	NO	3.79	
A_75	-2528.657817	0.328475	-2528.329342	NO	3.82	
A_76	-2528.660534	0.331193	-2528.329341	NO	3.82	
A_77	-2528.659676	0.330350	-2528.329326	NO	3.83	
A_78	-2528.662928	0.333605	-2528.329323	NO	3.83	
A_79	-2528.662302	0.332988	-2528.329314	NO	3.84	
A_80	-2528.660813	0.331512	-2528.329301	NO	3.84	
A_81	-2528.660826	0.331537	-2528.329289	NO	3.85	
A_82	-2528.658659	0.329390	-2528.329269	NO	3.86	
A_83	-2528.660436	0.331173	-2528.329263	NO	3.87	
A_84	-2528.660491	0.331321	-2528.329170	NO	3.93	
A_85	-2528.659661	0.330515	-2528.329146	NO	3.94	
A_86	-2528.660871	0.331749	-2528.329122	NO	3.96	

A_87	-2528.657690	0.328571	-2528.329119	NO	3.96	
A_88	-2528.660064	0.330954	-2528.329110	NO	3.96	
A_89	-2528.659648	0.330561	-2528.329087	NO	3.98	
A_90	-2528.659649	0.330601	-2528.329048	NO	4.00	
A_91	-2528.659783	0.330798	-2528.328985	NO	4.04	
A_92	-2528.658374	0.329451	-2528.328923	NO	4.08	
A_93	-2528.659790	0.330873	-2528.328917	NO	4.09	
A_94	-2528.661192	0.332377	-2528.328815	NO	4.15	
A_95	-2528.660188	0.331435	-2528.328753	NO	4.19	
A_96	-2528.661742	0.333028	-2528.328714	NO	4.21	
A_97	-2528.661742	0.333029	-2528.328713	NO	4.21	
A_98	-2528.658744	0.330035	-2528.328709	NO	4.22	
A_99	-2528.661201	0.332509	-2528.328692	NO	4.23	
A_100	-2528.660825	0.332145	-2528.328680	NO	4.23	
A_101	-2528.661201	0.332521	-2528.328680	NO	4.23	
A_102	-2528.660220	0.331580	-2528.328640	NO	4.26	
A_103	-2528.661873	0.333254	-2528.328619	NO	4.27	
A_104	-2528.659737	0.331120	-2528.328617	NO	4.27	
A_105	-2528.658744	0.330134	-2528.328610	NO	4.28	
A_106	-2528.659825	0.331221	-2528.328604	NO	4.28	
A_107	-2528.658821	0.330221	-2528.328600	NO	4.28	
A_108	-2528.661874	0.333300	-2528.328574	NO	4.30	
A_109	-2528.657169	0.328599	-2528.328570	NO	4.30	
A_110	-2528.659825	0.331257	-2528.328568	NO	4.30	

A_111	-2528.659043	0.330488	-2528.328555	NO	4.31	
A_112	-2528.659825	0.331276	-2528.328549	NO	4.32	
A_113	-2528.659743	0.331207	-2528.328536	NO	4.32	
A_114	-2528.659796	0.331295	-2528.328501	NO	4.35	
A_115	-2528.659068	0.330597	-2528.328471	NO	4.37	
A_116	-2528.659972	0.331534	-2528.328438	NO	4.39	
A_117	-2528.658992	0.330557	-2528.328435	NO	4.39	
A_118	-2528.659101	0.330673	-2528.328428	NO	4.39	
A_119	-2528.660658	0.332231	-2528.328427	NO	4.39	
A_120	-2528.660658	0.332233	-2528.328425	NO	4.39	
A_121	-2528.657922	0.329502	-2528.328420	NO	4.40	
A_122	-2528.659161	0.330782	-2528.328379	NO	4.42	
A_123	-2528.655974	0.327833	-2528.328141	NO	4.57	
A_124	-2528.658943	0.330845	-2528.328098	NO	4.60	
A_125	-2528.658737	0.330733	-2528.328004	NO	4.66	
A_126	-2528.659231	0.331251	-2528.327980	NO	4.67	
A_127	-2528.657783	0.329822	-2528.327961	NO	4.69	
A_128	-2528.660181	0.332236	-2528.327945	NO	4.70	
A_129	-2528.660161	0.332225	-2528.327936	NO	4.70	
A_130	-2528.660558	0.332661	-2528.327897	NO	4.73	
A_131	-2528.659048	0.331164	-2528.327884	NO	4.73	
A_132	-2528.658701	0.330820	-2528.327881	NO	4.74	
A_133	-2528.659480	0.331684	-2528.327796	NO	4.79	
A_134	-2528.658214	0.330419	-2528.327795	NO	4.79	

A_135	-2528.658701	0.330925	-2528.327776	NO	4.80	
A_136	-2528.658060	0.330420	-2528.327640	NO	4.89	
A_137	-2528.661455	0.333833	-2528.327622	NO	4.90	
A_138	-2528.659222	0.331605	-2528.327617	NO	4.90	
A_139	-2528.660036	0.332491	-2528.327545	NO	4.95	
A_140	-2528.658677	0.331145	-2528.327532	NO	4.95	
A_141	-2528.657534	0.330035	-2528.327499	NO	4.97	
A_142	-2528.658400	0.330908	-2528.327492	NO	4.98	
A_143	-2528.658063	0.330613	-2528.327450	NO	5.01	
A_144	-2528.659500	0.332066	-2528.327434	NO	5.02	
A_145	-2528.659048	0.331617	-2528.327431	NO	5.02	
A_146	-2528.658924	0.331535	-2528.327389	NO	5.04	
A_147	-2528.660501	0.333196	-2528.327305	NO	5.10	
A_148	-2528.658773	0.331503	-2528.327270	NO	5.12	
A_149	-2528.659970	0.332761	-2528.327209	NO	5.16	
A_150	-2528.659970	0.332767	-2528.327203	NO	5.16	
A_151	-2528.659051	0.331912	-2528.327139	NO	5.20	
A_152	-2528.658372	0.331337	-2528.327035	NO	5.27	
A_153	-2528.658372	0.331362	-2528.327010	NO	5.28	
A_154	-2528.659877	0.332943	-2528.326934	NO	5.33	
A_155	-2528.659200	0.332281	-2528.326919	NO	5.34	
A_156	-2528.659051	0.332160	-2528.326891	NO	5.36	
A_157	-2528.660189	0.333353	-2528.326836	NO	5.39	
A_158	-2528.659596	0.332763	-2528.326833	NO	5.39	

A_159	-2528.659039	0.332260	-2528.326779	NO	5.43	
A_160	-2528.660171	0.333493	-2528.326678	NO	5.49	
A_161	-2528.657976	0.331311	-2528.326665	NO	5.50	
A_162	-2528.660239	0.333622	-2528.326617	NO	5.53	
A_163	-2528.660653	0.334036	-2528.326617	NO	5.53	
A_164	-2528.661203	0.334640	-2528.326563	NO	5.56	
A_165	-2528.657402	0.330849	-2528.326553	NO	5.57	
A_166	-2528.659581	0.333030	-2528.326551	NO	5.57	
A_167	-2528.658794	0.332287	-2528.326507	NO	5.60	
A_168	-2528.659085	0.332615	-2528.326470	NO	5.62	
A_169	-2528.658795	0.332327	-2528.326468	NO	5.62	
A_170	-2528.658828	0.332481	-2528.326347	NO	5.70	
A_171	-2528.655707	0.329365	-2528.326342	NO	5.70	
A_172	-2528.658080	0.331743	-2528.326337	NO	5.70	
A_173	-2528.660751	0.334429	-2528.326322	NO	5.71	
A_174	-2528.657442	0.331152	-2528.326290	NO	5.73	
A_175	-2528.657156	0.330910	-2528.326246	NO	5.76	
A_176	-2528.659409	0.333164	-2528.326245	NO	5.76	
A_177	-2528.657635	0.331446	-2528.326189	NO	5.80	
A_178	-2528.657161	0.330976	-2528.326185	NO	5.80	
A_179	-2528.657988	0.331842	-2528.326146	NO	5.82	
A_180	-2528.658464	0.332376	-2528.326088	NO	5.86	
A_181	-2528.659542	0.333512	-2528.326030	NO	5.90	
A_182	-2528.658750	0.332744	-2528.326006	NO	5.91	

A_183	-2528.657039	0.331057	-2528.325982	NO	5.93	
A_184	-2528.657586	0.331681	-2528.325905	NO	5.98	
A_185	-2528.658173	0.332582	-2528.325591	NO	6.17	
A_186	-2528.657326	0.331744	-2528.325582	NO	6.18	
A_187	-2528.656602	0.331076	-2528.325526	NO	6.21	
A_188	-2528.659770	0.334420	-2528.325350	NO	6.32	
A_189	-2528.658531	0.333234	-2528.325297	NO	6.36	
A_190	-2528.658547	0.333305	-2528.325242	NO	6.39	
A_191	-2528.655094	0.329929	-2528.325165	NO	6.44	
A_192	-2528.658166	0.333090	-2528.325076	NO	6.50	
A_193	-2528.653709	0.328657	-2528.325052	NO	6.51	
A_194	-2528.658166	0.333142	-2528.325024	NO	6.53	
A_195	-2528.657195	0.332179	-2528.325016	NO	6.53	
A_196	-2528.657196	0.332210	-2528.324986	NO	6.55	
A_197	-2528.657934	0.332949	-2528.324985	NO	6.55	
A_198	-2528.657230	0.332270	-2528.324960	NO	6.57	
A_199	-2528.657581	0.332647	-2528.324934	NO	6.58	
A_200	-2528.656280	0.331450	-2528.324830	NO	6.65	
A_201	-2528.658203	0.333640	-2528.324563	NO	6.82	
A_202	-2528.655226	0.330791	-2528.324435	NO	6.90	
A_203	-2528.655226	0.330813	-2528.324413	NO	6.91	
A_204	-2528.657004	0.332690	-2528.324314	NO	6.97	
A_205	-2528.657021	0.332735	-2528.324286	NO	6.99	
A_206	-2528.656109	0.331914	-2528.324195	NO	7.05	

A_207	-2528.656710	0.332711	-2528.323999	NO	7.17	
A_208	-2528.657772	0.333802	-2528.323970	NO	7.19	
A_209	-2528.653587	0.329687	-2528.323900	NO	7.23	
A_210	-2528.654229	0.330457	-2528.323772	NO	7.31	
A_211	-2528.658043	0.334622	-2528.323421	NO	7.53	
A_212	-2528.658042	0.334682	-2528.323360	NO	7.57	
A_213	-2528.656671	0.333551	-2528.323120	NO	7.72	
A_214	-2528.653592	0.330697	-2528.322895	NO	7.86	
A_215	-2528.655405	0.332712	-2528.322693	NO	7.99	
A_216	-2528.655188	0.333196	-2528.321992	NO	8.43	
A_217	-2528.655579	0.334091	-2528.321488	NO	8.75	
A_218	-2528.646809	0.328968	-2528.317841	NO	11.04	
A_219	-2528.646556	0.329421	-2528.317135	NO	11.48	
A_220	-2528.646556	0.329430	-2528.317126	NO	11.48	
A_221	-2528.647110	0.330452	-2528.316658	NO	11.78	
A_222	-2528.645669	0.329054	-2528.316615	NO	11.80	
A_223	-2528.645669	0.329130	-2528.316539	NO	11.85	
A_224	-2528.646042	0.330278	-2528.315764	NO	12.34	
A_225	-2528.646921	0.331633	-2528.315288	NO	12.64	
A_226	-2528.643775	0.331709	-2528.312066	NO	14.66	
A_227	-2528.643522	0.331832	-2528.311690	NO	14.90	
A_228	-2528.642003	0.330556	-2528.311447	NO	15.05	
A_229	-2528.643472	0.332341	-2528.311131	NO	15.25	

**TS_A-B_1**	**-2528.631242**	**0.324604**	**-2528.306638**	**-894.154800**	**18.07**	
TS_A-B_2	-2528.631296	0.325139	-2528.306157	-881.486800	18.37	
TS_A-B_3	-2528.631382	0.326182	-2528.305200	-802.486800	18.97	
TS_A-B_4	-2528.632473	0.327324	-2528.305149	-1090.493600	19.00	
TS_A-B_5	-2528.631381	0.326561	-2528.304820	-802.469700	19.21	
TS_A-B_6	-2528.632881	0.328189	-2528.304692	-1029.725700	19.29	
TS_A-B_7	-2528.632881	0.328193	-2528.304688	-1029.855100	19.29	
TS_A-B_8	-2528.632385	0.328458	-2528.303927	-1026.749900	19.77	
TS_A-B_9	-2528.632558	0.328717	-2528.303841	-1044.355800	19.82	
TS_A-B_10	-2528.630188	0.326375	-2528.303813	-805.924500	19.84	
TS_A-B_11	-2528.632809	0.329174	-2528.303635	-1096.267900	19.95	
TS_A-B_12	-2528.629938	0.326440	-2528.303498	-1179.662400	20.04	
TS_A-B_13	-2528.633228	0.330732	-2528.302496	-983.470600	20.66	
**B_1**	**-2528.654046**	**0.331277**	**-2528.322769**	**NO**	**7.94**	
B_2	-2528.654035	0.331279	-2528.322756	NO	7.95	
B_3	-2528.654046	0.331301	-2528.322745	NO	7.96	
B_4	-2528.652702	0.330022	-2528.322680	NO	8.00	
B_5	-2528.654384	0.331979	-2528.322405	NO	8.17	
B_6	-2528.654384	0.331988	-2528.322396	NO	8.18	
B_7	-2528.652724	0.330337	-2528.322387	NO	8.18	
B_8	-2528.654383	0.332027	-2528.322356	NO	8.20	
B_9	-2528.652178	0.329836	-2528.322342	NO	8.21	
B_10	-2528.653221	0.330945	-2528.322276	NO	8.25	

B_11	-2528.653221	0.330946	-2528.322275	NO	8.25	
B_12	-2528.654416	0.332184	-2528.322232	NO	8.28	
B_13	-2528.654416	0.332193	-2528.322223	NO	8.29	
B_14	-2528.654416	0.332196	-2528.322220	NO	8.29	
B_15	-2528.654416	0.332220	-2528.322196	NO	8.30	
B_16	-2528.652869	0.331010	-2528.321859	NO	8.51	
B_17	-2528.652189	0.330445	-2528.321744	NO	8.59	
B_18	-2528.652391	0.330773	-2528.321618	NO	8.67	
B_19	-2528.652397	0.330858	-2528.321539	NO	8.71	
B_20	-2528.652429	0.331199	-2528.321230	NO	8.91	
B_21	-2528.652901	0.331909	-2528.320992	NO	9.06	
B_22	-2528.652900	0.332034	-2528.320866	NO	9.14	
B_23	-2528.650979	0.330491	-2528.320488	NO	9.37	
B_24	-2528.650604	0.330394	-2528.320210	NO	9.55	
B_25	-2528.651819	0.331779	-2528.320040	NO	9.66	
B_26	-2528.647953	0.327919	-2528.320034	NO	9.66	
B_27	-2528.650961	0.331026	-2528.319935	NO	9.72	
B_28	-2528.651360	0.331629	-2528.319731	NO	9.85	
B_29	-2528.651040	0.331332	-2528.319708	NO	9.86	
B_30	-2528.651674	0.332192	-2528.319482	NO	10.01	
B_31	-2528.651728	0.332320	-2528.319408	NO	10.05	
B_32	-2528.651277	0.331951	-2528.319326	NO	10.10	
B_33	-2528.651468	0.332192	-2528.319276	NO	10.14	
B_34	-2528.652677	0.333528	-2528.319149	NO	10.21	

B_35	-2528.650128	0.331112	-2528.319016	NO	10.30	
B_36	-2528.650484	0.331469	-2528.319015	NO	10.30	
B_37	-2528.652526	0.333516	-2528.319010	NO	10.30	
B_38	-2528.651225	0.332219	-2528.319006	NO	10.30	
B_39	-2528.650128	0.331207	-2528.318921	NO	10.36	
B_40	-2528.647764	0.329156	-2528.318608	NO	10.55	
B_41	-2528.652445	0.333847	-2528.318598	NO	10.56	
B_42	-2528.649869	0.331549	-2528.318320	NO	10.73	
B_43	-2528.652310	0.334053	-2528.318257	NO	10.77	
B_44	-2528.652369	0.334345	-2528.318024	NO	10.92	
B_45	-2528.651340	0.333317	-2528.318023	NO	10.92	
B_46	-2528.649789	0.331812	-2528.317977	NO	10.95	
B_47	-2528.649052	0.331188	-2528.317864	NO	11.02	
B_48	-2528.649958	0.332242	-2528.317716	NO	11.11	
B_49	-2528.649966	0.332970	-2528.316996	NO	11.57	
B_50	-2528.649825	0.332905	-2528.316920	NO	11.61	
B_51	-2528.648460	0.331641	-2528.316819	NO	11.68	
B_52	-2528.650301	0.333544	-2528.316757	NO	11.72	
B_53	-2528.649805	0.333161	-2528.316644	NO	11.79	
B_54	-2528.647869	0.331602	-2528.316267	NO	12.02	
B_55	-2528.648977	0.332746	-2528.316231	NO	12.05	
B_56	-2528.648991	0.333087	-2528.315904	NO	12.25	
B_57	-2528.648359	0.332601	-2528.315758	NO	12.34	
B_58	-2528.648355	0.332832	-2528.315523	NO	12.49	

B_59	-2528.648787	0.334040	-2528.314747	NO	12.98	
B_60	-2528.649041	0.336306	-2528.312735	NO	14.24	
B_61	-2528.646399	0.333843	-2528.312556	NO	14.35	
**TS_B-C_1**	**-2528.648804**	**0.329669**	**-2528.319135**	**-33.256000**	**10.22**	
TS_B-C_2	-2528.648804	0.329941	-2528.318863	-33.248800	10.39	
TS_B-C_3	-2528.649006	0.330631	-2528.318375	-29.171500	10.70	
TS_B-C_4	-2528.649049	0.331831	-2528.317218	-8.741300	11.43	
TS_B-C_5	-2528.649048	0.331831	-2528.317217	-8.595500	11.43	
TS_B-C_6	-2528.649048	0.331842	-2528.317206	-8.629200	11.43	
TS_B-C_7	-2528.648804	0.333447	-2528.315357	-32.954800	12.59	
**C_1**	**-2528.687014**	**0.334975**	**-2528.352039**	**NO**	**-10.42**	
C_2	-2528.686851	0.335031	-2528.351820	NO	-10.29	
C_3	-2528.686852	0.335053	-2528.351799	NO	-10.27	
C_4	-2528.688959	0.337239	-2528.351720	NO	-10.22	
C_5	-2528.688718	0.337196	-2528.351522	NO	-10.10	
C_6	-2528.688718	0.337207	-2528.351511	NO	-10.09	
C_7	-2528.688718	0.337214	-2528.351504	NO	-10.09	
C_8	-2528.685612	0.334238	-2528.351374	NO	-10.01	
C_9	-2528.687322	0.336321	-2528.351001	NO	-9.77	
C_10	-2528.689121	0.338279	-2528.350842	NO	-9.67	
C_11	-2528.687195	0.336459	-2528.350736	NO	-9.61	
C_12	-2528.687196	0.336488	-2528.350708	NO	-9.59	

C_13	-2528.687252	0.336617	-2528.350635	NO	-9.54	
C_14	-2528.686173	0.338485	-2528.347688	NO	-7.69	
**TS_C-D_1**	**-2528.687581**	**0.336309**	**-2528.351272**	**-179.409300**	**-9.94**	
TS_C-D_2	-2528.687581	0.336313	-2528.351268	-179.438400	-9.94	
TS_C-D_3	-2528.687321	0.336181	-2528.351140	-148.095700	-9.86	
TS_C-D_4	-2528.687322	0.336199	-2528.351123	-148.280500	-9.85	
TS_C-D_5	-2528.688367	0.337313	-2528.351054	-198.589600	-9.81	
TS_C-D_6	-2528.688367	0.337318	-2528.351049	-198.568300	-9.80	
TS_C-D_7	-2528.688367	0.337321	-2528.351046	-198.498600	-9.80	
TS_C-D_8	-2528.686389	0.335817	-2528.350572	-144.537300	-9.50	
**D_1**	**-2528.734296**	**0.334888**	**-2528.399408**	**NO**	**-40.15**	**0.00**
D_2	-2528.735775	0.339023	-2528.396752		-38.48	
D_3	-2528.735774	0.339034	-2528.396740		-38.47	
D_4	-2528.735656	0.339380	-2528.396276		-38.18	
D_5	-2528.735584	0.339604	-2528.395980		-38.00	
D_6	-2528.732563	0.339487	-2528.393076		-36.18	
D_7	-2528.730392	0.337916	-2528.392476		-35.80	
D_8	-2528.730116	0.340360	-2528.389756		-34.09	
**TS_D-F_1**	**-2528.732417**	**0.333627**	**-2528.398790**	**-676.282200**	**-39.76**	**0.39**
TS_D-F_2	-2528.732750	0.333983	-2528.398767	-576.496700	-39.75	0.40
TS_D-F_3	-2528.729138	0.336278	-2528.392860	-762.160600	-36.04	4.11

TS_D-F_4	-2528.728669	0.335956	-2528.392713	-743.988400	-35.95	4.20
**F_1**	**-2528.774097**	**0.337754**	**-2528.436343**	**NO**	**-63.33**	**-23.18**
F_2	-2528.774876	0.339771	-2528.435105	NO	-62.55	-22.40
F_3	-2528.774507	0.339761	-2528.434746	NO	-62.32	-22.18
F_4	-2528.774431	0.339713	-2528.434718	NO	-62.31	-22.16
F_5	-2528.774669	0.340041	-2528.434628	NO	-62.25	-22.10
F_6	-2528.774669	0.340048	-2528.434621	NO	-62.24	-22.10
F_7	-2528.773081	0.338641	-2528.434440	NO	-62.13	-21.98
F_8	-2528.773397	0.339068	-2528.434329	NO	-62.06	-21.91
F_9	-2528.773398	0.339095	-2528.434303	NO	-62.05	-21.90
F_10	-2528.774567	0.340304	-2528.434263	NO	-62.02	-21.87
F_11	-2528.772985	0.338822	-2528.434163	NO	-61.96	-21.81
F_12	-2528.773823	0.339721	-2528.434102	NO	-61.92	-21.77
F_13	-2528.773823	0.339813	-2528.434010	NO	-61.86	-21.71
F_14	-2528.773868	0.339903	-2528.433965	NO	-61.83	-21.69
F_15	-2528.773868	0.339903	-2528.433965	NO	-61.83	-21.69
F_16	-2528.775945	0.342169	-2528.433776	NO	-61.71	-21.57
F_17	-2528.775943	0.342178	-2528.433765	NO	-61.71	-21.56
F_18	-2528.775943	0.342182	-2528.433761	NO	-61.71	-21.56
F_19	-2528.773931	0.340210	-2528.433721	NO	-61.68	-21.53
F_20	-2528.773932	0.340227	-2528.433705	NO	-61.67	-21.52
F_21	-2528.771146	0.337535	-2528.433611	NO	-61.61	-21.46
F_22	-2528.773159	0.339608	-2528.433551	NO	-61.57	-21.43

F_23	-2528.774178	0.340708	-2528.433470	NO	-61.52	-21.37
F_24	-2528.773494	0.340137	-2528.433357	NO	-61.45	-21.30
F_25	-2528.772274	0.339118	-2528.433156	NO	-61.33	-21.18
F_26	-2528.770616	0.337561	-2528.433055	NO	-61.26	-21.11
F_27	-2528.773559	0.340525	-2528.433034	NO	-61.25	-21.10
F_28	-2528.773548	0.340571	-2528.432977	NO	-61.21	-21.06
F_29	-2528.773721	0.340842	-2528.432879	NO	-61.15	-21.00
F_30	-2528.772247	0.339535	-2528.432712	NO	-61.05	-20.90
F_31	-2528.773492	0.340954	-2528.432538	NO	-60.94	-20.79
F_32	-2528.771037	0.338580	-2528.432457	NO	-60.89	-20.74
F_33	-2528.771441	0.339105	-2528.432336	NO	-60.81	-20.66
F_34	-2528.773332	0.341186	-2528.432146	NO	-60.69	-20.54
F_35	-2528.772646	0.340601	-2528.432045	NO	-60.63	-20.48
F_36	-2528.771069	0.339141	-2528.431928	NO	-60.55	-20.41
F_37	-2528.772272	0.340568	-2528.431704	NO	-60.41	-20.27
F_38	-2528.769372	0.338077	-2528.431295	NO	-60.16	-20.01
F_39	-2528.769372	0.338078	-2528.431294	NO	-60.16	-20.01
F_40	-2528.769939	0.339878	-2528.430061	NO	-59.38	-19.24
F_41	-2528.770525	0.340540	-2528.429985	NO	-59.34	-19.19
F_42	-2528.770435	0.340753	-2528.429682	NO	-59.15	-19.00
F_43	-2528.767834	0.338604	-2528.429230	NO	-58.86	-18.71
**TS_D-E_1**	**-2528.717458**	**0.336960**	**-2528.380498**	**-173.281700**	**-28.28**	**11.87**
TS_D-E_2	-2528.717458	0.337076	-2528.380382	-172.954500	-28.21	11.94

TS_D-E_3	-2528.719748	0.339434	-2528.380314	-188.754300	-28.17	11.98
TS_D-E_4	-2528.719748	0.339437	-2528.380311	-188.784000	-28.16	11.98
TS_D-E_5	-2528.715095	0.337562	-2528.377533	-202.355400	-26.42	13.73
TS_D-E_6	-2528.715094	0.337565	-2528.377529	-202.051900	-26.42	13.73
TS_D-E_7	-2528.715095	0.337568	-2528.377527	-202.162200	-26.42	13.73
TS_D-E_8	-2528.715095	0.337572	-2528.377523	-202.403500	-26.42	13.73
TS_D-E_9	-2528.715096	0.337594	-2528.377502	-202.318900	-26.40	13.75
TS_D-E_10	-2528.715096	0.337600	-2528.377496	-202.197400	-26.40	13.75
TS_D-E_11	-2528.710732	0.334070	-2528.376662	-204.128200	-25.87	14.27
TS_D-E_12	-2528.710702	0.334468	-2528.376234	-204.666300	-25.61	14.54
TS_D-E_13	-2528.710700	0.334601	-2528.376099	-204.698800	-25.52	14.63
TS_D-E_14	-2528.710699	0.334661	-2528.376038	-204.670700	-25.48	14.66
TS_D-E_15	-2528.713122	0.337174	-2528.375948	-194.204800	-25.43	14.72
TS_D-E_16	-2528.710676	0.336459	-2528.374217	-170.957800	-24.34	15.81
TS_D-E_17	-2528.710676	0.336465	-2528.374211	-171.107100	-24.34	15.81
TS_D-E_18	-2528.710676	0.336485	-2528.374191	-171.010500	-24.32	15.82
**E_1**	**-2528.737742**	**0.335201**	**-2528.402541**	**NO**	**-42.11**	**-1.97**
E_2	-2528.736886	0.334800	-2528.402086	NO	-41.83	-1.68
E_3	-2528.735596	0.334762	-2528.400834	NO	-41.04	-0.90
E_4	-2528.737230	0.336845	-2528.400385	NO	-40.76	-0.61
E_5	-2528.736303	0.336007	-2528.400296	NO	-40.71	-0.56
E_6	-2528.737227	0.337119	-2528.400108	NO	-40.59	-0.44
E_7	-2528.737307	0.337308	-2528.399999	NO	-40.52	-0.37

E_8	-2528.737306	0.337349	-2528.399957	NO	-40.49	-0.35
E_9	-2528.737475	0.337562	-2528.399913	NO	-40.47	-0.32
E_10	-2528.737306	0.337413	-2528.399893	NO	-40.45	-0.30
E_11	-2528.737186	0.337315	-2528.399871	NO	-40.44	-0.29
E_12	-2528.735593	0.335831	-2528.399762	NO	-40.37	-0.22
E_13	-2528.738267	0.338572	-2528.399695	NO	-40.33	-0.18
E_14	-2528.737058	0.337611	-2528.399447	NO	-40.17	-0.02
E_15	-2528.738532	0.339114	-2528.399418	NO	-40.15	-0.01
E_16	-2528.737209	0.338145	-2528.399064	NO	-39.93	0.22
E_17	-2528.735830	0.336801	-2528.399029	NO	-39.91	0.24
E_18	-2528.736696	0.337696	-2528.399000	NO	-39.89	0.26
E_19	-2528.737421	0.338709	-2528.398712	NO	-39.71	0.44
E_20	-2528.736051	0.337402	-2528.398649	NO	-39.67	0.48
E_21	-2528.737951	0.339360	-2528.398591	NO	-39.64	0.51
E_22	-2528.738216	0.339720	-2528.398496	NO	-39.58	0.57
E_23	-2528.734534	0.336039	-2528.398495	NO	-39.58	0.57
E_24	-2528.737287	0.338889	-2528.398398	NO	-39.51	0.63
E_25	-2528.736487	0.338120	-2528.398367	NO	-39.50	0.65
E_26	-2528.736554	0.338308	-2528.398246	NO	-39.42	0.73
E_27	-2528.737373	0.339183	-2528.398190	NO	-39.38	0.76
E_28	-2528.736559	0.338423	-2528.398136	NO	-39.35	0.80
E_29	-2528.736650	0.338531	-2528.398119	NO	-39.34	0.81
E_30	-2528.734262	0.336285	-2528.397977	NO	-39.25	0.90
E_31	-2528.738234	0.340270	-2528.397964	NO	-39.24	0.91

E_32	-2528.737229	0.339351	-2528.397878	NO	-39.19	0.96
E_33	-2528.735583	0.337836	-2528.397747	NO	-39.11	1.04
E_34	-2528.736638	0.339112	-2528.397526	NO	-38.97	1.18
E_35	-2528.736160	0.338716	-2528.397444	NO	-38.92	1.23
E_36	-2528.738739	0.341319	-2528.397420	NO	-38.90	1.25
E_37	-2528.736575	0.339194	-2528.397381	NO	-38.88	1.27
E_38	-2528.737059	0.339681	-2528.397378	NO	-38.87	1.27
E_39	-2528.735438	0.338102	-2528.397336	NO	-38.85	1.30
E_40	-2528.733361	0.336141	-2528.397220	NO	-38.78	1.37
E_41	-2528.735678	0.338478	-2528.397200	NO	-38.76	1.39
E_42	-2528.736236	0.339096	-2528.397140	NO	-38.72	1.42
E_43	-2528.735765	0.338718	-2528.397047	NO	-38.67	1.48
E_44	-2528.736604	0.339661	-2528.396943	NO	-38.60	1.55
E_45	-2528.733108	0.336210	-2528.396898	NO	-38.57	1.57
E_46	-2528.733355	0.336727	-2528.396628	NO	-38.40	1.74
E_47	-2528.737981	0.341430	-2528.396551	NO	-38.36	1.79
E_48	-2528.736672	0.340483	-2528.396189	NO	-38.13	2.02
E_49	-2528.733957	0.337769	-2528.396188	NO	-38.13	2.02
E_50	-2528.733894	0.338205	-2528.395689	NO	-37.81	2.33
E_51	-2528.733576	0.337928	-2528.395648	NO	-37.79	2.36
E_52	-2528.734043	0.338820	-2528.395223	NO	-37.52	2.63
E_53	-2528.732827	0.338406	-2528.394421	NO	-37.02	3.13
E_54	-2528.735125	0.340729	-2528.394396	NO	-37.00	3.14

**TS_C-E-open_1**	**-2528.684587**	**0.336659**	**-2528.347928**	**-221.231400**	**-7.84**	**2.58**
TS_C-E-open_2	-2528.684360	0.336577	-2528.347783	-210.033500	-7.75	2.67
TS_C-E-open_3	-2528.684331	0.337190	-2528.347141	-198.409300	-7.35	3.07
TS_C-E-open_4	-2528.684330	0.337231	-2528.347099	-198.178000	-7.32	3.10
**E-open_1**	**-2528.721135**	**0.336620**	**-2528.384515**	**NO**	**-30.80**	**-20.38**
E-open_2	-2528.720190	0.335816	-2528.384374	NO	-30.71	-20.29
E-open_3	-2528.716469	0.332705	-2528.383764	NO	-30.33	-19.91
E-open_4	-2528.714189	0.332690	-2528.381499	NO	-28.91	-18.49
E-open_5	-2528.714829	0.333496	-2528.381333	NO	-28.81	-18.38
E-open_6	-2528.714828	0.333524	-2528.381304	NO	-28.79	-18.36
E-open_7	-2528.714839	0.333981	-2528.380858	NO	-28.51	-18.08
E-open_8	-2528.715071	0.334353	-2528.380718	NO	-28.42	-18.00
E-open_9	-2528.714890	0.334215	-2528.380675	NO	-28.39	-17.97
E-open_10	-2528.715796	0.335185	-2528.380611	NO	-28.35	-17.93
E-open_11	-2528.715869	0.335635	-2528.380234	NO	-28.12	-17.69
E-open_12	-2528.712372	0.332272	-2528.380100	NO	-28.03	-17.61
E-open_13	-2528.715104	0.335005	-2528.380099	NO	-28.03	-17.61
E-open_14	-2528.715546	0.335720	-2528.379826	NO	-27.86	-17.44
E-open_15	-2528.711937	0.332128	-2528.379809	NO	-27.85	-17.43
E-open_16	-2528.711937	0.332182	-2528.379755	NO	-27.82	-17.39
E-open_17	-2528.711415	0.332019	-2528.379396	NO	-27.59	-17.17
E-open_18	-2528.717305	0.338016	-2528.379289	NO	-27.52	-17.10
E-open_19	-2528.713413	0.334238	-2528.379175	NO	-27.45	-17.03

E-open_20	-2528.713413	0.334257	-2528.379156	NO	-27.44	-17.02
E-open_21	-2528.712497	0.333341	-2528.379156	NO	-27.44	-17.02
E-open_22	-2528.711571	0.332441	-2528.379130	NO	-27.42	-17.00
E-open_23	-2528.711459	0.332663	-2528.378796	NO	-27.21	-16.79
E-open_24	-2528.712097	0.333386	-2528.378711	NO	-27.16	-16.74
E-open_25	-2528.713770	0.335114	-2528.378656	NO	-27.13	-16.70
E-open_26	-2528.710963	0.332479	-2528.378484	NO	-27.02	-16.59
E-open_27	-2528.712622	0.334193	-2528.378429	NO	-26.98	-16.56
E-open_28	-2528.712154	0.333758	-2528.378396	NO	-26.96	-16.54
E-open_29	-2528.713284	0.335363	-2528.377921	NO	-26.66	-16.24
E-open_30	-2528.711099	0.333255	-2528.377844	NO	-26.62	-16.19
E-open_31	-2528.710865	0.333286	-2528.377579	NO	-26.45	-16.03
E-open_32	-2528.710864	0.333340	-2528.377524	NO	-26.42	-15.99
E-open_33	-2528.710581	0.333107	-2528.377474	NO	-26.38	-15.96
E-open_34	-2528.710070	0.332650	-2528.377420	NO	-26.35	-15.93
E-open_35	-2528.710289	0.332912	-2528.377377	NO	-26.32	-15.90
E-open_36	-2528.710289	0.332951	-2528.377338	NO	-26.30	-15.88
E-open_37	-2528.709723	0.332546	-2528.377177	NO	-26.20	-15.77
E-open_38	-2528.712957	0.335884	-2528.377073	NO	-26.13	-15.71
E-open_39	-2528.712160	0.335398	-2528.376762	NO	-25.94	-15.51
E-open_40	-2528.713500	0.336923	-2528.376577	NO	-25.82	-15.40
E-open_41	-2528.711741	0.335194	-2528.376547	NO	-25.80	-15.38
E-open_42	-2528.710522	0.334006	-2528.376516	NO	-25.78	-15.36
E-open_43	-2528.709826	0.333401	-2528.376425	NO	-25.73	-15.30

E-open_44	-2528.709361	0.333455	-2528.375906	NO	-25.40	-14.98
**TS_E-open-E_1**	**-2528.707929**	**0.338691**	**-2528.369238**	**-47.466400**	**-21.22**	**-10.79**
**TS_E-open-D_1**	**-2528.716813**	**0.338643**	**-2528.378170**	**-178.8094**		**-16.40**
TS_E-open-D_2	-2528.712931	0.337808	-2528.375123	-222.8461		-14.49
TS_E-open-D_3	-2528.711916	0.336797	-2528.375119	-135.77		-14.48
TS_E-open-D_4	-2528.714277	0.339550	-2528.374727	-219.8648		-14.24
TS_E-open-D_5	-2528.713346	0.340018	-2528.373328	-260.1944		-13.36
**R=Alk**						
**A_1**	**-2533.508291**	**0.415864**	**-2533.092427**	**NO**	**0.00**	
A_2	-2533.509123	0.417271	-2533.091852	NO	0.36	
A_3	-2533.506182	0.414731	-2533.091451	NO	0.61	
A_4	-2533.507260	0.416469	-2533.090791	NO	1.03	
A_5	-2533.508697	0.418000	-2533.090697	NO	1.09	
A_6	-2533.508889	0.418646	-2533.090243	NO	1.37	
A_7	-2533.508001	0.417830	-2533.090171	NO	1.42	
A_8	-2533.508088	0.418123	-2533.089965	NO	1.54	
A_9	-2533.506972	0.417172	-2533.089800	NO	1.65	
A_10	-2533.504314	0.414788	-2533.089526	NO	1.82	
A_11	-2533.506639	0.417680	-2533.088959	NO	2.18	
A_12	-2533.505168	0.416212	-2533.088956	NO	2.18	
A_13	-2533.502338	0.413417	-2533.088921	NO	2.20	

A_14	-2533.508166	0.419411	-2533.088755	NO	2.30	
A_15	-2533.505593	0.417116	-2533.088477	NO	2.48	
A_16	-2533.506348	0.417949	-2533.088399	NO	2.53	
A_17	-2533.503561	0.415355	-2533.088206	NO	2.65	
A_18	-2533.504805	0.416984	-2533.087821	NO	2.89	
A_19	-2533.506051	0.418430	-2533.087621	NO	3.02	
A_20	-2533.501182	0.413885	-2533.087297	NO	3.22	
A_21	-2533.503513	0.416230	-2533.087283	NO	3.23	
A_22	-2533.503768	0.416553	-2533.087215	NO	3.27	
A_23	-2533.505640	0.418535	-2533.087105	NO	3.34	
A_24	-2533.503867	0.416806	-2533.087061	NO	3.37	
A_25	-2533.502319	0.415470	-2533.086849	NO	3.50	
A_26	-2533.503978	0.417276	-2533.086702	NO	3.59	
A_27	-2533.504183	0.417581	-2533.086602	NO	3.66	
A_28	-2533.505116	0.418929	-2533.086187	NO	3.92	
A_29	-2533.505149	0.419055	-2533.086094	NO	3.97	
A_30	-2533.503521	0.417436	-2533.086085	NO	3.98	
A_31	-2533.502516	0.416474	-2533.086042	NO	4.01	
A_32	-2533.503225	0.417200	-2533.086025	NO	4.02	
A_33	-2533.503524	0.417526	-2533.085998	NO	4.03	
A_34	-2533.501885	0.415940	-2533.085945	NO	4.07	
A_35	-2533.504545	0.418696	-2533.085849	NO	4.13	
A_36	-2533.502576	0.416774	-2533.085802	NO	4.16	
A_37	-2533.504875	0.419079	-2533.085796	NO	4.16	

A_38	-2533.504178	0.418390	-2533.085788	NO	4.17	
A_39	-2533.502747	0.417126	-2533.085621	NO	4.27	
A_40	-2533.503762	0.418258	-2533.085504	NO	4.34	
A_41	-2533.502633	0.417166	-2533.085467	NO	4.37	
A_42	-2533.503301	0.417863	-2533.085438	NO	4.39	
A_43	-2533.502579	0.417260	-2533.085319	NO	4.46	
A_44	-2533.504314	0.419130	-2533.085184	NO	4.54	
A_45	-2533.500563	0.415429	-2533.085134	NO	4.58	
A_46	-2533.503113	0.418023	-2533.085090	NO	4.60	
A_47	-2533.504198	0.419136	-2533.085062	NO	4.62	
A_48	-2533.502201	0.417306	-2533.084895	NO	4.73	
A_49	-2533.499693	0.414901	-2533.084792	NO	4.79	
A_50	-2533.500861	0.416078	-2533.084783	NO	4.80	
A_51	-2533.502217	0.417449	-2533.084768	NO	4.81	
A_52	-2533.503379	0.418613	-2533.084766	NO	4.81	
A_53	-2533.500408	0.415808	-2533.084600	NO	4.91	
A_54	-2533.502972	0.418533	-2533.084439	NO	5.01	
A_55	-2533.503372	0.418996	-2533.084376	NO	5.05	
A_56	-2533.502582	0.418297	-2533.084285	NO	5.11	
A_57	-2533.502617	0.418350	-2533.084267	NO	5.12	
A_58	-2533.502006	0.417743	-2533.084263	NO	5.12	
A_59	-2533.502743	0.418587	-2533.084156	NO	5.19	
A_60	-2533.500228	0.416136	-2533.084092	NO	5.23	
A_61	-2533.503034	0.418964	-2533.084070	NO	5.24	

A_62	-2533.503373	0.419343	-2533.084030	NO	5.27	
A_63	-2533.500858	0.416868	-2533.083990	NO	5.29	
A_64	-2533.502890	0.419032	-2533.083858	NO	5.38	
A_65	-2533.502358	0.418542	-2533.083816	NO	5.40	
A_66	-2533.499000	0.415373	-2533.083627	NO	5.52	
A_67	-2533.501460	0.417882	-2533.083578	NO	5.55	
A_68	-2533.502852	0.419306	-2533.083546	NO	5.57	
A_69	-2533.502375	0.418866	-2533.083509	NO	5.60	
A_70	-2533.504113	0.420612	-2533.083501	NO	5.60	
A_71	-2533.498322	0.414962	-2533.083360	NO	5.69	
A_72	-2533.503836	0.420498	-2533.083338	NO	5.70	
A_73	-2533.502341	0.419012	-2533.083329	NO	5.71	
A_74	-2533.501819	0.418495	-2533.083324	NO	5.71	
A_75	-2533.501188	0.417944	-2533.083244	NO	5.76	
A_76	-2533.498320	0.415096	-2533.083224	NO	5.77	
A_77	-2533.500736	0.417605	-2533.083131	NO	5.83	
A_78	-2533.499058	0.416047	-2533.083011	NO	5.91	
A_79	-2533.502567	0.419687	-2533.082880	NO	5.99	
A_80	-2533.499362	0.416511	-2533.082851	NO	6.01	
A_81	-2533.499983	0.417138	-2533.082845	NO	6.01	
A_82	-2533.500137	0.417293	-2533.082844	NO	6.01	
A_83	-2533.502731	0.419894	-2533.082837	NO	6.02	
A_84	-2533.499983	0.417154	-2533.082829	NO	6.02	
A_85	-2533.502540	0.419712	-2533.082828	NO	6.02	

A_86	-2533.499420	0.416656	-2533.082764	NO	6.06	
A_87	-2533.500996	0.418259	-2533.082737	NO	6.08	
A_88	-2533.502976	0.420314	-2533.082662	NO	6.13	
A_89	-2533.504558	0.421923	-2533.082635	NO	6.14	
A_90	-2533.498678	0.416144	-2533.082534	NO	6.21	
A_91	-2533.502910	0.420379	-2533.082531	NO	6.21	
A_92	-2533.503194	0.420802	-2533.082392	NO	6.30	
A_93	-2533.498471	0.416232	-2533.082239	NO	6.39	
A_94	-2533.498667	0.416464	-2533.082203	NO	6.42	
A_95	-2533.502349	0.420180	-2533.082169	NO	6.44	
A_96	-2533.501574	0.419449	-2533.082125	NO	6.46	
A_97	-2533.501484	0.419428	-2533.082056	NO	6.51	
A_98	-2533.501484	0.419462	-2533.082022	NO	6.53	
A_99	-2533.500281	0.418313	-2533.081968	NO	6.56	
A_100	-2533.502452	0.421022	-2533.081430	NO	6.90	
A_101	-2533.499323	0.418280	-2533.081043	NO	7.14	
A_102	-2533.499584	0.418672	-2533.080912	NO	7.23	
A_103	-2533.499942	0.419051	-2533.080891	NO	7.24	
A_104	-2533.499843	0.419068	-2533.080775	NO	7.31	
A_105	-2533.499907	0.419318	-2533.080589	NO	7.43	
A_106	-2533.499527	0.419120	-2533.080407	NO	7.54	
A_107	-2533.497983	0.418066	-2533.079917	NO	7.85	
A_108	-2533.499475	0.419952	-2533.079523	NO	8.10	
A_109	-2533.500244	0.420763	-2533.079481	NO	8.12	

A_110	-2533.494150	0.415590	-2533.078560	NO	8.70	
A_111	-2533.498840	0.420425	-2533.078415	NO	8.79	
A_112	-2533.498139	0.419765	-2533.078374	NO	8.82	
A_113	-2533.491180	0.413299	-2533.077881	NO	9.13	
A_114	-2533.489972	0.412907	-2533.077065	NO	9.64	
A_115	-2533.494147	0.417594	-2533.076553	NO	9.96	
A_116	-2533.490256	0.414361	-2533.075895	NO	10.37	
A_117	-2533.490692	0.415396	-2533.075296	NO	10.75	
A_118	-2533.492213	0.416952	-2533.075261	NO	10.77	
A_119	-2533.492214	0.416969	-2533.075245	NO	10.78	
A_120	-2533.490061	0.414837	-2533.075224	NO	10.79	
A_121	-2533.492215	0.417030	-2533.075185	NO	10.82	
A_122	-2533.491100	0.416342	-2533.074758	NO	11.09	
A_123	-2533.489348	0.414642	-2533.074706	NO	11.12	
A_124	-2533.491811	0.417172	-2533.074639	NO	11.16	
A_125	-2533.489381	0.414791	-2533.074590	NO	11.19	
A_126	-2533.490781	0.416395	-2533.074386	NO	11.32	
A_127	-2533.489113	0.414738	-2533.074375	NO	11.33	
A_128	-2533.490769	0.416398	-2533.074371	NO	11.33	
A_129	-2533.489113	0.414745	-2533.074368	NO	11.33	
A_130	-2533.490210	0.416044	-2533.074166	NO	11.46	
A_131	-2533.488895	0.414819	-2533.074076	NO	11.52	
A_132	-2533.490032	0.416052	-2533.073980	NO	11.58	
A_133	-2533.493953	0.420165	-2533.073788	NO	11.70	

A_134	-2533.488062	0.414497	-2533.073565	NO	11.84	
A_135	-2533.487853	0.414357	-2533.073496	NO	11.88	
A_136	-2533.490173	0.416711	-2533.073462	NO	11.90	
A_137	-2533.489596	0.416139	-2533.073457	NO	11.90	
A_138	-2533.490685	0.417331	-2533.073354	NO	11.97	
A_139	-2533.488379	0.415092	-2533.073287	NO	12.01	
A_140	-2533.489764	0.416602	-2533.073162	NO	12.09	
A_141	-2533.490200	0.417052	-2533.073148	NO	12.10	
A_142	-2533.492089	0.418975	-2533.073114	NO	12.12	
A_143	-2533.491109	0.418057	-2533.073052	NO	12.16	
A_144	-2533.489627	0.416583	-2533.073044	NO	12.16	
A_145	-2533.490048	0.417068	-2533.072980	NO	12.20	
A_146	-2533.489626	0.416798	-2533.072828	NO	12.30	
A_147	-2533.489344	0.416578	-2533.072766	NO	12.34	
A_148	-2533.491211	0.418447	-2533.072764	NO	12.34	
A_149	-2533.487612	0.414892	-2533.072720	NO	12.37	
A_150	-2533.489668	0.417122	-2533.072546	NO	12.48	
A_151	-2533.488350	0.415891	-2533.072459	NO	12.53	
A_152	-2533.489716	0.417351	-2533.072365	NO	12.59	
A_153	-2533.488467	0.416218	-2533.072249	NO	12.66	
A_154	-2533.489397	0.417203	-2533.072194	NO	12.70	
A_155	-2533.486647	0.414478	-2533.072169	NO	12.71	
A_156	-2533.488413	0.416347	-2533.072066	NO	12.78	
A_157	-2533.489640	0.417728	-2533.071912	NO	12.87	

A_158	-2533.485900	0.414345	-2533.071555	NO	13.10	
A_159	-2533.488316	0.417494	-2533.070822	NO	13.56	
A_160	-2533.487575	0.416820	-2533.070755	NO	13.60	
A_161	-2533.491438	0.420785	-2533.070653	NO	13.66	
A_162	-2533.489168	0.418740	-2533.070428	NO	13.80	
A_163	-2533.490384	0.420246	-2533.070138	NO	13.99	
A_164	-2533.488300	0.418814	-2533.069486	NO	14.40	
A_165	-2533.487867	0.419041	-2533.068826	NO	14.81	
A_166	-2533.487348	0.419578	-2533.067770	NO	15.47	
A_167	-2533.486286	0.419776	-2533.066510	NO	16.26	
**TS_A-B_1**	**-2533.476325**	**0.409884**	**-2533.066441**	**-918.991600**	**16.31**	
TS_A-B_2	-2533.476111	0.410137	-2533.065974	-962.767700	16.60	
TS_A-B_3	-2533.474970	0.411191	-2533.063779	-983.728600	17.98	
TS_A-B_4	-2533.471067	0.407981	-2533.063086	-1010.656600	18.41	
TS_A-B_5	-2533.473091	0.410259	-2533.062832	-1025.348500	18.57	
TS_A-B_6	-2533.474449	0.411811	-2533.062638	-1181.823100	18.69	
TS_A-B_7	-2533.474943	0.412477	-2533.062466	-1091.422700	18.80	
**B_1**	**-2533.498908**	**0.413393**	**-2533.085515**	**NO**	**4.34**	
B_2	-2533.498780	0.414387	-2533.084393	NO	5.04	
B_3	-2533.499739	0.415427	-2533.084312	NO	5.09	
B_4	-2533.497872	0.413568	-2533.084304	NO	5.10	
B_5	-2533.499203	0.415615	-2533.083588	NO	5.55	

B_6	-2533.500340	0.416790	-2533.083550	NO	5.57	
B_7	-2533.498497	0.414981	-2533.083516	NO	5.59	
B_8	-2533.498227	0.414781	-2533.083446	NO	5.64	
B_9	-2533.497660	0.414378	-2533.083282	NO	5.74	
B_10	-2533.498682	0.415411	-2533.083271	NO	5.75	
B_11	-2533.498196	0.414972	-2533.083224	NO	5.77	
B_12	-2533.499335	0.416127	-2533.083208	NO	5.78	
B_13	-2533.498805	0.415701	-2533.083104	NO	5.85	
B_14	-2533.502309	0.419222	-2533.083087	NO	5.86	
B_15	-2533.497693	0.415072	-2533.082621	NO	6.15	
B_16	-2533.499302	0.416727	-2533.082575	NO	6.18	
B_17	-2533.498004	0.415453	-2533.082551	NO	6.20	
B_18	-2533.499084	0.416544	-2533.082540	NO	6.20	
B_19	-2533.497607	0.415070	-2533.082537	NO	6.21	
B_20	-2533.498231	0.415696	-2533.082535	NO	6.21	
B_21	-2533.498227	0.415722	-2533.082505	NO	6.23	
B_22	-2533.499355	0.416864	-2533.082491	NO	6.23	
B_23	-2533.499932	0.417460	-2533.082472	NO	6.25	
B_24	-2533.499051	0.416608	-2533.082443	NO	6.27	
B_25	-2533.498165	0.415752	-2533.082413	NO	6.28	
B_26	-2533.498397	0.416100	-2533.082297	NO	6.36	
B_27	-2533.499107	0.416868	-2533.082239	NO	6.39	
B_28	-2533.497771	0.415547	-2533.082224	NO	6.40	
B_29	-2533.497432	0.415235	-2533.082197	NO	6.42	

B_30	-2533.499967	0.417803	-2533.082164	NO	6.44	
B_31	-2533.500418	0.418283	-2533.082135	NO	6.46	
B_32	-2533.497954	0.415829	-2533.082125	NO	6.46	
B_33	-2533.498026	0.416009	-2533.082017	NO	6.53	
B_34	-2533.498278	0.416264	-2533.082014	NO	6.53	
B_35	-2533.497994	0.415988	-2533.082006	NO	6.54	
B_36	-2533.500640	0.418670	-2533.081970	NO	6.56	
B_37	-2533.497986	0.416027	-2533.081959	NO	6.57	
B_38	-2533.497987	0.416064	-2533.081923	NO	6.59	
B_39	-2533.498086	0.416488	-2533.081598	NO	6.80	
B_40	-2533.499214	0.417638	-2533.081576	NO	6.81	
B_41	-2533.495835	0.414290	-2533.081545	NO	6.83	
B_42	-2533.498532	0.417018	-2533.081514	NO	6.85	
B_43	-2533.498990	0.417538	-2533.081452	NO	6.89	
B_44	-2533.499007	0.417563	-2533.081444	NO	6.89	
B_45	-2533.496130	0.414705	-2533.081425	NO	6.90	
B_46	-2533.499649	0.418258	-2533.081391	NO	6.93	
B_47	-2533.497786	0.416417	-2533.081369	NO	6.94	
B_48	-2533.499002	0.417716	-2533.081286	NO	6.99	
B_49	-2533.497645	0.416362	-2533.081283	NO	6.99	
B_50	-2533.497862	0.416608	-2533.081254	NO	7.01	
B_51	-2533.497438	0.416224	-2533.081214	NO	7.04	
B_52	-2533.497549	0.416350	-2533.081199	NO	7.05	
B_53	-2533.497075	0.415919	-2533.081156	NO	7.07	

B_54	-2533.500282	0.419163	-2533.081119	NO	7.10	
B_55	-2533.498959	0.417878	-2533.081081	NO	7.12	
B_56	-2533.498966	0.417904	-2533.081062	NO	7.13	
B_57	-2533.499079	0.418075	-2533.081004	NO	7.17	
B_58	-2533.497741	0.416742	-2533.080999	NO	7.17	
B_59	-2533.498441	0.417486	-2533.080955	NO	7.20	
B_60	-2533.499346	0.418437	-2533.080909	NO	7.23	
B_61	-2533.499025	0.418116	-2533.080909	NO	7.23	
B_62	-2533.499347	0.418507	-2533.080840	NO	7.27	
B_63	-2533.499438	0.418652	-2533.080786	NO	7.30	
B_64	-2533.498472	0.417689	-2533.080783	NO	7.31	
B_65	-2533.497330	0.416550	-2533.080780	NO	7.31	
B_66	-2533.493673	0.413064	-2533.080609	NO	7.42	
B_67	-2533.499108	0.418602	-2533.080506	NO	7.48	
B_68	-2533.498025	0.417524	-2533.080501	NO	7.48	
B_69	-2533.498268	0.417811	-2533.080457	NO	7.51	
B_70	-2533.499466	0.419162	-2533.080304	NO	7.61	
B_71	-2533.497435	0.417155	-2533.080280	NO	7.62	
B_72	-2533.497509	0.417299	-2533.080210	NO	7.67	
B_73	-2533.498294	0.418093	-2533.080201	NO	7.67	
B_74	-2533.498779	0.418630	-2533.080149	NO	7.70	
B_75	-2533.497940	0.417839	-2533.080101	NO	7.73	
B_76	-2533.493644	0.413641	-2533.080003	NO	7.80	
B_77	-2533.497506	0.417506	-2533.080000	NO	7.80	

B_78	-2533.496486	0.416517	-2533.079969	NO	7.82	
B_79	-2533.498607	0.418843	-2533.079764	NO	7.95	
B_80	-2533.498535	0.418801	-2533.079734	NO	7.96	
B_81	-2533.496439	0.416707	-2533.079732	NO	7.97	
B_82	-2533.497692	0.418043	-2533.079649	NO	8.02	
B_83	-2533.499232	0.419587	-2533.079645	NO	8.02	
B_84	-2533.499232	0.419591	-2533.079641	NO	8.02	
B_85	-2533.497865	0.418250	-2533.079615	NO	8.04	
B_86	-2533.499217	0.419605	-2533.079612	NO	8.04	
B_87	-2533.499232	0.419641	-2533.079591	NO	8.05	
B_88	-2533.498687	0.419154	-2533.079533	NO	8.09	
B_89	-2533.497834	0.418318	-2533.079516	NO	8.10	
B_90	-2533.498687	0.419173	-2533.079514	NO	8.10	
B_91	-2533.498686	0.419194	-2533.079492	NO	8.12	
B_92	-2533.498012	0.418591	-2533.079421	NO	8.16	
B_93	-2533.498821	0.419407	-2533.079414	NO	8.17	
B_94	-2533.498421	0.419036	-2533.079385	NO	8.18	
B_95	-2533.500478	0.421099	-2533.079379	NO	8.19	
B_96	-2533.494976	0.415602	-2533.079374	NO	8.19	
B_97	-2533.498026	0.418730	-2533.079296	NO	8.24	
B_98	-2533.498865	0.419571	-2533.079294	NO	8.24	
B_99	-2533.498417	0.419142	-2533.079275	NO	8.25	
B_100	-2533.499433	0.420188	-2533.079245	NO	8.27	
B_101	-2533.496422	0.417355	-2533.079067	NO	8.38	

B_102	-2533.498066	0.419019	-2533.079047	NO	8.40	
B_103	-2533.497793	0.418758	-2533.079035	NO	8.40	
B_104	-2533.496498	0.417540	-2533.078958	NO	8.45	
B_105	-2533.498275	0.419318	-2533.078957	NO	8.45	
B_106	-2533.497561	0.418667	-2533.078894	NO	8.49	
B_107	-2533.495603	0.416717	-2533.078886	NO	8.50	
B_108	-2533.498461	0.419620	-2533.078841	NO	8.52	
B_109	-2533.498415	0.419614	-2533.078801	NO	8.55	
B_110	-2533.499326	0.420684	-2533.078642	NO	8.65	
B_111	-2533.497461	0.418826	-2533.078635	NO	8.65	
B_112	-2533.498400	0.419808	-2533.078592	NO	8.68	
B_113	-2533.496565	0.417987	-2533.078578	NO	8.69	
B_114	-2533.495894	0.417407	-2533.078487	NO	8.75	
B_115	-2533.498849	0.420459	-2533.078390	NO	8.81	
B_116	-2533.498272	0.419903	-2533.078369	NO	8.82	
B_117	-2533.498225	0.419929	-2533.078296	NO	8.87	
B_118	-2533.496027	0.417794	-2533.078233	NO	8.91	
B_119	-2533.495880	0.417823	-2533.078057	NO	9.02	
B_120	-2533.497007	0.419062	-2533.077945	NO	9.09	
B_121	-2533.497799	0.419862	-2533.077937	NO	9.09	
B_122	-2533.495075	0.417183	-2533.077892	NO	9.12	
B_123	-2533.496602	0.418820	-2533.077782	NO	9.19	
B_124	-2533.497929	0.420365	-2533.077564	NO	9.33	
B_125	-2533.497836	0.420429	-2533.077407	NO	9.43	

B_126	-2533.495316	0.417910	-2533.077406	NO	9.43	
B_127	-2533.496230	0.418869	-2533.077361	NO	9.45	
B_128	-2533.495405	0.418148	-2533.077257	NO	9.52	
B_129	-2533.497554	0.420394	-2533.077160	NO	9.58	
B_130	-2533.498240	0.421172	-2533.077068	NO	9.64	
B_131	-2533.498270	0.421330	-2533.076940	NO	9.72	
B_132	-2533.496580	0.420340	-2533.076240	NO	10.16	
B_133	-2533.496721	0.420598	-2533.076123	NO	10.23	
B_134	-2533.496922	0.421049	-2533.075873	NO	10.39	
B_135	-2533.495561	0.419783	-2533.075778	NO	10.45	
B_136	-2533.497757	0.422234	-2533.075523	NO	10.61	
B_137	-2533.497500	0.421979	-2533.075521	NO	10.61	
**TS_B_C_1**	**-2533.495507**	**0.413093**	**-2533.082414**	**-33.33**	**6.28**	
TS_B_C_2	-2533.495546	0.414105	-2533.081441	-45.26	6.89	
TS_B_C_3	-2533.496736	0.418088	-2533.078648	-25.40	8.65	
TS_B_C_4	-2533.494435	0.415819	-2533.078616	-69.94	8.67	
TS_B_C_5	-2533.495764	0.418061	-2533.077703	-107.37	9.24	
TS_B_C_6	-2533.492527	0.415055	-2533.077472	-120.47	9.38	
TS_B_C_7	-2533.495476	0.418132	-2533.077344	-35.82	9.46	
TS_B_C_8	-2533.494876	0.418026	-2533.076850	-23.40	9.77	
**C_1**	**-2533.531971**	**0.418624**	**-2533.113347**	**NO**	**-13.13**	
C_2	-2533.533231	0.419998	-2533.113233	NO	-13.06	

C_3	-2533.533231	0.420001	-2533.113230	NO	-13.05	
C_4	-2533.52993274	0.416751	-2533.11318174	NO	-13.02	
C_5	-2533.53249144	0.419746	-2533.11274544	NO	-12.75	
C_6	-2533.53350425	0.420771	-2533.11273325	NO	-12.74	
C_7	-2533.53350329	0.42078	-2533.11272329	NO	-12.74	
C_8	-2533.53122951	0.418531	-2533.11269851	NO	-12.72	
C_9	-2533.532494	0.419825	-2533.112669	NO	-12.70	
C_10	-2533.532496	0.419839	-2533.112657	NO	-12.69	
C_11	-2533.531150	0.418839	-2533.112311	NO	-12.48	
C_12	-2533.533542	0.421647	-2533.111895	NO	-12.22	
C_13	-2533.533542	0.421690	-2533.111852	NO	-12.19	
C_14	-2533.533535	0.421718	-2533.111817	NO	-12.17	
C_15	-2533.532488	0.420738	-2533.111750	NO	-12.13	
C_16	-2533.533537	0.421854	-2533.111683	NO	-12.08	
C_17	-2533.53062441	0.419004	-2533.111620	NO	-12.04	
C_18	-2533.53059214	0.419172	-2533.111420	NO	-11.92	
C_19	-2533.532492	0.421111	-2533.111381	NO	-11.89	
C_20	-2533.532492	0.421115	-2533.111377	NO	-11.89	
C_21	-2533.535247	0.423871	-2533.111376	NO	-11.89	
C_22	-2533.535247	0.423900	-2533.111347	NO	-11.87	
C_23	-2533.535247	0.423929	-2533.111318	NO	-11.85	
C_24	-2533.532262	0.421058	-2533.111204	NO	-11.78	
C_25	-2533.529758	0.418602	-2533.111156	NO	-11.75	
C_26	-2533.531918	0.420949	-2533.110969	NO	-11.64	

C_27	-2533.531635	0.420754	-2533.110881	NO	-11.58	
C_28	-2533.531635	0.420785	-2533.110850	NO	-11.56	
C_29	-2533.533386	0.422551	-2533.110835	NO	-11.55	
C_30	-2533.530710	0.419889	-2533.110821	NO	-11.54	
C_31	-2533.531993	0.421205	-2533.110788	NO	-11.52	
C_32	-2533.530803	0.420106	-2533.110697	NO	-11.46	
C_33	-2533.530106	0.419658	-2533.110448	NO	-11.31	
C_34	-2533.530107	0.419659	-2533.110448	NO	-11.31	
C_35	-2533.533874	0.423465	-2533.110409	NO	-11.28	
C_36	-2533.532938	0.422608	-2533.110330	NO	-11.23	
C_37	-2533.533598	0.423282	-2533.110316	NO	-11.23	
C_38	-2533.533602	0.423310	-2533.110292	NO	-11.21	
C_39	-2533.532938	0.422658	-2533.110280	NO	-11.20	
C_40	-2533.529838	0.419708	-2533.110130	NO	-11.11	
C_41	-2533.533487	0.423420	-2533.110067	NO	-11.07	
C_42	-2533.534304	0.424261	-2533.110043	NO	-11.05	
C_43	-2533.532228	0.422197	-2533.110031	NO	-11.05	
C_44	-2533.531033	0.421045	-2533.109988	NO	-11.02	
C_45	-2533.530701	0.420770	-2533.109931	NO	-10.98	
C_46	-2533.534303	0.424373	-2533.109930	NO	-10.98	
C_47	-2533.532955	0.423028	-2533.109927	NO	-10.98	
C_48	-2533.530701	0.420781	-2533.109920	NO	-10.98	
C_49	-2533.534304	0.424393	-2533.109911	NO	-10.97	
C_50	-2533.530804	0.421074	-2533.109730	NO	-10.86	

C_51	-2533.534014	0.424300	-2533.109714	NO	-10.85	
C_52	-2533.531973	0.422261	-2533.109712	NO	-10.85	
C_53	-2533.531456	0.421785	-2533.109671	NO	-10.82	
C_54	-2533.529278	0.419743	-2533.109535	NO	-10.74	
C_55	-2533.533475	0.423945	-2533.109530	NO	-10.73	
C_56	-2533.533474	0.423971	-2533.109503	NO	-10.72	
C_57	-2533.531560	0.422104	-2533.109456	NO	-10.69	
C_58	-2533.533476	0.424079	-2533.109397	NO	-10.65	
C_59	-2533.530923	0.421655	-2533.109268	NO	-10.57	
C_60	-2533.531325	0.422149	-2533.109176	NO	-10.51	
C_61	-2533.531154	0.421982	-2533.109172	NO	-10.51	
C_62	-2533.530674	0.421521	-2533.109153	NO	-10.50	
C_63	-2533.533229	0.424115	-2533.109114	NO	-10.47	
C_64	-2533.532646	0.423542	-2533.109104	NO	-10.47	
C_65	-2533.531925	0.422838	-2533.109087	NO	-10.45	
C_66	-2533.533509	0.424462	-2533.109047	NO	-10.43	
C_67	-2533.532650	0.423605	-2533.109045	NO	-10.43	
C_68	-2533.531154	0.422214	-2533.108940	NO	-10.36	
C_69	-2533.532624	0.423766	-2533.108858	NO	-10.31	
C_70	-2533.531122	0.422303	-2533.108819	NO	-10.29	
C_71	-2533.534285	0.425659	-2533.108626	NO	-10.17	
C_72	-2533.531686	0.423092	-2533.108594	NO	-10.14	
C_73	-2533.530960	0.422426	-2533.108534	NO	-10.11	
C_74	-2533.532644	0.424155	-2533.108489	NO	-10.08	

C_75	-2533.530979	0.422744	-2533.108235	NO	-9.92	
C_76	-2533.532577	0.424406	-2533.108171	NO	-9.88	
C_77	-2533.531516	0.423377	-2533.108139	NO	-9.86	
C_78	-2533.531010	0.423081	-2533.107929	NO	-9.73	
C_79	-2533.531010	0.423098	-2533.107912	NO	-9.72	
C_80	-2533.529237	0.421365	-2533.107872	NO	-9.69	
C_81	-2533.531061	0.423259	-2533.107802	NO	-9.65	
C_82	-2533.531021	0.423282	-2533.107739	NO	-9.61	
C_83	-2533.529511	0.421846	-2533.107665	NO	-9.56	
C_84	-2533.529973	0.423159	-2533.106814	NO	-9.03	
C_85	-2533.531503	0.424829	-2533.106674	NO	-8.94	
C_86	-2533.530801	0.424655	-2533.106146	NO	-8.61	
C_87	-2533.530832	0.425114	-2533.105718	NO	-8.34	
C_88	-2533.528028	0.423581	-2533.104447	NO	-7.54	
**TS_C-D_1**	**-2533.529894**	**0.420880**	**-2533.109014**	**-143.03**	**-10.41**	
TS_C-D_2	-2533.529894	0.420891	-2533.109003	-142.99	-10.40	
TS_C-D_3	-2533.528946	0.420809	-2533.108137	-141.04	-9.86	
TS_C-D_4	-2533.528452	0.420871	-2533.107581	-147.70	-9.51	
TS_C-D_5	-2533.529303	0.422472	-2533.106831	-143.91	-9.04	
TS_C-D_6	-2533.529205	0.424362	-2533.104843	-145.90	-7.79	
TS_C-D_7	-2533.529205	0.424390	-2533.104815	-145.97	-7.77	
**D_1**	**-2533.58190104**	**0.417837**	**-2533.16406404**	**NO**	**-44.95**	**0.00**

D_2	-2533.583168	0.420338	-2533.162830	NO	-44.18	
D_3	-2533.582748	0.420027	-2533.162721	NO	-44.11	
D_4	-2533.58194912	0.419259	-2533.16269012	NO	-44.09	
D_5	-2533.583778	0.422181	-2533.161597	NO	-43.40	
D_6	-2533.582780	0.421934	-2533.160846	NO	-42.93	
D_7	-2533.581407	0.420682	-2533.160725	NO	-42.86	
D_8	-2533.584669	0.424238	-2533.160431	NO	-42.67	
D_9	-2533.581724	0.421540	-2533.160184	NO	-42.52	
D_10	-2533.582043	0.422121	-2533.159922	NO	-42.35	
D_11	-2533.582064	0.422364	-2533.159700	NO	-42.21	
D_12	-2533.581651	0.421968	-2533.159683	NO	-42.20	
D_13	-2533.583216	0.423647	-2533.159569	NO	-42.13	
D_14	-2533.582080	0.422555	-2533.159525	NO	-42.11	
D_15	-2533.581562	0.422241	-2533.159321	NO	-41.98	
D_16	-2533.582456	0.423357	-2533.159099	NO	-41.84	
D_17	-2533.583208	0.424170	-2533.159038	NO	-41.80	
D_18	-2533.583099	0.424135	-2533.158964	NO	-41.75	
D_19	-2533.578288	0.419358	-2533.158930	NO	-41.73	
D_20	-2533.580994	0.422189	-2533.158805	NO	-41.65	
D_21	-2533.581975	0.423264	-2533.158711	NO	-41.59	
D_22	-2533.581634	0.422958	-2533.158676	NO	-41.57	
D_23	-2533.582052	0.423472	-2533.158580	NO	-41.51	
D_24	-2533.579251	0.420702	-2533.158549	NO	-41.49	
D_25	-2533.582188	0.423658	-2533.158530	NO	-41.48	

D_26	-2533.582072	0.423695	-2533.158377	NO	-41.38	
D_27	-2533.582332	0.423990	-2533.158342	NO	-41.36	
D_28	-2533.581682	0.423385	-2533.158297	NO	-41.33	
D_29	-2533.582792	0.424563	-2533.158229	NO	-41.29	
D_30	-2533.582089	0.423863	-2533.158226	NO	-41.29	
D_31	-2533.584210	0.425987	-2533.158223	NO	-41.29	
D_32	-2533.581238	0.423059	-2533.158179	NO	-41.26	
D_33	-2533.581631	0.423486	-2533.158145	NO	-41.24	
D_34	-2533.582808	0.424848	-2533.157960	NO	-41.12	
D_35	-2533.581767	0.423850	-2533.157917	NO	-41.10	
D_36	-2533.581956	0.424044	-2533.157912	NO	-41.09	
D_37	-2533.581875	0.424079	-2533.157796	NO	-41.02	
D_38	-2533.580804	0.423010	-2533.157794	NO	-41.02	
D_39	-2533.581755	0.424018	-2533.157737	NO	-40.98	
D_40	-2533.582763	0.425026	-2533.157737	NO	-40.98	
D_41	-2533.582090	0.424356	-2533.157734	NO	-40.98	
D_42	-2533.582077	0.424403	-2533.157674	NO	-40.94	
D_43	-2533.583094	0.425494	-2533.157600	NO	-40.90	
D_44	-2533.581629	0.424100	-2533.157529	NO	-40.85	
D_45	-2533.580884	0.423365	-2533.157519	NO	-40.85	
D_46	-2533.576570	0.419113	-2533.157457	NO	-40.81	
D_47	-2533.581389	0.424067	-2533.157322	NO	-40.72	
D_48	-2533.580751	0.423430	-2533.157321	NO	-40.72	
D_49	-2533.579611	0.422532	-2533.157079	NO	-40.57	

D_50	-2533.579421	0.422526	-2533.156895	NO	-40.45	
D_51	-2533.578177	0.421328	-2533.156849	NO	-40.43	
D_52	-2533.582333	0.425652	-2533.156681	NO	-40.32	
D_53	-2533.582019	0.425346	-2533.156673	NO	-40.32	
D_54	-2533.582378	0.425758	-2533.156620	NO	-40.28	
D_55	-2533.582493	0.425907	-2533.156586	NO	-40.26	
D_56	-2533.583097	0.426516	-2533.156581	NO	-40.26	
D_57	-2533.582026	0.425472	-2533.156554	NO	-40.24	
D_58	-2533.579469	0.422925	-2533.156544	NO	-40.23	
D_59	-2533.581408	0.424864	-2533.156544	NO	-40.23	
D_60	-2533.583582	0.427111	-2533.156471	NO	-40.19	
D_61	-2533.582991	0.426792	-2533.156199	NO	-40.02	
D_62	-2533.582714	0.426574	-2533.156140	NO	-39.98	
D_63	-2533.579947	0.423853	-2533.156094	NO	-39.95	
D_64	-2533.580521	0.424513	-2533.156008	NO	-39.90	
D_65	-2533.581266	0.425270	-2533.155996	NO	-39.89	
D_66	-2533.579263	0.423314	-2533.155949	NO	-39.86	
D_67	-2533.580910	0.424996	-2533.155914	NO	-39.84	
D_68	-2533.581266	0.425356	-2533.155910	NO	-39.84	
D_69	-2533.578820	0.422966	-2533.155854	NO	-39.80	
D_70	-2533.579093	0.423278	-2533.155815	NO	-39.78	
D_71	-2533.582089	0.426286	-2533.155803	NO	-39.77	
D_72	-2533.582334	0.426591	-2533.155743	NO	-39.73	
D_73	-2533.581005	0.425341	-2533.155664	NO	-39.68	

D_74	-2533.579682	0.424026	-2533.155656	NO	-39.68	
D_75	-2533.582926	0.427437	-2533.155489	NO	-39.57	
D_76	-2533.580532	0.425141	-2533.155391	NO	-39.51	
D_77	-2533.581279	0.426057	-2533.155222	NO	-39.40	
D_78	-2533.580553	0.425346	-2533.155207	NO	-39.40	
D_79	-2533.581279	0.426076	-2533.155203	NO	-39.39	
D_80	-2533.580142	0.424955	-2533.155187	NO	-39.38	
D_81	-2533.578854	0.423676	-2533.155178	NO	-39.38	
D_82	-2533.580141	0.424978	-2533.155163	NO	-39.37	
D_83	-2533.579435	0.424460	-2533.154975	NO	-39.25	
D_84	-2533.578505	0.423599	-2533.154906	NO	-39.21	
D_85	-2533.580233	0.425329	-2533.154904	NO	-39.21	
D_86	-2533.580609	0.425977	-2533.154632	NO	-39.03	
D_87	-2533.579702	0.425270	-2533.154432	NO	-38.91	
D_88	-2533.579552	0.425132	-2533.154420	NO	-38.90	
D_89	-2533.578421	0.424510	-2533.153911	NO	-38.58	
D_90	-2533.579396	0.425565	-2533.153831	NO	-38.53	
D_91	-2533.578756	0.425076	-2533.153680	NO	-38.44	
D_92	-2533.578837	0.425199	-2533.153638	NO	-38.41	
D_93	-2533.580460	0.426894	-2533.153566	NO	-38.37	
D_94	-2533.582304	0.428763	-2533.153541	NO	-38.35	
D_95	-2533.578971	0.425464	-2533.153507	NO	-38.33	
D_96	-2533.579671	0.426423	-2533.153248	NO	-38.17	
D_97	-2533.581037	0.428500	-2533.152537	NO	-37.72	

D_98	-2533.579126	0.426790	-2533.152336	NO	-37.59	
D_99	-2533.576895	0.424561	-2533.152334	NO	-37.59	
D_100	-2533.576166	0.424504	-2533.151662	NO	-37.17	
D_101	-2533.577007	0.425604	-2533.151403	NO	-37.01	
**TS_D-F_1**	**-2533.579583**	**0.419266**	**-2533.160317**	**-749.57**		**2.35**
TS_D-F_2	-2533.579583	0.419271	-2533.160312	-749.63		2.35
TS_D-F_3	-2533.579583	0.419273	-2533.160310	-749.60		2.36
TS_D-F_4	-2533.578817	0.418941	-2533.159876	-765.85		2.63
TS_D-F_5	-2533.577502	0.417908	-2533.159594	-569.21		2.81
TS_D-F_6	-2533.577502	0.417926	-2533.159576	-569.05		2.82
TS_D-F_7	-2533.577675	0.418428	-2533.159247	-959.04		3.02
TS_D-F_8	-2533.577767	0.418527	-2533.159240	-900.74		3.03
TS_D-F_9	-2533.577606	0.418541	-2533.159065	-940.90		3.14
TS_D-F_10	-2533.578031	0.419331	-2533.158700	-738.80		3.37
TS_D-F_11	-2533.580061	0.421424	-2533.158637	-769.93		3.41
TS_D-F_12	-2533.576720	0.418395	-2533.158325	-943.13		3.60
TS_D-F_13	-2533.577415	0.419221	-2533.158194	-951.21		3.68
TS_D-F_14	-2533.577132	0.419433	-2533.157699	-957.24		3.99
TS_D-F_15	-2533.576115	0.418496	-2533.157619	-914.91		4.04
TS_D-F_16	-2533.577767	0.420256	-2533.157511	-961.58		4.11
TS_D-F_17	-2533.577981	0.420484	-2533.157497	-908.69		4.12
TS_D-F_18	-2533.578498	0.421113	-2533.157385	-889.31		4.19
TS_D-F_19	-2533.577920	0.420745	-2533.157175	-933.67		4.32

TS_D-F_20	-2533.577920	0.420758	-2533.157162	-932.80		4.33
TS_D-F_21	-2533.577920	0.420769	-2533.157151	-932.62		4.34
TS_D-F_22	-2533.577920	0.420772	-2533.157148	-932.92		4.34
TS_D-F_23	-2533.578822	0.421885	-2533.156937	-926.68		4.47
TS_D-F_24	-2533.577078	0.420246	-2533.156832	-961.68		4.54
TS_D-F_25	-2533.577952	0.421209	-2533.156743	-966.27		4.59
TS_D-F_26	-2533.576569	0.419901	-2533.156668	-943.29		4.64
TS_D-F_27	-2533.577992	0.421373	-2533.156619	-962.94		4.67
TS_D-F_28	-2533.575717	0.419172	-2533.156545	-917.46		4.72
TS_D-F_29	-2533.577658	0.421176	-2533.156482	-901.44		4.76
TS_D-F_30	-2533.579094	0.422633	-2533.156461	-968.79		4.77
TS_D-F_31	-2533.576775	0.420328	-2533.156447	-964.58		4.78
TS_D-F_32	-2533.579094	0.422698	-2533.156396	-968.66		4.81
TS_D-F_33	-2533.579094	0.422699	-2533.156395	-968.66		4.81
TS_D-F_34	-2533.577940	0.421677	-2533.156263	-919.11		4.89
TS_D-F_35	-2533.577941	0.421684	-2533.156257	-918.74		4.90
TS_D-F_36	-2533.579212	0.423294	-2533.155918	-949.86		5.11
TS_D-F_37	-2533.579212	0.423316	-2533.155896	-950.06		5.13
TS_D-F_38	-2533.575701	0.420186	-2533.155515	-945.04		5.36
TS_D-F_39	-2533.578038	0.422688	-2533.155350	-987.11		5.47
TS_D-F_40	-2533.577449	0.422554	-2533.154895	-897.37		5.75
TS_D-F_41	-2533.575467	0.421048	-2533.154419	-943.32		6.05
**F_1**	**-2533.62104352**	**0.422579**	**-2533.19846452**	**NO**		**-21.59**

F_2	-2533.622116	0.423954	-2533.198162	NO		-21.40
F_3	-2533.622096	0.424452	-2533.197644	NO		-21.07
F_4	-2533.621756	0.424211	-2533.197545	NO		-21.01
F_5	-2533.623429	0.425961	-2533.197468	NO		-20.96
F_6	-2533.620302	0.422928	-2533.197374	NO		-20.90
F_7	-2533.621921	0.424579	-2533.197342	NO		-20.88
F_8	-2533.621095	0.423896	-2533.197199	NO		-20.79
F_9	-2533.623057	0.425885	-2533.197172	NO		-20.78
F_10	-2533.621751	0.424709	-2533.197042	NO		-20.69
F_11	-2533.621167	0.424201	-2533.196966	NO		-20.65
F_12	-2533.622184	0.425223	-2533.196961	NO		-20.64
F_13	-2533.621167	0.424229	-2533.196938	NO		-20.63
F_14	-2533.621053	0.424139	-2533.196914	NO		-20.61
F_15	-2533.621901	0.425032	-2533.196869	NO		-20.59
F_16	-2533.621178	0.424374	-2533.196804	NO		-20.54
F_17	-2533.622126	0.425389	-2533.196737	NO		-20.50
F_18	-2533.621014	0.424312	-2533.196702	NO		-20.48
F_19	-2533.621084	0.424516	-2533.196568	NO		-20.40
F_20	-2533.623257	0.426720	-2533.196537	NO		-20.38
F_21	-2533.623256	0.426721	-2533.196535	NO		-20.38
F_22	-2533.620625	0.424102	-2533.196523	NO		-20.37
F_23	-2533.622617	0.426143	-2533.196474	NO		-20.34
F_24	-2533.622136	0.425704	-2533.196432	NO		-20.31
F_25	-2533.620777	0.424396	-2533.196381	NO		-20.28

F_26	-2533.621559	0.425189	-2533.196370	NO		-20.27
F_27	-2533.620126	0.423803	-2533.196323	NO		-20.24
F_28	-2533.621249	0.424930	-2533.196319	NO		-20.24
F_29	-2533.621477	0.425212	-2533.196265	NO		-20.21
F_30	-2533.622946	0.426683	-2533.196263	NO		-20.21
F_31	-2533.620857	0.424675	-2533.196182	NO		-20.15
F_32	-2533.620059	0.423938	-2533.196121	NO		-20.12
F_33	-2533.622810	0.426748	-2533.196062	NO		-20.08
F_34	-2533.623713	0.427683	-2533.196030	NO		-20.06
F_35	-2533.621267	0.425408	-2533.195859	NO		-19.95
F_36	-2533.622116	0.426291	-2533.195825	NO		-19.93
F_37	-2533.622595	0.426808	-2533.195787	NO		-19.91
F_38	-2533.621338	0.425579	-2533.195759	NO		-19.89
F_39	-2533.622756	0.427009	-2533.195747	NO		-19.88
F_40	-2533.621338	0.425596	-2533.195742	NO		-19.88
F_41	-2533.622756	0.427019	-2533.195737	NO		-19.88
F_42	-2533.620627	0.424892	-2533.195735	NO		-19.87
F_43	-2533.619127	0.423400	-2533.195727	NO		-19.87
F_44	-2533.623129	0.427423	-2533.195706	NO		-19.86
F_45	-2533.621402	0.425734	-2533.195668	NO		-19.83
F_46	-2533.621402	0.425739	-2533.195663	NO		-19.83
F_47	-2533.619457	0.423813	-2533.195644	NO		-19.82
F_48	-2533.622057	0.426433	-2533.195624	NO		-19.80
F_49	-2533.621725	0.426101	-2533.195624	NO		-19.80

F_50	-2533.620646	0.425057	-2533.195589	NO		-19.78
F_51	-2533.620473	0.424886	-2533.195587	NO		-19.78
F_52	-2533.621975	0.426440	-2533.195535	NO		-19.75
F_53	-2533.620618	0.425092	-2533.195526	NO		-19.74
F_54	-2533.620618	0.425096	-2533.195522	NO		-19.74
F_55	-2533.621399	0.425940	-2533.195459	NO		-19.70
F_56	-2533.618070	0.422623	-2533.195447	NO		-19.69
F_57	-2533.620437	0.425012	-2533.195425	NO		-19.68
F_58	-2533.619500	0.424167	-2533.195333	NO		-19.62
F_59	-2533.621818	0.426518	-2533.195300	NO		-19.60
F_60	-2533.621835	0.426586	-2533.195249	NO		-19.57
F_61	-2533.618872	0.423635	-2533.195237	NO		-19.56
F_62	-2533.620972	0.425824	-2533.195148	NO		-19.51
F_63	-2533.618855	0.423715	-2533.195140	NO		-19.50
F_64	-2533.622353	0.427224	-2533.195129	NO		-19.49
F_65	-2533.617959	0.422915	-2533.195044	NO		-19.44
F_66	-2533.619933	0.424946	-2533.194987	NO		-19.40
F_67	-2533.619694	0.424760	-2533.194934	NO		-19.37
F_68	-2533.623892	0.428985	-2533.194907	NO		-19.35
F_69	-2533.620342	0.425458	-2533.194884	NO		-19.34
F_70	-2533.620491	0.425651	-2533.194840	NO		-19.31
F_71	-2533.621717	0.426885	-2533.194832	NO		-19.31
F_72	-2533.618062	0.423240	-2533.194822	NO		-19.30
F_73	-2533.622170	0.427406	-2533.194764	NO		-19.26

F_74	-2533.621333	0.426613	-2533.194720	NO		-19.24
F_75	-2533.621460	0.426778	-2533.194682	NO		-19.21
F_76	-2533.620495	0.425856	-2533.194639	NO		-19.19
F_77	-2533.622899	0.428262	-2533.194637	NO		-19.19
F_78	-2533.620495	0.425868	-2533.194627	NO		-19.18
F_79	-2533.619019	0.424401	-2533.194618	NO		-19.17
F_80	-2533.619019	0.424430	-2533.194589	NO		-19.15
F_81	-2533.619182	0.424612	-2533.194570	NO		-19.14
F_82	-2533.618934	0.424366	-2533.194568	NO		-19.14
F_83	-2533.619689	0.425183	-2533.194506	NO		-19.10
F_84	-2533.620295	0.425800	-2533.194495	NO		-19.10
F_85	-2533.622142	0.427659	-2533.194483	NO		-19.09
F_86	-2533.620241	0.425767	-2533.194474	NO		-19.08
F_87	-2533.620862	0.426388	-2533.194474	NO		-19.08
F_88	-2533.621924	0.427450	-2533.194474	NO		-19.08
F_89	-2533.619777	0.425379	-2533.194398	NO		-19.03
F_90	-2533.617760	0.423453	-2533.194307	NO		-18.98
F_91	-2533.619681	0.425426	-2533.194255	NO		-18.95
F_92	-2533.619691	0.425463	-2533.194228	NO		-18.93
F_93	-2533.621564	0.427406	-2533.194158	NO		-18.88
F_94	-2533.619131	0.425007	-2533.194124	NO		-18.86
F_95	-2533.620936	0.426825	-2533.194111	NO		-18.85
F_96	-2533.619274	0.425350	-2533.193924	NO		-18.74
F_97	-2533.619362	0.425451	-2533.193911	NO		-18.73

F_98	-2533.620143	0.426274	-2533.193869	NO		-18.70
F_99	-2533.619804	0.425947	-2533.193857	NO		-18.70
F_100	-2533.619312	0.425556	-2533.193756	NO		-18.63
F_101	-2533.621121	0.427386	-2533.193735	NO		-18.62
F_102	-2533.620591	0.426949	-2533.193642	NO		-18.56
F_103	-2533.621836	0.428206	-2533.193630	NO		-18.55
F_104	-2533.617845	0.424245	-2533.193600	NO		-18.53
F_105	-2533.620601	0.427012	-2533.193589	NO		-18.53
F_106	-2533.620543	0.426958	-2533.193585	NO		-18.52
F_107	-2533.619641	0.426124	-2533.193517	NO		-18.48
F_108	-2533.620270	0.426758	-2533.193512	NO		-18.48
F_109	-2533.618340	0.425063	-2533.193277	NO		-18.33
F_110	-2533.618447	0.425178	-2533.193269	NO		-18.33
F_111	-2533.618340	0.425076	-2533.193264	NO		-18.32
F_112	-2533.618882	0.425635	-2533.193247	NO		-18.31
F_113	-2533.620206	0.426968	-2533.193238	NO		-18.31
F_114	-2533.615770	0.422548	-2533.193222	NO		-18.30
F_115	-2533.617603	0.424400	-2533.193203	NO		-18.29
F_116	-2533.615793	0.422624	-2533.193169	NO		-18.26
F_117	-2533.617453	0.424297	-2533.193156	NO		-18.26
F_118	-2533.618728	0.425629	-2533.193099	NO		-18.22
F_119	-2533.618728	0.425630	-2533.193098	NO		-18.22
F_120	-2533.620135	0.427249	-2533.192886	NO		-18.09
F_121	-2533.619982	0.427133	-2533.192849	NO		-18.06

F_122	-2533.619085	0.426252	-2533.192833	NO		-18.05
F_123	-2533.621335	0.428526	-2533.192809	NO		-18.04
F_124	-2533.620034	0.427278	-2533.192756	NO		-18.00
F_125	-2533.617826	0.425316	-2533.192510	NO		-17.85
F_126	-2533.620385	0.427917	-2533.192468	NO		-17.82
F_127	-2533.617365	0.425073	-2533.192292	NO		-17.71
F_128	-2533.620514	0.428295	-2533.192219	NO		-17.67
F_129	-2533.618906	0.426903	-2533.192003	NO		-17.53
F_130	-2533.617383	0.425461	-2533.191922	NO		-17.48
F_131	-2533.620853	0.428951	-2533.191902	NO		-17.47
F_132	-2533.620099	0.428294	-2533.191805	NO		-17.41
F_133	-2533.618943	0.427146	-2533.191797	NO		-17.40
F_134	-2533.620085	0.428326	-2533.191759	NO		-17.38
F_135	-2533.616756	0.425074	-2533.191682	NO		-17.33
F_136	-2533.614915	0.423327	-2533.191588	NO		-17.27
F_137	-2533.617941	0.426776	-2533.191165	NO		-17.01
F_138	-2533.616393	0.425354	-2533.191039	NO		-16.93
F_139	-2533.615592	0.424562	-2533.191030	NO		-16.92
F_140	-2533.617653	0.426693	-2533.190960	NO		-16.88
F_141	-2533.616539	0.426030	-2533.190509	NO		-16.59
F_142	-2533.615824	0.425517	-2533.190307	NO		-16.47
F_143	-2533.617017	0.426766	-2533.190251	NO		-16.43
F_144	-2533.616984	0.426741	-2533.190243	NO		-16.43
F_145	-2533.616389	0.426213	-2533.190176	NO		-16.39

F_146	-2533.614402	0.424388	-2533.190014	NO		-16.28
F_147	-2533.616045	0.426436	-2533.189609	NO		-16.03
F_148	-2533.613831	0.424243	-2533.189588	NO		-16.02
F_149	-2533.615110	0.425636	-2533.189474	NO		-15.94
F_150	-2533.614573	0.425432	-2533.189141	NO		-15.74
**TS_D-E_1**	**-2533.548970**	**0.419681**	**-2533.12928862**	**-181.955600**		**21.82**
TS_D-E_2	-2533.548970	0.419722	-2533.1292477	-181.838200		21.85
TS_D-E_3	-2533.549039	0.420377	-2533.12866162	-180.074800		22.22
TS_D-E_4	-2533.547056	0.418713	-2533.12834276	-173.439100		22.42
TS_D-E_5	-2533.547056	0.418716	-2533.12833966	-173.493800		22.42
TS_D-E_6	-2533.548043	0.419927	-2533.12811627	-171.816400		22.56
TS_D-E_7	-2533.548043	0.419928	-2533.12811527	-171.907100		22.56
TS_D-E_8	-2533.547897	0.420353	-2533.12754367	-191.356600		22.92
TS_D-E_9	-2533.547897	0.420356	-2533.127541	-191.273200		22.92
TS_D-E_10	-2533.548332	0.420897	-2533.127435	-193.994200		22.98
TS_D-E_11	-2533.547747	0.420367	-2533.127380	-182.715000		23.02
TS_D-E_12	-2533.547386	0.420092	-2533.127294	-188.829600		23.07
TS_D-E_13	-2533.547173	0.419975	-2533.127198	-178.804800		23.13
TS_D-E_14	-2533.549452	0.422512	-2533.126940	-198.752600		23.30
TS_D-E_15	-2533.546941	0.421691	-2533.125250	-171.610200		24.36
TS_D-E_16	-2533.546627	0.421902	-2533.124725	-197.639500		24.69
TS_D-E_17	-2533.546817	0.423016	-2533.123801	-177.563300		25.27
TS_D-E_18	-2533.546037	0.423107	-2533.122930	-174.230300		25.81

TS_D-E_19	-2533.540638	0.420173	-2533.120465	-35.530400		27.36
TS_D-E_20	-2533.535321	0.416527	-2533.118794	-129.316700		28.41
**E_1**	**-2533.589091**	**0.421138**	**-2533.167953**	**NO**		**-2.44**
E_2	-2533.587677	0.420144	-2533.167533	NO		-2.18
E_3	-2533.588614	0.421088	-2533.167526	NO		-2.17
E_4	-2533.587153	0.420454	-2533.166699	NO		-1.65
E_5	-2533.589345	0.422682	-2533.166663	NO		-1.63
E_6	-2533.587965	0.421334	-2533.166631	NO		-1.61
E_7	-2533.588067	0.421492	-2533.166575	NO		-1.58
E_8	-2533.588447	0.421971	-2533.166476	NO		-1.51
E_9	-2533.586581	0.420113	-2533.166468	NO		-1.51
E_10	-2533.586608	0.420225	-2533.166383	NO		-1.46
E_11	-2533.585895	0.419783	-2533.166112	NO		-1.28
E_12	-2533.587850	0.421851	-2533.165999	NO		-1.21
E_13	-2533.586572	0.420613	-2533.165959	NO		-1.19
E_14	-2533.588939	0.423060	-2533.165879	NO		-1.14
E_15	-2533.587840	0.422080	-2533.165760	NO		-1.06
E_16	-2533.587913	0.422156	-2533.165757	NO		-1.06
E_17	-2533.588035	0.422282	-2533.165753	NO		-1.06
E_18	-2533.586464	0.420787	-2533.165677	NO		-1.01
E_19	-2533.589714	0.424302	-2533.165412	NO		-0.85
E_20	-2533.586163	0.420947	-2533.165216	NO		-0.72
E_21	-2533.588983	0.423858	-2533.165125	NO		-0.67

E_22	-2533.588984	0.423919	-2533.165065	NO		-0.63
E_23	-2533.587741	0.422681	-2533.165060	NO		-0.62
E_24	-2533.588000	0.422946	-2533.165054	NO		-0.62
E_25	-2533.587390	0.422417	-2533.164973	NO		-0.57
E_26	-2533.587965	0.423044	-2533.164921	NO		-0.54
E_27	-2533.587876	0.422958	-2533.164918	NO		-0.54
E_28	-2533.587561	0.422647	-2533.164914	NO		-0.53
E_29	-2533.585153	0.420250	-2533.164903	NO		-0.53
E_30	-2533.587395	0.422511	-2533.164884	NO		-0.51
E_31	-2533.584912	0.420045	-2533.164867	NO		-0.50
E_32	-2533.587395	0.422535	-2533.164860	NO		-0.50
E_33	-2533.588020	0.423207	-2533.164813	NO		-0.47
E_34	-2533.587577	0.422803	-2533.164774	NO		-0.45
E_35	-2533.585972	0.421227	-2533.164745	NO		-0.43
E_36	-2533.586181	0.421451	-2533.164730	NO		-0.42
E_37	-2533.587366	0.422647	-2533.164719	NO		-0.41
E_38	-2533.588068	0.423366	-2533.164702	NO		-0.40
E_39	-2533.588068	0.423368	-2533.164700	NO		-0.40
E_40	-2533.589599	0.424921	-2533.164678	NO		-0.39
E_41	-2533.588530	0.423865	-2533.164665	NO		-0.38
E_42	-2533.586398	0.421774	-2533.164624	NO		-0.35
E_43	-2533.585485	0.420920	-2533.164565	NO		-0.31
E_44	-2533.589237	0.424673	-2533.164564	NO		-0.31
E_45	-2533.585560	0.421013	-2533.164547	NO		-0.30

E_46	-2533.587834	0.423307	-2533.164527	NO		-0.29
E_47	-2533.588085	0.423561	-2533.164524	NO		-0.29
E_48	-2533.586844	0.422328	-2533.164516	NO		-0.28
E_49	-2533.586567	0.422082	-2533.164485	NO		-0.26
E_50	-2533.587092	0.422615	-2533.164477	NO		-0.26
E_51	-2533.586955	0.422483	-2533.164472	NO		-0.26
E_52	-2533.588291	0.423828	-2533.164463	NO		-0.25
E_53	-2533.586890	0.422475	-2533.164415	NO		-0.22
E_54	-2533.586890	0.422481	-2533.164409	NO		-0.22
E_55	-2533.586118	0.421737	-2533.164381	NO		-0.20
E_56	-2533.588564	0.424197	-2533.164367	NO		-0.19
E_57	-2533.585328	0.420981	-2533.164347	NO		-0.18
E_58	-2533.588914	0.424595	-2533.164319	NO		-0.16
E_59	-2533.588182	0.423864	-2533.164318	NO		-0.16
E_60	-2533.586890	0.422578	-2533.164312	NO		-0.16
E_61	-2533.587794	0.423526	-2533.164268	NO		-0.13
E_62	-2533.588372	0.424114	-2533.164258	NO		-0.12
E_63	-2533.587779	0.423537	-2533.164242	NO		-0.11
E_64	-2533.586954	0.422715	-2533.164239	NO		-0.11
E_65	-2533.586086	0.421849	-2533.164237	NO		-0.11
E_66	-2533.585483	0.421254	-2533.164229	NO		-0.10
E_67	-2533.586829	0.422602	-2533.164227	NO		-0.10
E_68	-2533.587923	0.423696	-2533.164227	NO		-0.10
E_69	-2533.589352	0.425128	-2533.164224	NO		-0.10

E_70	-2533.587202	0.422990	-2533.164212	NO		-0.09
E_71	-2533.587374	0.423182	-2533.164192	NO		-0.08
E_72	-2533.586499	0.422324	-2533.164175	NO		-0.07
E_73	-2533.588763	0.424611	-2533.164152	NO		-0.06
E_74	-2533.588764	0.424617	-2533.164147	NO		-0.05
E_75	-2533.584986	0.420905	-2533.164081	NO		-0.01
E_76	-2533.587907	0.423877	-2533.164030	NO		0.02
E_77	-2533.587395	0.423409	-2533.163986	NO		0.05
E_78	-2533.588088	0.424107	-2533.163981	NO		0.05
E_79	-2533.587531	0.423567	-2533.163964	NO		0.06
E_80	-2533.586975	0.423023	-2533.163952	NO		0.07
E_81	-2533.587101	0.423170	-2533.163931	NO		0.08
E_82	-2533.586805	0.422879	-2533.163926	NO		0.09
E_83	-2533.586805	0.422890	-2533.163915	NO		0.09
E_84	-2533.590833	0.426947	-2533.163886	NO		0.11
E_85	-2533.588943	0.425057	-2533.163886	NO		0.11
E_86	-2533.585334	0.421493	-2533.163841	NO		0.14
E_87	-2533.585918	0.422090	-2533.163828	NO		0.15
E_88	-2533.586479	0.422669	-2533.163810	NO		0.16
E_89	-2533.586670	0.422868	-2533.163802	NO		0.16
E_90	-2533.585958	0.422187	-2533.163771	NO		0.18
E_91	-2533.586418	0.422712	-2533.163706	NO		0.22
E_92	-2533.588330	0.424633	-2533.163697	NO		0.23
E_93	-2533.586546	0.422862	-2533.163684	NO		0.24

E_94	-2533.585939	0.422261	-2533.163678	NO		0.24
E_95	-2533.587811	0.424166	-2533.163645	NO		0.26
E_96	-2533.587127	0.423556	-2533.163571	NO		0.31
E_97	-2533.587279	0.423715	-2533.163564	NO		0.31
E_98	-2533.586264	0.422706	-2533.163558	NO		0.32
E_99	-2533.587143	0.423667	-2533.163476	NO		0.37
E_100	-2533.586217	0.422826	-2533.163391	NO		0.42
E_101	-2533.587865	0.424484	-2533.163381	NO		0.43
E_102	-2533.586477	0.423102	-2533.163375	NO		0.43
E_103	-2533.586237	0.422925	-2533.163312	NO		0.47
E_104	-2533.585969	0.422662	-2533.163307	NO		0.47
E_105	-2533.586216	0.422917	-2533.163299	NO		0.48
E_106	-2533.587663	0.424423	-2533.163240	NO		0.52
E_107	-2533.587038	0.423824	-2533.163214	NO		0.53
E_108	-2533.588358	0.425148	-2533.163210	NO		0.54
E_109	-2533.586223	0.423066	-2533.163157	NO		0.57
E_110	-2533.586060	0.422961	-2533.163099	NO		0.61
E_111	-2533.587146	0.424070	-2533.163076	NO		0.62
E_112	-2533.586394	0.423454	-2533.162940	NO		0.71
E_113	-2533.586404	0.423465	-2533.162939	NO		0.71
E_114	-2533.585123	0.422240	-2533.162883	NO		0.74
E_115	-2533.586124	0.423315	-2533.162809	NO		0.79
E_116	-2533.585354	0.422597	-2533.162757	NO		0.82
E_117	-2533.585947	0.423228	-2533.162719	NO		0.84

E_118	-2533.585947	0.423241	-2533.162706	NO		0.85
E_119	-2533.584498	0.421797	-2533.162701	NO		0.86
E_120	-2533.584374	0.421775	-2533.162599	NO		0.92
E_121	-2533.587733	0.425170	-2533.162563	NO		0.94
E_122	-2533.586505	0.424035	-2533.162470	NO		1.00
E_123	-2533.586443	0.424035	-2533.162408	NO		1.04
E_124	-2533.586965	0.424566	-2533.162399	NO		1.04
E_125	-2533.588398	0.426013	-2533.162385	NO		1.05
E_126	-2533.587851	0.425505	-2533.162346	NO		1.08
E_127	-2533.586163	0.423825	-2533.162338	NO		1.08
E_128	-2533.584582	0.422267	-2533.162315	NO		1.10
E_129	-2533.585479	0.423201	-2533.162278	NO		1.12
E_130	-2533.586695	0.424423	-2533.162272	NO		1.12
E_131	-2533.585559	0.423292	-2533.162267	NO		1.13
E_132	-2533.584984	0.422740	-2533.162244	NO		1.14
E_133	-2533.587276	0.425036	-2533.162240	NO		1.14
E_134	-2533.584697	0.422520	-2533.162177	NO		1.18
E_135	-2533.583822	0.421661	-2533.162161	NO		1.19
E_136	-2533.586600	0.424448	-2533.162152	NO		1.20
E_137	-2533.584269	0.422132	-2533.162137	NO		1.21
E_138	-2533.586012	0.423905	-2533.162107	NO		1.23
E_139	-2533.586299	0.424264	-2533.162035	NO		1.27
E_140	-2533.583232	0.421216	-2533.162016	NO		1.29
E_141	-2533.587020	0.425025	-2533.161995	NO		1.30

E_142	-2533.585909	0.423917	-2533.161992	NO		1.30
E_143	-2533.581881	0.419900	-2533.161981	NO		1.31
E_144	-2533.586164	0.424214	-2533.161950	NO		1.33
E_145	-2533.583231	0.421332	-2533.161899	NO		1.36
E_146	-2533.586914	0.425036	-2533.161878	NO		1.37
E_147	-2533.585550	0.423686	-2533.161864	NO		1.38
E_148	-2533.585550	0.423697	-2533.161853	NO		1.39
E_149	-2533.586243	0.424458	-2533.161785	NO		1.43
E_150	-2533.586334	0.424556	-2533.161778	NO		1.43
E_151	-2533.586730	0.424995	-2533.161735	NO		1.46
E_152	-2533.583166	0.421435	-2533.161731	NO		1.46
E_153	-2533.585501	0.423908	-2533.161593	NO		1.55
E_154	-2533.585778	0.424213	-2533.161565	NO		1.57
E_155	-2533.584053	0.422558	-2533.161495	NO		1.61
E_156	-2533.585661	0.424341	-2533.161320	NO		1.72
E_157	-2533.584102	0.422801	-2533.161301	NO		1.73
E_158	-2533.588043	0.426806	-2533.161237	NO		1.77
E_159	-2533.585391	0.424187	-2533.161204	NO		1.79
E_160	-2533.586234	0.425035	-2533.161199	NO		1.80
E_161	-2533.585033	0.423983	-2533.161050	NO		1.89
E_162	-2533.584442	0.423405	-2533.161037	NO		1.90
E_163	-2533.583411	0.422386	-2533.161025	NO		1.91
E_164	-2533.585490	0.424512	-2533.160978	NO		1.94
E_165	-2533.584409	0.423597	-2533.160812	NO		2.04

E_166	-2533.586061	0.425254	-2533.160807	NO		2.04
E_167	-2533.585353	0.424554	-2533.160799	NO		2.05
E_168	-2533.586450	0.425651	-2533.160799	NO		2.05
E_169	-2533.585072	0.424316	-2533.160756	NO		2.08
E_170	-2533.586325	0.425640	-2533.160685	NO		2.12
E_171	-2533.586291	0.425779	-2533.160512	NO		2.23
E_172	-2533.586914	0.426406	-2533.160508	NO		2.23
E_173	-2533.585777	0.425332	-2533.160445	NO		2.27
E_174	-2533.586474	0.426306	-2533.160168	NO		2.44
E_175	-2533.585210	0.425054	-2533.160156	NO		2.45
E_176	-2533.582310	0.422155	-2533.160155	NO		2.45
E_177	-2533.585756	0.425700	-2533.160056	NO		2.52
E_178	-2533.585537	0.425828	-2533.159709	NO		2.73
E_179	-2533.584207	0.424769	-2533.159438	NO		2.90
E_180	-2533.583231	0.423806	-2533.159425	NO		2.91
E_181	-2533.582993	0.423569	-2533.159424	NO		2.91
E_182	-2533.586155	0.427002	-2533.159153	NO		3.08
E_183	-2533.583807	0.424677	-2533.159130	NO		3.10
E_184	-2533.583807	0.424678	-2533.159129	NO		3.10
E_185	-2533.583885	0.425117	-2533.158768	NO		3.32
E_186	-2533.584821	0.426316	-2533.158505	NO		3.49
E_187	-2533.585866	0.427389	-2533.158477	NO		3.51
E_188	-2533.584624	0.426617	-2533.158007	NO		3.80
E_189	-2533.582352	0.424555	-2533.157797	NO		3.93

Table S12. Total energies (Hartree), Gibbs free energies (Hartree), relative free energies (kcal mol^{-1}), the magnitudes of the imaginary frequencies for the transition states (cm^{-1}) for the calculations shown in Fig 10.

Level of theory	*System*	*Etot*	*Gcorr*	*G*	*Imaginary freq.*	*G,rel*
PBE0-D3/def2-SVP	TF2NH_1	-1825.425070	0.023285	-1825.401785	NO	
PBE0-D3/def2-SVP	TF2NH_2	-1825.425070	0.023286	-1825.401784	NO	
PBE0-D3/def2-SVP	TF2NH_3	-1825.425070	0.023294	-1825.401776	NO	
PBE0-D3/def2-SVP	TF2NH_4	-1825.425070	0.023296	-1825.401774	NO	
PBE0-D3/def2-SVP	TF2NH_5	-1825.422688	0.023014	-1825.399674	NO	
PBE0-D3/def2-SVP	TF2NH_6	-1825.422689	0.023023	-1825.399666	NO	
PBE0-D3/def2-SVP	TF2NH_7	-1825.422688	0.023063	-1825.399625	NO	
**SCS-RI-MP2-COSMO/def2-QZVP//RI-MP2/def2-TZVP**	**TF2NH_1**	**-1825.880006**	**0.024094**	**-1825.855912**	**NO**	
PBE0-D3/def2-SVP	Tf2N-_1	-1824.980675	0.011433	-1824.969242	NO	
PBE0-D3/def2-SVP	Tf2N-_2	-1824.980675	0.011457	-1824.969218	NO	
PBE0-D3/def2-SVP	Tf2N-_3	-1824.980793	0.012401	-1824.968392	NO	
PBE0-D3/def2-SVP	Tf2N-_4	-1824.980793	0.012403	-1824.968390	NO	
PBE0-D3/def2-SVP	Tf2N-_5	-1824.980793	0.012403	-1824.968390	NO	
PBE0-D3/def2-SVP	Tf2N-_6	-1824.980793	0.012407	-1824.968386	NO	
**SCS-RI-MP2-COSMO/def2-QZVP//RI-MP2/def2-TZVP**	**TF2N-_1**	**-1825.452293**	**0.012066**	**-1825.440227**	**NO**	
	**X=H**					
**SCS-RI-MP2-COSMO/def2-QZVP//RI-MP2/def2-TZVP**	**G_1**	**-1296.026109**	**0.259444**	**-1295.766666**	**NO**	**0.00**
**SCS-RI-MP2-COSMO/def2-QZVP//RI-MP2/def2-TZVP**	**G_1 + TF2N-_1**			**-3121.206892**		
**SCS-RI-MP2-COSMO/def2-QZVP//RI-MP2/def2-TZVP**	**TS_G-I1_1**	**-1296.014320**	**0.258682**	**-1295.755638**	**-411.45**	**6.92**

**SCS-RI-MP2-COSMO/def2-QZVP//RI-MP2/def2-TZVP**	**I1**	**-1296.018784**	**0.260484**	**-1295.758300**	**NO**	**5.25**
**SCS-RI-MP2-COSMO/def2-QZVP//RI-MP2/def2-TZVP**	**TS_I1_H**	**-1296.015834**	**0.260465**	**-1295.755369**	**-221.03**	**7.09**
B3LYP-D3/def2-TZVP	H_1	-1298.421179	0.258422	-1298.162757	NO	
B3LYP-D3/def2-TZVP	H_2	-1298.419152	0.260844	-1298.158308	NO	
B3LYP-D3/def2-TZVP	H_3	-1298.418816	0.260295	-1298.158521	NO	
**SCS-RI-MP2-COSMO/def2-QZVP//RI-MP2/def2-TZVP**	**H_1**	**-1296.055832**	0.260647	**-1295.795185**	**NO**	**-17.90**
PBE0-D3/def2-SVP	19_1	-1295.71393804	0.250538	-1295.463400	NO	
PBE0-D3/def2-SVP	19_2	-1295.711240	0.248692	-1295.462548	NO	
PBE0-D3/def2-SVP	19_3	-1295.713142	0.250603	-1295.462539	NO	
PBE0-D3/def2-SVP	19_4	-1295.711810	0.249451	-1295.462359	NO	
PBE0-D3/def2-SVP	19_5	-1295.710307	0.249225	-1295.461082	NO	
PBE0-D3/def2-SVP	19_6	-1295.711580	0.251116	-1295.460464	NO	
**SCS-RI-MP2-COSMO/def2-QZVP//RI-MP2/def2-TZVP**	**19_1**	**-1295.682776**	**0.248425**	**-1295.434351**	**NO**	
**SCS-RI-MP2-COSMO/def2-QZVP//RI-MP2/def2-TZVP**	**19_1 + TF2NH_1**			**-3121.290263**		**-52.32**
**SCS-RI-MP2-COSMO/def2-QZVP//RI-MP2/def2-TZVP**	**TS_I1-I**	**-1296.014286**	**0.259379**	**-1295.754907**	**-111.1**	**7.38**
B3LYP-D3/6-31G(d)	I_1	-1298.026596	0.260923	-1297.765673	NO	
B3LYP-D3/6-31G(d)	I_2	-1298.026596	0.260929	-1297.765667	NO	
B3LYP-D3/6-31G(d)	I_3	-1298.024661	0.262298	-1297.762363	NO	
B3LYP-D3/6-31G(d)	I_4	-1298.024707	0.262893	-1297.761814	NO	
B3LYP-D3/6-31G(d)	I_5	-1298.024707	0.262898	-1297.761809	NO	
B3LYP-D3/6-31G(d)	I_6	-1298.019728	0.262449	-1297.757279	NO	
**SCS-RI-MP2-COSMO/def2-QZVP//RI-MP2/def2-TZVP**	**I_1**	**-1296.046679**	**0.260141**	**-1295.786538**	**NO**	**-12.47**

**SCS-RI-MP2-COSMO/def2-QZVP//RI-MP2/def2-TZVP**	**TS_I-H1**	**-1296.026057**	**0.260065**	**-1295.765992**	**-185.45**	**0.42**
B3LYP-D3/6-31G(d)	H1_1	-1298.026596	0.260923	-1297.765673	NO	
B3LYP-D3/6-31G(d)	H1_2	-1298.026596	0.260929	-1297.765667	NO	
B3LYP-D3/6-31G(d)	H1_3	-1298.024661	0.262298	-1297.762363	NO	
B3LYP-D3/6-31G(d)	H1_4	-1298.024707	0.262893	-1297.761814	NO	
B3LYP-D3/6-31G(d)	H1_5	-1298.024707	0.262898	-1297.761809	NO	
B3LYP-D3/6-31G(d)	H1_6	-1298.019728	0.262449	-1297.757279	NO	
**SCS-RI-MP2-COSMO/def2-QZVP//RI-MP2/def2-TZVP**	**H1**	**-1296.053632**	**0.262320**	**-1295.791312**	**NO**	**-15.47**
PBE0-D3/def2-SVP	18_1	-1295.710163	0.248740	-1295.461423	NO	
PBE0-D3/def2-SVP	18_2	-1295.710163	0.248755	-1295.461408	NO	
PBE0-D3/def2-SVP	18_3	-1295.711118	0.249790	-1295.461328	NO	
PBE0-D3/def2-SVP	18_4	-1295.709356	0.248494	-1295.460862	NO	
PBE0-D3/def2-SVP	18_5	-1295.709556	0.248803	-1295.460753	NO	
PBE0-D3/def2-SVP	18_6	-1295.709557	0.248814	-1295.460743	NO	
PBE0-D3/def2-SVP	18_7	-1295.710763	0.250054	-1295.460709	NO	
PBE0-D3/def2-SVP	18_8	-1295.708853	0.248326	-1295.460527	NO	
PBE0-D3/def2-SVP	18_9	-1295.708252	0.247771	-1295.460481	NO	
PBE0-D3/def2-SVP	18_10	-1295.710125	0.249805	-1295.460320	NO	
PBE0-D3/def2-SVP	18_11	-1295.707878	0.248017	-1295.459861	NO	
PBE0-D3/def2-SVP	18_12	-1295.709465	0.249750	-1295.459715	NO	
PBE0-D3/def2-SVP	18_13	-1295.708710	0.249521	-1295.459189	NO	
PBE0-D3/def2-SVP	18_14	-1295.708100	0.249082	-1295.459018	NO	
PBE0-D3/def2-SVP	18_15	-1295.707651	0.249509	-1295.458142	NO	
PBE0-D3/def2-SVP	18_16	-1295.707157	0.249909	-1295.457248	NO	
PBE0-D3/def2-SVP	18_17	-1295.704692	0.251121	-1295.453571	NO	
**SCS-RI-MP2-COSMO/def2-QZVP//RI-MP2/def2-TZVP**	**18_1**	**-1295.680597**	**0.247568**	**-1295.433029**	**NO**	

**SCS-RI-MP2-COSMO/def2-QZVP//RI-MP2/def2-TZVP**	**18_1 + TF2NH_1**			**-3121.288941**		**-51.49**
	**X=Cl**					
**SCS-RI-MP2-COSMO/def2-QZVP//RI-MP2/def2-TZVP**	**G_1**	**-1755.1576680172**	**0.247492**	**-1754.910176**	**NO**	**0.00**
**SCS-RI-MP2-COSMO/def2-QZVP//RI-MP2/def2-TZVP**	**G_1 + TF2N-_1**			**-3580.350403**		**0.00**
**SCS-RI-MP2-COSMO/def2-QZVP//RI-MP2/def2-TZVP**	**TS_G-I1_1**	**-1755.1456367696**	**0.246703**	**-1754.898933**	**-417.78**	**7.05**
**SCS-RI-MP2-COSMO/def2-QZVP//RI-MP2/def2-TZVP**	**I1**	**-1755.1496333513**	**0.2486078842**	**-1754.901025**	**NO**	**5.74**
**SCS-RI-MP2-COSMO/def2-QZVP//RI-MP2/def2-TZVP**	**TS_I1_H**	**-1755.144012**	**0.248075**	**-1754.895938**	**-277.56**	**8.93**
B3LYP-D3/def2-TZVP	H_1	-1758.046255	0.249770	-1757.796485	NO	
B3LYP-D3/def2-TZVP	H_2	-1758.042713	0.249296	-1757.793417	NO	
B3LYP-D3/def2-TZVP	H_3	-1758.044642	0.250075	-1757.794567	NO	
B3LYP-D3/def2-TZVP	H_4	-1758.043906	0.248227	-1757.795679	NO	
B3LYP-D3/def2-TZVP	H_5	-1758.044230	0.248664	-1757.795566	NO	
B3LYP-D3/def2-TZVP	H_6	-1758.040386	0.249735	-1757.790651	NO	
**SCS-RI-MP2-COSMO/def2-QZVP//RI-MP2/def2-TZVP**	**H_1**	**-1755.188277**	**0.249930**	**-1754.938347**	**NO**	**-17.68**
PBE0-D3/def2-SVP	19_1	-1755.021546	0.238781	-1754.782765	NO	
PBE0-D3/def2-SVP	19_2	-1755.019942	0.238117	-1754.781825	NO	
PBE0-D3/def2-SVP	19_3	-1755.021181	0.240604	-1754.780577	NO	
PBE0-D3/def2-SVP	19_4	-1755.019559	0.239238	-1754.780321	NO	
PBE0-D3/def2-SVP	19_5	-1755.015525	0.237646	-1754.777879	NO	
PBE0-D3/def2-SVP	19_6	-1755.013883	0.236543	-1754.777340	NO	
PBE0-D3/def2-SVP	19_7	-1755.015317	0.239062	-1754.776255	NO	

PBE0-D3/def2-SVP	19_8	-1755.013085	0.237639	-1754.775446	NO	
PBE0-D3/def2-SVP	19_9	-1755.012140	0.238198	-1754.773942	NO	
PBE0-D3/def2-SVP	19_1	-1754.8150286534	0.237646	-1754.577382	NO	
**SCS-RI-MP2-COSMO/def2-QZVP//RI-MP2/def2-TZVP**	**19_1 + TF2NH_1**			**-3580.433294**		**-52.02**
**SCS-RI-MP2-COSMO/def2-QZVP//RI-MP2/def2-TZVP**	**TS_I1-I**	**-1755.145669**	**0.247321**	**-1754.898348**	**-91.44**	**7.42**
B3LYP-D3/6-31G(d)	I_1	-1757.622878	0.249816	-1757.373062	NO	
B3LYP-D3/6-31G(d)	I_2	-1757.622878	0.249846	-1757.373032	NO	
B3LYP-D3/6-31G(d)	I_3	-1757.620483	0.249787	-1757.370696	NO	
B3LYP-D3/6-31G(d)	I_4	-1757.620484	0.249798	-1757.370686	NO	
B3LYP-D3/6-31G(d)	I_5	-1757.620398	0.250972	-1757.369426	NO	
B3LYP-D3/6-31G(d)	I_6	-1757.615584	0.251953	-1757.363631	NO	
**SCS-RI-MP2-COSMO/def2-QZVP//RI-MP2/def2-TZVP**	**I_1**	**-1755.182760**	**0.248357**	**-1754.934403**	**NO**	**-15.20**
**SCS-RI-MP2-COSMO/def2-QZVP//RI-MP2/def2-TZVP**	**TS_I-H1**	**-1755.159666**	**0.2483565035**	**-1754.911309**		**-0.71**
B3LYP-D3/def2-TZVP	H1_1	-1758.04097192	0.246294	-1757.794678	NO	
B3LYP-D3/def2-TZVP	H1_2	-1758.04211503	0.247749	-1757.794366	NO	
B3LYP-D3/def2-TZVP	H1_3	-1758.04162646	0.247489	-1757.794137	NO	
**SCS-RI-MP2-COSMO/def2-QZVP//RI-MP2/def2-TZVP**	**H1_1**	**-1755.182598**	**0.248547**	**-1754.934051**	**NO**	**-14.98**
PBE0-D3/def2-SVP	18_1	-1755.020992	0.237126	-1754.783866	NO	
PBE0-D3/def2-SVP	18_2	-1755.020234	0.236543	-1754.783691	NO	
PBE0-D3/def2-SVP	18_3	-1755.020231	0.236690	-1754.783541	NO	
PBE0-D3/def2-SVP	18_4	-1755.018227	0.236188	-1754.782039	NO	
PBE0-D3/def2-SVP	18_5	-1755.020027	0.238007	-1754.782020	NO	
PBE0-D3/def2-SVP	18_6	-1755.020115	0.238183	-1754.781932	NO	

PBE0-D3/def2-SVP	18_7	-1755.019112	0.237335	-1754.781777	NO	
PBE0-D3/def2-SVP	18_8	-1755.018095	0.236358	-1754.781737	NO	
PBE0-D3/def2-SVP	18_9	-1755.018762	0.237490	-1754.781272	NO	
PBE0-D3/def2-SVP	18_10	-1755.018359	0.237270	-1754.781089	NO	
PBE0-D3/def2-SVP	18_11	-1755.017877	0.236898	-1754.780979	NO	
PBE0-D3/def2-SVP	18_12	-1755.018649	0.237854	-1754.780795	NO	
PBE0-D3/def2-SVP	18_13	-1755.018838	0.238297	-1754.780541	NO	
PBE0-D3/def2-SVP	18_14	-1755.017796	0.237911	-1754.779885	NO	
PBE0-D3/def2-SVP	18_15	-1755.016747	0.238755	-1754.777992	NO	
PBE0-D3/def2-SVP	18_16	-1755.014145	0.238021	-1754.776124	NO	
PBE0-D3/def2-SVP	18_17	-1755.014324	0.239204	-1754.775120	NO	
PBE0-D3/def2-SVP	18_18	-1755.013801	0.238977	-1754.774824	NO	
**SCS-RI-MP2-COSMO/def2-QZVP//RI-MP2/def2-TZVP**	**18_1**	**-1754.816028**	**0.236176**	**-1754.579852**	**NO**	
**SCS-RI-MP2-COSMO/def2-QZVP//RI-MP2/def2-TZVP**	**18_1 + TF2NH_1**			**-3580.435764**		**-53.57**

8.4.Cartesian coordinates

Cartesian coordinates for the calculations shown in Fig 6-9 (the most stable ($\Delta G_{298,\mathrm{DCM}}$) conformations as computed at the B3LYP-D3-SMD/6-31+G(d,p) level of theory

Alk_A

O 0.451369 -0.011089 1.735669

C 2.504677 -0.962418 0.334175

C 3.170203 -0.479646 -0.796025

C 3.183674 -1.504023 1.432106

C 4.565431 -0.547779 -0.823999

C 4.575006 -1.556523 1.388653

C 5.262386 -1.081421 0.263397

H 2.611926 -0.074171 -1.632612

H 2.640028 -1.870373 2.296734

H 5.101932 -0.181265 -1.693448

H 5.122835 -1.966939 2.231095

H 6.347069 -1.127773 0.237922

C 0.161169 -2.460073 0.770294

C 0.758743 -3.569400 0.166123

C -0.934496 -2.579140 1.628238

C 0.250652 -4.837561 0.453155

C -1.423545 -3.857390 1.908124

C -0.835385 -4.982629 1.322752

H 1.595104 -3.453154 -0.513095

H	-1.393184	-1.703851	2.071628
H	0.707589	-5.709327	-0.004809
H	-2.268024	-3.966985	2.581535
H	-1.223699	-5.972634	1.542846
S	0.728244	-0.801947	0.339186
C	0.702943	1.924866	-2.838747
C	-0.024551	0.955152	-2.871208
C	-2.293911	-0.008284	-3.249731
C	-0.458180	-1.403035	-2.736385
C	-2.756110	-1.429457	-2.898300
H	-2.406192	0.227534	-4.312621
H	-2.784565	0.751282	-2.640534
H	-3.227382	-1.473499	-1.915637
H	-3.395762	-1.879247	-3.657130
N	-0.864517	-0.097745	-2.907702
O	-1.528015	-2.218934	-2.829936
O	0.677955	-1.799584	-2.530821
S	-2.413443	1.302149	0.371753
O	-2.239149	-0.105464	-0.048501
O	-2.981067	2.231701	-0.618568
O	-1.224651	1.840519	1.121810
H	-0.197973	0.750722	1.527547
C	-3.721023	1.195833	1.717644
F	-4.872689	0.712668	1.215236
F	-3.967229	2.404430	2.253307
F	-3.312481	0.372567	2.703042
C	1.582079	3.094399	-2.763625
H	2.609208	2.771466	-2.983615
H	1.305559	3.794876	-3.561957
C	1.556170	3.824714	-1.403087
H	2.209302	4.703390	-1.487107
H	0.541657	4.196210	-1.211383
C	2.015971	2.953411	-0.230910
H	1.308322	2.125095	-0.104990
H	2.985272	2.502812	-0.478917
C	2.120425	3.730625	1.087472
H	2.860712	4.536288	0.970513
H	1.157382	4.221154	1.284892
C	2.500361	2.869021	2.302807
H	2.481823	3.500811	3.200907
H	1.735050	2.096909	2.450148
C	3.873745	2.196055	2.189065
H	4.663837	2.937592	2.012102
H	4.124725	1.654003	3.108283
H	3.903723	1.474083	1.367189

Alk_TS_A-B

O	-1.369014	-1.427360	1.636537
C	-3.004938	-0.210014	-0.146996
C	-2.111058	-0.280655	-1.220450

C	-4.005452	0.761695	-0.089700
C	-2.230475	0.643742	-2.257918
C	-4.122160	1.677315	-1.142177
C	-3.236154	1.619314	-2.220429
H	-1.334358	-1.037966	-1.243669
H	-4.681065	0.812375	0.759813
H	-1.538706	0.603527	-3.094150
H	-4.892735	2.441491	-1.107746
H	-3.322061	2.338420	-3.029707
C	-3.205293	-2.908533	0.347658
C	-2.184199	-3.843012	0.186978
C	-4.500725	-3.147058	-0.121428
C	-2.463121	-5.039812	-0.482860
C	-4.766459	-4.344573	-0.788652
C	-3.748822	-5.290176	-0.970326
H	-1.192132	-3.635119	0.572446
H	-5.287963	-2.410289	0.016485
H	-1.672771	-5.771956	-0.621237
H	-5.766877	-4.539491	-1.163983
H	-3.961202	-6.220264	-1.489643
S	-2.850911	-1.362075	1.252753
C	1.199991	1.516876	-0.300025
C	1.142556	0.391600	0.229656
C	1.645859	-1.086307	2.154160
C	1.097649	-1.986383	0.007134

C	1.378532	-2.598564	2.219394
H	0.993179	-0.521099	2.818404
H	2.691215	-0.827183	2.327964
H	2.203166	-3.161655	2.654841
H	0.437198	-2.831147	2.716586
N	1.306475	-0.795328	0.746018
O	1.246679	-3.028277	0.828553
O	0.856190	-2.057418	-1.174240
H	2.617525	1.538888	-0.630991
S	4.779875	1.203198	0.075315
O	4.176186	0.933327	1.384688
O	3.731514	1.702574	-0.954046
O	6.015387	1.978717	-0.017311
C	5.199568	-0.492215	-0.626774
F	5.717919	-0.381181	-1.855976
F	4.096438	-1.255273	-0.686441
F	6.095472	-1.090274	0.172818
C	0.448181	2.745431	-0.673655
H	1.134681	3.599664	-0.682907
H	0.107069	2.612358	-1.709039
C	-0.752000	3.028950	0.245023
H	-1.416607	2.158306	0.237836
H	-0.392325	3.150262	1.274886
C	-1.528676	4.275576	-0.187480
H	-0.852659	5.142254	-0.212572

H	-1.890928	4.131205	-1.214951
C	-2.718985	4.583873	0.728385
H	-3.362011	3.695259	0.791927
H	-2.354012	4.775627	1.747932
C	-3.557423	5.777347	0.255888
H	-2.919233	6.670306	0.198586
H	-3.911028	5.584395	-0.766960
C	-4.757229	6.062101	1.165205
H	-4.432967	6.285479	2.189386
H	-5.340192	6.918307	0.806215
H	-5.429976	5.196320	1.212308

Alk_B

O	-1.617763	-1.533511	0.352837
C	-4.173326	-1.787240	-0.434515
C	-4.309761	-2.664622	0.640050
C	-5.204500	-1.583943	-1.355393
C	-5.523000	-3.339982	0.809403
C	-6.412556	-2.262008	-1.173852
C	-6.571634	-3.138868	-0.093934
H	-3.479381	-2.813035	1.323337
H	-5.076628	-0.907446	-2.196398
H	-5.644772	-4.025102	1.643443
H	-7.223903	-2.111534	-1.879934
H	-7.510838	-3.667904	0.039209
C	-3.057261	0.706190	-0.045243
C	-3.148013	0.912061	1.333356
C	-3.276518	1.737545	-0.958733
C	-3.474517	2.184227	1.804327
C	-3.613599	3.006699	-0.475212
C	-3.709931	3.229071	0.900826
H	-2.951896	0.094052	2.019661
H	-3.173264	1.564449	-2.026272
H	-3.541370	2.362686	2.873559
H	-3.779808	3.821035	-1.173610
H	-3.957955	4.219060	1.272057
S	-2.579200	-0.930991	-0.679197
C	1.607755	0.684743	2.040643
C	1.421976	-0.353839	1.293027
C	1.058779	-2.747935	0.904629
C	0.902105	-1.144598	-0.910411
C	0.846503	-3.418459	-0.463069
H	0.207288	-2.874873	1.571344
H	1.989918	-3.044984	1.384644
H	1.704569	-4.013250	-0.772498
H	-0.073924	-3.998759	-0.507006
N	1.153434	-1.319489	0.533417
O	0.711056	-2.336614	-1.432056
O	0.867803	-0.082505	-1.463072
H	2.641409	0.991219	2.200475

S	4.593886	-1.544151	0.159675
O	3.589685	-2.078530	-0.794714
O	4.024136	-1.143243	1.474387
O	5.875770	-2.276634	0.218358
C	5.065139	0.111046	-0.602243
F	3.988144	0.918176	-0.701544
F	5.580300	-0.048729	-1.837274
F	5.986816	0.744252	0.153584
C	0.483692	1.490409	2.658249
H	-0.472852	0.988942	2.489554
H	0.668328	1.519916	3.738311
C	0.440208	2.922064	2.089758
H	-0.337870	3.465164	2.641402
H	1.391445	3.427997	2.301486
C	0.140471	2.975894	0.588151
H	0.941715	2.470087	0.034755
H	-0.777546	2.410644	0.383690
C	-0.017469	4.404456	0.057263
H	0.875820	4.993147	0.313463
H	-0.863603	4.889078	0.566050
C	-0.239114	4.458931	-1.459313
H	0.629992	4.015880	-1.964873
H	-1.099522	3.828037	-1.717892
C	-0.469881	5.879907	-1.982796
H	0.384077	6.530073	-1.754339
H	-0.614589	5.889083	-3.069584
H	-1.359998	6.329550	-1.524239

Alk_TS_B-C

O	1.074656	1.005446	0.952469
C	3.536997	0.200970	0.220964
C	3.906679	0.184132	1.566733
C	4.297108	-0.436225	-0.761188
C	5.079647	-0.479513	1.932307
C	5.474311	-1.089441	-0.383894
C	5.863302	-1.111556	0.958704
H	3.280429	0.668637	2.309396
H	3.976219	-0.436592	-1.799212
H	5.379750	-0.506643	2.975705
H	6.071591	-1.596517	-1.135398
H	6.770741	-1.632391	1.249598
C	2.606428	2.735924	-0.395378
C	3.269737	3.140001	-1.556359
C	2.364165	3.614685	0.659691
C	3.726233	4.457642	-1.645333
C	2.820351	4.932294	0.555184
C	3.503070	5.351612	-0.591602
H	3.430767	2.446473	-2.377314
H	1.826194	3.270476	1.537237
H	4.246621	4.785937	-2.540168

H	2.640620	5.628856	1.368901
H	3.853964	6.376568	-0.668446
S	1.995562	1.022726	-0.283457
C	-0.777895	-1.387345	0.970359
C	-1.091129	-0.149371	0.756020
C	-2.280176	1.876771	1.511898
C	-1.663193	1.579890	-0.801215
C	-2.986860	2.869626	0.585069
H	-1.466808	2.332477	2.079622
H	-2.971606	1.337263	2.157327
H	-4.031511	2.608478	0.418222
H	-2.877716	3.905415	0.901330
N	-1.684646	0.946783	0.520682
O	-2.299653	2.734513	-0.701014
O	-1.110227	1.128166	-1.763584
H	-1.650063	-2.032121	0.806529
S	-4.650337	-0.574281	0.146234
O	-4.921824	-0.013167	1.492570
O	-4.275358	0.431860	-0.878803
O	-3.832385	-1.812079	0.135669
C	-6.339440	-1.169721	-0.431923
F	-7.217712	-0.147786	-0.493301
F	-6.839021	-2.095052	0.412521
F	-6.261072	-1.724445	-1.658128
C	0.537667	-2.012453	1.340424
H	1.203231	-1.250287	1.746552
H	0.337278	-2.757334	2.118838
C	1.189682	-2.703331	0.126687
H	0.494831	-3.440696	-0.294988
H	1.367764	-1.957336	-0.658269
C	2.510558	-3.383122	0.503170
H	3.187512	-2.637606	0.939829
H	2.322340	-4.134545	1.283129
C	3.200145	-4.049939	-0.692290
H	2.521443	-4.788779	-1.142335
H	3.389315	-3.291968	-1.465711
C	4.522293	-4.733669	-0.323680
H	5.185627	-3.996991	0.148767
H	4.330419	-5.507860	0.432247
C	5.229634	-5.359207	-1.530143
H	5.464112	-4.599105	-2.286220
H	6.169915	-5.842114	-1.239258
H	4.598621	-6.118600	-2.008613

Alk_C

O	-0.556982	1.665054	1.011112
C	-1.578889	0.766178	-1.275796
C	-2.627515	1.671997	-1.076524
C	-1.681941	-0.330986	-2.138404
C	-3.809681	1.475264	-1.788536

C	-2.874620	-0.501821	-2.845666	H	1.767546	-0.227107	3.990649
C	-3.930307	0.397455	-2.673971	H	4.112510	0.171806	3.540429
H	-2.532624	2.492378	-0.374416	H	3.850846	0.045310	1.766847
H	-0.856254	-1.027355	-2.250696	N	1.254644	1.013947	2.351639
H	-4.638889	2.160384	-1.643954	O	3.301282	1.850554	2.633669
H	-2.976990	-1.346307	-3.519766	O	1.640590	3.310174	2.148400
H	-4.857411	0.251016	-3.219150	H	-0.651363	-0.010757	3.898049
C	0.895834	2.216156	-1.211254	S	2.005678	-1.988121	-0.616923
C	2.284790	2.020094	-1.215414	O	1.075683	-2.368038	0.472704
C	0.296837	3.354399	-1.766400	O	1.358167	-1.654141	-1.910854
C	3.090979	3.001599	-1.793222	O	3.098729	-1.067209	-0.215101
C	1.122038	4.322761	-2.333107	C	2.917576	-3.592754	-0.989275
C	2.512496	4.146907	-2.347116	F	2.060268	-4.556434	-1.384327
H	2.719419	1.119113	-0.791707	F	3.566673	-4.040549	0.105847
H	-0.778521	3.486325	-1.762272	F	3.827908	-3.419911	-1.969011
H	4.167511	2.865644	-1.810264	C	-2.465258	0.148884	2.622766
H	0.679606	5.213436	-2.767937	H	-3.088295	0.464286	3.470455
H	3.144746	4.906929	-2.796371	H	-2.759639	0.751269	1.759957
S	-0.020475	0.920535	-0.416795	C	-2.724950	-1.348053	2.342311
C	-1.025382	0.392073	2.959742	H	-2.149096	-1.646405	1.456160
C	-0.133114	1.004336	2.176010	H	-2.333929	-1.938490	3.181152
C	2.010025	-0.106856	2.929056	C	-4.207541	-1.690081	2.132414
C	2.017281	2.173391	2.355824	H	-4.301998	-2.781889	2.054006
C	3.438835	0.396678	2.714278	H	-4.778375	-1.393982	3.024914
H	1.800008	-1.028521	2.384642	C	-4.838246	-1.050417	0.888357

H	-4.820037	0.043772	0.977073
H	-4.227831	-1.295447	0.007505
C	-6.283865	-1.503900	0.647404
H	-6.307667	-2.598652	0.552845
H	-6.890826	-1.257883	1.530252
C	-6.913489	-0.872020	-0.598165
H	-6.347382	-1.133029	-1.500294
H	-7.946114	-1.211253	-0.741602
H	-6.929447	0.222342	-0.522365

Alk_TS_C-D

O	-1.226886	-0.258159	-0.598316
C	-0.499196	0.481797	2.030670
C	-1.481002	1.012994	2.887809
C	0.280295	-0.633683	2.401711
C	-1.689315	0.401001	4.116666
C	0.040200	-1.239768	3.635926
C	-0.944645	-0.732638	4.484239
H	-2.055227	1.887368	2.607285
H	1.074015	-0.991919	1.753783
H	-2.434345	0.804909	4.794933
H	0.629371	-2.103430	3.926353
H	-1.126071	-1.206122	5.444471
C	-1.244787	2.413762	0.047813
C	-0.600976	3.588812	-0.368209
C	-2.642765	2.312373	0.102838
C	-1.383018	4.695833	-0.701667
C	-3.404477	3.425307	-0.245428
C	-2.777507	4.614215	-0.640398
H	0.481899	3.640008	-0.426386
H	-3.119590	1.385369	0.395215
H	-0.900046	5.615106	-1.017006
H	-4.487560	3.361368	-0.214878
H	-3.380161	5.476434	-0.909705
S	-0.181019	1.044244	0.401224
C	-1.666793	-2.338845	0.392104
C	-0.887168	-1.530984	-0.383163
C	0.750861	-1.365615	-2.286273
C	1.108438	-2.971802	-0.590693
C	2.092850	-2.090663	-2.479409
H	0.896284	-0.288648	-2.204737
H	0.028231	-1.584655	-3.078712
H	2.191577	-2.558755	-3.459282
H	2.940427	-1.435802	-2.280556
N	0.291684	-1.941724	-1.016598
O	2.106656	-3.147971	-1.480176
O	0.979027	-3.660533	0.405835
H	-1.440705	-3.400617	0.379899
C	-2.847533	-1.900985	1.186488
H	-2.661638	-2.161765	2.239053

H	-2.961252	-0.816656	1.142345
C	-4.152662	-2.604902	0.740883
H	-4.056387	-3.682191	0.927351
H	-4.962007	-2.243118	1.388345
C	-4.529500	-2.377294	-0.731421
H	-3.765056	-2.832180	-1.377129
H	-5.464942	-2.915730	-0.936561
C	-4.697141	-0.903783	-1.123335
H	-5.411582	-0.422830	-0.437849
H	-3.741699	-0.380857	-0.997770
C	-5.172332	-0.709535	-2.568427
H	-4.473755	-1.218547	-3.247619
H	-6.146286	-1.200899	-2.701915
C	-5.281156	0.766617	-2.964850
H	-4.308078	1.267440	-2.888940
H	-5.636727	0.882013	-3.995426
H	-5.980402	1.301059	-2.309281
S	3.286490	0.753549	-0.177550
O	2.806344	-0.582464	0.256213
O	2.858871	1.146765	-1.542977
O	3.199116	1.819028	0.848230
C	5.140224	0.488067	-0.373779
F	5.706418	0.114356	0.792198
F	5.752547	1.618170	-0.786039
F	5.395702	-0.475190	-1.284929

Alk_D

O	0.766497	2.693301	2.171567
C	-1.819231	0.429823	1.123254
C	-2.896884	0.411680	2.013265
C	-0.446452	0.149456	1.631390
C	-2.672910	0.284363	3.379635
C	-0.296926	0.150748	3.097219
C	-1.376327	0.166404	3.934312
H	-0.386018	-0.976099	1.455238
H	-3.532137	0.292732	4.044892
H	0.703192	0.028667	3.495268
H	-1.249617	0.096270	5.008917
C	-3.771053	0.618975	-0.853918
C	-4.399831	-0.630643	-0.939281
C	-4.487770	1.806604	-1.042132
C	-5.770620	-0.681490	-1.202227
C	-5.857973	1.740106	-1.311986
C	-6.498189	0.499322	-1.389582
H	-3.825735	-1.540188	-0.794880
H	-3.986767	2.767613	-0.976007
H	-6.266751	-1.645420	-1.266390
H	-6.420717	2.657103	-1.459872
H	-7.562708	0.451725	-1.600122
S	-1.996682	0.694957	-0.585532

C	0.741895	0.717300	0.808862
C	0.808557	2.225798	1.037533
C	1.058009	4.541680	0.202667
C	1.077636	2.804404	-1.404360
C	0.886115	5.096682	-1.212044
H	2.042735	4.757254	0.626905
H	0.286562	4.888189	0.890015
H	-0.137874	5.421634	-1.413182
H	1.589793	5.890952	-1.458466
N	0.928332	3.095245	-0.037212
O	1.167220	3.963910	-2.084025
O	1.134165	1.721597	-1.942786
H	0.569323	0.498699	-0.242224
S	-0.667877	-3.464264	0.212475
O	-0.603992	-4.937734	0.293109
O	-1.961861	-2.880727	-0.204351
O	-0.017014	-2.749520	1.354529
C	0.483693	-3.047682	-1.217540
F	0.545758	-1.714670	-1.413768
F	0.058714	-3.617800	-2.362241
F	1.734758	-3.487714	-0.972006
C	2.094505	0.066115	1.187604
H	2.379961	0.362254	2.202837
H	1.941233	-1.018620	1.194285
C	3.233823	0.411176	0.221074
H	2.937951	0.143554	-0.799622
H	3.409030	1.496284	0.219056
C	4.536176	-0.310195	0.585808
H	4.824697	-0.047826	1.614134
H	4.358500	-1.395188	0.580848
C	5.693432	0.016937	-0.365067
H	5.397950	-0.234900	-1.393992
H	5.878139	1.101438	-0.353829
C	6.993602	-0.719718	-0.019913
H	7.287382	-0.470580	1.009625
H	6.808192	-1.802844	-0.034019
C	8.143996	-0.385972	-0.975642
H	8.373741	0.686982	-0.958318
H	9.059040	-0.927392	-0.708147
H	7.887961	-0.653195	-2.008579
H	-3.909261	0.536884	1.649221

Alk_TS_D-E

O	-0.354833	1.543659	1.280965
C	-1.768450	1.432143	-1.484056
C	-0.766286	0.490626	-1.837213
C	-1.433618	2.785109	-1.342563
C	0.545890	0.939959	-2.112146
C	-0.123283	3.193811	-1.575423
C	0.872067	2.277328	-1.960101

H	-2.185386	3.511858	-1.057358	N	1.402596	0.218005	1.866189
H	1.296791	0.222599	-2.420151	O	2.962961	-1.057191	2.807287
H	0.127645	4.245006	-1.465459	O	1.289541	-2.086833	1.676868
H	1.888307	2.608939	-2.141672	H	0.484896	-1.124407	-0.335158
C	-4.248922	1.962067	-0.230804	S	3.846969	0.325129	-0.895515
C	-3.709396	2.332252	1.010448	O	3.859072	0.740482	-2.316895
C	-5.505896	2.429420	-0.633777	O	3.882697	1.430439	0.092328
C	-4.434376	3.187439	1.841128	O	2.900837	-0.771472	-0.568448
C	-6.228136	3.275316	0.214433	C	5.526107	-0.495177	-0.665992
C	-5.694228	3.656574	1.448209	F	5.656046	-1.576246	-1.461917
H	-2.730237	1.971962	1.309142	F	5.694378	-0.899338	0.611933
H	-5.916154	2.142068	-1.597248	F	6.528363	0.358888	-0.962577
H	-4.016580	3.482103	2.799536	C	-1.525212	-1.284251	0.611083
H	-7.203072	3.638892	-0.096647	C	-1.758101	-2.644304	-0.047922
H	-6.255436	4.317322	2.102511	H	-1.638421	-2.542360	-1.134165
S	-3.404623	0.785727	-1.291267	H	-0.980137	-3.337948	0.292282
C	-0.239160	-0.665171	0.326917	C	-3.144703	-3.217241	0.264148
C	0.240953	0.481199	1.184704	H	-3.914969	-2.505315	-0.064166
C	2.152235	1.183506	2.671115	H	-3.259084	-3.321242	1.352462
C	1.859273	-1.079508	2.064790	C	-3.387804	-4.574184	-0.405838
C	3.376852	0.332037	3.049749	H	-3.257907	-4.468525	-1.492821
H	1.562005	1.490324	3.539164	H	-2.620247	-5.286923	-0.070942
H	2.424367	2.048174	2.066880	C	-4.777815	-5.155137	-0.117832
H	4.229984	0.533865	2.403162	H	-5.543284	-4.440860	-0.452504
H	3.651415	0.405965	4.100925	H	-4.907265	-5.260327	0.968487

C	-5.013347	-6.509453	-0.794451
H	-6.012630	-6.900658	-0.570736
H	-4.923831	-6.426807	-1.884876
H	-4.280430	-7.252824	-0.456686
H	-1.665959	-1.308371	1.703616
H	-2.322977	-0.566189	0.296056
H	-1.046263	-0.524627	-2.103870

Alk_E

O	1.433014	0.014944	3.253662
C	-3.219182	0.978401	0.022517
C	-4.596158	1.174901	0.201905
C	-2.759577	0.052444	-0.924108
C	-5.507392	0.445699	-0.566764
C	-3.676373	-0.672056	-1.690742
C	-5.049282	-0.478828	-1.511872
H	-4.950374	1.888771	0.939125
H	-1.696409	-0.108124	-1.058140
H	-6.573300	0.597314	-0.422829
H	-3.315095	-1.392129	-2.418862
H	-5.760114	-1.049831	-2.102181
C	-1.673699	3.361056	0.179356
C	-1.799766	3.482589	-1.210907
C	-1.164445	4.431099	0.932361
C	-1.412595	4.669588	-1.840003
C	-0.767523	5.607666	0.291530
C	-0.889626	5.732887	-1.096768
H	-2.190987	2.661822	-1.802383
H	-1.079999	4.349099	2.012721
H	-1.515767	4.756044	-2.918125
H	-0.372875	6.429303	0.882710
H	-0.586744	6.650260	-1.592688
S	-2.056343	1.852048	1.080650
C	0.709875	-1.176799	1.207858
C	1.258042	-0.020341	2.062084
C	1.947403	2.403543	1.118360
C	1.077231	0.631224	-0.066932
C	1.780824	2.726841	-0.395485
H	2.989348	2.441128	1.435822
H	1.324033	3.034504	1.752508
H	1.100945	3.550941	-0.596208
H	2.729069	2.837239	-0.915468
N	1.462157	1.019390	1.146180
O	1.132977	1.508594	-0.999354
O	0.635241	-0.572965	-0.177380
H	1.460863	-1.965011	1.132856
S	4.463697	-0.646865	0.021484
O	5.928495	-0.495896	0.139851
O	3.762178	-1.136350	1.235188
O	3.759047	0.462206	-0.674439

C	4.260507	-2.078816	-1.184072
F	4.829972	-1.799035	-2.373358
F	2.952596	-2.338310	-1.401977
F	4.829250	-3.203564	-0.704783
C	-0.658942	-1.682661	1.615537
H	-0.550390	-2.064848	2.637735
H	-1.354334	-0.839508	1.658358
C	-1.200429	-2.772130	0.683323
H	-1.170303	-2.412429	-0.351845
H	-0.548512	-3.654114	0.732024
C	-2.641883	-3.157522	1.033470
H	-2.677904	-3.553680	2.058127
H	-3.263609	-2.251493	1.025302
C	-3.238744	-4.182194	0.062905
H	-3.179206	-3.782576	-0.959893
H	-2.628584	-5.097029	0.073338
C	-4.696484	-4.539959	0.376144
H	-4.758297	-4.948974	1.394280
H	-5.299432	-3.621170	0.375259
C	-5.292598	-5.543355	-0.616524
H	-5.274815	-5.144418	-1.638663
H	-4.725375	-6.482637	-0.618224
H	-6.333688	-5.782865	-0.370253

Alk_TS_D-F

O	2.490863	-2.149423	2.065828
C	1.417409	0.759617	0.981578
C	1.497320	1.840307	1.865257
C	0.512300	-0.346330	1.278512
C	0.811023	1.808177	3.075284
C	-0.078413	-0.366129	2.594630
C	0.027326	0.703461	3.453326
H	-0.454074	0.215858	0.637559
H	0.893829	2.660071	3.743987
H	-0.686494	-1.219443	2.870757
H	-0.482197	0.695765	4.410528
C	2.783425	2.430655	-0.792013
C	4.122014	2.791668	-0.597790
C	1.837834	3.372755	-1.221980
C	4.516644	4.112435	-0.834436
C	2.242379	4.690303	-1.445707
C	3.578937	5.060303	-1.255146
H	4.844310	2.054143	-0.261322
H	0.800013	3.087770	-1.366979
H	5.554002	4.397497	-0.685166
H	1.512212	5.424485	-1.773368
H	3.888001	6.085877	-1.435661
S	2.284919	0.721574	-0.551891
C	0.702911	-1.672150	0.516192
C	2.046890	-2.273267	0.930324

C	4.053595	-3.629028	0.387932
C	2.437555	-3.348660	-1.313116
C	4.620629	-4.011502	-0.980429
H	3.861589	-4.496867	1.025498
H	4.682774	-2.918879	0.924081
H	5.313335	-3.261353	-1.370608
H	5.079797	-4.999004	-1.004692
N	2.779238	-3.004347	0.001256
O	3.458438	-4.037910	-1.859973
O	1.407710	-3.113110	-1.907667
H	0.707954	-1.464728	-0.554952
S	-2.037291	2.064282	0.016959
O	-2.009965	2.415557	1.445979
O	-1.339591	0.749579	-0.296553
O	-1.731003	3.122697	-0.958648
C	-3.826084	1.596675	-0.326210
F	-4.213276	0.591349	0.477207
F	-4.627274	2.653626	-0.099416
F	-3.985334	1.210028	-1.602582
C	-0.389920	-2.748163	0.746455
H	-0.098598	-3.624832	0.157719
H	-0.391930	-3.063227	1.796539
C	-1.790351	-2.304803	0.312321
H	-2.150685	-1.495615	0.957512
H	-1.727533	-1.883495	-0.696195
C	-2.808040	-3.452598	0.342013
H	-2.512258	-4.217552	-0.390362
H	-2.774041	-3.937437	1.327908
C	-4.249686	-2.998361	0.059414
H	-4.925471	-3.857221	0.176703
H	-4.549034	-2.263710	0.821489
C	-4.462300	-2.386936	-1.332556
H	-4.157607	-3.116986	-2.096107
H	-3.808368	-1.516828	-1.458689
C	-5.912805	-1.958783	-1.577320
H	-6.230197	-1.209719	-0.841162
H	-6.037963	-1.518664	-2.573675
H	-6.598953	-2.811670	-1.499472
H	2.108679	2.700476	1.620412

Alk_F

O	2.108888	-2.616147	1.885369
C	3.419408	-0.319481	-1.026881
C	4.684322	-0.391774	-1.630773
C	3.285040	-0.471329	0.372297
C	5.826118	-0.624699	-0.863041
C	4.444107	-0.732072	1.117192
C	5.701828	-0.806681	0.516118
H	4.768446	-0.276689	-2.706978
H	6.799069	-0.677290	-1.342902

H	4.362022	-0.871064	2.189540
H	6.579388	-1.006250	1.124392
C	0.983984	-1.420095	-1.914070
C	1.515104	-2.699033	-1.692286
C	-0.404105	-1.247321	-2.013332
C	0.656599	-3.793608	-1.561743
C	-1.253610	-2.350285	-1.896390
C	-0.728131	-3.626161	-1.668060
H	2.587879	-2.839719	-1.608037
H	-0.827987	-0.258499	-2.152419
H	1.076252	-4.778906	-1.378802
H	-2.326289	-2.196525	-1.958315
H	-1.390840	-4.480647	-1.567279
S	2.023053	0.034651	-2.109520
C	1.934994	-0.341423	1.058773
C	1.403427	-1.700382	1.494065
C	-0.527075	-3.217425	1.930634
C	-1.011968	-1.040079	1.223578
C	-2.023941	-3.056249	1.634336
H	-0.315894	-3.361430	2.992657
H	-0.069889	-4.022250	1.355579
H	-2.343249	-3.596524	0.742448
H	-2.665950	-3.300092	2.479841
N	0.009532	-1.917351	1.489010
O	-2.194546	-1.629050	1.356488
O	-0.908498	0.153499	0.926417
H	1.232656	0.082697	0.347172
S	-3.733375	1.379287	-0.776873
O	-4.155584	2.615642	-1.424412
O	-3.260628	0.238338	-1.557906
O	-2.760836	1.719739	0.407625
C	-5.232754	0.771727	0.186395
F	-6.209902	0.464832	-0.677978
F	-5.665879	1.727248	1.017969
F	-4.917291	-0.317449	0.896301
H	-2.030531	0.982853	0.603260
C	1.949858	0.598610	2.300269
H	0.941537	0.610678	2.729306
H	2.616706	0.181431	3.062435
C	2.376609	2.034104	1.959997
H	2.471259	2.593095	2.900869
H	3.376763	2.011649	1.512463
C	1.400420	2.779907	1.037786
H	1.256046	2.217026	0.106795
H	0.417879	2.818806	1.526464
C	1.851144	4.204971	0.679908
H	1.017129	4.728053	0.191383
H	2.067912	4.764910	1.602001
C	3.072518	4.264041	-0.249591
H	3.929097	3.768195	0.224132

H	2.851976	3.691957	-1.161789
C	3.464651	5.697207	-0.623977
H	3.720778	6.282180	0.268626
H	4.332287	5.715171	-1.294135
H	2.639980	6.212940	-1.131994

Ph_A

O	0.337226	0.393118	-1.844312
C	2.361181	1.089364	-0.273955
C	2.917404	1.438604	0.960630
C	3.141955	0.780366	-1.387753
C	4.307913	1.495800	1.066593
C	4.532242	0.836950	-1.257393
C	5.112983	1.195925	-0.037923
H	2.283820	1.647469	1.816485
H	2.682154	0.489453	-2.324464
H	4.759098	1.762599	2.017270
H	5.156028	0.591774	-2.111340
H	6.194224	1.232480	0.055726
C	0.060430	2.703490	-0.524630
C	0.843650	3.650888	-1.194064
C	-1.192120	3.018122	0.016472
C	0.349771	4.948392	-1.323685
C	-1.668740	4.323098	-0.122050
C	-0.901121	5.282669	-0.788950
H	1.814093	3.386090	-1.599845
H	-1.779817	2.262980	0.527245
H	0.941940	5.698275	-1.838996
H	-2.636241	4.586429	0.293535
H	-1.275495	6.296986	-0.890670
S	0.566092	0.997643	-0.350115
C	1.263762	-1.896864	1.340292
C	2.247889	-2.404770	0.435476
C	3.612317	-2.429919	0.786200
C	1.854776	-2.855757	-0.842635
C	4.561696	-2.893804	-0.124576
H	3.917482	-2.075554	1.765788
C	2.810603	-3.327653	-1.742167
H	0.805203	-2.819197	-1.117823
C	4.164811	-3.346135	-1.388186
H	5.612323	-2.900911	0.151390
H	2.498717	-3.673286	-2.723771
H	4.906724	-3.708988	-2.093996
C	0.364811	-1.420224	2.003892
C	-1.827339	-1.617273	3.176698
C	-0.736459	0.474543	2.982454
C	-2.787607	-0.455866	3.463767
H	-1.566071	-2.187360	4.073177
H	-2.203446	-2.282566	2.399189
H	-3.486303	-0.288680	2.642027

H	-3.318991	-0.554168	4.409768
N	-0.646200	-0.876519	2.697735
O	-1.930912	0.724153	3.555867
O	0.108525	1.329057	2.783523
S	-2.444880	-1.384593	-0.605578
O	-2.326927	-0.166562	0.223706
O	-2.812033	-2.631047	0.087616
O	-1.325195	-1.544581	-1.599622
H	-0.324712	-0.382650	-1.762279
C	-3.913463	-1.021348	-1.720446
F	-4.133597	-2.034367	-2.576973
F	-3.694689	0.098555	-2.434741
F	-5.026186	-0.841860	-0.983851

Ph_TS_A-B

O	2.361238	-0.564466	-1.769978
C	4.383957	-0.969531	-0.047839
C	5.574589	-0.588971	0.578283
C	3.818028	-2.226505	0.155946
C	6.197193	-1.488368	1.446446
C	4.451601	-3.119376	1.027363
C	5.636144	-2.751989	1.672109
H	6.006192	0.393256	0.403913
H	2.899500	-2.496760	-0.353049
H	7.119436	-1.203865	1.944539
H	4.014929	-4.098803	1.199944
H	6.124954	-3.448343	2.347273
C	2.964722	1.384791	0.017641
C	3.445838	2.694041	-0.051998
C	2.025862	0.999583	0.979873
C	2.988483	3.634733	0.878737
C	1.570412	1.947984	1.895172
C	2.054480	3.261901	1.848284
H	4.164695	2.980210	-0.815052
H	1.655194	-0.019295	1.014607
H	3.354442	4.656254	0.835579
H	0.835294	1.662052	2.641543
H	1.692331	3.996541	2.561421
S	3.574412	0.185137	-1.208302
C	-1.612528	1.098623	-0.793161
C	-1.533657	2.542777	-0.645426
C	-0.324977	3.221613	-0.899794
C	-2.670114	3.277005	-0.260470
C	-0.262782	4.607910	-0.775704
H	0.556133	2.653993	-1.181248
C	-2.598823	4.665409	-0.132933
H	-3.604091	2.760699	-0.061326
C	-1.397921	5.333512	-0.393109
H	0.673615	5.122226	-0.969943
H	-3.481016	5.224119	0.165634

H	-1.345048	6.414249	-0.296519
C	-0.933832	0.074709	-1.037790
C	-0.354382	-1.667774	-2.684917
C	0.067427	-1.986703	-0.350958
C	0.506042	-2.913102	-2.425488
H	0.125082	-0.964705	-3.364517
H	-1.363359	-1.911297	-3.023841
H	0.085782	-3.821988	-2.854014
H	1.540914	-2.772799	-2.737050
N	-0.433310	-1.085430	-1.329116
O	0.511660	-3.080093	-0.973709
O	0.074088	-1.808999	0.842130
H	-2.951267	0.515713	-0.649253
S	-4.285612	-1.305468	-0.255810
O	-4.067798	0.206977	-0.542673
O	-5.648504	-1.701723	-0.605609
O	-3.146087	-2.113941	-0.699172
C	-4.208623	-1.322052	1.624291
F	-4.341610	-2.580357	2.066725
F	-5.193611	-0.572479	2.138893
F	-3.030441	-0.833998	2.035015

Ph_B

O	1.668252	-0.231513	-1.396492
C	3.537073	0.607927	0.346753
C	2.491541	1.073834	1.144017
C	4.869301	0.929985	0.619757
C	2.791848	1.864988	2.256087
C	5.157240	1.721019	1.735335
C	4.120427	2.186750	2.552596
H	1.466225	0.822270	0.899692
H	5.671238	0.568855	-0.019177
H	1.985754	2.231401	2.885001
H	6.188137	1.977517	1.960849
H	4.348538	2.804512	3.416341
C	3.381231	-2.059562	-0.379568
C	2.650747	-2.443729	0.750285
C	4.256072	-2.942647	-1.017821
C	2.807317	-3.736935	1.247736
C	4.411479	-4.235640	-0.504684
C	3.688026	-4.630701	0.623622
H	1.970668	-1.750516	1.233194
H	4.810393	-2.632163	-1.899369
H	2.242967	-4.046108	2.122539
H	5.091921	-4.929302	-0.989547
H	3.806877	-5.635479	1.018606
S	3.169770	-0.411161	-1.116950
C	-1.555809	2.204441	-0.626633
C	-0.563332	3.285992	-0.698518
C	-0.970820	4.585861	-0.352383

C	0.767416	3.050280	-1.092945
C	-0.054994	5.638775	-0.396834
H	-1.998668	4.764598	-0.049615
C	1.674094	4.106298	-1.133142
H	1.089461	2.045498	-1.352508
C	1.267173	5.401332	-0.785839
H	-0.373704	6.641177	-0.126920
H	2.701966	3.918457	-1.429440
H	1.979737	6.220576	-0.817796
C	-1.338578	0.939300	-0.849976
C	-1.094545	-1.023900	-2.301946
C	-0.603874	-1.148591	0.074632
C	-0.701648	-2.439954	-1.848229
H	-0.368752	-0.584026	-2.984043
H	-2.105338	-0.971779	-2.702567
H	-1.517757	-3.152982	-1.952872
H	0.204110	-2.800401	-2.334097
N	-1.050167	-0.268340	-1.028177
O	-0.405008	-2.345977	-0.423207
O	-0.452124	-0.780512	1.204779
H	-2.588314	2.445168	-0.371691
S	-4.405857	-0.867439	-0.382229
O	-3.995165	0.456525	-0.923443
O	-5.727815	-1.352823	-0.828256
O	-3.318620	-1.879122	-0.373122
C	-4.660702	-0.505541	1.447214
F	-4.989078	-1.626412	2.120096
F	-5.650057	0.393728	1.629510
F	-3.537882	-0.001918	1.999101

Ph_TS_B-C

O	-1.505833	-0.329214	-1.072369
C	-2.296620	-2.314403	0.564836
C	-2.885672	-3.100621	-0.431211
C	-1.795235	-2.874314	1.741787
C	-2.980853	-4.477848	-0.232749
C	-1.901803	-4.256904	1.931935
C	-2.492193	-5.054288	0.948210
H	-3.263100	-2.644101	-1.341017
H	-1.326957	-2.249162	2.496982
H	-3.438146	-5.101535	-0.995237
H	-1.516673	-4.705436	2.842705
H	-2.570869	-6.127174	1.097659
C	-3.840791	-0.027430	0.209944
C	-4.543921	0.245785	1.385184
C	-4.421158	0.121822	-1.049242
C	-5.874747	0.661246	1.290395
C	-5.752405	0.538831	-1.130572
C	-6.477307	0.807807	0.035826
H	-4.067738	0.143132	2.356771

H	-3.834385	-0.070269	-1.941710
H	-6.434720	0.879923	2.194792
H	-6.219120	0.659818	-2.103826
H	-7.509836	1.137692	-0.032483
S	-2.099826	-0.526989	0.340003
C	0.441489	1.824846	-0.078047
C	-0.743101	2.646183	0.175816
C	-0.782841	3.398186	1.364224
C	-1.819414	2.716383	-0.727703
C	-1.897554	4.183198	1.658972
H	0.054420	3.354766	2.055058
C	-2.925616	3.509828	-0.430171
H	-1.788248	2.140991	-1.645197
C	-2.971821	4.238265	0.764234
H	-1.925736	4.752759	2.583104
H	-3.756027	3.554166	-1.128103
H	-3.839275	4.850503	0.993380
C	0.636353	0.797136	-0.858517
C	1.267245	-1.497376	-1.516228
C	1.915352	0.405847	-2.855703
C	2.391756	-1.830104	-2.499001
H	0.294142	-1.882171	-1.821821
H	1.502179	-1.776583	-0.490497
H	3.361203	-1.917268	-2.009657
H	2.176989	-2.696903	-3.121482
N	1.219892	-0.019761	-1.631087
O	2.467803	-0.668174	-3.390805
O	1.922758	1.526190	-3.280047
H	1.355237	2.030185	0.496767
S	3.857587	0.185598	0.561232
O	3.145498	-1.021480	1.048491
O	4.082289	0.222353	-0.905728
O	3.398958	1.465664	1.156891
C	5.591667	-0.022691	1.261162
F	6.159256	-1.157597	0.803501
F	5.560176	-0.087907	2.607898
F	6.385745	1.008643	0.913039

Ph_C

O	-1.620099	1.020874	-0.355699
C	-0.895638	-1.075646	-1.801748
C	-1.235305	-0.763084	-3.123352
C	-1.129130	-2.340945	-1.245808
C	-1.842317	-1.749141	-3.899639
C	-1.736333	-3.312913	-2.039950
C	-2.093838	-3.016514	-3.359602
H	-1.031425	0.217227	-3.539044
H	-0.836771	-2.562365	-0.224790
H	-2.115012	-1.528000	-4.926673
H	-1.923539	-4.299439	-1.628425

H	-2.564084	-3.778419	-3.974074
C	0.710700	1.288526	-1.688387
C	2.093044	1.087580	-1.776459
C	0.061572	2.354180	-2.318823
C	2.840060	1.983406	-2.542723
C	0.831968	3.239402	-3.074514
C	2.213505	3.053995	-3.189074
H	2.566172	0.256822	-1.267681
H	-1.003810	2.507348	-2.201905
H	3.913683	1.845134	-2.623385
H	0.348235	4.079052	-3.563559
H	2.804292	3.751160	-3.775695
S	-0.205525	0.119797	-0.681855
C	-2.751239	1.000025	1.753561
C	-1.722206	1.398521	0.987811
C	0.157373	1.868068	2.643620
C	-0.357516	3.428554	0.960328
C	1.316049	2.841496	2.422148
H	0.491930	0.831096	2.652510
H	-0.416204	2.096581	3.548361
H	1.689268	3.301111	3.336333
H	2.129533	2.377002	1.859962
N	-0.636486	2.156472	1.438035
O	0.741345	3.900006	1.590592
O	-0.978797	4.051564	0.122198
H	-2.764368	1.445321	2.745701
S	2.448100	-1.551998	1.113981
O	1.988513	-1.807873	-0.279650
O	2.452655	-0.118217	1.494654
O	1.921459	-2.486006	2.132996
C	4.279778	-1.976593	1.029290
F	4.914901	-1.189659	0.134809
F	4.465755	-3.259266	0.657418
F	4.872185	-1.801686	2.228031
C	-3.822431	0.038960	1.475537
C	-4.088582	-0.528274	0.210488
C	-4.633714	-0.349151	2.563284
C	-5.111130	-1.463685	0.054819
H	-3.503433	-0.237764	-0.651134
C	-5.656779	-1.283800	2.402707
H	-4.451113	0.084353	3.543149
C	-5.898049	-1.849100	1.146447
H	-5.296391	-1.890252	-0.927045
H	-6.264159	-1.569385	3.256848
H	-6.693825	-2.577147	1.017466

Ph_TS_C-D

O	-1.195732	0.120199	-1.568962
C	-0.975492	0.322326	1.238740
C	-2.079388	0.875407	1.913696

C	-0.423431	-0.918540	1.618234	C	2.284198	-1.871711	-2.939028
C	-2.639120	0.162725	2.964634	H	1.231944	0.024528	-2.638323
C	-1.012484	-1.621421	2.670233	H	0.479670	-1.008672	-3.878736
C	-2.118819	-1.089539	3.332679	H	2.521986	-2.258562	-3.930672
H	-2.483971	1.838116	1.628615	H	3.142144	-1.352802	-2.511736
H	0.456558	-1.307447	1.117689	N	0.206683	-1.680055	-1.897747
H	-3.489793	0.576241	3.496691	O	1.986950	-3.009440	-2.083314
H	-0.602198	-2.582014	2.963977	O	0.406576	-3.605554	-0.590549
H	-2.577229	-1.640613	4.148154	H	-1.851461	-3.087211	-1.067328
C	-0.860767	2.639305	-0.462547	S	3.442454	0.394299	-0.007678
C	0.109656	3.654300	-0.469872	O	2.448143	-0.706295	-0.036531
C	-2.213833	2.901453	-0.721747	O	4.431486	0.366724	-1.110751
C	-0.301596	4.966776	-0.708160	O	2.888672	1.733048	0.315986
C	-2.599074	4.217369	-0.968757	C	4.474073	-0.015352	1.513014
C	-1.648629	5.246715	-0.956614	F	3.707576	-0.047436	2.624309
H	1.153853	3.419364	-0.285009	F	5.443521	0.903350	1.706505
H	-2.940489	2.099214	-0.738172	F	5.066581	-1.220386	1.385053
H	0.434399	5.764581	-0.707311	C	-3.348132	-1.618303	-0.509017
H	-3.641717	4.438686	-1.174448	C	-4.030387	-0.444444	-0.895065
H	-1.960052	6.268773	-1.150509	C	-3.959842	-2.469462	0.437726
S	-0.216264	1.016178	-0.181784	C	-5.258246	-0.118282	-0.323001
C	-2.054028	-2.021970	-1.045099	H	-3.606476	0.188436	-1.664324
C	-1.054798	-1.212002	-1.506573	C	-5.183075	-2.132571	1.016381
C	1.011682	-1.004921	-2.922653	H	-3.455774	-3.386542	0.729520
C	0.821167	-2.830324	-1.433628	C	-5.834891	-0.952436	0.643552

H	-5.774306	0.784074	-0.638998
H	-5.627947	-2.792424	1.755675
H	-6.791259	-0.692754	1.088289

Ph_D

O	2.696207	0.903412	1.916651
C	-0.172548	0.805300	0.745008
C	-1.031302	1.021493	1.818730
C	0.651321	-0.446021	0.690892
C	-1.063459	0.124637	2.879442
C	0.659706	-1.247259	1.936897
C	-0.210154	-1.005960	2.955325
H	-1.685418	1.884188	1.834592
H	0.010101	-1.073017	0.025762
H	-1.754786	0.316591	3.694239
H	1.329048	-2.100284	1.970738
H	-0.246899	-1.652602	3.824810
C	-1.552726	2.882884	-0.493582
C	-1.416478	4.250254	-0.231380
C	-2.809773	2.305298	-0.721242
C	-2.562618	5.051232	-0.193728
C	-3.944995	3.115247	-0.670506
C	-3.822753	4.485637	-0.410579
H	-0.436756	4.683800	-0.055284
H	-2.892214	1.240532	-0.913956
H	-2.467347	6.113588	0.010291
H	-4.923017	2.673692	-0.836924
H	-4.709855	5.111405	-0.377525
S	-0.084008	1.858904	-0.633754
C	2.018245	-0.326209	-0.045319
C	2.670936	-1.681451	-0.299748
C	2.241413	-2.436724	-1.400339
C	3.660502	-2.205163	0.542571
C	2.788602	-3.695850	-1.652556
H	1.478029	-2.033902	-2.060955
C	4.214027	-3.464091	0.287120
H	4.004703	-1.641332	1.405589
C	3.778767	-4.213063	-0.809220
H	2.446437	-4.269541	-2.509067
H	4.981288	-3.856607	0.948379
H	4.207650	-5.191303	-1.006745
C	2.922698	0.617534	0.746217
C	5.007280	1.954206	0.882066
C	4.391183	1.092362	-1.231534
C	6.178072	2.013111	-0.101333
H	5.270017	1.478470	1.827008
H	4.568553	2.936534	1.079046
H	6.637471	2.997847	-0.176153
H	6.936687	1.253808	0.104163
N	4.054714	1.123382	0.130752

O	5.580739	1.700593	-1.394797
O	3.747936	0.632174	-2.148887
H	1.840093	0.115599	-1.027418
S	-3.109343	-1.832532	0.263697
O	-2.918037	-3.299628	0.318713
O	-3.453959	-1.181687	1.549348
O	-2.113443	-1.099213	-0.564045
C	-4.689386	-1.635017	-0.742684
F	-4.999404	-0.329732	-0.906368
F	-5.733813	-2.231077	-0.130921
F	-4.559904	-2.182817	-1.968702

Ph_TS_D-E

O	0.276857	1.439407	2.148386
C	1.976645	-1.092059	1.624360
C	1.191913	-1.151816	0.426249
C	1.353371	-1.283605	2.865350
C	-0.169063	-1.580027	0.517185
C	0.003457	-1.614531	2.920181
C	-0.759828	-1.783691	1.746236
H	1.928327	-1.206204	3.783036
H	-0.741384	-1.702069	-0.395628
H	-0.462643	-1.772046	3.888409
H	-1.799731	-2.080241	1.808273
C	4.349523	-1.400229	0.117364
C	5.293604	-0.640832	-0.583466
C	4.014775	-2.693598	-0.310694
C	5.893072	-1.173669	-1.728309
C	4.603892	-3.206382	-1.467583
C	5.544280	-2.450296	-2.177605
H	5.546033	0.360580	-0.252001
H	3.297922	-3.288710	0.245974
H	6.622261	-0.581633	-2.273662
H	4.335226	-4.202999	-1.805674
H	6.004817	-2.857900	-3.072709
S	3.692575	-0.714189	1.641461
C	0.827592	0.961622	-0.146473
C	-0.060050	1.445094	0.971165
C	-2.307536	2.268580	1.612071
C	-1.688443	2.363491	-0.669950
C	-3.496204	2.653583	0.723533
H	-1.937344	3.102816	2.214728
H	-2.531504	1.415654	2.253117
H	-4.209388	1.836249	0.621698
H	-3.994431	3.569826	1.038428
N	-1.311145	1.897246	0.600084
O	-2.910732	2.901704	-0.589339
O	-1.015396	2.363121	-1.680075
H	0.308664	0.605055	-1.025532
S	-3.728567	-1.080317	-0.189175

O	-3.349189	-2.512876	-0.224351
O	-4.046241	-0.545958	1.158097
O	-2.899826	-0.189252	-1.035174
C	-5.398783	-1.051002	-1.058077
F	-5.885708	0.207193	-1.126536
F	-6.304709	-1.807276	-0.402625
F	-5.301800	-1.524491	-2.318030
C	2.104621	1.566080	-0.384564
C	2.814142	2.297821	0.603687
C	2.640045	1.482516	-1.698424
C	3.989357	2.963564	0.266324
H	2.422319	2.355620	1.611711
C	3.805944	2.154408	-2.023506
H	2.107355	0.909274	-2.451544
C	4.479762	2.901498	-1.041752
H	4.521925	3.531613	1.022371
H	4.199137	2.104019	-3.033690
H	5.392529	3.430296	-1.300657
H	1.693423	-1.244946	-0.529240

Ph_E

O	-1.209796	2.253777	2.370815
C	3.405308	-1.864359	0.164331
C	3.229191	-0.743475	0.989224
C	3.207711	-3.150693	0.683690
C	2.841419	-0.913284	2.320825
C	2.830680	-3.314895	2.021191
C	2.645386	-2.198184	2.840789
H	3.384194	0.253565	0.590819
H	3.342440	-4.018122	0.044943
H	2.704527	-0.042864	2.955383
H	2.678880	-4.315228	2.416810
H	2.352455	-2.325896	3.879217
C	2.412344	-1.041602	-2.310487
C	1.261081	-1.840979	-2.353098
C	2.397838	0.237508	-2.879964
C	0.094131	-1.349830	-2.940884
C	1.229832	0.716309	-3.484130
C	0.075349	-0.070676	-3.508523
H	1.272918	-2.832432	-1.911538
H	3.285255	0.861396	-2.835188
H	-0.802333	-1.962764	-2.950934
H	1.221486	1.711911	-3.919048
H	-0.837055	0.309923	-3.958269
S	3.929446	-1.659307	-1.551036
C	0.057784	1.717006	0.312744
C	-0.728462	1.451306	1.611129
C	-1.317351	-0.968354	2.604550
C	-0.095536	-0.495925	0.702757
C	-0.835382	-2.254209	1.871844

H	-2.403591	-0.883737	2.619564
H	-0.906083	-0.877055	3.610521
H	-0.160670	-2.869983	2.461705
H	-1.649760	-2.832414	1.443674
N	-0.747242	0.060311	1.728647
O	-0.024038	-1.775043	0.702089
O	0.420515	0.307127	-0.154665
H	-0.636113	2.071716	-0.449791
S	-3.378788	-0.055744	-0.827480
O	-3.820493	-0.247268	-2.224250
O	-2.668531	-1.207342	-0.214127
O	-2.766438	1.261099	-0.518298
C	-4.989364	-0.007773	0.145118
F	-5.670754	-1.163905	0.006537
F	-5.786029	0.996858	-0.269044
F	-4.746970	0.171809	1.462429
C	1.293712	2.549760	0.414622
C	1.705037	3.275806	-0.712380
C	2.065838	2.581421	1.584596
C	2.887501	4.016329	-0.673795
H	1.104077	3.250880	-1.616357
C	3.245868	3.324754	1.622210
H	1.753538	2.030450	2.465776
C	3.659556	4.039107	0.492407
H	3.203283	4.575248	-1.549537
H	3.840558	3.346065	2.530419
H	4.579376	4.615918	0.523166

Ph_TS_D-F

O	2.670817	1.352805	1.776387
C	-0.441555	1.155471	1.000213
C	-1.367890	1.461647	2.001604
C	0.377688	-0.043897	1.128067
C	-1.425131	0.694553	3.161667
C	0.351059	-0.724411	2.399201
C	-0.563492	-0.396730	3.374012
H	-0.462075	-0.811455	0.535027
H	-2.153870	0.956998	3.923162
H	1.016346	-1.569645	2.535267
H	-0.615235	-0.964920	4.296280
C	-1.710234	3.190878	-0.440373
C	-2.955713	2.718523	-0.876465
C	-1.547099	4.517230	-0.022419
C	-4.048990	3.588056	-0.880529
C	-2.648177	5.378746	-0.036075
C	-3.896550	4.915143	-0.463217
H	-3.069744	1.686494	-1.194234
H	-0.577103	4.870238	0.313854
H	-5.017635	3.227267	-1.213812
H	-2.528410	6.408438	0.287839

H	-4.749504	5.587626	-0.472503
S	-0.287233	2.094975	-0.480805
C	1.622787	-0.178207	0.224040
C	2.246778	-1.573012	0.216285
C	3.253454	-1.939076	1.121159
C	1.793083	-2.516094	-0.716644
C	3.795667	-3.227867	1.095834
H	3.616745	-1.223417	1.853733
C	2.329985	-3.804997	-0.738574
H	1.015692	-2.240114	-1.421573
C	3.333784	-4.165011	0.167117
H	4.576093	-3.496568	1.802178
H	1.967513	-4.524794	-1.467026
H	3.754326	-5.166445	0.146591
C	2.657266	0.869431	0.651391
C	4.706750	2.168441	0.150229
C	3.727298	0.949328	-1.622162
C	5.668510	2.046848	-1.033421
H	5.155572	1.861341	1.094910
H	4.289699	3.173912	0.256882
H	6.074823	3.001207	-1.366322
H	6.476181	1.334761	-0.846117
N	3.648050	1.230088	-0.250357
O	4.849338	1.509695	-2.113417
O	2.942452	0.330433	-2.306884
H	1.328226	0.029773	-0.805975
S	-2.735330	-1.500751	-0.315471
O	-1.215928	-1.611304	-0.313683
O	-3.312345	-1.280262	1.020277
O	-3.262407	-0.692130	-1.427089
C	-3.182133	-3.273929	-0.756687
F	-2.637072	-3.625840	-1.932996
F	-4.517845	-3.397605	-0.851636
F	-2.742416	-4.121706	0.188718
H	-2.035282	2.307185	1.887735

Ph_F

O	0.060316	-0.297010	-0.945644
C	0.276197	-0.276329	2.611178
C	-0.678399	-0.686305	3.552440
C	-0.023049	0.765081	1.700851
C	-1.935143	-0.079415	3.601882
C	-1.290217	1.356077	1.766755
C	-2.239149	0.943576	2.704393
H	-0.432003	-1.485197	4.244632
H	-2.666717	-0.409501	4.333417
H	-1.541098	2.154171	1.076934
H	-3.214008	1.421319	2.726363
C	1.876280	-2.217276	1.311171
C	0.711759	-2.895270	0.926126

C	3.088065	-2.477361	0.653121
C	0.760372	-3.819685	-0.119612
C	3.131272	-3.422222	-0.376130
C	1.968611	-4.092786	-0.770025
H	-0.230448	-2.693526	1.423814
H	3.988951	-1.937503	0.932209
H	-0.151062	-4.327745	-0.422401
H	4.073401	-3.619461	-0.879753
H	2.002086	-4.816390	-1.579361
S	1.901360	-1.052192	2.684871
C	1.015780	1.235060	0.684403
C	0.825654	2.675532	0.203378
C	0.045871	2.998446	-0.915639
C	1.438427	3.707544	0.927806
C	-0.121333	4.332645	-1.301393
H	-0.444120	2.216223	-1.487787
C	1.266682	5.039921	0.547453
H	2.051485	3.463304	1.790875
C	0.487009	5.356909	-0.570987
H	-0.728794	4.567000	-2.171106
H	1.747661	5.828784	1.118956
H	0.357569	6.393103	-0.870401
C	1.058047	0.314396	-0.527999
C	2.214624	-0.626228	-2.489007
C	3.481558	0.721558	-0.993088
C	3.713434	-0.708482	-2.798783
H	1.644920	-0.112356	-3.266895
H	1.772977	-1.603923	-2.299017
H	4.138696	-1.681144	-2.546740
H	3.958127	-0.441637	-3.826443
N	2.206239	0.183455	-1.259413
O	4.337338	0.277947	-1.925916
O	3.802136	1.464533	-0.094281
H	1.994352	1.187865	1.161398
S	-3.122518	-1.480982	-0.980906
O	-2.559589	-1.687088	-2.313270
O	-4.107138	-2.399778	-0.422644
O	-2.012317	-1.224491	0.097929
C	-3.949103	0.211207	-1.036151
F	-4.524794	0.492373	0.137493
F	-3.034122	1.151915	-1.312405
F	-4.883182	0.211934	-1.995901
H	-1.153729	-0.775216	-0.291180

Ph_TS_C-E-open

O	0.64104	-0.72024	-1.39211
C	0.74564	-2.70243	0.56895
C	-0.51245	-3.33719	0.55353
C	1.93160	-3.45103	0.63344
C	-0.57339	-4.72761	0.57217

C	1.84974	-4.84205	0.65544
C	0.60546	-5.48126	0.61679
H	-1.42760	-2.75394	0.53011
H	2.89874	-2.96958	0.68753
H	-1.54018	-5.22057	0.55850
H	2.76303	-5.42631	0.70805
H	0.55429	-6.56573	0.63277
C	2.28925	-0.31488	0.91135
C	3.39484	-0.60730	0.09976
C	2.38350	0.54220	2.01775
C	4.62565	-0.04093	0.42265
C	3.62948	1.09107	2.32783
C	4.74476	0.80152	1.53582
H	3.28486	-1.22134	-0.78521
H	1.50548	0.77597	2.61143
H	5.48709	-0.24530	-0.20541
H	3.72086	1.75384	3.18267
H	5.70764	1.24231	1.77665
S	0.66321	-0.93536	0.55532
C	-0.89961	0.83043	-2.31800
C	0.34788	0.48456	-1.86749
C	1.22207	2.87202	-1.64347
C	2.63947	1.17332	-2.40819
C	2.68563	3.28981	-1.51461
H	0.63755	3.05605	-0.74332
H	0.74070	3.34089	-2.50996
H	2.90560	4.26820	-1.94048
H	3.04113	3.23340	-0.48181
N	1.39366	1.42776	-1.86165
O	3.40553	2.28706	-2.29288
O	3.03541	0.14859	-2.92942
H	-0.97344	1.81255	-2.77694
C	-2.13815	0.08741	-2.24289
C	-3.33669	0.79195	-2.51432
C	-2.22917	-1.28659	-1.91731
C	-4.57385	0.16121	-2.42582
H	-3.28517	1.84563	-2.77495
C	-3.46908	-1.91342	-1.83686
H	-1.32596	-1.85781	-1.75709
C	-4.64615	-1.19428	-2.08133
H	-5.48197	0.72329	-2.62291
H	-3.51992	-2.96999	-1.58941
H	-5.61086	-1.68882	-2.01359
S	-1.30065	1.93084	2.06678
O	-1.79840	3.26940	2.45524
O	-0.67970	1.13317	3.15401
O	-0.57288	1.87621	0.77240
C	-2.88326	0.97796	1.69993
F	-3.59302	1.57621	0.72234
F	-2.61347	-0.28407	1.30315

F	-3.66967	0.90396	2.79479	H	-0.15807	2.92063	1.67005
				S	4.40948	0.81100	-0.89692
Ph_E-open				C	-0.78023	0.64479	-0.38044
O	-1.57674	2.34315	-1.86583	C	-1.65351	1.78911	-0.77799
C	5.20452	-0.33649	0.23191	C	-2.87410	1.46552	1.45415
C	5.78947	0.13033	1.41877	C	-3.26885	3.39849	0.16078
C	5.30857	-1.68600	-0.12847	C	-4.19916	2.12069	1.83352
C	6.45880	-0.76533	2.25439	H	-2.09090	1.65745	2.19431
C	5.99413	-2.57207	0.70866	H	-2.98557	0.39680	1.28823
C	6.56371	-2.11572	1.90051	H	-5.05731	1.58165	1.42286
H	5.71724	1.17967	1.68729	H	-4.32156	2.27369	2.90493
H	4.85948	-2.04338	-1.04883	N	-2.56992	2.17748	0.19828
H	6.90576	-0.40521	3.17641	O	-4.14059	3.42659	1.19483
H	6.07492	-3.61834	0.42882	O	-3.13028	4.32073	-0.60779
H	7.09062	-2.80784	2.55080	H	-0.99338	0.16682	0.56520
C	3.01706	1.43550	-0.00712	C	0.22376	0.06344	-1.14575
C	2.37209	2.56027	-0.57490	C	0.69037	0.56040	-2.41153
C	2.51784	0.86743	1.18540	C	0.86595	-1.09729	-0.58752
C	1.24073	3.08834	0.02920	C	1.72818	-0.07392	-3.05999
C	1.38465	1.40773	1.78003	C	1.89707	-1.72328	-1.25475
C	0.71949	2.49697	1.19280	H	0.49895	-1.48845	0.35513
H	2.76487	3.01250	-1.48057	C	2.34570	-1.20072	-2.48021
H	3.00993	0.01243	1.63313	H	2.37254	-2.59943	-0.82840
H	0.74319	3.94385	-0.41578	H	3.16247	-1.68822	-3.00359
H	0.99460	0.96183	2.69002	S	-2.78074	-2.12776	-0.24109

O	-2.36515	-3.19006	-1.18096
O	-3.14027	-0.82402	-0.86099
O	-1.95805	-1.99907	0.99046
C	-4.43323	-2.74281	0.41928
F	-4.29414	-3.93290	1.03810
F	-4.94974	-1.86997	1.31184
F	-5.32846	-2.89466	-0.57661
H	0.21609	1.43152	-2.84096
H	2.08716	0.30481	-4.01115

Ph_TS_E-open-E

O	-1.607690	3.590380	-0.753270
C	4.757710	-0.341360	-0.260670
C	4.934770	-1.329590	-1.241170
C	5.176600	-0.561130	1.057270
C	5.511050	-2.550410	-0.887360
C	5.765580	-1.782980	1.396540
C	5.927960	-2.778560	0.429270
H	4.618560	-1.149830	-2.264000
H	5.039600	0.205550	1.812040
H	5.639100	-3.320010	-1.642830
H	6.087300	-1.955580	2.419310
H	6.378780	-3.729130	0.698650
C	2.471160	0.934490	-1.307060
C	1.836210	1.996990	-2.001190
C	1.787270	-0.285490	-1.107870
C	0.545020	1.834660	-2.475490
C	0.492490	-0.430670	-1.584920
C	-0.147280	0.632780	-2.243230
H	2.365810	2.930210	-2.167850
H	2.261610	-1.100190	-0.575400
H	0.058830	2.650560	-2.999510
H	-0.039700	-1.354330	-1.395090
H	-1.154000	0.505710	-2.625930
S	4.091940	1.264890	-0.706530
C	-0.980080	1.549540	0.354010
C	-1.905700	2.501640	-0.304390
C	-3.700080	0.995050	-1.328260
C	-4.028360	1.988130	0.772520
C	-5.126950	0.738670	-0.811070
H	-3.688570	1.371910	-2.352480
H	-3.073830	0.103640	-1.245240
H	-5.387930	-0.319070	-0.784480
H	-5.876780	1.303340	-1.371220
N	-3.268360	2.031790	-0.378940
O	-5.127710	1.235800	0.555780
O	-3.787880	2.553700	1.824670
H	-1.397260	0.561390	0.522760
C	0.254660	1.841100	0.918470
C	0.914050	3.118280	0.828460

C	0.923130	0.767940	1.612930
C	2.155430	3.292920	1.397360
C	2.159630	0.968280	2.187360
H	0.429840	-0.197890	1.669520
C	2.792380	2.218170	2.056430
H	2.659400	0.157400	2.705220
H	3.770010	2.373190	2.502340
S	-2.142010	-2.240430	1.089620
O	-2.622770	-3.179730	2.124700
O	-2.849260	-0.935930	1.026060
O	-0.668540	-2.142560	0.938090
C	-2.672810	-3.064500	-0.519570
F	-2.085560	-4.267670	-0.673850
F	-2.332570	-2.302190	-1.587090
F	-4.008710	-3.244980	-0.559580
H	0.420320	3.934300	0.317550
H	2.658130	4.251640	1.326000

Ph_TS_E-open-D

O	-3.55090	-2.31364	0.04886
C	0.43444	-1.56428	-1.72602
C	0.79252	-0.41094	-2.44232
C	-0.95153	-1.95026	-1.65606
C	-0.17335	0.28857	-3.15082
C	-1.89338	-1.26267	-2.48803
C	-1.52002	-0.14025	-3.19093
C	3.10788	-1.72037	-0.78035
C	3.18207	-0.41816	-0.26387
C	4.27143	-2.41954	-1.12935
C	4.43058	0.18680	-0.11611
C	5.51675	-1.80646	-0.96112
C	5.59832	-0.50476	-0.45844
H	2.28430	0.12662	0.00306
H	4.21007	-3.42784	-1.52806
H	4.48468	1.19797	0.27578
H	6.41901	-2.34766	-1.23016
H	6.56695	-0.02980	-0.33299
S	1.55480	-2.61316	-0.89224
C	-1.46788	-1.14622	0.31854
C	-0.79260	-1.94867	1.29795
C	0.40758	-1.42703	1.85289
C	-1.25270	-3.22008	1.73805
C	1.09052	-2.11969	2.84878
H	0.76194	-0.45598	1.52993
C	-0.54897	-3.91206	2.71097
H	-2.15106	-3.64300	1.30679
C	0.61743	-3.36231	3.27501
H	1.99180	-1.69429	3.27839
H	-0.90283	-4.88304	3.04357
H	1.15299	-3.91170	4.04392

C	-2.96303	-1.24589	0.13509
C	-2.98866	1.29971	0.34872
C	-4.92501	0.15524	-0.38915
C	-4.23704	2.16613	0.50489
H	-2.36115	1.61596	-0.48753
H	-2.39259	1.28114	1.25851
H	-4.57044	2.23402	1.54379
H	-4.13114	3.16098	0.07470
N	-3.60199	-0.01513	0.06466
O	-5.26205	1.45531	-0.24734
O	-5.66358	-0.67169	-0.86941
H	-1.01972	-0.17680	0.12984
S	0.57635	2.45203	1.06941
O	1.55018	1.96342	2.07286
O	0.29115	1.48530	-0.02508
O	-0.61199	3.15389	1.60543
C	1.52102	3.80770	0.16859
F	1.88154	4.79486	1.01308
F	0.76800	4.35265	-0.81014
F	2.64344	3.31714	-0.39909
H	1.82125	-0.07620	-2.46805
H	0.12271	1.17435	-3.70488
H	-2.24527	0.40356	-3.78725
H	-2.91436	-1.62640	-2.52411
H	-1.20921	-2.94750	-1.31160

Cartesian coordinates for the calculations shown in Fig 10 – the most stable (ΔG_{298}) conformations as computed at the RI-MP2/def2-TZVP level of theory

Tf_2NH

N	-0.0000173	-0.0000165	-0.7780541
H	-0.0000446	-0.0000500	-1.7956745
S	-1.2182527	-0.8810501	-0.0666222
S	1.2182555	0.8810658	-0.0667465
O	1.6634430	1.7952530	-1.0668295
O	0.8325961	1.2383282	1.2536896
O	-1.6634762	-1.7953224	-1.0666112
O	-0.8325323	-1.2382023	1.2538260
C	2.5347921	-0.4064940	0.0720994
C	-2.5347940	0.4065099	0.0721773
F	3.6221386	0.1675394	0.5631571
F	2.1452011	-1.3851002	0.8696087
F	2.7980056	-0.8985588	-1.1339479
F	-2.1452253	1.3851241	0.8696878
F	-3.6221533	-0.1675196	0.5632115
F	-2.7979761	0.8985635	-1.1338815

Tf_2N^-

N	0.0000772	0.0000341	-0.7627683
S	1.1500039	0.8034583	-0.0351840
S	-1.1499905	-0.8034538	-0.0354774
O	-0.9296716	-1.1532128	1.3424097
O	-1.6674474	-1.7952368	-0.9401283
O	1.6676488	1.7953078	-0.9396545
O	0.9294106	1.1531124	1.3426858
C	-2.4995274	0.4542994	0.0294241
C	2.4995195	-0.4543086	0.0298909
F	-3.5897068	-0.0812199	0.5980666
F	-2.1457163	1.5214709	0.7461926
F	-2.8423338	0.8711958	-1.1928060
F	3.5895672	0.0811398	0.5988527
F	2.8426126	-0.8710656	-1.1923064
F	2.1455339	-1.5215610	0.7464525

G(H)

O	-0.1283991	-2.2042960	-0.4153486
C	1.6046687	-0.1820315	-0.3750983
C	2.5031560	-0.7857382	0.5139722
C	1.3643145	1.2078651	-0.3684310
C	3.1986362	0.0093423	1.4084425
C	2.0856918	1.9888870	0.5378178
C	2.9890274	1.3904958	1.4071917
H	1.9345090	3.0584484	0.5811182
C	1.7118186	-2.3218030	-2.1519202
S	0.6093078	-1.1286337	-1.4277982
C	-0.7580351	-1.9136663	1.8448384
C	-0.8848408	-1.5277586	0.5751180
H	-0.0002405	-2.6619471	2.0516874
C	-1.5970982	-1.3981048	2.9619730
H	2.3419619	-1.7734036	-2.8532942
H	2.3060696	-2.8118628	-1.3845794
H	1.0890550	-3.0407966	-2.6847349
H	3.8969438	-0.4390850	2.1037881
N	-1.6434486	-0.4914343	0.0591521
C	-2.7977987	-0.6722181	-0.8157435
H	-2.5465085	-1.2566623	-1.7013384
H	-3.6127251	-1.1628881	-0.2767342
C	-3.0961855	0.7870249	-1.1445662
H	-4.1566216	1.0095847	-1.2275846
C	-1.6227667	0.7961618	0.5989366
O	-2.5697417	1.5305127	-0.0279769
H	-2.5712488	1.1098120	-2.0455588
O	-0.8915256	1.2121292	1.4573585
H	-1.0162875	-0.7139397	3.5851869
H	-2.4714688	-0.8575378	2.6018747
H	-1.9312244	-2.2237606	3.5917306
H	3.5323275	2.0157555	2.1062819

O	0.4513729	1.6749338	-1.2383047
C	0.1358548	3.0760321	-1.1599615
H	1.0128203	3.6765978	-1.4048364
H	-0.6374912	3.2375836	-1.9049023
H	-0.2440458	3.3206727	-0.1674492
H	2.6322667	-1.8618615	0.5209528

$TS_{G\text{-}I1}$(H)

O	-0.7734962	-2.2010733	0.1843593
C	1.0368982	-0.4155531	-0.9004408
C	2.2281133	-0.5653836	-0.1631248
C	0.4999894	0.8727638	-1.1695985
C	2.9139893	0.5623947	0.2492111
C	1.2154549	1.9927011	-0.7441290
C	2.4046763	1.8309832	-0.0452386
H	0.8348662	2.9871702	-0.9307176
C	1.1093839	-3.1597943	-1.3793431
S	0.0742916	-1.7105372	-1.4356263
C	-0.1218632	-0.9217891	2.0179035
C	-0.9892928	-1.1567911	0.9921023
H	0.7566459	-1.5577490	2.0215292
C	-0.3434233	0.0079254	3.1501396
H	1.8691772	-3.0722027	-2.1563736
H	1.5426776	-3.2862703	-0.3906391
H	0.4440148	-3.9952964	-1.5996576
N	-2.1198881	-0.4010754	0.7163884
C	-3.2952267	-0.9439862	0.0429049
H	-3.0711656	-1.1992325	-0.9957329
H	-3.6777854	-1.8228942	0.5605130
C	-4.2224539	0.2598567	0.1517329
H	-4.8049252	0.2415367	1.0739700
C	-2.1275625	0.9881845	0.6682684
O	-3.3316104	1.3939750	0.2045398
H	-4.8796877	0.3880879	-0.7040240
O	-1.2264798	1.7354227	0.9557022
H	0.2296673	0.9283621	2.9963853
H	-1.3904930	0.2928682	3.2437588
H	-0.0054947	-0.4477994	4.0819313
H	2.9425000	2.7136755	0.2816797
O	-0.6672238	0.9046639	-1.8248543
C	-1.1595147	2.1943958	-2.2387325
H	-0.4328865	2.6780037	-2.8928412
H	-2.0731879	1.9864870	-2.7868462
H	-1.3775879	2.8100488	-1.3664164
H	2.6286467	-1.5493741	0.0434900
H	3.8419087	0.4592523	0.7979105

I_1(H)

O	-1.8784784	-0.1086261	1.3285036
C	0.9503803	0.4328142	1.4075684

C	1.1079870	1.8359178	1.5748075
C	1.5529925	-0.2186533	0.2822299
C	1.9203079	2.5426068	0.7128022
C	2.3560281	0.5221964	-0.5820674
C	2.5318166	1.8848937	-0.3641368
H	2.8219128	0.0489812	-1.4351912
C	-0.5141924	0.4849064	3.7476352
S	0.0624805	-0.5792926	2.4254993
C	-1.1389824	1.5479894	-0.1046384
C	-1.5411504	0.2273186	0.1325321
H	-1.2451269	2.1534493	0.7913912
C	-0.8167278	2.2424241	-1.3568811
H	0.3326240	0.8431382	4.3319013
H	-1.1128233	1.2955485	3.3390922
H	-1.1458235	-0.1527602	4.3661742
H	2.0726118	3.6047215	0.8613631
N	-1.6192685	-0.7664483	-0.8357133
C	-2.3299369	-2.0095844	-0.5361637
H	-1.8120046	-2.5692676	0.2454901
H	-3.3522045	-1.8095997	-0.2198810
C	-2.2383277	-2.6854368	-1.8942718
H	-3.0619842	-2.4023154	-2.5509492
C	-0.7393782	-0.9720761	-1.8985416
O	-1.0143243	-2.1661906	-2.4591450
H	-2.1514052	-3.7673462	-1.8454497
O	0.1587243	-0.2545179	-2.2624114
H	0.2836698	2.2897276	-1.4703507
H	-1.1903553	1.7359778	-2.2437144
H	-1.1760627	3.2725989	-1.3190277
H	3.1678330	2.4446361	-1.0415320
O	1.3041341	-1.5244835	0.1799095
C	2.0794167	-2.2812315	-0.7761950
H	3.1418753	-2.1687682	-0.5571608
H	1.7745064	-3.3130080	-0.6315396
H	1.8485786	-1.9555850	-1.7896647
H	0.6485176	2.3333649	2.4201565

$TS_{I1\text{-}H}$(H)

O	0.3264357	2.3137755	0.5326636
C	-1.4979155	0.2575550	-0.5721573
C	-2.4076178	0.3053432	0.5424188
C	-0.9043326	-1.0040704	-0.9077006
C	-2.9450179	-0.8745232	1.0593469
C	-1.4057226	-2.1597801	-0.3273097
C	-2.4347565	-2.0883673	0.6266342
H	-1.0022931	-3.1248997	-0.6028434
C	-2.1438095	2.9040316	-0.9766503
S	-1.0455554	1.5777650	-1.4985650
C	-0.5379553	0.6135114	1.8427398
C	0.4990969	1.1646518	1.0017660

H	-1.1742096	1.4321918	2.1687228
C	-0.4533444	-0.5629908	2.7300054
H	-3.1829787	2.6145703	-1.1245176
H	-1.9293772	3.1870791	0.0511015
H	-1.9022908	3.7385303	-1.6350072
H	-3.7154252	-0.8320801	1.8192827
N	1.6767840	0.5171563	0.6536896
C	2.6978446	1.2819319	-0.0648515
H	2.3545456	1.5164595	-1.0743599
H	2.9294576	2.2078747	0.4581124
C	3.8368270	0.2766717	-0.0512439
H	4.4632394	0.3742366	0.8362725
C	1.9500468	-0.8529550	0.5476754
O	3.1725261	-1.0038858	0.0075629
H	4.4511915	0.2935599	-0.9471586
O	1.2303108	-1.7746876	0.8332293
H	-0.6196674	-1.5031004	2.1907617
H	0.5553370	-0.6488057	3.1463931
H	-1.1780007	-0.4822704	3.5396733
H	-2.8329400	-3.0134481	1.0295034
O	0.0637301	-0.9368987	-1.8222110
C	0.5956089	-2.1835123	-2.3153578
H	-0.1989960	-2.7656678	-2.7840718
H	1.3381791	-1.9018764	-3.0552837
H	1.0615922	-2.7386549	-1.5011991
H	-2.8730071	1.2525391	0.7858730

H(H)

O	-0.3520884	-0.6803955	2.2844021
C	1.3104693	-0.1486562	-0.7102617
C	1.2924396	-1.5227903	-0.1572194
C	1.7221649	0.9249984	0.1306633
C	2.1292809	-1.7384646	1.0388810
C	2.4002771	0.6430204	1.3037496
C	2.6452366	-0.6913366	1.7279984
H	2.7848745	1.4589495	1.9043288
C	0.5397477	-1.2426417	-3.1253138
S	0.8280218	0.2990614	-2.2506873
C	-0.2401796	-1.8541497	0.2029282
C	-0.7429944	-0.7693031	1.1335382
H	-0.7873813	-1.8162043	-0.7342812
C	-0.3792313	-3.2258655	0.8480912
H	0.4503590	-0.9521292	-4.1721826
H	1.3885737	-1.9170689	-3.0125963
H	-0.3942679	-1.6958425	-2.8019428
H	2.2648852	-2.7560437	1.3839315
N	-1.6586116	0.1588401	0.6521946
C	-2.0885640	1.2538638	1.5167161
H	-2.3082917	0.8913427	2.5187692
H	-1.3017633	2.0097692	1.5775013

C	-3.3206798	1.7402641	0.7648023
H	-3.4197316	2.8216455	0.7342740
C	-2.2658463	0.2549996	-0.6112962
O	-3.1306095	1.2772911	-0.5898300
H	-4.2388319	1.2915639	1.1443163
O	-2.0617741	-0.4232338	-1.5874305
H	-1.4365869	-3.4949409	0.8898949
H	0.1344069	-3.9868070	0.2568768
H	0.0127855	-3.2292251	1.8638211
H	3.2282086	-0.8551218	2.6257297
O	1.4753602	2.1440272	-0.3616299
C	2.0227853	3.2703465	0.3430972
H	1.7283521	4.1398643	-0.2358826
H	1.6050234	3.3316330	1.3493109
H	3.1107009	3.1990107	0.3851893
H	1.5329505	-2.2625812	-0.9293519

23(H)

O	2.9021441	-1.9850320	-0.6712616
C	-1.4935471	-0.3999809	0.0899452
C	-0.2638903	-0.7774236	-0.4746561
C	-2.4353889	0.2887459	-0.7056428
C	0.0015460	-0.4826407	-1.8167542
C	-2.1401465	0.6083516	-2.0312023
C	-0.9243567	0.2097058	-2.5837498
H	-2.8514337	1.1444350	-2.6446369
C	-2.1101605	0.7444469	2.5241265
S	-1.9056830	-0.8739002	1.7387869
C	0.7503768	-1.5522093	0.3348275
C	2.1435813	-1.2029612	-0.1247435
H	0.6421662	-1.2408347	1.3775148
C	0.5147155	-3.0547334	0.2170896
H	-2.2698830	0.5424013	3.5840963
H	-1.2014076	1.3296784	2.3977188
H	-2.9739764	1.2634253	2.1156119
H	0.9361803	-0.8079508	-2.2644760
N	2.5656714	0.1200643	0.0348351
C	3.8568693	0.5376356	-0.4859431
H	4.6666757	0.1560234	0.1406129
H	3.9996029	0.1747353	-1.5021460
C	3.7014304	2.0487288	-0.3986888
H	3.3000962	2.4680890	-1.3233571
C	1.9833110	1.1341174	0.7939857
O	2.7351092	2.2566929	0.6459405
H	4.6141824	2.5697815	-0.1202241
O	1.0083164	1.0823995	1.4941209
H	1.2475000	-3.6143579	0.8004347
H	-0.4844760	-3.2895995	0.5874928
H	0.5887561	-3.3759595	-0.8224707
H	-0.7087493	0.4425130	-3.6206743

O	-3.6137511	0.6002817	-0.1047371
C	-4.5911458	1.2468309	-0.9050371
H	-4.2398890	2.2221727	-1.2526077
H	-4.8693690	0.6308881	-1.7643056
H	-5.4559073	1.3812892	-0.2599555

$TS_{I1\text{-}I}$(H)

O	0.3541967	-2.0046481	-1.0280333
C	-2.0136694	-0.1035777	0.2232967
C	-3.0137810	0.6061022	-0.4910446
C	-0.8713765	0.6422930	0.7202189
C	-2.9666347	1.9790573	-0.5586571
C	-0.8904366	2.0643599	0.6927646
C	-1.9180717	2.7104769	0.0547085
H	-0.0738275	2.6156180	1.1380885
C	-3.4721080	-2.3688211	-0.2399761
S	-2.0743022	-1.7284949	0.6985481
C	-0.2203917	0.2167596	-1.3736381
C	0.7326978	-0.8348908	-0.9558554
H	-1.0525655	-0.3174416	-1.8254256
C	0.0731714	1.4912999	-2.0648716
H	-4.4048264	-1.8960383	0.0631930
H	-3.2993624	-2.2610152	-1.3100147
H	-3.5140246	-3.4292975	0.0068513
H	-3.7651490	2.5110838	-1.0641698
N	2.0347770	-0.5707870	-0.5695091
C	2.9266412	-1.6974861	-0.3029379
H	2.5845840	-2.2459333	0.5777628
H	2.9604721	-2.3763888	-1.1526811
C	4.2297740	-0.9491386	-0.0697207
H	4.7950894	-0.8064877	-0.9910617
C	2.5554754	0.5949767	0.0003456
O	3.8109543	0.3526179	0.4010617
H	4.8630080	-1.3901987	0.6947566
O	1.9915532	1.6486231	0.1602962
H	-0.8404283	2.0898184	-2.1507261
H	0.8678845	2.0789560	-1.6189025
H	0.3399015	1.2224603	-3.0979379
H	-1.9314560	3.7938568	0.0233480
O	-0.0640889	-0.0682858	1.5211691
C	0.5307639	0.6094066	2.6546116
H	-0.2484944	1.1111073	3.2291280
H	0.9743698	-0.1862185	3.2465422
H	1.2939885	1.3139854	2.3329865
H	-3.8512480	0.0732409	-0.9231644

I(H)

O	-1.5131731	-2.2814082	-0.1233167
C	2.0649272	0.3228233	0.0311558
C	3.2509722	-0.2585509	-0.4525240

C	0.9179293	-0.5649970	0.4243116
C	3.4277434	-1.6201628	-0.3451766
C	1.2764181	-1.9817790	0.6680846
C	2.4589576	-2.4873899	0.2376040
H	0.5072111	-2.6080292	1.1061646
C	3.2245077	2.7348521	-0.5198353
S	1.7501296	1.9595141	0.1649900
C	-0.0325721	-0.5496932	-0.8680171
C	-1.3651216	-1.0992047	-0.3792162
H	-0.1359385	0.5076978	-1.1091109
C	0.4389464	-1.3443304	-2.0757215
H	4.1048523	2.4831848	0.0702467
H	3.3544115	2.4589393	-1.5653837
H	3.0384034	3.8060739	-0.4504475
H	4.3674661	-2.0445824	-0.6867563
N	-2.4304386	-0.2296649	-0.2891064
C	-3.7176548	-0.7321442	0.1833580
H	-4.0344519	-1.5959839	-0.3971983
H	-3.6424229	-1.0175988	1.2347713
C	-4.5858244	0.4973083	-0.0373439
H	-5.3263569	0.6582192	0.7409817
C	-2.4190568	1.1819941	-0.2788145
O	-3.6617063	1.6054125	-0.0021349
H	-5.0661551	0.4921747	-1.0162195
O	-1.4876161	1.9199888	-0.4658819
H	1.4467709	-1.0571525	-2.3797864
H	0.4077206	-2.4163018	-1.8875503
H	-0.2267483	-1.1277319	-2.9136670
H	2.6850546	-3.5408877	0.3477989
O	0.1272298	0.0133362	1.4317287
C	0.7392207	-0.0399318	2.7284015
H	1.7088374	0.4661564	2.7293696
H	0.0605584	0.4792004	3.4002002
H	0.8653663	-1.0744596	3.0560074
H	4.0565328	0.3530090	-0.8374758

$TS_{I\text{-}H1}$(H)

O	0.4840326	1.5488235	-1.0218740
C	-2.0136746	0.4836446	0.4969048
C	-1.3238753	1.4510939	1.2194834
C	-1.4937704	-0.8556284	0.5087883
C	-0.1613195	1.1381345	1.9413630
C	-0.2463861	-1.1311706	1.1595791
C	0.3875188	-0.1324790	1.9103468
H	0.1160167	-2.1493145	1.2161541
C	-3.7366380	2.4792715	-0.2078159
S	-3.4954065	0.7028791	-0.3609211
C	0.1294754	-0.7984837	-0.9597570
C	0.9472775	0.4413061	-0.7914550
H	0.6886814	-1.7231275	-0.8500304

C	-0.8107815	-0.7522929	-2.1168344
H	-3.9070828	2.7654803	0.8296199
H	-2.8951388	3.0255765	-0.6332155
H	-4.6346471	2.6959309	-0.7857455
H	0.3092339	1.9149808	2.5344904
N	2.2746444	0.3027037	-0.4339420
C	3.1235554	1.4831107	-0.3135869
H	3.0440090	2.1110064	-1.1990232
H	2.8333538	2.0665163	0.5635379
C	4.4854960	0.8178746	-0.1615249
H	5.1438197	1.3167507	0.5438334
C	2.9278154	-0.8308717	0.0799664
O	4.1915433	-0.4962236	0.3652789
H	4.9896539	0.6883172	-1.1193290
O	2.4536280	-1.9214260	0.2770778
H	-1.5234449	-1.5763922	-2.1191208
H	-1.3310640	0.1999873	-2.1987226
H	-0.1761124	-0.8762183	-3.0055252
H	1.2908962	-0.3767481	2.4558146
O	-2.3231217	-1.7826248	0.0592739
C	-1.9619717	-3.1717825	0.2080166
H	-1.8221356	-3.4125002	1.2615856
H	-2.8052429	-3.7241138	-0.1942559
H	-1.0606424	-3.3991589	-0.3635070
H	-1.7054452	2.4627779	1.2616313

H_1(H)

O	-2.2186639	1.5084456	0.9058038
C	0.8604010	-1.4210856	0.1883267
C	0.8666867	-2.2710864	-0.9070446
C	-0.3918357	-1.2243470	0.8278543
C	-0.3107235	-2.8696563	-1.4332324
C	-1.6287000	-1.4875709	0.0872412
C	-1.5340960	-2.5281039	-0.9510468
H	-2.5119098	-1.5607600	0.7213197
C	3.4846655	-0.9091776	-0.3570815
S	2.2397999	-0.6543880	0.9201958
C	-1.7993158	-0.1152206	-0.8015372
C	-1.4574078	1.0968695	0.0441396
H	-1.0931164	-0.2082628	-1.6241427
C	-3.2374367	-0.0148467	-1.2854046
H	3.7784105	-1.9555556	-0.4266811
H	3.1270648	-0.5266322	-1.3138296
H	4.3465160	-0.3249862	-0.0345823
H	-0.2165343	-3.5821703	-2.2434304
N	-0.2410141	1.7229884	-0.1467944
C	0.0878796	2.8963353	0.6590065
H	-0.5252496	3.7446211	0.3500127
H	-0.0905352	2.6983927	1.7143809
C	1.5652012	3.0688623	0.3250978

H	2.2111766	2.6100696	1.0736622
C	0.7568058	1.4846077	-1.1140778
O	1.7550479	2.3546165	-0.9142064
H	1.8571443	4.1022211	0.1591445
O	0.7582320	0.6579566	-1.9902449
H	-3.5083680	-0.8765257	-1.8941426
H	-3.9282246	0.0760502	-0.4472687
H	-3.3408671	0.8790766	-1.9043971
H	-2.4480392	-2.9190888	-1.3807717
O	-0.3588738	-0.6835962	2.0077385
C	-1.5322919	-0.7077027	2.8817213
H	-1.8785783	-1.7358936	2.9718528
H	-1.1566746	-0.3480013	3.8337397
H	-2.2973091	-0.0429098	2.4910969
H	1.8150135	-2.5166449	-1.3711383

22(H)

O	2.9003172	1.6538077	1.4099201
C	-2.4511121	-0.3076692	0.3503333
C	-2.2128064	-1.1789731	1.4173243
C	-1.4106645	0.5260746	-0.0973948
C	-0.9712091	-1.1829309	2.0483287
C	-0.1672405	0.5330454	0.5417040
C	0.0382217	-0.3240060	1.6274034
C	-4.9901640	-1.3798506	0.3456700
S	-3.9994052	-0.1129578	-0.4589221
C	0.8717459	1.5438295	0.1116640
C	2.2451126	1.0929972	0.5492437
H	0.8610482	1.6018535	-0.9788358
C	0.5561068	2.9147576	0.7058960
H	-5.9687100	-1.3314809	-0.1323033
H	-4.5716507	-2.3746305	0.1926469
H	-5.1102221	-1.1807230	1.4105486
H	-0.8011970	-1.8502512	2.8859889
N	2.7691695	-0.0720450	-0.0175196
C	4.0418633	-0.5954771	0.4524169
H	4.8688635	0.0149173	0.0819513
H	4.0768637	-0.6083424	1.5402131
C	4.0040662	-1.9840561	-0.1688877
H	3.5669830	-2.7195918	0.5090936
C	2.3202004	-0.7805365	-1.1304360
O	3.1356456	-1.8514195	-1.3077644
H	4.9705853	-2.3341212	-0.5228943
O	1.3991057	-0.5274529	-1.8592233
H	1.2965734	3.6545787	0.3979559
H	-0.4287243	3.2297914	0.3607970
H	0.5555528	2.8670210	1.7948871
H	0.9843885	-0.3068396	2.1598784
O	-1.6624298	1.4152235	-1.1122372
C	-1.5313348	0.8380405	-2.4246230

H	-0.4928237	0.5763794	-2.6237255
H	-2.1575942	-0.0511468	-2.5179606
H	-1.8756497	1.6010906	-3.1199141
H	-2.9921653	-1.8375256	1.7774063

G(Cl)

O	0.5079189	2.0778335	-0.8627133
C	-0.9661483	-0.1324842	-0.8998208
C	-2.0991775	0.4873534	-0.3610159
C	-0.6183869	-1.4643699	-0.6018280
C	-2.9211706	-0.2515642	0.4739492
C	-1.4793194	-2.1847074	0.2319426
C	-2.6138677	-1.5851606	0.7596814
H	-1.2608337	-3.2111745	0.4925651
C	-0.8025003	1.6885154	-2.9978399
S	0.1797443	0.7794414	-1.8262034
C	0.4955520	2.1959397	1.4986639
C	1.0131615	1.6567214	0.3947125
H	-0.3542469	2.8570822	1.3643483
C	1.0248243	1.9609241	2.8705278
H	-1.1616794	0.9620730	-3.7281241
H	-1.6278673	2.1915026	-2.4995186
H	-0.1349975	2.4065953	-3.4751152
N	1.9763246	0.6690258	0.2828122
C	3.3125314	0.8806187	-0.2666898
H	3.2744381	1.2965475	-1.2738804
H	3.8897080	1.5473524	0.3795413
C	3.8239832	-0.5562262	-0.2468098
H	4.8792122	-0.6424620	-0.0019842
C	1.9205213	-0.5204619	1.0126255
O	3.0688665	-1.2005806	0.7983220
H	3.6112702	-1.0705429	-1.1857817
O	1.0149237	-0.9064358	1.7026726
H	0.3507464	1.3050619	3.4267455
H	2.0080293	1.4924815	2.8564409
H	1.0947170	2.9055090	3.4116976
H	-3.2669244	-2.1518280	1.4131603
O	0.5210741	-1.9368442	-1.1362398
C	0.9272293	-3.2576543	-0.7357653
H	0.1988965	-3.9966568	-1.0721520
H	1.8771051	-3.4282341	-1.2334552
H	1.0585189	-3.2981496	0.3461524
H	-2.3242469	1.5285409	-0.5596313
Cl	-4.3119701	0.4776974	1.1655075

$TS_{G\text{-}I1}$(Cl)

O	-0.7547213	-2.2067820	0.1912849
C	1.0215186	-0.4143073	-0.9137369
C	2.1894309	-0.5730662	-0.1450764
C	0.4898439	0.8721371	-1.1929073

C	2.8602356	0.5577145	0.2883993
C	1.2035931	1.9887432	-0.7519159
C	2.3718851	1.8317678	-0.0205483
H	0.8355004	2.9863920	-0.9477776
C	1.1041463	-3.1586543	-1.4035711
S	0.0595811	-1.7155496	-1.4384414
C	-0.1048496	-0.9239063	2.0230028
C	-0.9711705	-1.1589701	0.9962266
H	0.7716902	-1.5627956	2.0318155
C	-0.3249990	0.0116587	3.1504533
H	1.8489091	-3.0629079	-2.1942417
H	1.5566551	-3.2852438	-0.4234563
H	0.4400761	-3.9977778	-1.6143993
N	-2.0985303	-0.4026132	0.7129517
C	-3.2826144	-0.9483939	0.0565590
H	-3.0704323	-1.2134048	-0.9820705
H	-3.6616611	-1.8216593	0.5861966
C	-4.2061392	0.2587500	0.1647140
H	-4.7819686	0.2477491	1.0911721
C	-2.1060218	0.9864681	0.6605023
O	-3.3124803	1.3914870	0.2041490
H	-4.8691212	0.3832172	-0.6871168
O	-1.2013129	1.7332736	0.9383698
H	0.2539539	0.9281326	2.9933949
H	-1.3706734	0.3028563	3.2406591
H	0.0098453	-0.4407063	4.0849417
H	2.9111565	2.7056536	0.3267704
O	-0.6673182	0.9096758	-1.8646500
C	-1.1552195	2.2036692	-2.2709031
H	-0.4235415	2.6922750	-2.9156404
H	-2.0648338	2.0013774	-2.8277485
H	-1.3791828	2.8117199	-1.3946183
H	2.5925446	-1.5516696	0.0796143
Cl	4.2897458	0.3916501	1.2221924

I_1(Cl)

O	-1.8889354	-0.2437483	1.3073171
C	0.9186129	0.3529701	1.4342939
C	0.9806017	1.7545697	1.6555254
C	1.5658265	-0.2195833	0.2922149
C	1.7561810	2.5375471	0.8243918
C	2.3406947	0.6018300	-0.5242817
C	2.4301465	1.9646881	-0.2638548
H	2.8497592	0.1938126	-1.3866273
C	-0.5623320	0.2420872	3.7627876
S	0.0483500	-0.7321109	2.3872793
C	-1.2057704	1.5130612	-0.0342047
C	-1.5485684	0.1650848	0.1333780
H	-1.3591758	2.0719959	0.8851965
C	-0.8901563	2.2760527	-1.2467063

H	0.2747686	0.6014565	4.3604766
H	-1.1927464	1.0490259	3.3969055
H	-1.1663814	-0.4493679	4.3504053
N	-1.5677511	-0.7842152	-0.8798124
C	-2.2376869	-2.0658011	-0.6561161
H	-1.7099855	-2.6480681	0.1020731
H	-3.2702367	-1.9182394	-0.3445024
C	-2.1052257	-2.6650484	-2.0465387
H	-2.9303511	-2.3768300	-2.6991052
C	-0.6693853	-0.9016269	-1.9410752
O	-0.8937494	-2.0736018	-2.5660954
H	-1.9798248	-3.7442427	-2.0533094
O	0.2064236	-0.1340429	-2.2538649
H	0.2106534	2.3553253	-1.3472379
H	-1.2381930	1.8020257	-2.1619051
H	-1.2747723	3.2942330	-1.1644251
H	3.0344797	2.5972173	-0.9052274
O	1.3820370	-1.5292488	0.1252876
C	2.1949377	-2.1976882	-0.8648665
H	3.2504090	-2.0509467	-0.6325946
H	1.9355534	-3.2482595	-0.7789989
H	1.9542309	-1.8271308	-1.8606599
H	0.4862576	2.2041609	2.5076379
Cl	1.8456445	4.2298869	1.0897994

$TS_{I1\text{-}H}$(Cl)

O	-0.7510017	2.2654179	-0.7407711
C	0.7180500	0.4857444	1.0417159
C	1.8892547	0.6025206	0.2172641
C	0.1956369	-0.8180649	1.3020929
C	2.6729021	-0.5269127	-0.0348044
C	0.9734820	-1.9247006	0.9836091
C	2.2109004	-1.7780102	0.3408975
H	0.6223396	-2.9212332	1.2160261
C	0.9337755	3.2189474	1.3887905
S	-0.1678278	1.8064574	1.5676736
C	0.5945515	0.6385154	-1.6714604
C	-0.6722061	1.0767976	-1.1695940
H	1.2211687	1.5119674	-1.8358102
C	0.8957171	-0.5572472	-2.4879267
H	1.8521661	3.0414391	1.9465700
H	1.1138424	3.4292570	0.3377808
H	0.3898272	4.0521502	1.8340690
N	-1.8136528	0.3010030	-1.0295034
C	-3.1108923	0.9559667	-0.8466899
H	-3.0334778	1.7923372	-0.1571475
H	-3.4751202	1.3183877	-1.8101200
C	-3.9433753	-0.1932940	-0.2912936
H	-4.9395948	-0.2575767	-0.7207228
C	-1.9422404	-1.0885473	-0.9376732

O	-3.2234532	-1.3868971	-0.6544082
H	-4.0082828	-0.1608548	0.7972665
O	-1.0766503	-1.9176580	-1.0462950
H	1.0639691	-1.4462338	-1.8685886
H	0.0423549	-0.8137553	-3.1214368
H	1.7746655	-0.3803551	-3.1064263
H	2.8103389	-2.6563018	0.1269412
O	-1.0091930	-0.8446648	1.8771593
C	-1.5223706	-2.1346030	2.2692420
H	-0.8516628	-2.5974833	2.9942932
H	-2.4830288	-1.9297729	2.7317056
H	-1.6506244	-2.7688315	1.3914688
H	2.3201142	1.5812289	0.0455632
Cl	4.1397283	-0.3401297	-0.9036570

H(Cl)

O	0.4134285	-1.1708344	1.9388298
C	0.4282116	0.9782044	-0.8367240
C	1.5425068	0.0039730	-0.7206284
C	0.1036894	1.7785892	0.2877483
C	2.5332111	0.3152422	0.3317256
C	0.9974522	1.8569172	1.3475125
C	2.2321083	1.1727845	1.3487467
H	0.7746813	2.5094469	2.1839171
C	0.1207110	0.1060981	-3.4437516
S	-0.6011970	1.1302621	-2.1549071
C	0.8026159	-1.4043503	-0.4113734
C	-0.0613774	-1.2185358	0.8176926
H	0.1663526	-1.5730903	-1.2754029
C	1.7512575	-2.5759945	-0.2282386
H	1.1814558	0.3230534	-3.5678400
H	-0.0636671	-0.9481658	-3.2462553
H	-0.4108059	0.3931619	-4.3512724
N	-1.4266501	-1.0304618	0.6406558
C	-2.2706460	-0.7983724	1.8079437
H	-2.3197371	-1.6977267	2.4252001
H	-1.8790196	0.0191134	2.4118722
C	-3.5909793	-0.4670747	1.1258378
H	-3.7148657	0.6049620	0.9663714
C	-2.2186607	-1.3232336	-0.4859745
O	-3.5008291	-1.0965664	-0.1696205
H	-4.4622333	-0.8732261	1.6321791
O	-1.8561987	-1.7145436	-1.5666612
H	2.4580050	-2.6486862	-1.0554410
H	2.2972737	-2.5157268	0.7107636
H	1.1523194	-3.4900818	-0.2157381
H	2.9453094	1.3487132	2.1446584
O	-1.0526627	2.4460848	0.1897308
C	-1.3361854	3.4392242	1.1892065
H	-0.5468517	4.1922023	1.2108833

H	-2.2739886	3.8926723	0.8836928
H	-1.4478194	2.9757479	2.1710361
H	2.0449719	-0.1704134	-1.6764749
Cl	4.0574632	-0.4297089	0.2342296

23(Cl)

O	-3.0437342	0.1816591	1.8274850
C	1.5882457	-0.0182446	0.4415448
C	0.2933777	0.5249568	0.5854634
C	2.4905877	0.5259002	-0.4964340
C	-0.0344550	1.6408603	-0.1980352
C	2.0992530	1.5896018	-1.3073218
C	0.8401002	2.1561636	-1.1441359
H	2.7701803	2.0103463	-2.0437240
C	2.5358992	-2.6259992	0.3460509
S	2.1305881	-1.3070199	1.5184698
C	-0.6539619	-0.0652908	1.5990802
C	-2.0932555	-0.1457353	1.1437952
H	-0.3272532	-1.1033617	1.7389130
C	-0.5608388	0.6628111	2.9375265
H	3.4249559	-2.3762763	-0.2269868
H	2.7254252	-3.5094855	0.9570842
H	1.6842723	-2.8086403	-0.3057827
N	-2.3339493	-0.6194181	-0.1514574
C	-3.6597930	-0.4963235	-0.7320290
H	-4.3513355	-1.2148915	-0.2850661
H	-4.0478982	0.5106460	-0.5876284
C	-3.3277813	-0.8168053	-2.1807399
H	-3.0458822	0.0800760	-2.7361084
C	-1.5388008	-1.4590850	-0.9229231
O	-2.1774735	-1.6801220	-2.1019730
H	-4.1127117	-1.3534137	-2.7080462
O	-0.4841891	-1.9629057	-0.6374831
H	0.4686163	0.6207165	3.2975542
H	-0.8541502	1.7080408	2.8291174
H	-1.2154303	0.2001144	3.6761983
H	0.5414338	3.0101947	-1.7399417
O	3.7259606	-0.0349724	-0.5423952
C	4.6568694	0.5382641	-1.4483095
H	4.8361735	1.5914749	-1.2166946
H	5.5778847	-0.0240101	-1.3164678
H	4.3157711	0.4425957	-2.4825933
Cl	-1.5509713	2.4670384	0.0023442

$TS_{I1\text{-}I}$(Cl)

O	0.3080971	-2.0123030	-1.0147827
C	-1.9899105	-0.0974830	0.2385914
C	-2.9859000	0.5978377	-0.4888457
C	-0.8745386	0.6563237	0.7826472
C	-2.9396121	1.9727020	-0.5581238

C	-0.8958711	2.0730116	0.7400335
C	-1.9066255	2.7164268	0.0738347
H	-0.0971841	2.6371042	1.2017559
C	-3.4031543	-2.3779981	-0.2833535
S	-2.0452511	-1.7267130	0.7039502
C	-0.2367157	0.1968312	-1.4337500
C	0.6975131	-0.8398051	-0.9791512
H	-1.0789420	-0.3334629	-1.8716050
C	0.0316706	1.4903438	-2.0868012
H	-4.3514836	-1.9197979	-0.0070955
H	-3.1955234	-2.2593766	-1.3461439
H	-3.4386990	-3.4409178	-0.0459759
N	1.9967695	-0.5787862	-0.5753298
C	2.8775739	-1.7069909	-0.2804583
H	2.5228863	-2.2388225	0.6054404
H	2.9152818	-2.4002011	-1.1185075
C	4.1832774	-0.9636472	-0.0459734
H	4.7579910	-0.8376397	-0.9639156
C	2.5182315	0.5911660	-0.0180360
O	3.7673845	0.3475564	0.4018069
H	4.8067062	-1.3969026	0.7309365
O	1.9584215	1.6505332	0.1230217
H	-0.8744518	2.1114306	-2.0736476
H	0.8685368	2.0463102	-1.6797235
H	0.2001174	1.2730565	-3.1524683
H	-1.9326009	3.7992626	0.0269191
O	-0.0723059	-0.0510353	1.5769208
C	0.5875447	0.6345694	2.6710885
H	-0.1592492	1.1511382	3.2745032
H	1.0501959	-0.1592950	3.2501423
H	1.3403460	1.3262912	2.3012144
H	-3.8127378	0.0678091	-0.9444418
Cl	-4.1620770	2.8237724	-1.3981548

I(Cl)

O	-1.5497174	-2.3939039	-0.0380750
C	1.4749528	0.8433232	0.1116942
C	2.7653281	0.4862128	-0.2892561
C	0.4850517	-0.2248490	0.4989214
C	3.1800519	-0.8232328	-0.1205798
C	1.0950277	-1.5375661	0.8260291
C	2.3646862	-1.8380662	0.4727550
H	0.4394871	-2.2801616	1.2679116
C	2.2029086	3.4039348	-0.5009528
S	0.8529356	2.3996868	0.1406080
C	-0.4068060	-0.4329981	-0.8055797
C	-1.6249421	-1.2142179	-0.3370957
H	-0.7035956	0.5749634	-1.0935238
C	0.2577285	-1.1594836	-1.9654856
H	3.0763914	3.3399016	0.1470649

H	2.4448385	3.1172167	-1.5236907
H	1.8240493	4.4254499	-0.4941537
N	-2.8406442	-0.5667644	-0.3093107
C	-4.0221828	-1.2932361	0.1471687
H	-3.9305372	-1.5219645	1.2110749
H	-4.1453807	-2.2218043	-0.4062112
C	-5.1033230	-0.2644771	-0.1477280
H	-5.8879731	-0.2238084	0.6024411
C	-3.1026659	0.8200063	-0.3568290
O	-4.4127795	1.0027309	-0.1321865
H	-5.5388235	-0.3970462	-1.1385483
O	-2.3253568	1.7177758	-0.5480542
H	-0.4020764	-1.1088380	-2.8337967
H	1.2006354	-0.6875061	-2.2485199
H	0.4316738	-2.2095142	-1.7348172
H	2.7884749	-2.8212064	0.6381628
O	-0.4237112	0.2344120	1.4683866
C	0.1534167	0.3530872	2.7760721
H	-0.6286396	0.7608204	3.4113410
H	0.4649767	-0.6234440	3.1545217
H	1.0087136	1.0348852	2.7709700
H	3.4716829	1.2131681	-0.6689310
Cl	4.7498235	-1.2494460	-0.5883975

$TS_{I\text{-}H1}$(Cl)

O	-0.3244123	0.5519347	1.7905998
C	2.0361098	0.3819712	-0.1495512
C	1.2887916	1.5361287	-0.3368318
C	1.5509229	-0.8367482	-0.7349559
C	0.0905229	1.5127631	-1.0748118
C	0.2720062	-0.8531991	-1.3794582
C	-0.4277818	0.3408926	-1.5982952
H	-0.0663313	-1.7515535	-1.8799167
C	3.7282756	1.9413322	1.3135351
S	3.5476063	0.2687945	0.6757895
C	-0.0497253	-1.5117190	0.6498602
C	-0.8342549	-0.3245414	1.1058436
H	-0.6410325	-2.2814628	0.1618416
C	0.9477903	-2.0011921	1.6450585
H	3.8383238	2.6615001	0.5030975
H	2.8925929	2.2025643	1.9625539
H	4.6471527	1.9257677	1.8990548
N	-2.1746035	-0.2642877	0.7833928
C	-2.9845413	0.8618970	1.2381201
H	-2.8652166	1.0189428	2.3085510
H	-2.6904605	1.7711361	0.7081323
C	-4.3717829	0.3670256	0.8487821
H	-5.0245372	1.1451196	0.4636705
C	-2.8780705	-1.0285897	-0.1647099
O	-4.1350686	-0.5767689	-0.2215540

H	-4.8628050	-0.1680895	1.6616910
O	-2.4436611	-1.9182238	-0.8527465
H	1.6184783	-2.7577919	1.2402504
H	0.3532053	-2.4903789	2.4294964
H	1.5176335	-1.1953788	2.1046144
H	-1.3560402	0.3319024	-2.1556514
O	2.4183443	-1.8305995	-0.7645027
C	2.0852470	-3.0228552	-1.5077650
H	2.9569958	-3.6626477	-1.4148751
H	1.2127917	-3.5144993	-1.0745506
H	1.9091640	-2.7736178	-2.5539221
H	1.6261896	2.4837550	0.0622528
Cl	-0.7473391	2.9875371	-1.3059101

H_1(Cl)

O	-0.0426896	-0.4523294	1.6863864
C	1.9929069	0.5514150	0.0653672
C	1.0849202	1.5133925	-0.3531055
C	1.8625904	-0.7449202	-0.5045975
C	0.0400536	1.2316516	-1.2775639
C	0.6557387	-1.1294905	-1.2433982
C	-0.1534218	-0.0160976	-1.7779452
H	0.8610549	-1.8970299	-1.9949392
C	3.0811585	2.5052857	1.6263716
S	3.3352325	0.7947045	1.1315941
C	-0.3775172	-1.8568710	-0.2102186
C	-0.8153084	-0.8280378	0.8160778
H	-1.2101828	-2.1393993	-0.8522552
C	0.1865809	-3.0748053	0.5035200
H	3.1900040	3.1873326	0.7836220
H	2.1147185	2.6301657	2.1145770
H	3.8711999	2.7104567	2.3484738
N	-2.1115092	-0.3603400	0.7955457
C	-2.5519037	0.6051808	1.7979475
H	-2.3006489	0.2663133	2.8010577
H	-2.0800246	1.5740471	1.6175784
C	-4.0484228	0.6182736	1.5179736
H	-4.5047324	1.5998113	1.6093037
C	-3.0707408	-0.4707544	-0.2335760
O	-4.1632283	0.2071784	0.1383970
H	-4.5878852	-0.1053774	2.1295928
O	-2.9536916	-1.0431705	-1.2853944
H	0.4131484	-3.8708860	-0.2056363
H	-0.5804258	-3.4581893	1.1806625
H	1.0625233	-2.8357973	1.1074708
H	-0.9798320	-0.2451451	-2.4393471
O	2.8579589	-1.5473376	-0.2746236
C	3.0899117	-2.7464104	-1.0646220
H	4.1113940	-3.0259912	-0.8286800
H	2.4028332	-3.5320603	-0.7711135

H	2.9957462	-2.5039986	-2.1221498
H	1.1781552	2.5331409	0.0019284
Cl	-0.9999051	2.5265192	-1.7174222

22(Cl)

O	3.0634232	0.2148791	1.7846065
C	-1.5754100	-0.9205253	0.0918348
C	-2.5874093	-0.0405836	-0.2927524
C	-0.3652129	-0.4120716	0.5980822
C	-2.3596074	1.3292725	-0.1921164
C	-0.1337636	0.9655250	0.6634599
C	-1.1505197	1.8358140	0.2599774
C	-3.3366106	-2.8944252	-0.6668286
S	-1.6663172	-2.6698188	-0.0381869
C	1.1661619	1.5652451	1.1515441
C	2.3389457	0.6410518	0.9039112
H	1.3343938	2.4564091	0.5385435
C	1.0961174	1.9742761	2.6200354
H	-3.4708249	-3.9719920	-0.7617619
H	-4.0813725	-2.5056601	0.0276143
H	-3.4589655	-2.4367174	-1.6483895
N	2.5537370	0.2448983	-0.4122455
C	3.3738805	-0.9170567	-0.7046604
H	4.4278995	-0.6418318	-0.7877752
H	3.2546906	-1.6648113	0.0772575
C	2.7644569	-1.3339558	-2.0347203
H	3.4819153	-1.7377329	-2.7448920
C	1.9770321	0.7549388	-1.5756807
O	2.2368338	-0.1119441	-2.5874873
H	1.9355233	-2.0305227	-1.8929320
O	1.3823130	1.7892777	-1.7238509
H	2.0356319	2.4274338	2.9381067
H	0.2895455	2.6963684	2.7564933
H	0.9008829	1.1177307	3.2636254
H	-0.9851373	2.9072403	0.2766738
O	0.6081848	-1.3244798	0.9378170
C	0.5278111	-1.7706143	2.3006408
H	1.1246419	-2.6789144	2.3613248
H	0.9465413	-1.0162911	2.9680199
H	-0.5070877	-1.9908875	2.5707366
H	-3.5333607	-0.3909738	-0.6821419
Cl	-3.6114342	2.4211195	-0.6798132

9.References

(1) Mulder, J. A.; Hsung, R. P.; Frederick, M. O.; Tracey, M. R.; Zificsak, C. A. *Org. Lett.* **2002**, *4* (8), 1383–1386.

(2) Istrate, F. M.; Buzas, A. K.; Jurberg, I. D.; Odabachian, Y.; Gagosz, F. *Org. Lett.* **2008**, *10* (5), 925–928.

(3) Peng, B.; Huang, X.; Xie, L. G.; Maulide, N. *Angew. Chem. Int. Ed.* **2014**, *53* (33), 8718–8721.

(4) Lai, P.-S.; Dubland, J. A.; Sarwar, M. G.; Chudzinski, M. G.; Taylor, M. S. *Tetrahedron* **2011**, *67* (39), 7586–7592.

(5) N. D. Buezo, I. Alonso, J. C. Carretero, *J. Am. Chem. Soc.* **1998**, *120*, 7129–7130.

(6) Serreqi, A. N.; Kazlauskas, R. J. *Can. J. Chem. Can. Chim.* **1995**, *73* (8), 1357–1367.

(7) Yoshida, S.; Uchida, K.; Hosoya, T. *Chem. Lett.* **2014**, *43* (1), 116–118.

(8) Shi, F.; Tse, M. K.; Kaiser, H. M.; Beller, M. *Adv. Synth. Catal.* **2007**, *349* (16), 2425–2430.

(9) P. Jacob III, A. T. Shulgin, *Synth. Commun.* **1981**, *11*, 957-968.

(10) K. W. Bentley, W. I. Rushworth, *Ger. Offen.* **1972**, 23. Patent DE2160148.

(11) Bruker SAINT V8.32B and V7.68A. Copyright 2005-2015 Bruker AXS.

(12) Sheldrick, G. M. SADABS. University of Göttingen, Germany 1996.

(13) Dolomanov, O. V.; Bourhis, L. J.; Gildea, R. J.; Howard, J. A. K.; Puschmann, H.; IUCr. *J. Appl. Crystallogr.* **2009**, *42* (2), 339–341.

(14) Hübschle, C. B.; Sheldrick, G. M.; Dittrich, B.; IUCr. *J. Appl. Crystallogr.* **2011**, *44* (6), 1281–1284.

(15) Sheldrick, G. M. SHELXS. University of Göttingen, Germany 1996.

(16) Sheldrick, G. M. SHELXL. University of Göttingen, Germany 1996.

(17) Spek, A. L.; IUCr. *Acta Crystallogr. Sect. D Biol. Crystallogr.* **2009**, *65* (2), 148–155.

(18) Banks, J. L.; Beard, H. S.; Cao, Y.; Cho, A. E.; Damm, W.; Farid, R.; Felts, A. K.; Halgren, T. A.; Mainz, D. T.; Maple, J. R.; Murphy, R.; Philipp, D. M.; Repasky, M. P.; Zhang, L. Y.; Berne, B. J.; Friesner, R. A.; Gallicchio, E.; Levy, R. M. *J. Comput. Chem.* **2005**, *26* (16), 1752–1780.

(19) MacroModel. Schrödinger, LLC, New York 2017.

(20) Becke, A. *J. Chem. Phys.* **1993**, *98*, 5648–5652.

(21) Lee, C.; Yang, W.; Parr, R. G. *Phys. Rev. B* **1988**, *37* (2), 785–789.

(22) Vosko, S. H.; Wilk, L.; Nusair, M. *Can. J. Phys.* **1980**, *58* (8), 1200–1211.

(23) Stephens, P. J.; Devlin, F. J.; Chabalowski, C. F.; Frisch, M. J. *J. Phys. Chem.* **1994**, *98* (45), 11623–11627.

(24) Grimme, S.; Antony, J.; Ehrlich, S.; Krieg, H. *J. Chem. Phys.* **2010**, *132* (15), 154104.

(25) Hehre, W. J.; Ditchfield, R.; Pople, J. A. *J. Chem. Phys.* **1972**, *56* (5), 2257–2261.

(26) Weigend, F.; Häser, M. *Theor. Chem. Accounts Theory, Comput. Model. (Theoretica Chim. Acta)* **1997**, *97* (1–4), 331–340.

(27) Adamo, C.; Scuseria, G. E.; Barone, V. *J. Chem. Phys.* **1999**, *111* (7), 2889.

(28) Zhao, Y.; Truhlar, D. G. *Theor. Chem. Acc.* **2008**, *120* (1–3), 215–241.

(29) Riplinger, C.; Neese, F. *J. Chem. Phys.* **2013**, *138* (3), 34106.

(30) Riplinger, C.; Sandhoefer, B.; Hansen, A.; Neese, F. *J. Chem. Phys.* **2013**, *139* (13), 134101.

(31) Kendall, R. A.; Dunning, T. H.; Harrison, R. J. *J. Chem. Phys.* **1992**, *96* (9), 6796–6806.

(32) Marenich, A. V.; Cramer, C. J.; Truhlar, D. G. *J. Phys. Chem. B* **2009**, *113* (18), 6378–6396.

(33) Klamt, A.; Schüürmann, G. *J. Chem. Soc. Perkin Trans. 2* **1993**, No. 5, 799–805.

(34) Frisch; Trucks, G.; Schlegel, H.; Scuseria, G.; Robb, M.; Cheeseman, J.; Scalmani, G.; Barone, V.; Mennucci, B.; Petersson, G.; Nakatsuji, H.; Caricato, M.; Li, X.; Hratchian, H.; Izmaylov, A.; Bloino, J.; Zheng, G.; Sonnenberg, J.; Hada, M.; Ehara, M.; Toyota, K.; Fukuda, R.; Hasegawa, J.; Ishida, M.; Nakajima, T.; Honda, Y.; Kitao, O.; Nakai, H.; Vreven, T.; Montgomery, J.; Peralta, J.; Ogliaro, F.; Bearpark, M.; Heyd, J.; Brothers, E.; Kudin, K.; Staroverov, V.; Kobayashi, R.; Normand, J.; Raghavachari, K.; Rendell, A.; Burant, J.; Iyengar, S.; Tomasi, J.; Cossi, M.; Rega, N.; Millam, J.; Klene, M.; Knox, J.; Cross, J.; Bakken, V.; Adamo, C.; Jaramillo, J.; Gomperts, R.; Stratmann, R.; Yazyev, O.; Austin, A.; Cammi, R.; Pomelli, C.; Ochterski, J.; Martin, R.; Morokuma, K.; Zakrzewski, V.; Voth, G.; Salvador, P.; Dannenberg, J.; Dapprich, S.; Daniels, A.; Farkas; Foresman, J.; Ortiz, J.; Cioslowski, J.; Fox, D. *Gaussian 09, Revis. B.01, Gaussian, Inc., Wallingford CT* **2013**.

(35) TURBOMOLE V7.2 2017, a development of University of Karlsruhe and Forschungszentrum Karlsruhe GmbH, 1989-2007, TURBOMOLE GmbH, since 2007; available from http://www.turbomole.com.

(36) Neese, F. *Wiley Interdiscip. Rev. Comput. Mol. Sci.* **2012**, *2* (1), 73–78.

(37) ChemCraft https://www.chemcraftprog.com.

(38) Grimme, S. *J. Chem. Phys.* **2003**, *118* (20), 9095–9102.
